# Benzene with Alkyl Chains Is a Universal Scaffold for Multivalent Virucidal Antivirals

**DOI:** 10.1021/acscentsci.4c00054

**Published:** 2024-04-04

**Authors:** Yong Zhu, Matteo Gasbarri, Soumaila Zebret, Sujeet Pawar, Gregory Mathez, Jacob Diderich, Alma Delia Valencia-Camargo, Doris Russenberger, Heyun Wang, Paulo Henrique Jacob Silva, Jay-ar B. Dela Cruz, Lixia Wei, Valeria Cagno, Christian Münz, Roberto F. Speck, Daniel Desmecht, Francesco Stellacci

**Affiliations:** a Institute of Materials, École Polytechnique Fédérale de Lausanne, Station 12, 1015 Lausanne, Switzerland; b Institute of Microbiology, University Hospital of Lausanne and University of Lausanne, Rue du Bugnon 48, 1011 Lausanne, Switerland; c Institute of Experimental Immunology, University of Zürich, Winterthurerstrasse 190, CH-8057 Zürich, Switzerland; d Department of Infectious Diseases and Hospital Hygiene, University Hospital Zurich, University of Zurich Rämistrasse 100, 8091 Zurich, Switzerland; e Faculty of Veterinary Medicine Department of Pathology, University of Liège, Sart Tilman B43a, 4000 Liège, Belgium

## Abstract

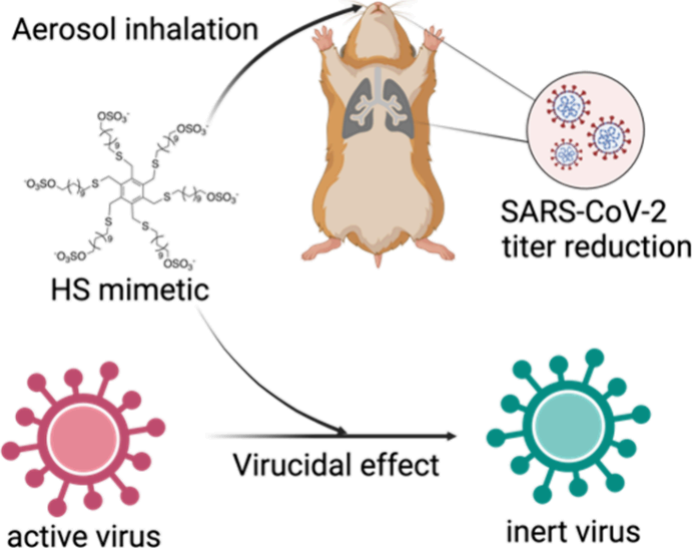

Most viruses start their invasion by binding to glycoproteins’ moieties on the cell surface (heparan sulfate proteoglycans [HSPG] or sialic acid [SA]). Antivirals mimicking these moieties multivalently are known as broad-spectrum multivalent entry inhibitors (MEI). Due to their reversible mechanism, efficacy is lost when concentrations fall below an inhibitory threshold. To overcome this limitation, we modify MEIs with hydrophobic arms rendering the inhibitory mechanism irreversible, i.e., preventing the efficacy loss upon dilution. However, all our HSPG-mimicking MEIs only showed reversible inhibition against HSPG-binding SARS-CoV-2. Here, we present a systematic investigation of a series of small molecules, all containing a core and multiple hydrophobic arms terminated with HSPG-mimicking moieties. We identify the ones that have irreversible inhibition against all viruses including SARS-CoV-2 and discuss their design principles. We show efficacy *in vivo* against SARS-CoV-2 in a Syrian hamster model through both intranasal instillation and aerosol inhalation in a therapeutic setting (12 h postinfection). We also show the utility of the presented design rules in producing SA-mimicking MEIs with irreversible inhibition against SA-binding influenza viruses.

## Abstract 2

Multivalent sulfated benzene derivative inhibits viruses through a virucidal mechanism. It shows therapeutic efficacy *in vivo* against SARS-CoV-2 when administered intranasally 12 h postinfection.

## Introduction

Viruses have evolved their means of attachment to the receptors on the host cells to initiate infection.^1^ The majority of the virus receptors target proteins, mostly in a virus-specific way.^2^ In contrast, there exist only a few known virus receptors that target carbohydrates, but such targets are very common in viruses and are usually shared across multiple virus species. Heparan sulfate (HS) is an unbranched sulfated glycosaminoglycan (GAG) expressed ubiquitously on the cell surface. It interacts with various proteins to regulate cell adhesion, migration, organogenesis, blood clotting, inflammation, tumor metastasis, and responses to injury.^3^ It has been reported to mediate the entry of a very large number of viruses, such as herpes simplex virus-2 (HSV-2),^4,5^ SARS-CoV-2,^6,7^ HIV,^8^ and Kaposi’s sarcoma-associated herpesvirus (KHSV).^9^ Another type of cell-surface carbohydrate involved in viral invasion is sialic acids, a diverse family of nine-carbon monosaccharides commonly found at the outermost end of the sugar chains attached to proteins and lipids. *N*-acetylneuraminic acid (Neu5Ac) is not only the most prevalent type of sialic acid but also the only type present in humans. Although Neu5Ac can bind to a variety of viruses such as reovirus,^10^ rotavirus,^11,12^ enterovirus,^13^ cardiovirus,^14^ and protoparvovirus,^15^ it is mostly studied to facilitate the binding to the hemagglutinin (HA) of influenza viruses, including influenza A and B. In human and other mammalian hosts, HAs preferentially bind to Neu5Ac-α-(2,6)-Gal trisaccharide (6SLN).^16^

The fact that these two types of carbohydrates bind to so many different viruses has been used as means to create broad-spectrum antivirals.^17−19^ The principle is that a molecule mimicking one of these carbohydrates should bind to the virus and prevent the initial step of the viral binding, which is indeed believed to be the recognition of the virus by these carbohydrates on the cell membrane. To achieve this goal these carbohydrate-mimicking molecules must have strong binding to the virus. It has been shown that molecules composed of a scaffold functionalized with multivalent units display dramatically higher affinity when compared to a single carbohydrate unit.^20,21^ As HS mimicking multivalent entry inhibitors (MEI), natural sulfated polysaccharides, including cellulose sulfate,^22^ carrageenan,^23^ as well as the synthesized polyaromatic sulfonate PRO2000,^24^ have demonstrated picomolar to micromolar inhibition^22−24^ against HS-dependent viruses. Highly multivalent nanographenes have achieved outstanding inhibition at femtomole concentrations.^25^ These molecules have consistently shown large therapeutic windows mainly due to their limited toxicity and are all broad-spectrum, however, none of them have successfully passed the phase III clinical trials yet.^26^ One of the primary reasons for the failure is believed to be the loss of efficacy once the molecule dilutes in body fluids below its inhibitory concentration.^26^ This is a limitation for all extracellular antiviral molecules whose inhibitory mechanism is reversible (virustatic mechanism). There exists a class of extracellular antivirals with irreversible inhibition, known as virucidal molecules, which typically damage the structural integrity of viral particles. However, they have the significant limitation of being overly toxic. Often, in the literature, reports on viral entry inhibitors, not only for HS mimetics but also for sialic acid mimetics, do not contain tests or information on whether the molecules are virustatic or virucidal despite the significant difference in translation potential between the two mechanisms.^27,28^ Yet, we believe that the vast majority of reported MEIs are virustatic.

Recently, we developed a strategy to modify glycan-mimicking virustatic molecules so as to render them virucidal while keeping the original low toxicity profile.^29−32^ For example, Sarid et al.^33^ had shown a gold nanoparticle core coated with 2-mercaptoethanesulfonic acid (MES) ligands as an effective HS mimic, exhibiting the same antiviral properties that have been known for years for heparin^34^ and its many mimics.^22−24^ Indeed, these particles have reversible broad-spectrum antiviral effects against HSV-1^33^ and influenza viruses.^35^ To render these particles virucidal, we retained the gold core and introduced a longer HS-mimicking hydrophobic ligand: 11-mercapto-1-undecane sulfonic acid (MUS);^29^ the resulting particles retained the nanomolar^35^ broad spectrum and the low toxicity profile but showed clear virucidal inhibition. *In vivo* data against RSV^29^ corroborated the efficacy of this design. Then, we changed the gold core into a β-cyclodextrin^30^ functionalized on the primary face with the same ligands used for the nanoparticles to improve biocompatibility while retaining a similar broad-spectrum and virucidal profile. The resulting molecule showed the expected properties with *in vivo* efficacy against HSV-2, albeit with only micromolar inhibitory concentrations. We then used a dendritic polyglycerol^31^ to increase the multivalency while keeping an all-organic core. The resulting macromolecules were broad-spectrum and virucidal and had nanomolar inhibition. To develop virucidal anti-influenza materials, in our modified β-cyclodextrins, we replaced the sulfonic^29^ and sulfate^31^ end groups with sialic-acid-mimicking 6′SLNs^32^ while keeping the rest of the molecule the same, including the hydrophobic linkers. The resulting modified cyclodextrin showed nanomolar inhibition with a virucidal mechanism against a few strains of influenza, as well as *in vivo* efficiency against pandemic H1N1/California/09 when given 24 h postinfection.

Despite these successes, there are still many open questions. First, all the designed virucidal materials used to date have been based on nanoparticles and macromolecules, and no molecularly defined compound has been explored. Second, all studies to date have focused on the linkers and the targeting moieties with little attention to the role of the core. Finally, it is important to state that all the described HS-mimicking molecules showed potent inhibition against SARS-CoV-2 but surprisingly only with a reversible mechanism (virustatic).^36^ Although the previously mentioned HS entry inhibitors showed *in vivo* efficacy against HSV-2^30^ and RSV,^29^ to the best of our knowledge, there is currently no HS mimetic with virucidal properties that has demonstrated efficacy against SARS-CoV-2 *in vivo*. To address all of these issues, we decided to investigate smaller and simpler scaffolds to enable a precise and systematic study of the structure–antiviral activity relationship of molecularly defined compounds where the role of the core and the linkers could be determined. This also enabled us to find MEIs with irreversible inhibition of SARS-CoV-2. Here, we present these antiviral small molecules. We evaluate how different cores and linkers differing in length, multivalency, and hydrophobicity affect viral inhibition and the virucidal property. The most promising candidate, a benzene core modified with alkyl sulfates, demonstrated broad-spectrum virucidal activity against SARS-CoV-2, H1N1, HIV, EBV, and KHSV *in vitro* and efficacy against SARS-CoV-2 in a Syrian hamster model. We also assessed the benzene scaffold versatility by replacing the end group with an SA mimicking group and showing their efficacy against H1N1.

## Results and Discussion

**Figure 1:**
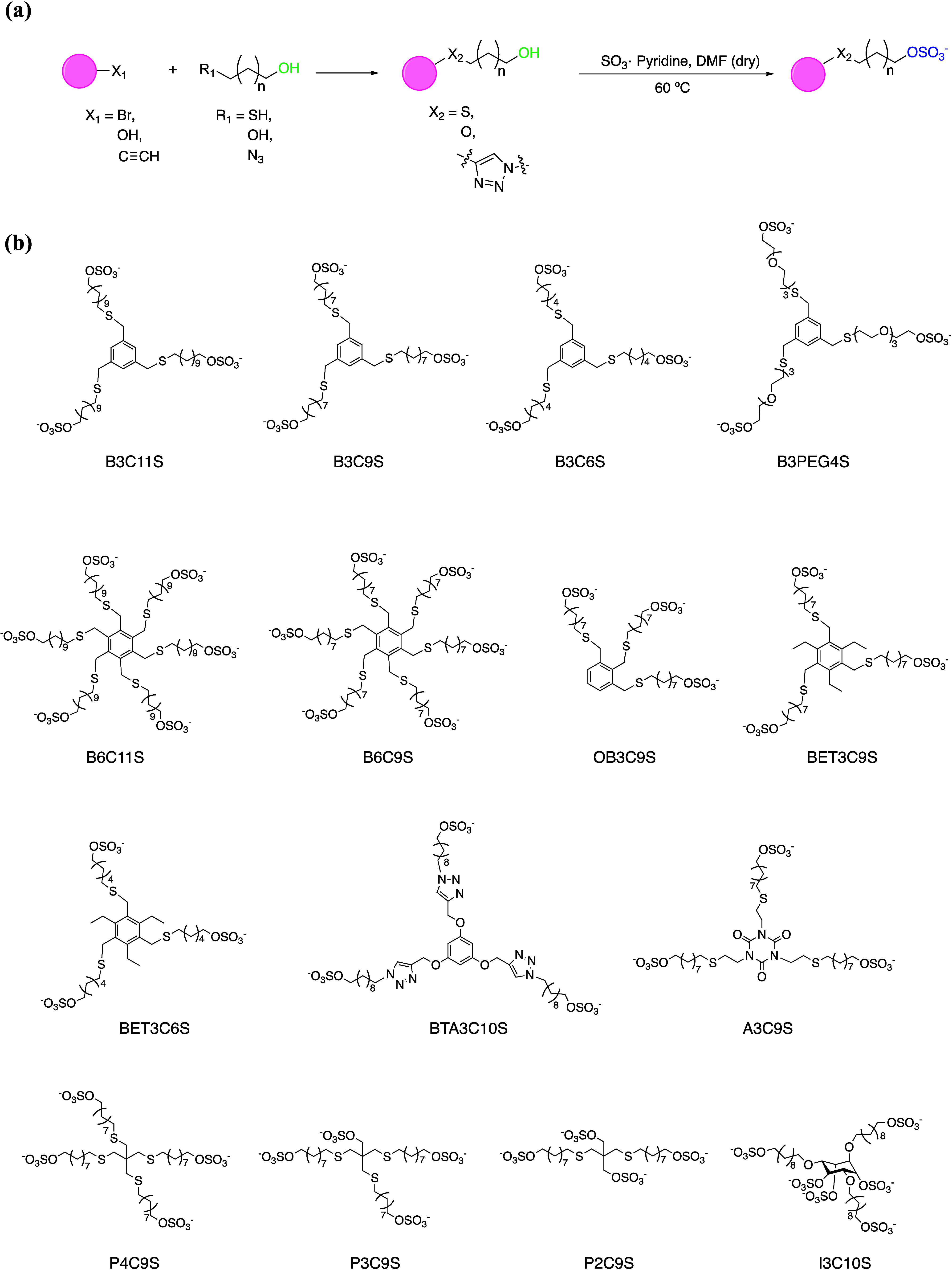
(a) General scheme for the synthesis of the molecules whose structure is shown in (b).

We synthesized a series of molecules all with the same architecture, namely a core and a small number (three to six) of alkyl sulfate ligands. We used a general synthetic route, which starts with Cu(I)-catalyzed azide–alkyne cycloaddition or a nucleophilic substitution, either between a bromide core and thioalcohol, or between a hydroxyl core and a diol linker to produce the multivalent alcohol (Figure 1a). The versatility of the first reactions provides various core structures and enables control over the ligands’ number, position, and density by changing the core or linker precursors. The second step, the sulfation with SO_3_-pyridine, efficiently converts all the hydroxyls into sulfates. Using this strategy, we managed to obtain a library of molecularly defined multivalent organic sulfates (Figure 1b; detailed synthesis methods, yields, and analytical data are presented in the Supporting Information Section 1).

We evaluated the half-maximal viral inhibition concentrations (EC_50_) and the virucidal activity of the synthesized multivalent organic sulfates against the HS-binding HSV-2 (Table 1). The 99%-maximal viral inhibition concentrations (EC_99_) of the compounds were applied for virucidal titration to examine the (ir)reversibility of the inhibition. Half-maximal cytotoxicity concentrations (CC_50_) were measured on Vero cells as well. The first molecule we studied was B3C11S (B = benzene, 3 = three linkers, C11 = an 11-methylenes-long linker, S = sulfate) that demonstrated inhibitory effects on HSV-2 at 26.2 μM and displayed a virucidal mechanism (Table 1 and Figure S1) indicating that the design of such small molecules leads to antiviral properties similar to the ones we reported on larger ones.^29−31^ We focused on analyzing the impact of scaffold linkers on antiviral activity. As shown in Table 1, benzene-bearing molecules with relatively long alkyl linkers (from nine to 11 methylenes; B3C11S, B3C9S, B6C11S, B6C9S, OB3C9S, BET3C9S, and BTA3C10S) showed potent *irreversible* inhibition (EC_50_ ranging from 3 to 30 μM), while B3C6S, a benzene-bearing molecule with shorter alkyl linkers (six methylenes long) inhibited the virus only *reversibly* with a significantly higher EC_50_ (78.4 [95% CI, 70.4–86.6] μM). The latter was modified by adding three extra ethyl linkers to the benzene core. The resulting molecule (BET3C6S) showed better efficacy (EC_50_ = 28.5 [95% CI, 24.7–33.1] μM) than the original one (B3C6S, EC_50_ = 78.4 [95% CI, 70.4–86.6] μM) through a virucidal mechanism. This suggests that what leads to a virucidal mechanism is the total amount of hydrophobicity of the molecules, not just the hydrophobicity of the linkers. No inhibition was observed for B3PEG4S, a benzene sulfate with a hydrophilic oligo (ethylene glycol) linker, even at 400 μM. These data demonstrate the importance of the hydrophobic chain to the antiviral potency as well as on the virucidal mechanism. On the other hand, the substitution position (B3C9S vs OB3C9S) and the number of linkers (B3C11S vs B6C11S, B3C9S vs B6C9S) had only a very subtle effect on the inhibition and cytotoxicity at the limit of the resolution of our measurements.

**Table 1:** HSV-2 Inhibition and Virucidal Activity of Multivalent Organic Sulfates

	HSV-2			
compound	EC_50_ μM (95% CI)	virucidal	CC_50_ μM (95% CI)	SI
B3C11S	26.2 (22.5–30.8)	Y	384 (367–399)	15
B3C9S	7.92 (6.80–9.12)	Y	331 (261)	42
B3C6S	78.4 (70.4–86.6)	N	>530	>7
B3PEG4S	>400	NT^a^	NT	NT
B6C11S	2.59 (2.16–3.06)	Y	200 (191–208)	77
B6C9S	14.0 (12.9–15.1)	Y	42.7 (−50.5)	3
OB3C9S	18.0 (17.2–18.6)	Y	135 (125–144)	8
BET3C9S	13.1 (12.1–14.0)	Y	81.6 (66.4–108)	6
BET3C6S	28.5 (24.7–33.1)	Y	>477	>17
BTA3C10S	17.1 (15.5–18.5)	Y	>371	>22
P4C9S	6.48 (6.01–6.92)	Y	185 (168–204)	29
P3C9S	24.4 (20.3–28.6)	Y	522 (−676)	21
P2C9S	326 (293–359)	Y	NT	NT
A3C9S	20.1 (18.6 – 21.9)	Y	221 (207–236)	11
I3C10S	163 (136–196)	N	>359	>2

a NT denotes not tested.

We also investigated the role of the cores in the antiviral activity. P4C9S, a neo-pentane-based molecule, showed strong virucidal inhibition (EC_50_ = 6.5 [95% CI, 6.01–6.92] μM) comparable to that of benzene-based B3C9S (EC_50_ = 7.9 [95% CI, 6.80–9.12] μM, Table 1), indicating the core rigidity does not contribute to the antiviral activity. Also for the neo-pentane core, when one or two linkers were replaced with a sulfate (P4C9S vs P3C9S or P2C9S) the antiviral mechanism remained virucidal, accompanied by an increase in EC_50_ (24.4 [95% CI, 20.3–28.6] μM and 326 [95% CI, 293–359] μM, respectively). The core can nevertheless play a role in determining the mechanism. In fact, when we used inositol as a core (I3C10S), we achieved a decent EC_50_ (163 [95% CI, 136–196] μM) but only with a virustatic mechanism. I3C10S has a lower inhibitory concentration when compared to the virucidal P2C9S, indicating that the inhibitory concentrations and the virucidal mechanism are not necessarily linked.

We also investigated the impact of the virus-binding group that mimics heparan sulfate. We synthesized a multivalent sulfonate molecule, B3C11Sulfonate, which possesses the same scaffold structure as B3C11S (Figure 1b) but differs only in the sulfonate/sulfate terminal group. The synthesis involved modifying sodium 11-mercaptoundecane-1-sulfonate to 1,3,5-tris(bromomethyl)benzene (details provided in Supplementary Section 2). B3C11Sulfonate exhibited an EC_50_ of 115 (95% CI, 90.2–141) μM against HSV-2 as opposed to the 26.2 (95% CI, 22.5–30.8) μM found for B3C11S. Interestingly, the inhibition mechanism of the two molecules differed, with B3C11Sulfonate being virustatic and B3C11S being virucidal. This surprising result points to the fact that the virucidal mechanism cannot be trivialized in a hydrophobic effect as very similar molecules show drastically different antiviral effects (Figure S2). This data is markedly different from what happens on a cyclodextrin scaffold. On such a scaffold, both CD-M11Sulfate (for detailed synthesis and characterization, see Supplementary Section 3) and CD-M11Sulfonate^30^ are virucidal against HSV-2 (Figure S3), with very similar EC_50_ values (28.6 and 28.5 μg/mL,^30^ respectively).

Among the tested compounds in the library, B6C11S displayed the lowest EC_50_ and the highest selectivity index (Table 1) and was therefore selected as the best anti-HSV-2 candidate and used in the subsequent antiviral mechanism study.

First, we focused on better establishing the extracellular mechanism of action of these molecules. To do so, we performed the inhibition assay under four different conditions as follows:

(i) Cell pretreatment: incubating B6C11S with the cells for 1 h prior to inoculation with the virus.
(ii) Virus pretreatment: incubating B6C11S with the virus for 1 h prior to inoculation with the mixture.
(iii) Cotreatment: simultaneous addition of B6C11S and the viral inoculum onto the cells.
(iv) Post-treatment: addition of B6C11S 1 h after inoculation of the cell with the virus.

**Figure 2:**
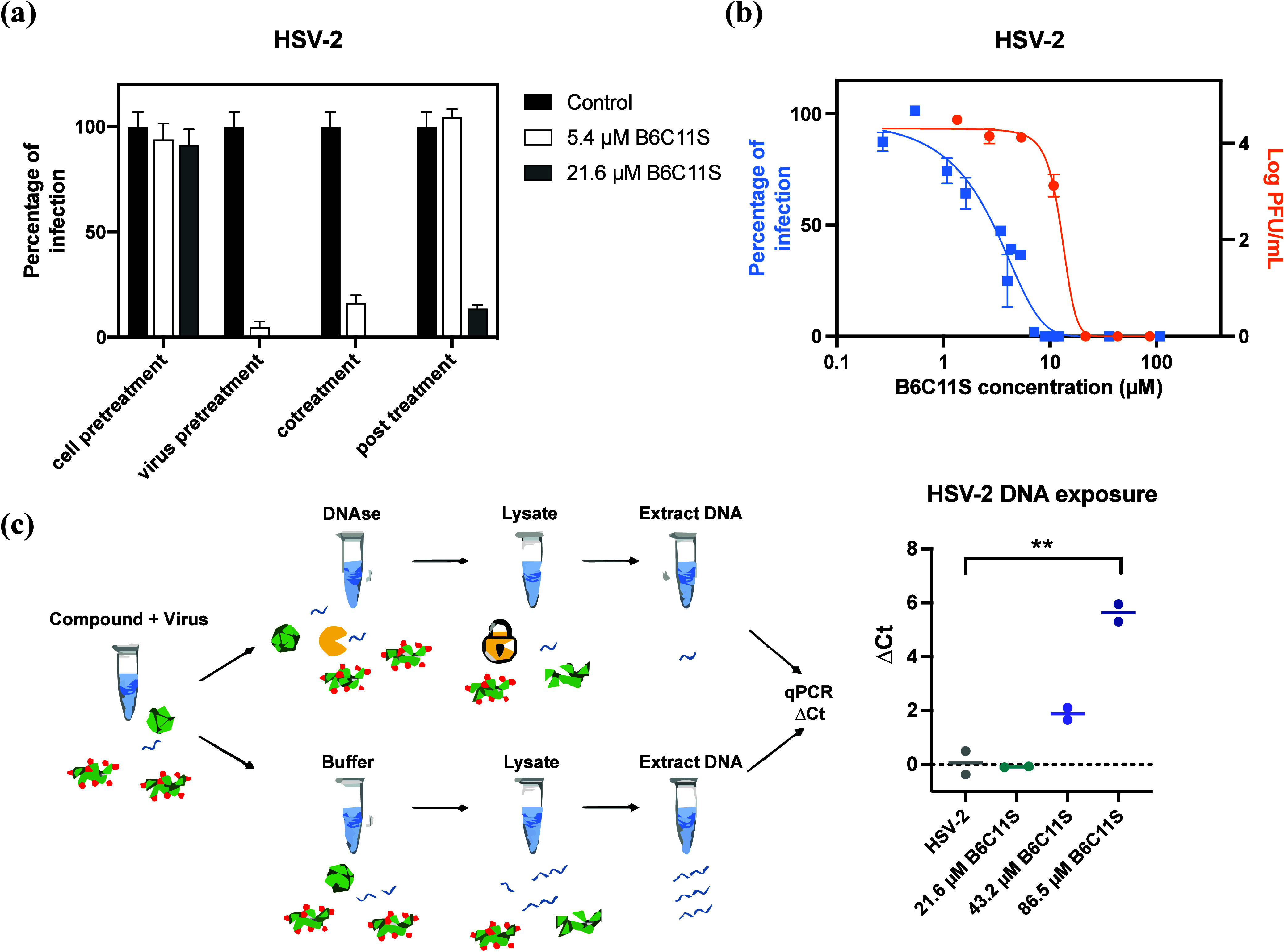
Antiviral mechanism of B6C11S. (a) Plot of the percentage of HSV-2 infection (*n* = 2) with 5.4 and 21.6 μM B6C11S in the cell pretreatment, virus pretreatment, cotreatment and post-treatment. (b) Percentages of viral infection (*n* = 2) and infectious viral titers (*n* = 2) at different B6C11S concentrations. (c) Schematic drawing demonstrating the mechanism of the DNA exposure assay (adapted from ref (37) with permission) and log_2_ DNA fold change (ΔCt = Ct_DNase_ – Ct_control_) of HSV-2 samples incubated with different concentrations of B6C11S. Error bars represent standard errors of the mean, and averages are plotted. A two-tailed unpaired *t* test was used to analyze the data, and the statistical significance is indicated by asterisks (*, <0.05; **, <0.01; ***, <0.001).

As shown in Figure 2a, we found that B6C11S decreased the percentage of infected cells by over 85% under conditions (ii) and (iii) already at 5.4 μM and by 85% under condition (iv) at 21.6 μM. In contrast, treating the cells in the absence of the virus [condition (i)] showed no inhibition even at 21.6 μM, which strongly suggests that B6C11S affects the virus particles only extracellularly and has little effect on the cells. The demonstrated post-treatment efficacy at 21.6 μM also suggests the molecule’s potential utilization in infection scenarios with multiple cycles of replication, given its ability to deactivate the extracellular progeny.

We then focused on investigating the virucidal mechanism. Figure 2b shows the inhibitory curve obtained for this compound (blue), performed with a standard dose–response assay, as well as the viral titer found as a result of virucidal assays performed at many initial concentrations (see Supporting Information Section 4 for a description of this assay). If the two curves overlap, it means that whatever effect the compound has on the virus will be virucidal. We observe a significant difference between the two curves, implying that the compound has a virustatic (reversible) effect at lower concentrations and it is only at higher concentrations, after binding (virustatic), that the compound is able to irreversibly modify the virus (virucidal). The difference between the half inhibitory concentrations (EC_50 virustatic_ = 2.6 μM, EC_50 virucidal_ = 12.8 μM) indicates that to have a virucidal effect, one needs a higher concentration of molecules when compared to the virustatic effect.

To determine whether the virucidal effect is caused by the damage to the viral particles, we performed DNA release assays. Briefly, we incubated HSV-2 and B6C11S mixtures with DNase or buffer, inactivated the DNase, and then quantified the DNA amounts in the two samples (Figure 2c). The results showed that B6C11S induced the release of viral DNA at virucidal concentrations of 43.2 and 86.5 μM. This indicates that B6C11S is capable of damaging the viral envelope as well as its capsid, eventually leading to the release of the DNA.

**Figure 3:**
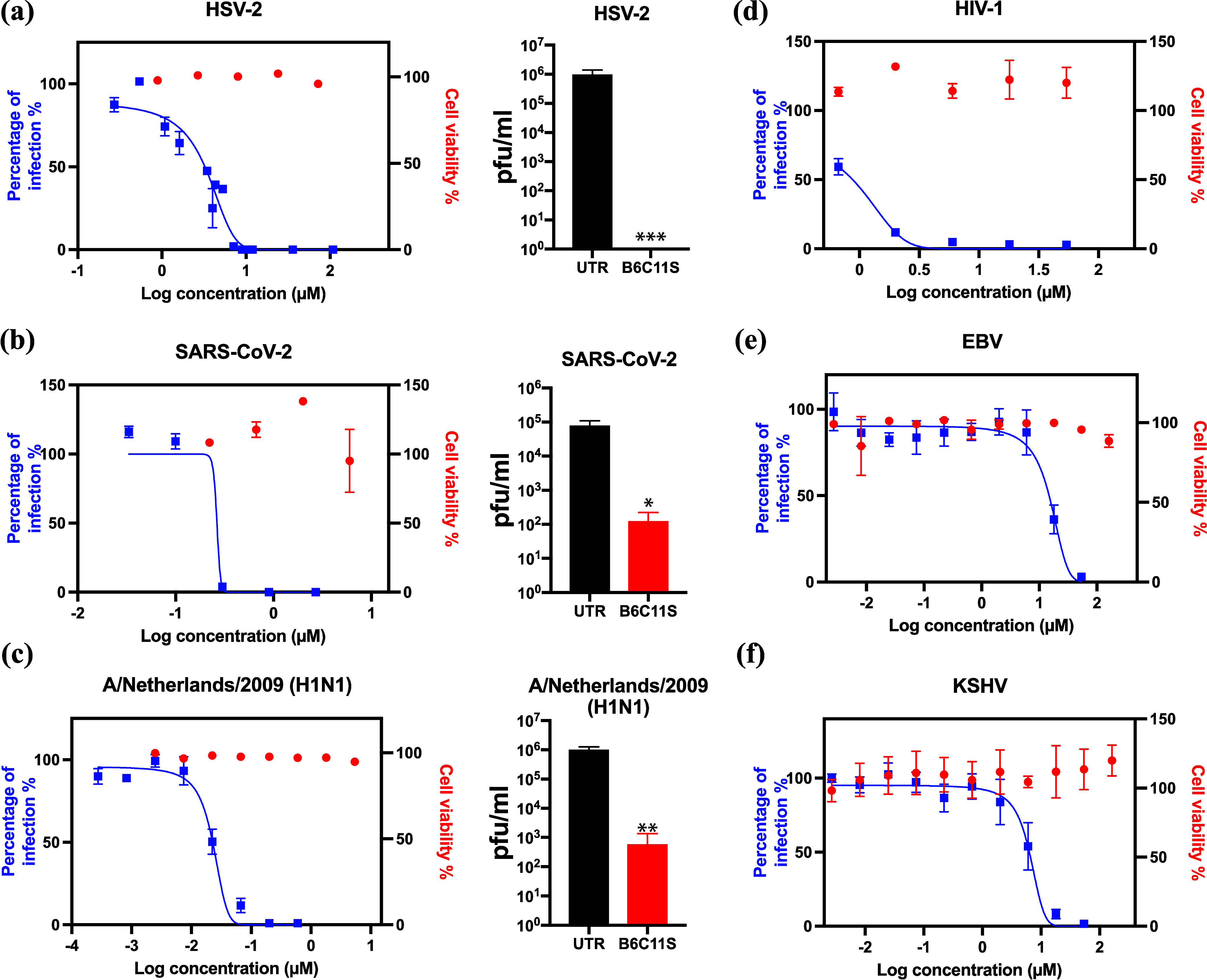
B6C11S viral inhibition curves and virucidal results (bar charts of infectious viral titer in B6C11S-treated and untreated virus samples) on HSV-2 (a), SARS-CoV-2 (b), and H1N1 (c). B6C11S viral inhibition curves on HIV-1 (d), EBV (e), and KSHV (f). Results are expressed as averages and standard errors from two independent experiments conducted in duplicate (a, b, and c) or triplicate (d, e, and f). Statistical significance was analyzed with a two-tailed unpaired *t* test. The asterisks represent the *P* value (*, <0.05; **, <0.01; ***, <0.001).

Overall, the results discussed so far are in line with what was previously presented for similar yet larger compounds.^29−31^ We tested all the multivalent sulfates on other HSPG-dependent viruses, including SARS-CoV-2 (Alpha and Omicron strain), HIV-1 and Kaposi’s sarcoma-associated herpesvirus (KSHV), as well as HSPG-independent Epstein–Barr virus (EBV) and H1N1 (A/Netherlands/2009), and the results are depicted in Figures S4–S8 and summarized in Table S1. As shown in Figure 3 and Table 2, B6C11S shows nanomolar to micromolar inhibition against all the tested viruses and exhibits virucidal effects against HSV-2, SARS-CoV-2 Alpha, and H1N1. B6C11S’s broad antiviral activity beyond HSPG-dependent viruses may be attributed to the nonspecificity of charge-based interactions between sulfate groups and basic amino acids^3,7^ on the viral protein. Although EBV and H1N1 do not rely on HSPG for cell entry, it has been found to possess a high binding affinity^38,39^ for sulfated glycans. In addition, both sulfated glycans^40^ and their mimetics^41^ demonstrate inhibitory effects against influenza. Importantly, in stark contrast to our previously published compounds that exhibited only virustatic inhibition^36^ against SARS-CoV-2, B6C11S has a virucidal mechanism against it. This property is shared with all the other molecules that we tested that showed virucidal mechanisms against HSV-2 (Table S1) with the exception of two of them (BET3C9S and P3C9S). At present we do not have an explanation for these findings, as our results paint a complex picture that deserves further in-depth investigation.

**Table 2:** Viral Inhibition, Cytotoxicity, and Selectivity Index of B6C11S on Different Viruses

virus	EC_50_ μM (95% CI)	virucidal	CC_50_ μM (95% CI)	SI
HSV-2	2.59 (2.16–3.06)	Y	200 (191–208)	77
SARS-CoV-2	0.24 (0.001–0.79)	Y	17.5 (7.7–42.4)	73
HIV-1	0.79 (0.73–0.86)	NT^a^	>54	>68
EBV	13.7 (9.56–19.2)	NT	>162	>12
KSHV	5.99 (4.22–8.28)	NT	>162	>27
H1N1	0.023 (0.019–0.028)	Y	>5.4	>235

a NT denotes not tested.

**Figure 4:**
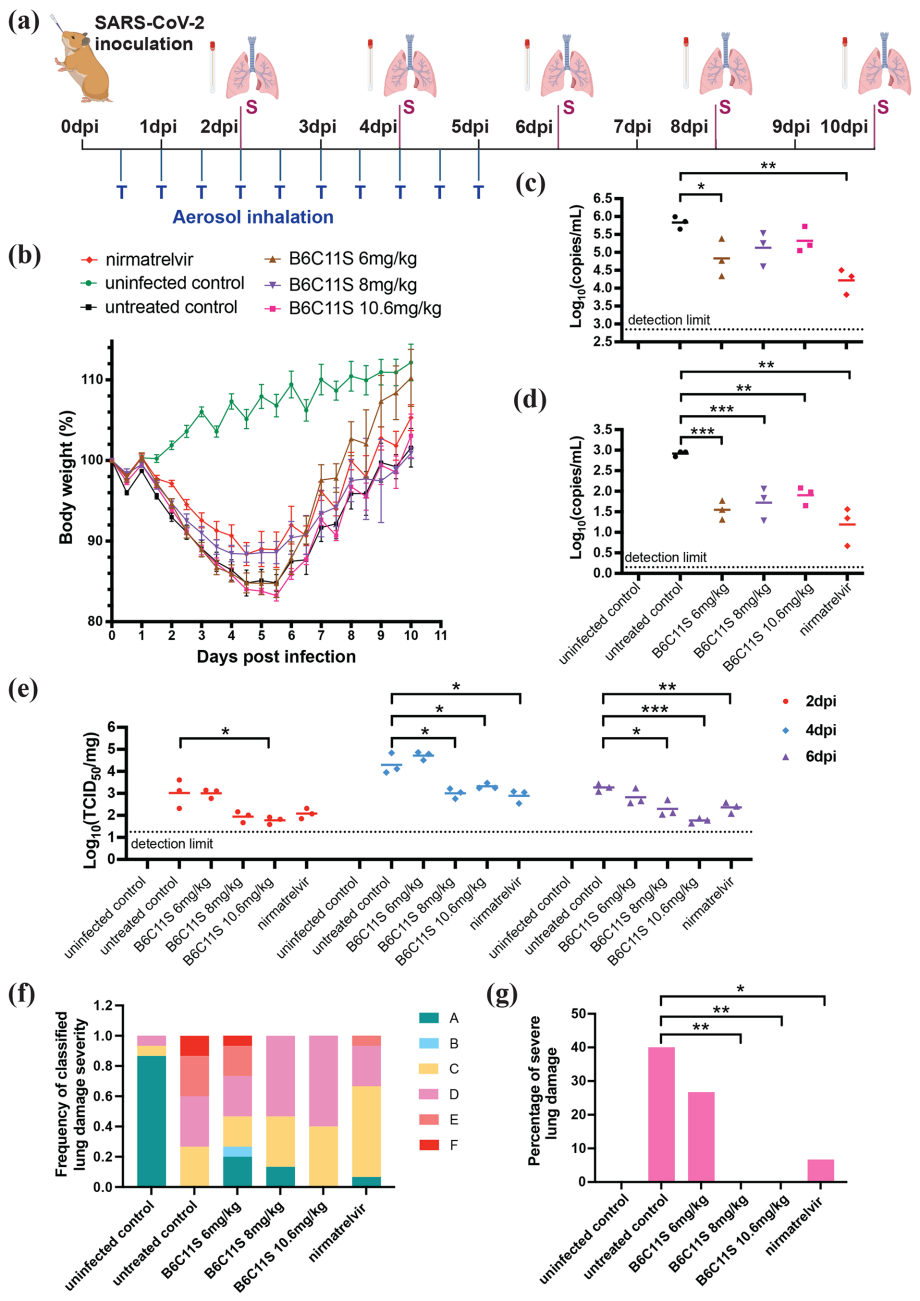
*In vivo* efficacy of B6C11S against SARS-CoV-2 infection in Syrian hamsters (*n* = 15). (a) Schematic drawing of study design. (b) Body weight loss of uninfected, untreated, nirmatrelvir-treated, and B6C11S-treated hamsters over time. Results are presented as mean percentages of initial body weight ± SEM. Viral RNA levels in the oral swab (c) and lung (d) of uninfected, untreated, nirmatrelvir-treated and B6C11S-treated hamsters at day 8 postinfection were determined by RT-qPCR. Infectious viral titer (TCID_50_) in the lung samples at days 2, 4, and 6 postinfection are presented in (e). Data in (c), (d), and (e) were analyzed with a two-tailed unpaired *t* test. Error bars represent standard errors of the mean, and averages are plotted. Histopathological evaluation was performed on lung tissues from all hamsters, with ratings from A to F (A indicating minimal damage, F indicating maximum damage), considering lesions, inflammatory patterns, cellular damage, regeneration, and healing. (f) Distribution of lung damage categories across various groups (*n* = 15). (g) Percentage of severe lung damage (E and F) among different groups. *P* values were computed using a χ^2^ test, followed by Benjamini–Hochberg correction for multiple testing. Asterisks indicate significance levels (*, < 0.05; **, < 0.01; ***, < 0.001).

To assess the *in vivo* effectiveness of virucidal B6C11S against the SARS-CoV-2 Alpha variant, we infected Syrian hamsters with 10^5^ TCID_50_ of the virus. The hamsters were divided into groups (*n* = 15), and three doses of B6C11S (6, 8, or 10.6 mg/kg/day) were given intranasally through aerosol inhalation, twice per day for five days starting from 12 hpi. A positive control group was given FDA-approved nirmatrelvir^42,43^ (250 mg/kg) orally at the same time. Hamster bodyweight was monitored twice daily, and three hamsters in each group were sacrificed at 2, 4, 6, 8, and 10 dpi (Figure 4a) to measure viral titers in oral swabs and lung tissues. Treatments with 6 and 8 mg/kg B6C11S decreased the body weight change of infected hamsters (Figure 4b), while the 10.6 mg/kg dose showed no significant change compared to the untreated group; the histopathology data discussed below does not show a clear reason for this behavior. We did not find any notable differences between the treated groups (both for B6C11S and nirmatrelvir) and the untreated groups in viral titers in either the swab samples or lung tissues at 2, 4, 6, and 10 dpi (Figure S9). However, at 8 dpi (Figure 4c), both the nirmatrelvir (*P* = 0.0021) and 6 mg/kg B6C11S (*P* = 0.035) groups showed significantly lower viral RNA levels than the untreated group. In lung samples at the same time point at 8 dpi (Figure 4d), all the B6C11S groups (*P* = 0.0003 for 6 mg/kg, 0.0006 for 8 mg/kg, and 0.0068 for 10.6 mg/kg) as well as the nirmatrelvir group (*P* = 0.0031) showed significantly decreased viral RNA copies. The infectious viral titer (TCID_50_) in the lungs exhibited temporal changes (Figure 4e), dropping below the limit of detection at 8 and 10 dpi. At 2 dpi, only the high dose of 10.6 mg/kg B6C11S demonstrated a significant reduction in the infectious viral load (*P* = 0.033), while no significant difference was observed between the nirmatrelvir group and the untreated control (*P* = 0.079). At 4 and 6 dpi, both the middle and high doses of B6C11S, along with nirmatrelvir, significantly decreased the viral titer (*P* = 0.013 and 0.014 for 8 mg/kg B6C11S at 4 and 6 dpi, respectively; 0.026 and 0.0002 for 10.6 mg/kg B6C11S at 4 and 6 dpi, respectively; and 0.012 and 0.0066 for nirmatrelvir at 4 and 6 dpi, respectively). Notably, there was a clear dose-dependent antiviral effect of B6C11S on the infectious viral titer at 6 dpi.

Histopathological evaluation of the lung tissues showed that both the B6C11S treatment and the nirmatrelvir resulted in improved pulmonary conditions in hamsters (Figure 4f). This effect is particularly significant for reducing severe lung damage (Figure 4g) with 8 mg/kg B6C11S (*P* = 0.006), 10.6 mg/kg B6C11S (*P* = 0.006), and nirmatrelvir (*P* = 0.03). In a separate experiment, we found that intranasal instillation of the B3C9S solution reduced the body weight loss of infected hamsters (Figure S10). B3C9S was also evaluated with aerosol treatment but was found to be less effective (Figure S11) than B6C11S. Overall, our findings demonstrate that B6C11S can effectively reduce viral titer and alleviate illness severity in hamsters infected with SARS-CoV-2 Alpha. In comparison to the main protease inhibitor nirmatrelvir, which exclusively inhibits human coronaviruses,^44^ B6C11S possesses the extra advantage of suppressing multiple respiratory viruses, including SARS-CoV-2, H1N1, and RSV.

To assess the potential of alkyl benzene as a scaffold for virucidal materials with other viral attachment ligands, we modified *N*-acetylneuraminic acid (sialic acid, SA) and 6-sialyl-N-acetyllactosamine (6SLN) (as depicted in Figure S12a) onto the alkyl benzene (synthesis, yields, and analytical data are presented in the Supporting Information Section 5). Both SA and 6SLN bind to the hemagglutinin (Figure S12b) of influenza virus.^45^ BTA3C10SA and B3C10SA have the same number of SA ligands and similar ligand spacing (Figure S12c), but B3C10SA exhibits stronger viral inhibition (EC_50_ = 20 μM, Table 3) against H1N1 compared to BTA3C10SA (EC_50_ = 66 μM, Table 3). This is probably because the extra three triazole groups on the scaffold of BTA3C10SA add a hydrophilic property to the core and make it less potent, as observed with multivalent sulfated molecules. By modifying a more potent viral attachment ligand 6SLN on the scaffold of B3C10SA, we obtained B3C106SLN with an EC_50_ of 0.32 μM and a selectivity index above 1000 (Table 3). As shown in Figure S12c, all three compounds are virucidal, suggesting that the benzene alkyl scaffold could also be applied for developing the virucidal compounds with other viral attachment ligands.

**Table 3:** Viral Inhibition, Cytotoxicity, and Selectivity Index of Benzene-Based Sialic Acids on Influenza A/Netherlands/2009 (H1N1)

compound	EC_50_ μM (95% CI)	virucidal	CC_50_ μM (95% CI)	SI
BTA3C10SA	66.2 (62.1–70.3)	Y	>260	>4
B3C10SA	20.2 (18.0–25.7)	Y	>565	>28
B3C106SLN	0.32 (0.17–0.60)	Y	>349	>1090

## Conclusion

In summary, a simple and robust synthetic route was developed to produce molecularly defined multivalent sulfates for studying the relationship between their structure and antiviral activity. We found that long (minimum methylene number > 6), hydrophobic linkers and a rather hydrophobic, uncharged core are essential for low EC_50_ values and irreversible viral inhibition. The position and number of linkers, however, do not appear to influence the antiviral potency of the multivalent sulfates. The most potent molecule B6C11S was found to inhibit several viruses, including HSPG-dependent HSV-2, SARS-CoV-2, HIV-1, KSHV, and HSPG-independent EBV and H1N1 in the nanomolar to micromolar range, without displaying cytotoxicity in the tested concentration range. Its antiviral mechanism on HSV-2 is independent of the host cell and involves damaging the viral envelope and releasing viral DNA. Furthermore, a few of the molecules tested showed irreversible inhibition of SARS-CoV-2 in stark contrast with the reversible inhibition of our previously published materials. B6C11S was found to reduce the body weight loss and oral swab and lung viral titers of infected Syrian hamsters to a similar level as the FDA-approved SARS-CoV-2 antiviral nirmatrelvir, indicating its potential for therapeutic use against SARS-CoV-2 *in vivo*. We also found that this benzene scaffold modified with other viral attachment ligands, such as sialic acids, exhibits an irreversible antiviral effect against sialic-acid-dependent viruses. Together, this study shows that the benzene alkyl scaffold is a universal platform for designing multivalent virucidal antivirals, as well as for studying in-depth how molecules can irreversibly inhibit viral infections.

## Supporting Information Available

Complete synthetic methods and characterization (NMR, MS) of the molecules; *in vitro* biological evaluation methods (dose–response antiviral assay, cytotoxicity assay, virucidal assay); methods of the SARS-CoV-2 hamster study (treatment regimen, viral RNA and infectious titer measurements, histopathology examination); comparison of *in vitro* antiviral efficacy of all multivalent sulfates; and supplementary *in vivo* antiviral results for both B3C9S and B6C11S (PDF)
