# Supplementary material for: Benzene with Alkyl Chains Is a Universal Scaffold for Multivalent Virucidal Antivirals
Source: ACS Cent Sci. 2024 Apr 4;10(5):1012–21. doi: 10.1021/acscentsci.4c00054 (PMC11117723; doi:10.1021/acscentsci.4c00054)
Supplement: Supplementary file 1 — oc4c00054_si_001.pdf [file oc4c00054_si_001.pdf]

Supporting information

Benzene with Alkyl Chains is a Universal Scaffold for Multivalent Virucidal Antivirals

Yong Zhu, Matteo Gasbarri, Soumaila Zebret, Sujeet Pawar, Gregory Mathez, Jacob Diderich, Alma Delia Valencia-Camargo, Doris Russenberger, Heyun Wang, Paulo Henrique Jacob Silva, Jay-ar B. Dela Cruz, Lixia Wei, Valeria Cagno, Christian Münz, Roberto Speck, Daniel Desmecht, Francesco Stellacci

Table of contents

Table of contents 0

Supplementary figures and tables 1

Materials and reagents for synthesis 10

1. Synthesis of multivalent sulfated antivirals 10

2. Synthesis of multivalent sulfonate B3C11Sulfonate 67

3. Synthesis of multivalent sulfonate CD-M11sulfates 68

4. *In vitro* antiviral and cytotoxicity assays 69

5. Synthesis of multivalent sialic acids B3TAC10SA 72

6. SARS-COV-2 infection model in Syrian hamsters 81

Ethics statement 82

7. Reference 83

Supplementary figures and tables

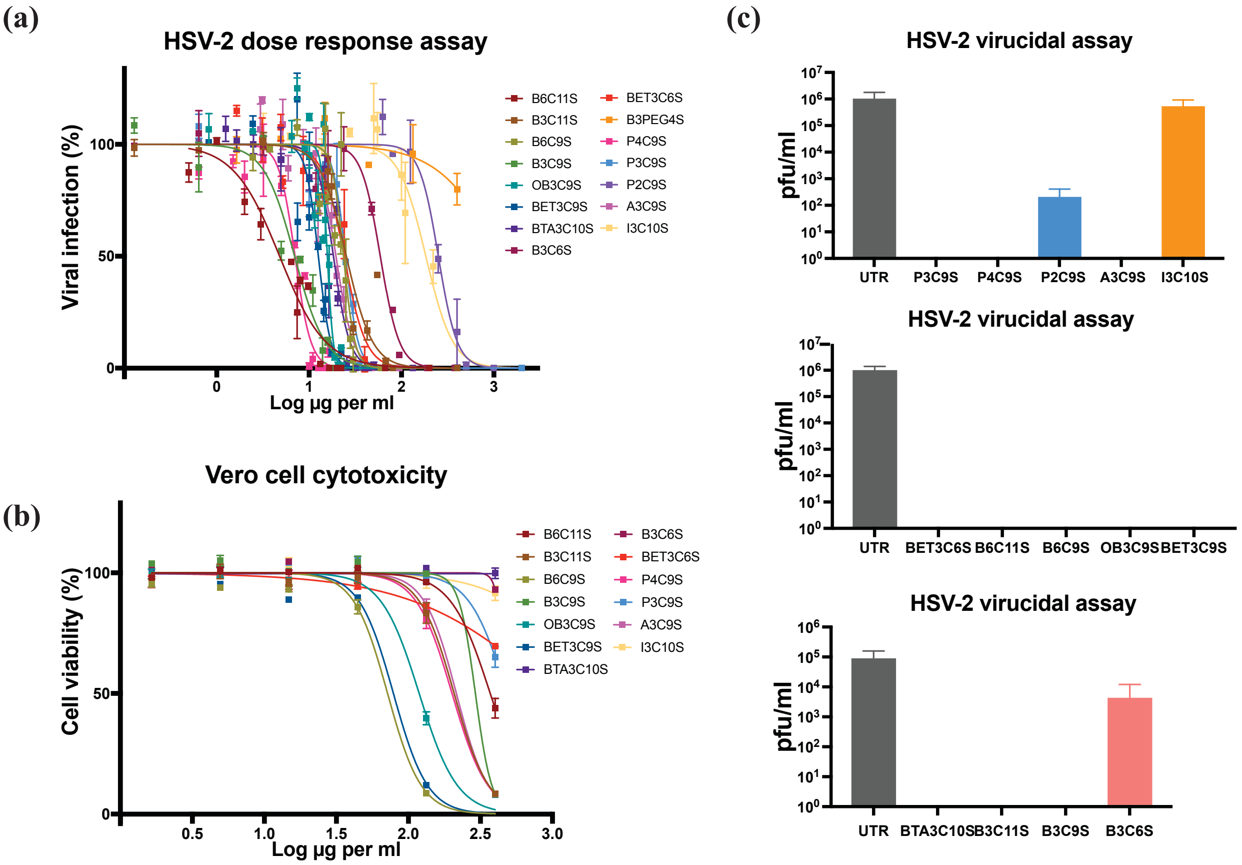

Figure S1. HSV-2 inhibition and cytotoxicity of multivalent sulfates on Vero cells

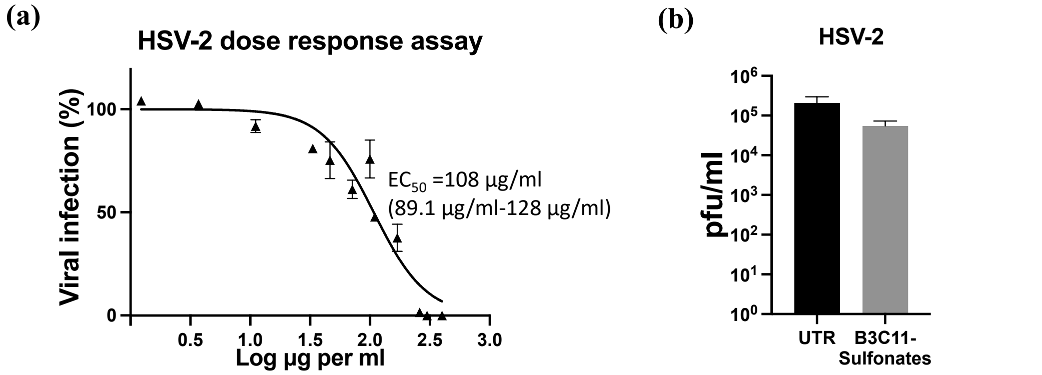

Figure S2. (a) HSV-2 inhibition and (b) virucidal results of B3C11Sulfonates on Vero cells.

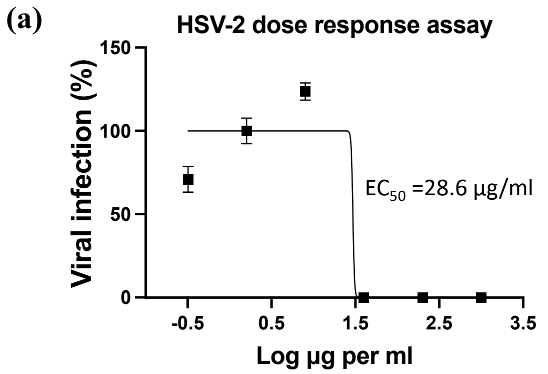

Figure S3. (a) HSV-2 inhibition and (b) virucidal results of CD-M11Sulfate on Vero cells.

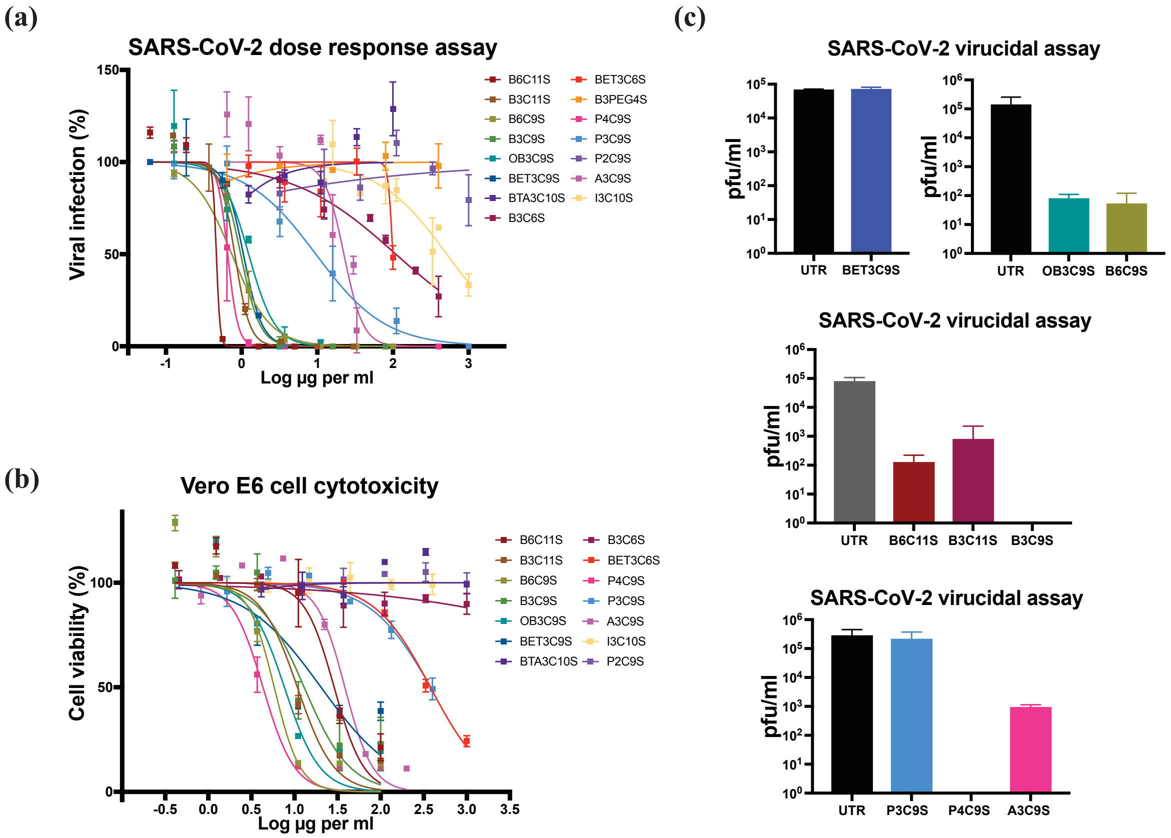

Figure S4. SARS-CoV-2 (alpha strain) inhibition and cytotoxicity of multivalent sulfates on Vero E6 cells.

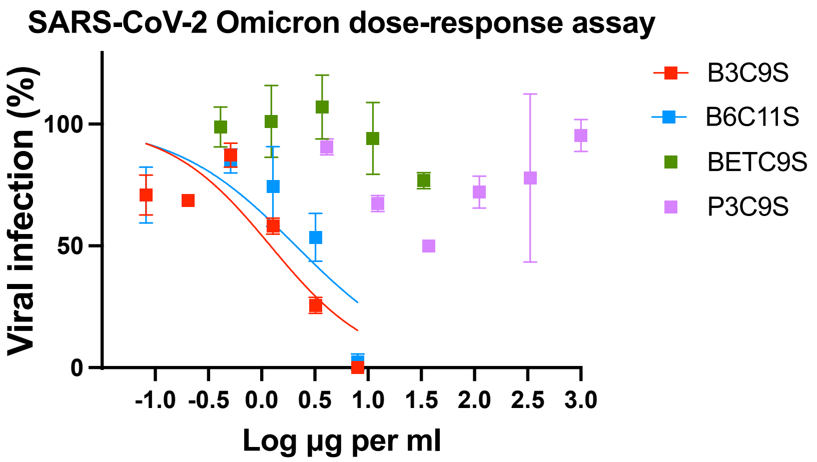

Figure S5. SARS-CoV-2 (Omicron strain) inhibition on Vero E6 cells.

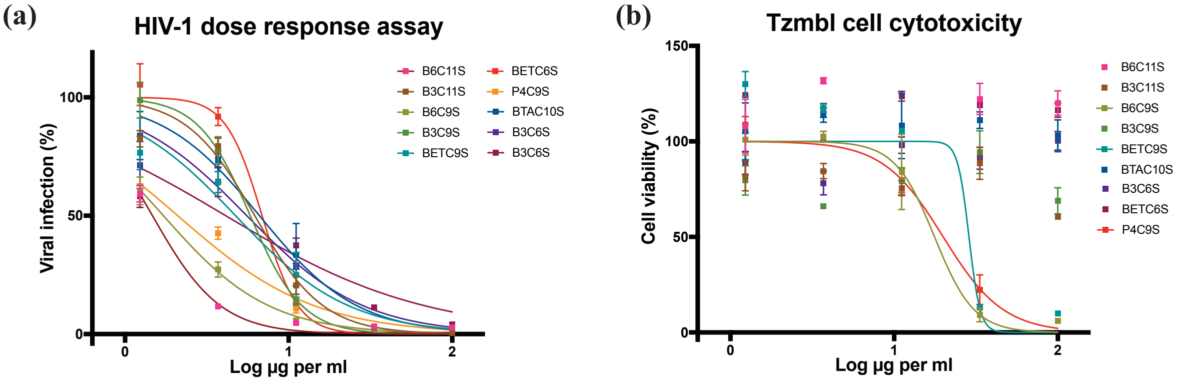

Figure S6. HIV-1 inhibition and cytotoxicity of multivalent sulfates on Tzmbl cells.

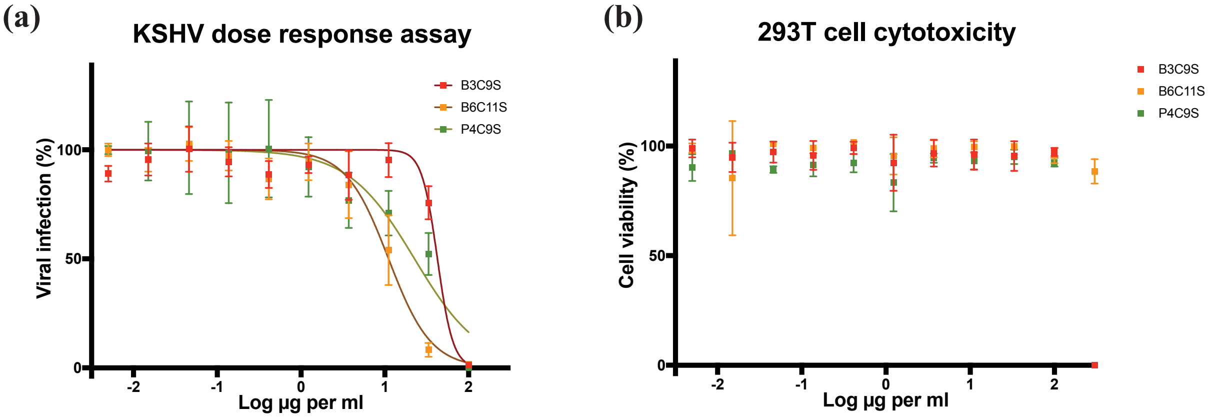

Figure S7. KSHV inhibition (a) and cytotoxicity (b) of multivalent sulfates on 293T cells.

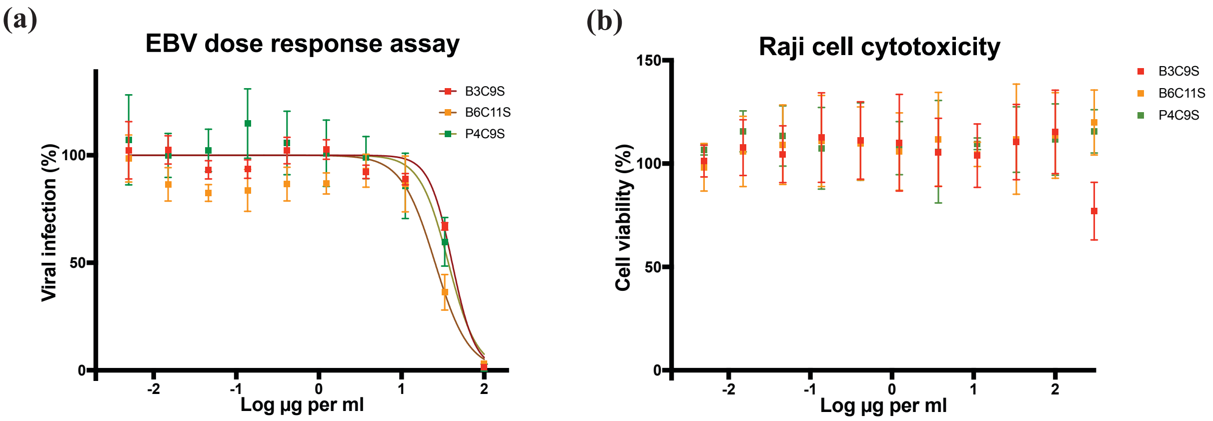

Figure S8. EBV inhibition (a) and cytotoxicity (b) of multivalent sulfates on Raji T cell

Table S1. Broad-spectrum viral inhibition of multivalent sulfates

Compound	Virus	EC_{50} µM (95% CI)	Virucidal	CC_{50} µM (95% CI)	SI
	HSV-2	2.59 (2.16-3.06)	Y	200 (191-208)	77
	SARS-CoV-2 (Alpha)	0.24 (0.001-0.79)	Y	17.5 (7.7-42.4)	73
B6C11S	SARS-CoV-2 (Omicron)	1.14 (0.40-4.70)	NT*	17.5 (7.7-42.4)	15
	HIV-1	0.79 (0.73-0.86)	NT	>54	>68
	EBV	13.7 (9.56-19.2)	NT	>162	>12
	KSHV	5.99 (4.22-8.28)	NT	>162	>27
	H1N1	0.023 (0.019-0.028)	Y	>5.4	>235
B3C11S	HSV-2	26.2 (22.5 - 30.8)	Y	384 (367-399)	15
	SARS-CoV-2 (Alpha)	0.84 (0.69-)	Y	11.2 (8.5-15.9)	13
	HIV-1	6.44 (5.44-7.60)	NT	>100	>15
B3C9S	HSV-2	7.92 (6.80-9.12)	Y	331 (261-)	42
	SARS-CoV-2 (Alpha)	1.10 (-1.47)	Y	14.7 (9.0-27.6)	13
	SARS-CoV-2 (Omicron)	1.37 (0.62-2.64)	NT	14.7 (9.0-27.6)	11
	HIV-1	6.34 (5.81-6.94)	NT	>113	18
	EBV	46.5 (40.3-55.7)	NT	>341	>7
	KSHV	48.2 (-64.4)	NT	162	3
	H1N1	5.11 (-7.48)	NT	16.6 (-21.9)	3
B3C6S	HSV-2	78.4 (70.4-86.6)	N	>530	>7
	SARS-CoV-2 (Alpha)	195 (146-260)	N	>530	>2.7
	HIV-1	5.47 (3.09-8.69)	NT	>133	24

B6C9S	HSV-2	14.0 (12.9-15.1)	Y	42.7 (-50.5)	3
	SARS-CoV-2 (Alpha)	0.44 (0.35-0.55)	Y	3.9 (2.7-5.7)	9
	HIV-1	1.02 (0.95-1.1)	NT	10.5 (-12.9)	10
OB3C9S	HSV-2	18.0 (17.2-18.6)	Y	135 (125-144)	8
	SARS-CoV-2 (Alpha)	1.40 (1.12-1.75)	Y	18.4 (11.8-30.3)	13
BET3C9S	HSV-2	13.1 (12.1-14.0)	Y	81.6 (66.4-108)	6
	SARS-CoV-2 (Alpha)	1.10 (0.91-1.10)	N	17.0 (9.0-36.2)	15
	SARS-CoV-2 (Omicron)	>104	NT	NT	-
	HIV-1	6.12 (4.88-7.61)	NT	29.7	5
BET3C6S	HSV-2	28.5 (24.7-3.1)	Y	>477	>17
	SARS-CoV-2 (Alpha)	>119	NT	451 (393-521)	-
	HIV-1	8.22 (2.78-8.69)	NT	>119	>14
BTA3C10S	HSV-2	17.1 (15.5-18.5)	Y	>371	>22
	SARS-CoV-2 (Alpha)	>93	NT	>929	-
	HIV-1	5.25 (3.64-7.27)	NT	>93	>18
	H1N1	22.9 (11.6-47.6)	NT	>930	>41
P4C9S	HSV-2	6.48 (6.01-6.92)	Y	185 (168-204)	29
	SARS-CoV-2 (Alpha)	0.48 (0.46-0.75)	Y	4.0 (3.2-5.1)	8
	HIV-1	1.94 (1.59-2.31)	NT	18.4 (15.1-22.3)	9
	EBV	33.9 (25.5-43.5)	NT	>276	>8
	KSHV	20.8 (13.1-32.1)	NT	122	6
P3C9S	HSV-2	24.4 (20.3-28.6)	Y	522 (-676)	21
	SARS-CoV-2 (Alpha)	10.3 (5.8-19.0)	N	413 (332-557)	40
	SARS-CoV-2 (Omicron)	>1078	-	413 (332-557)	-
P2C9S	HSV-2	326 (293-359)	Y	NT	-
	SARS-CoV-2 (Alpha)	>1300	NT	>1300	-
A3C9S	HSV-2	20.1 (18.6 – 21.9)	Y	221 (207-236)	11

	SARS-CoV-2 (Alpha)	23.1 (18.1-29.1)	Y	39.5 (30.9-50.0)	2
I3C10S	HSV-2	163 (136-196)	N	>359	>2
	SARS-CoV-2 (Alpha)	458 (297-882)	NT	>356	-
B3PEG4S	HSV-2	>400	NT	NT	-
	SARS-CoV-2 (Alpha)	>400	NT	NT	-

NT* denotes not tested.

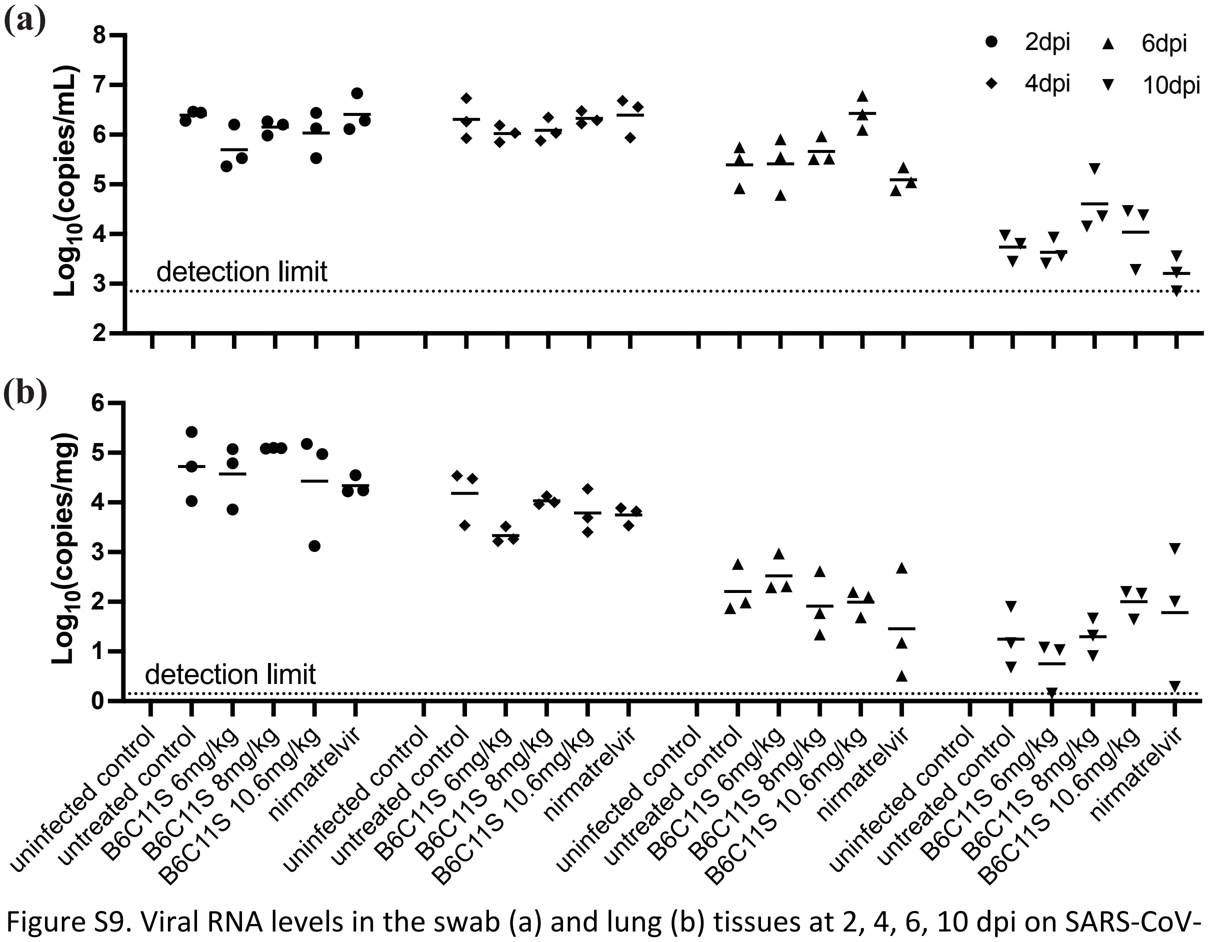

Figure S9. Viral RNA levels in the swab (a) and lung (b) tissues at 2, 4, 6, 10 dpi on SARS-CoV-2 Syrian hamster model (aerosol treatment). No statistical difference was observed within the groups at the same timepoint. Data were analyzed with the One-Way ANOVA test with Prism 9.

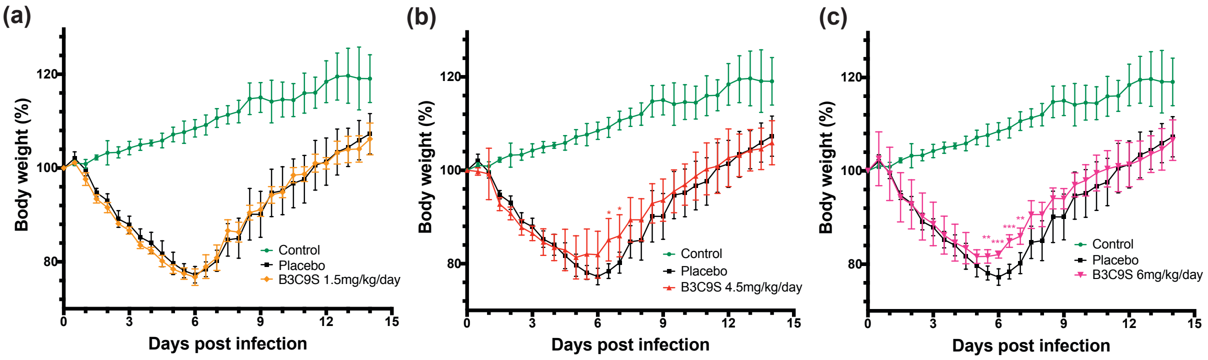

Figure S10. Body weight of Syrian hamsters treated with 1.5mg/kg/day B3C9S (a), 4.5mg/kg/day B3C9S (b), 6mg/kg/day B3C9S (c) by intranasal instillation. 25 female Syrian hamsters were randomly assigned into five groups (control, placebo and three doses of B3C9S), each containing 5 hamsters. At day 0, each hamster was weighed and inoculated intranasally with 100 µl PBS (control) or 100 µl 10^6 TCID50/mL SARS-CoV-2 (placebo and B3C9S). Starting from 12 h after the inoculation, the hamsters in the placebo group and the B3C9S group were each administered with 100 µl PBS or 100 µl B3C9S (1.125, 3.375, 4.5 µg/µl) every day, respectively. The body weight of each hamster was measured every 12 h. The weight measurements of each hamster were normalized by the initial weight and plotted against time post infection. The error bars represent the means ± standard deviations (n = 5). The asterisks represent the p value (*, <0.05; **, <0.01; ***, <0.001) calculated by two-tailed unpaired t test.

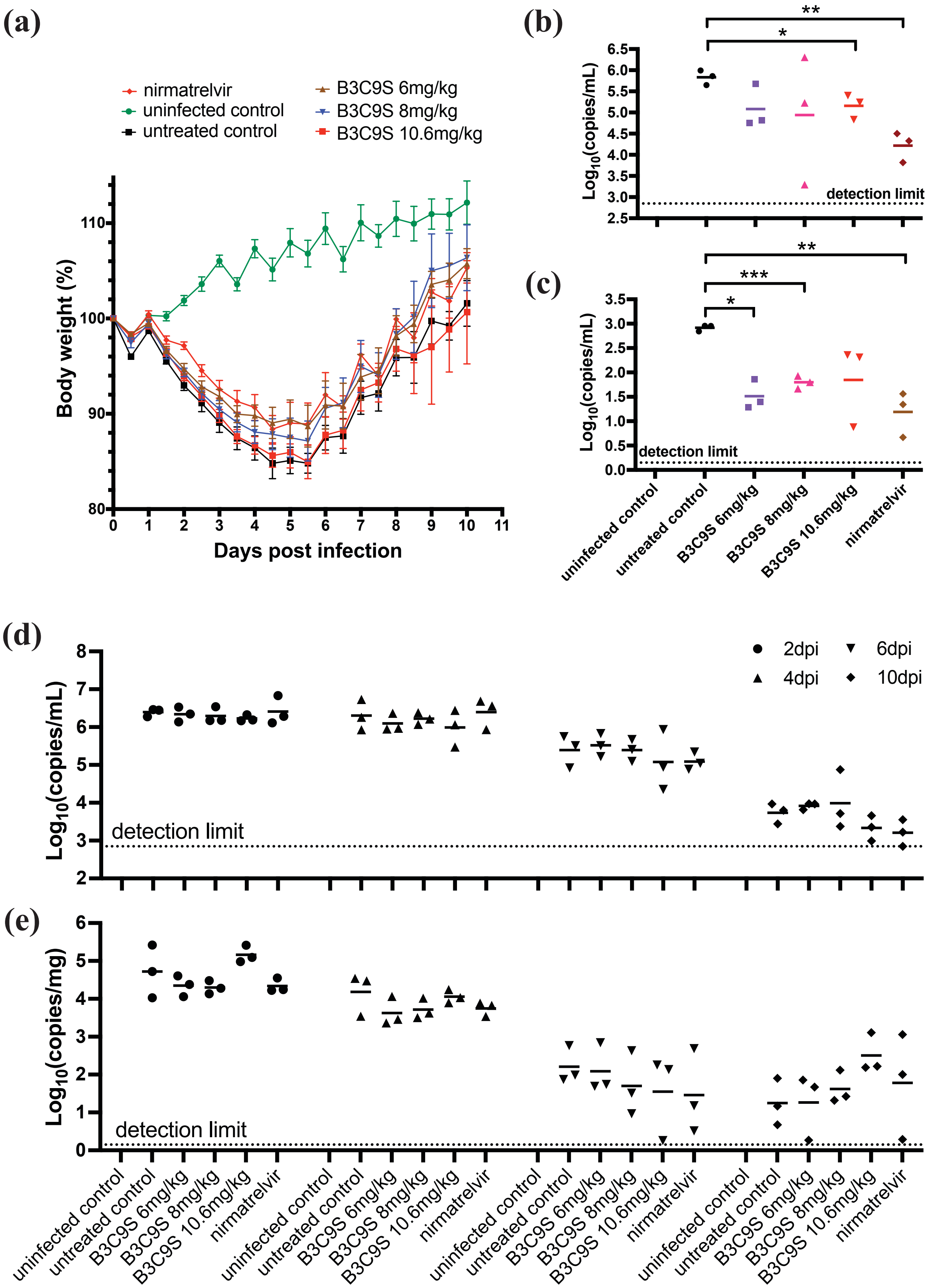

Figure S11. Efficacy of B3C9S on SARS-CoV-2 Syrian hamster model (aerosol treatment). (a) body weight loss percentage of 6, 8, 10.6 mg/kg B3C9S treated, nirmatrelvir treated, untreated and uninfected hamsters. Viral RNA levels in the swab (b) and lung (c) at 8dpi. The

viral titer in the oral swab was significantly reduced by B3C9S at a dose of 10.6 mg/kg ($P < 0.05$), and in the lungs, B3C9S at doses of 6 mg/kg ($P < 0.05$) and 8 mg/kg ($P < 0.001$) significantly decreased the viral titer. No statistical difference of viral titer was observed among groups treated with B3C9S, nirmatrelvir and the untreated control in the oral swab (d) and lung (e) tissues at 2, 4, 6, 10 dpi. Data were analyzed with two-tailed unpaired t test. The asterisks represent the p value (*, <0.05; **, <0.01; ***, <0.001).

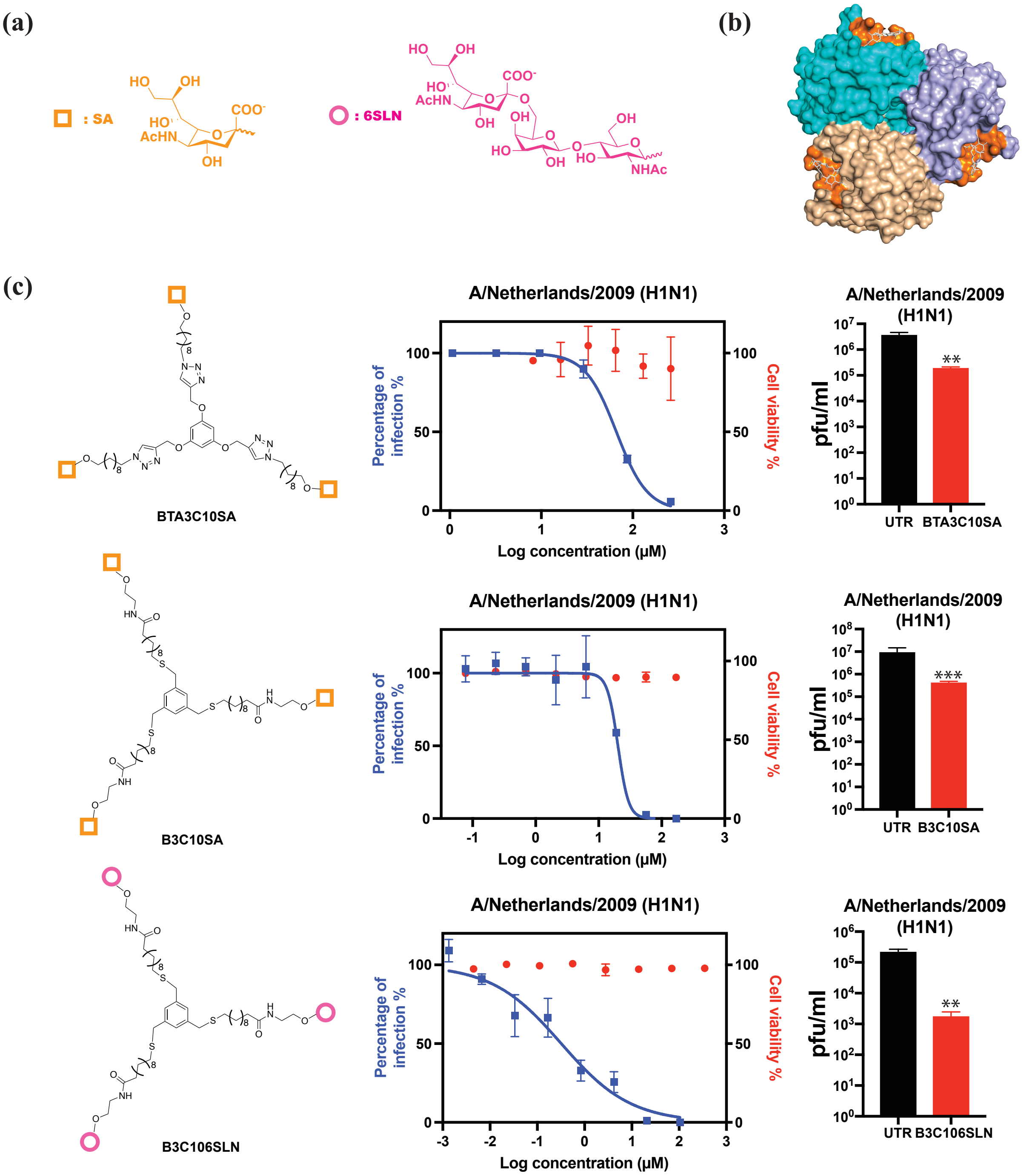

Figure S12. Benzene-based scaffold modified with sialic acid ligands also shows virucidal activity against influenza. (a) Chemical structure of SA and 6SLN. (b) Top view of a Hemagglutinin trimer in complex with ligand 6SLN (H1, influenza A/California/04/2009, PDB 3UBN[1] in a surface representation. The amino acid residues interacting with 6SLN are colored orange and the 6SLN residues are shown in a stick representation colored white. (c) Benzene-sialic acid's inhibition curves against influenza A/ Netherlands/2009 (H1N1), MDCK cell viability curves over concentrations and virucidal results. Results are plotted as averages and standard errors. Statistical significance was analyzed with two-tailed unpaired t-test. The asterisks represent the p value (**, <0.01; ***, <0.001).

Materials and reagents for synthesis

All chemicals were purchased as reagent grade and used without further purification unless otherwise noted. DMF and DMSO (Extra dry) were purchased from Sigma. Analytical thin layer chromatography (TLC) was performed on silica gel 60 F254 aluminum supported plate (layer thickness 0.2mm). Visualization of the spots was achieved by exposure to UV light (254nm) and/or charring with a solution of 5% (v/v) sulfuric acid in EtOH or ceric ammonium molybdate, followed by gentle heating. Column chromatography on Silica gel or C18 reverse phase column was carried out with Biotage Selekt Systems. High resolution mass spectra (HRMS) were obtained by electrospray ionization (ESI). For ^{1}H nuclear magnetic resonance (NMR) spectra, chemical shifts were reported in parts per million (ppm) calibrated with tetramethylsilane (δ = 0.00 ppm) in $CDCl_3$. ^{13}C NMR spectra were calibrated with tetramethylsilane (δ = 0.0 ppm) in $CDCl_3$. Coupling constants (J) were given in Hertz (Hz).

1. Synthesis of multivalent sulfated antivirals

General procedure A: alkyl thioethers synthesis

Dibromides, tribromides, tetrabromides or hexabromides (1 g) and cesium carbonate (1.1eq per bromide for hexabromides, 1.05 eq per bromide for others) were added into 10 mL of dry DMF under argon atmosphere. The mixture was stirred at 60 °C, and mercapto alkanol (1.1eq per bromide for hexabromides, 1.05 eq per bromide for others, dissolved in 6 mL DMF) was added dropwise to the mixture. After stirring at 60 °C for 2 days, cesium carbonate was filtered out and DMF was evaporated. The crude product was purified by flash column chromatography (gradient elute: pure DCM - DCM/MeOH=50/1 - DCM/MeOH=15/1) to yield a white powder. Recrystallization from ethanol could be processed to obtain higher purity of product.

General procedure B: O-sulfation

100 mg diol, triol, tetraol or hexaol and sulfur trioxide pyridine complex (10 eq per hydroxyl) were dissolved in 0.8mL of dry DMF and stirred under Argon at 60°C for two days. Sephadex LH-20 (MeOH) was used to remove excess sulfur trioxide pyridine. Crude product was then dissolved in 5mL 1M NaOH and stirred overnight, followed by addition of Dowex H+ resin and adjusting pH to 8.5. After filtering out the resin and removing the solvent, Biotage C18 reverse-phase column with $MeOH/H_2O$ 10%-100% elution was used to purify the product.

B3C11OH

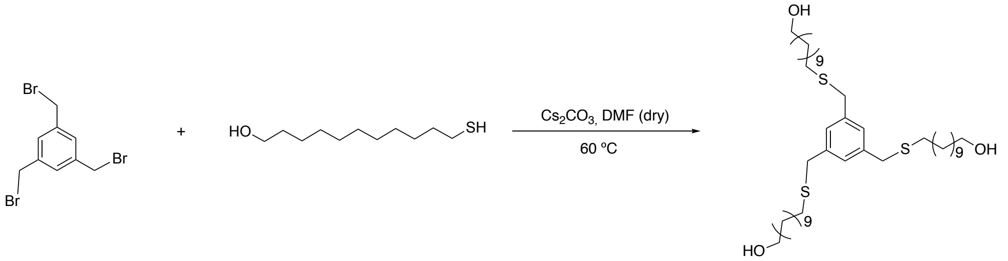

1,3,5-tris(bromomethyl)-benzene and 11-mercapto-undecanol were used to synthesize compound B3C11OH following the general method A. The product was a white powder, yield 58.4%. ^{1}H NMR (400 MHz, $CDCl_3$) δ 7.11 (s, 3H, Ar-H), 3.65 (s, 6H, Ar-CH_2), 3.61 (t, J = 6.6 Hz, 6H, CH_2-O), 2.37 (t, J = 7.4 Hz, 6H), 1.74 (s, 4H), 1.62 – 1.15 (m, 60H). ^{13}C NMR (101 MHz, $CDCl_3$) δ 139.04, 127.93, 62.98, 36.08, 32.78, 31.43, 29.60, 29.54, 29.53, 29.44, 29.27, 29.25, 28.93, 25.76. HRMS (nanochip-ESI/LTQ-Orbitrap) m/z: [M + Na]$^+$ Calcd for $C_{42}H_{78}NaO_3S_3^+$ 749.5005; Found 749.5024.

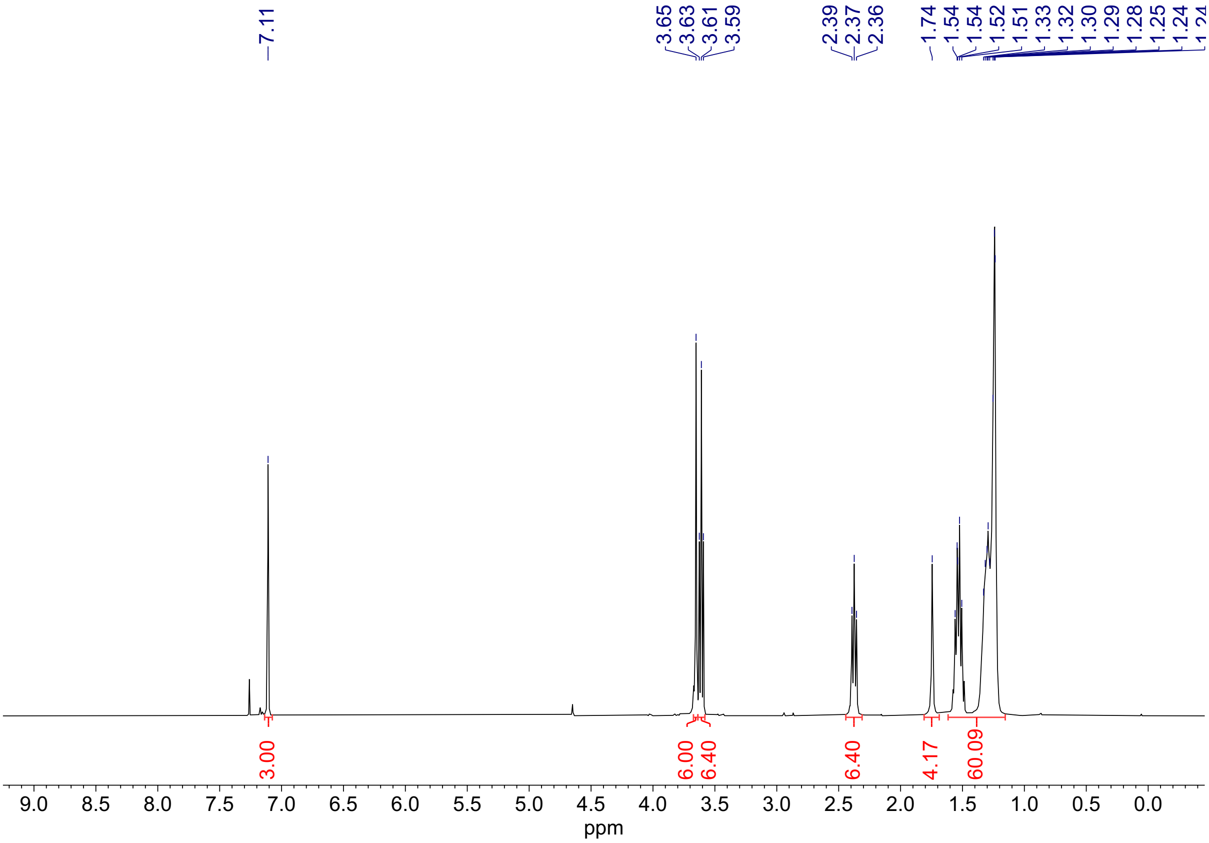

Figure S13. ^{1}H-NMR ($CDCl_3$, 400 MHz) of B3C11OH

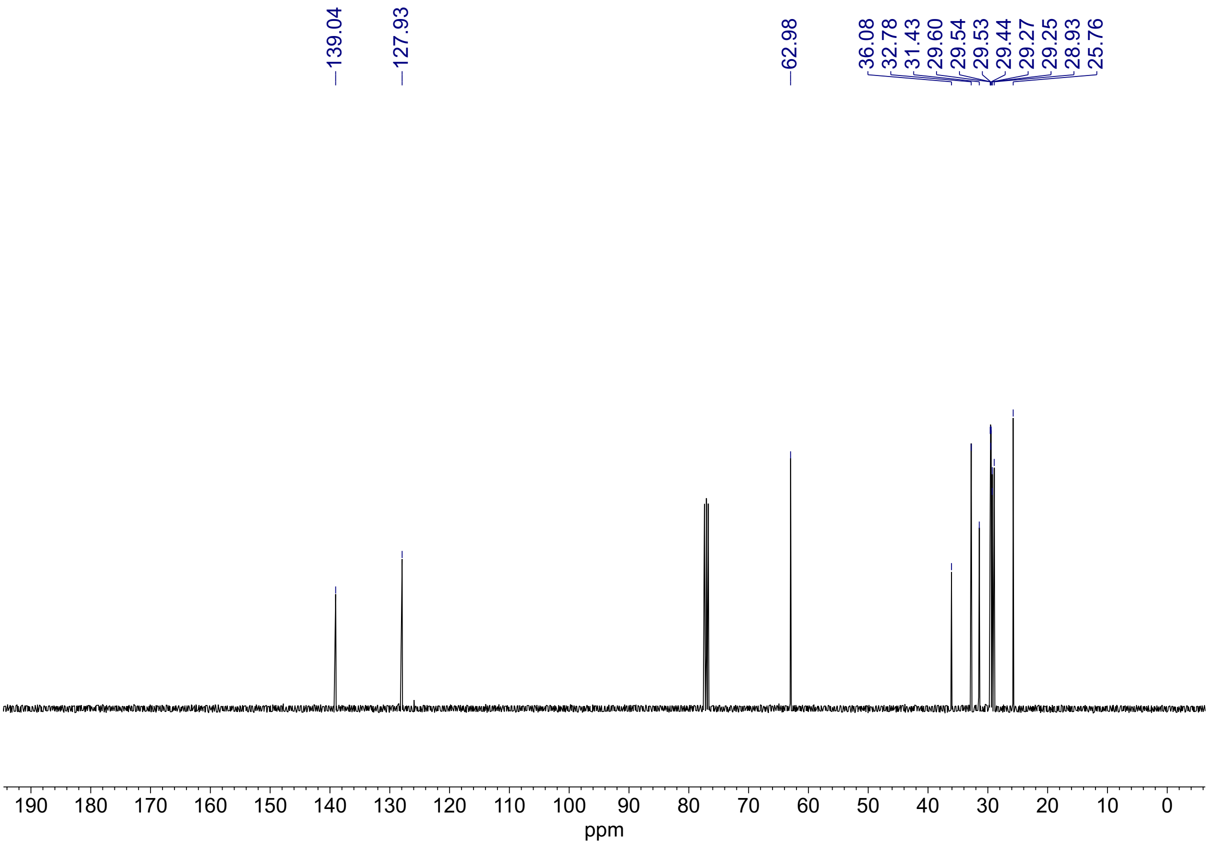

Figure S14. ^{13}C-NMR ($CDCl_3$, 101 MHz) of B3C11OH

B3C11S

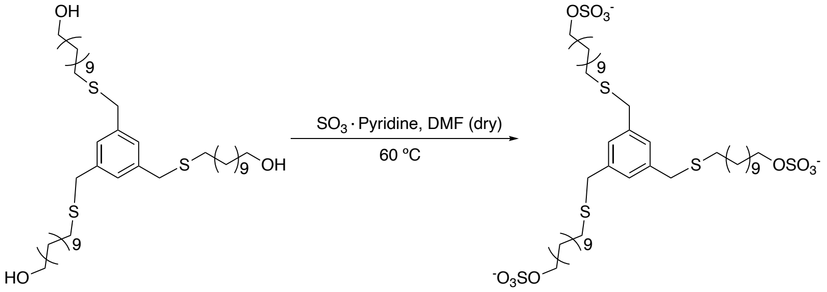

From compound B3C11OH following the general method B gave compound B3C11S as a white powder, yield 61.8%. ^{1}H NMR (400 MHz, D_2O) δ 7.13 (s, 3H, Ar-H), 4.03 (t, *J* = 6.7 Hz, 6H, CH_2-SO_4-), 3.64 (s, 6H, Ar-CH_2), 2.37 (d, *J* = 7.6 Hz, 6H, S-CH_2), 1.80 – 1.09 (m, 54H, CH_2-$\underline{CH_2}$-CH_2). ^{13}C NMR (101 MHz, D_2O) δ 139.50, 128.33, 69.68, 36.51, 31.85, 30.12, 29.97, 29.95, 29.83, 29.70, 29.48, 25.92. HRMS (nanochip-ESI/LTQ-Orbitrap) m/z: $[M]^{3-}$ Calcd for $C_{42}H_{75}O_{12}S_6^{3-}$ 321.1200; Found 321.1165.

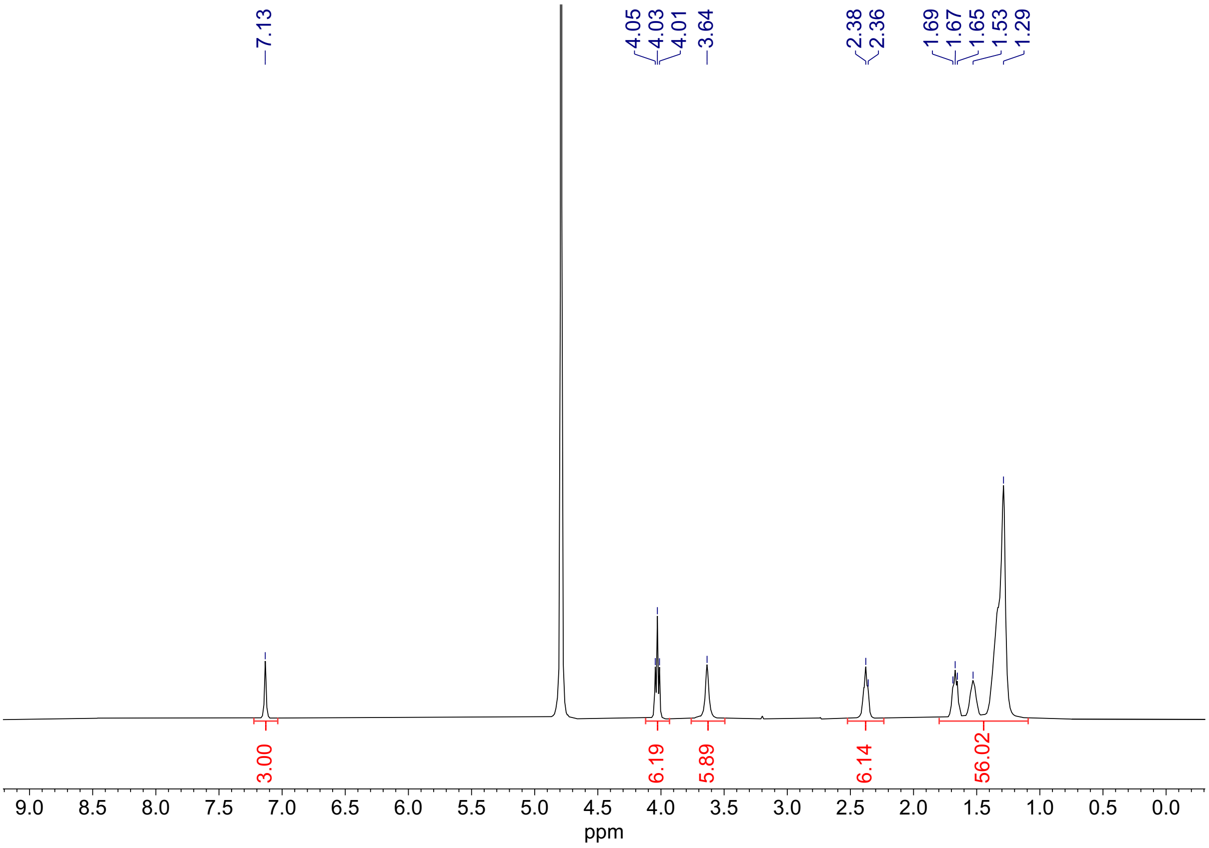

Figure S15. ^{1}H-NMR (D_2O, 400 MHz) of B3C11S

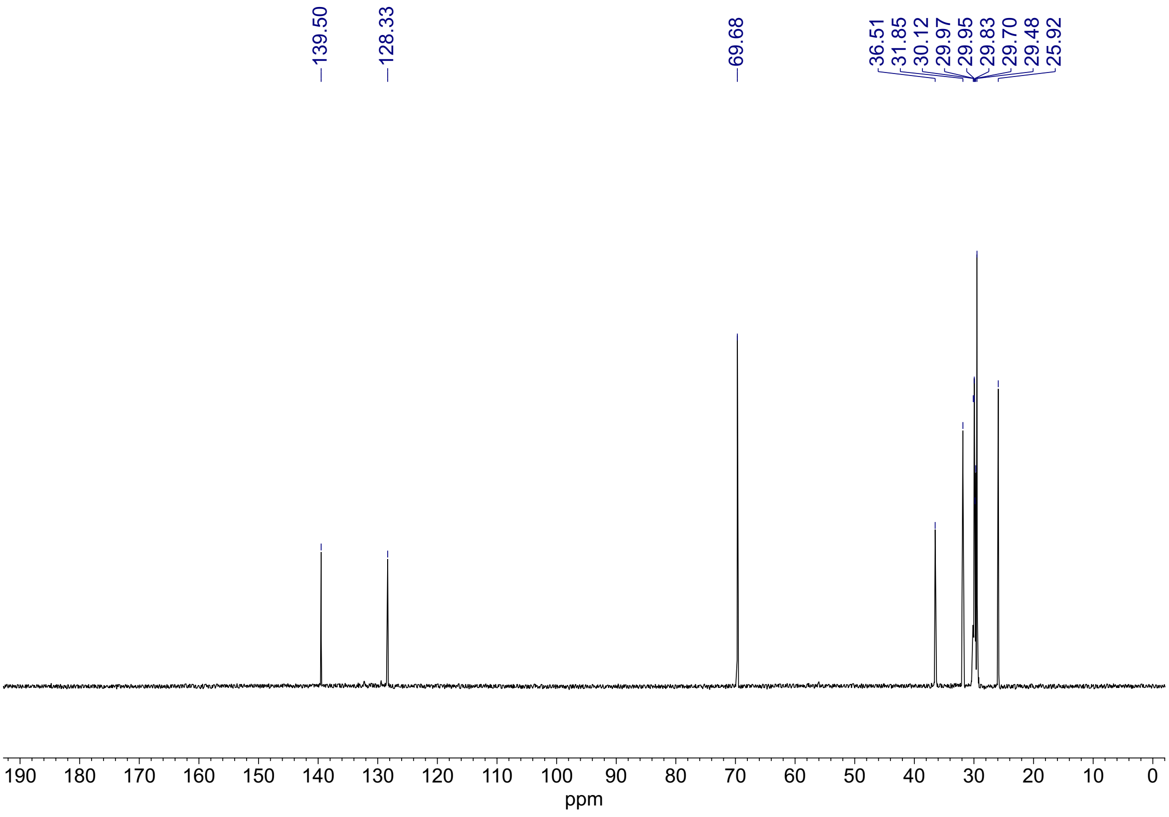

Figure S16. ^{13}C-NMR (D_2O, 101 MHz) of B3C11S

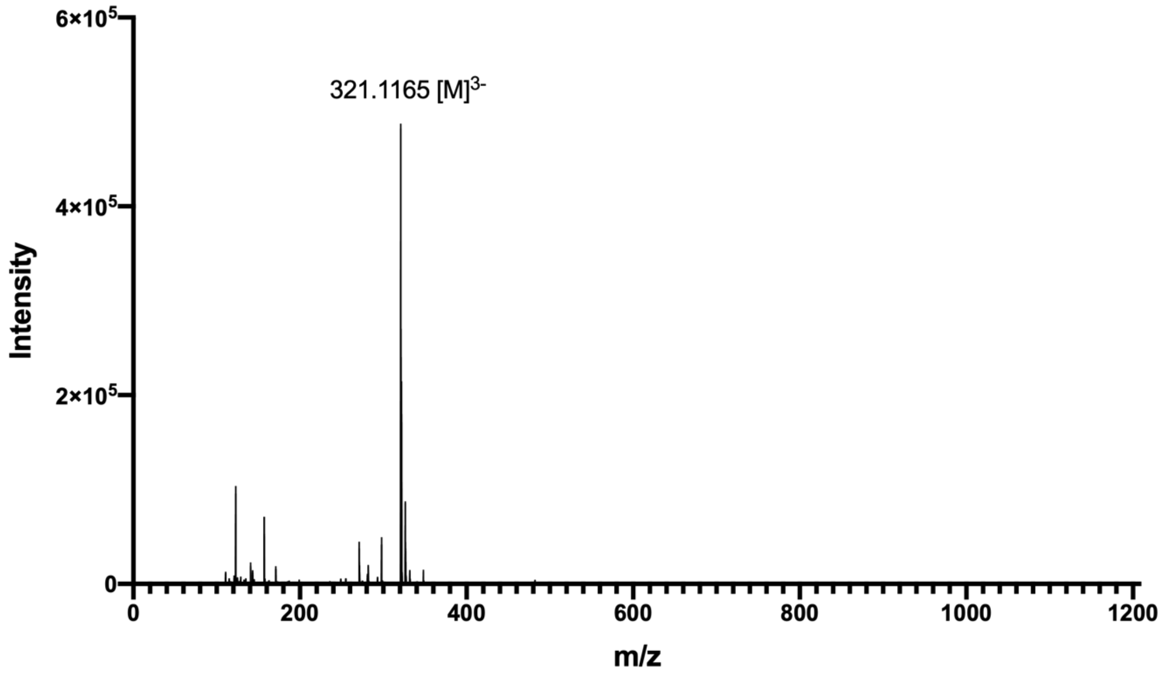

Figure S17. MS (nanochip-ESI/LTQ-orbitrap) of B3C11S

B3C9OH

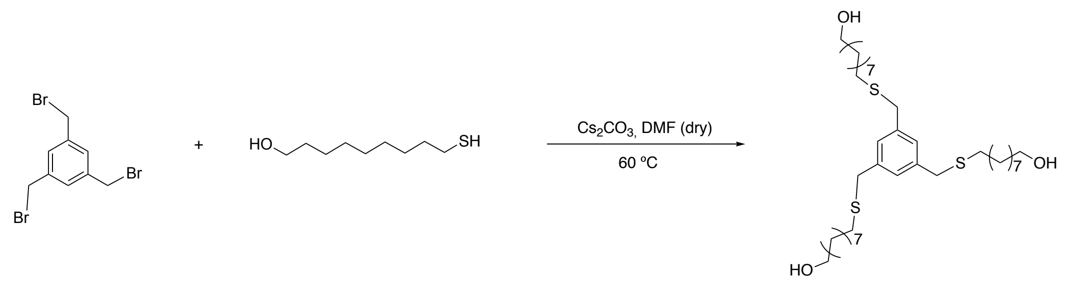

1,3,5-tris(bromomethyl)-benzene and 9-mercapto-1-nonanol were used to synthesize compound B3C9OH following the general method A. The product was a white powder, yield 54.6%. ^{1}H NMR (400 MHz, $CDCl_3$) δ 7.15 (s, 3H, Ar-H), 3.67 (m, 12H, Ar-CH_2, CH_2-O), 2.47 – 2.37 (m, 6H, S-CH_2-C), 1.77 – 1.22 (m, 45H, C-CH_2-C, OH).^{13}C NMR (101 MHz, $CDCl_3$) δ 139.06, 127.95, 63.04, 36.10, 32.75, 31.45, 29.50, 29.38, 29.26, 29.19, 28.91, 25.75. HRMS (nanochip-ESI/LTQ-Orbitrap) m/z: $[M + Na]^+$ Calcd for $C_{36}H_{66}NaO_3S_3^+$ 665.4066; Found 665.4083.

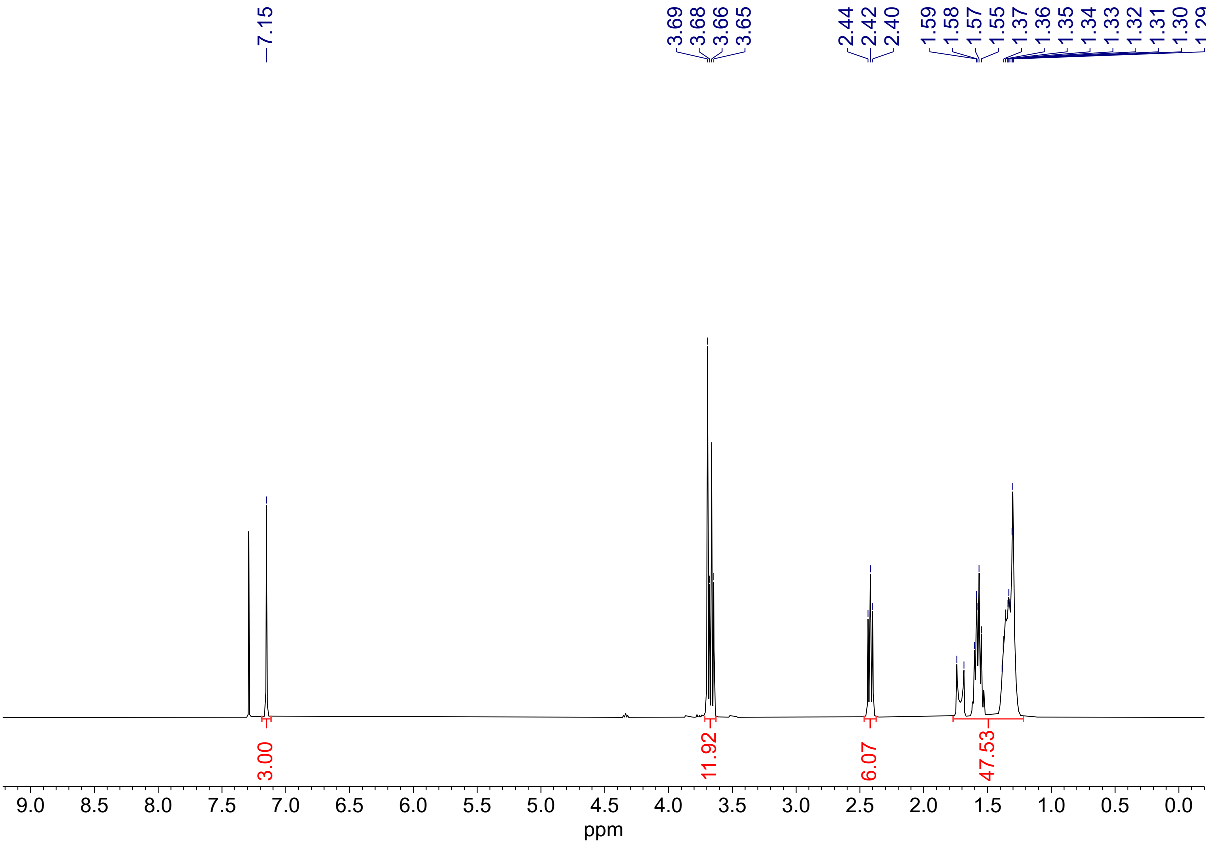

Figure S18. ^{1}H-NMR ($CDCl_3$, 400 MHz) of B3C9OH

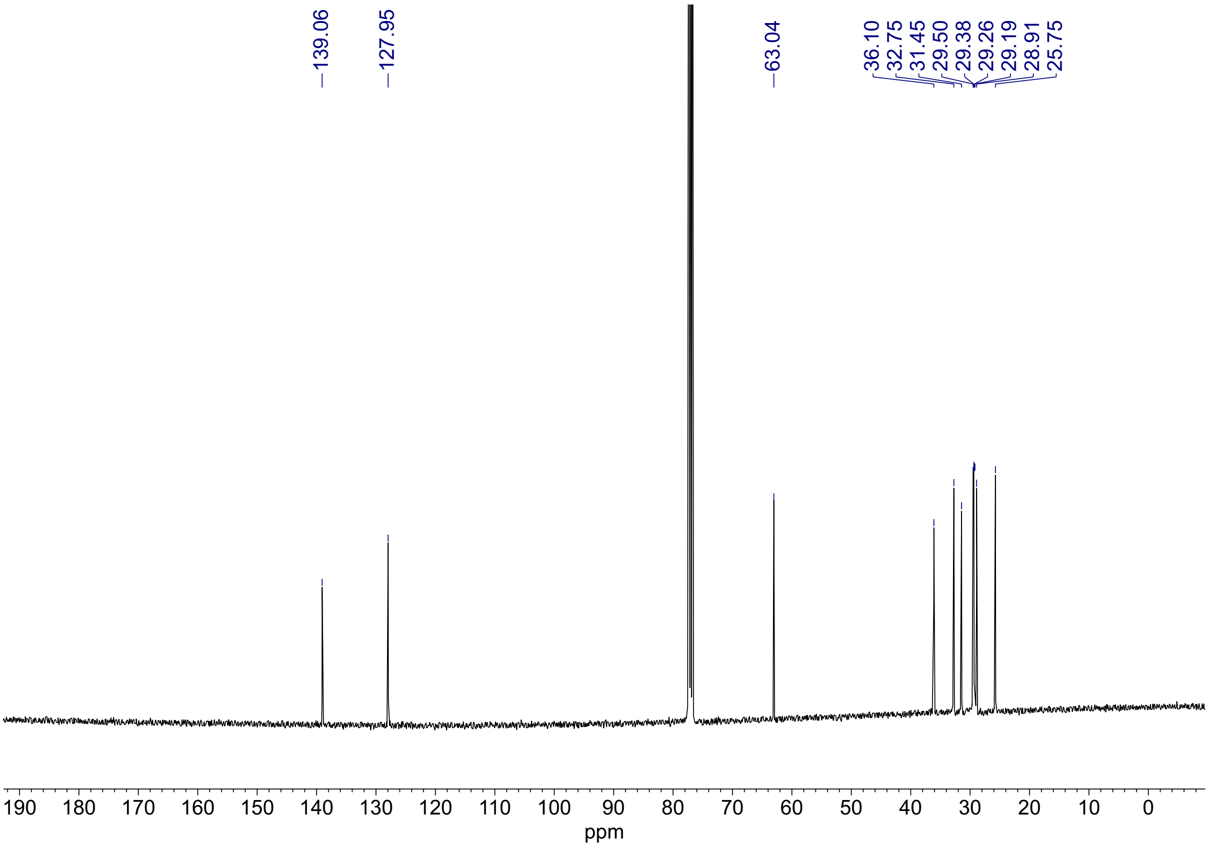

Figure S19. ^{13}C-NMR ($CDCl_3$, 101 MHz) of B3C9OH

B3C9S

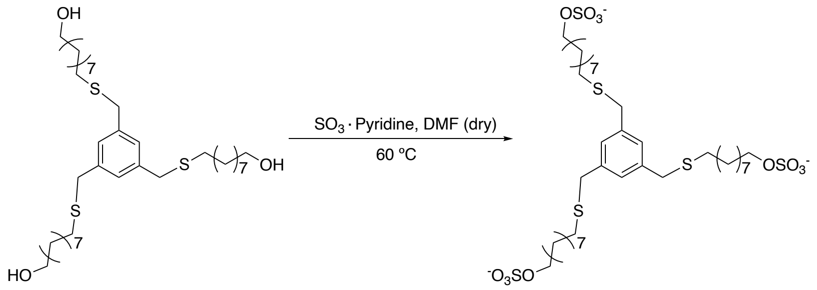

From compound B3C9OH following the general method B gave compound B3C9S as a white powder, yield 65.4%. ^{1}H NMR (400 MHz, D_2O) δ 7.15 (s, 3H, Ar-H), 4.03 (t, J = 6.7 Hz, 6H, CH_2-O), 3.65 (s, 6H, Ar-CH_2), 2.39 (t, J = 7.4 Hz, 6H, S-CH_2-C), 1.74 – 1.06 (m, 42H, C-CH_2-C). ^{13}C NMR (101 MHz, D_2O) δ 139.03, 128.06, 69.32, 35.74, 31.15, 29.47, 29.37, 29.31, 29.23, 29.02, 28.95, 25.46. HRMS (nanochip-ESI/LTQ-Orbitrap) m/z: $[M]^{3-}$ Calcd for $C_{36}H_{63}O_{12}S_6^{3-}$ 293.0881; Found 293.0873.

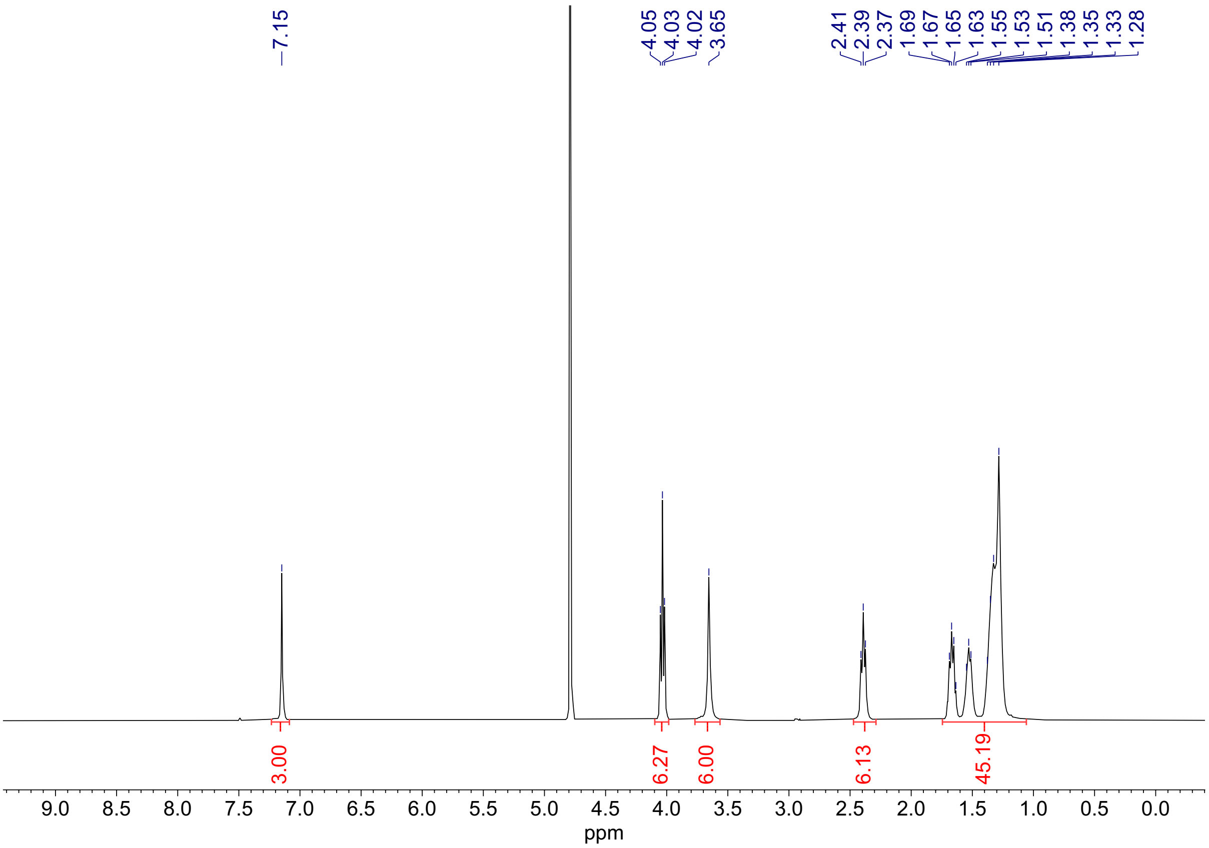

Figure S20. ^{1}H-NMR (D_2O, 400 MHz) of B3C9S

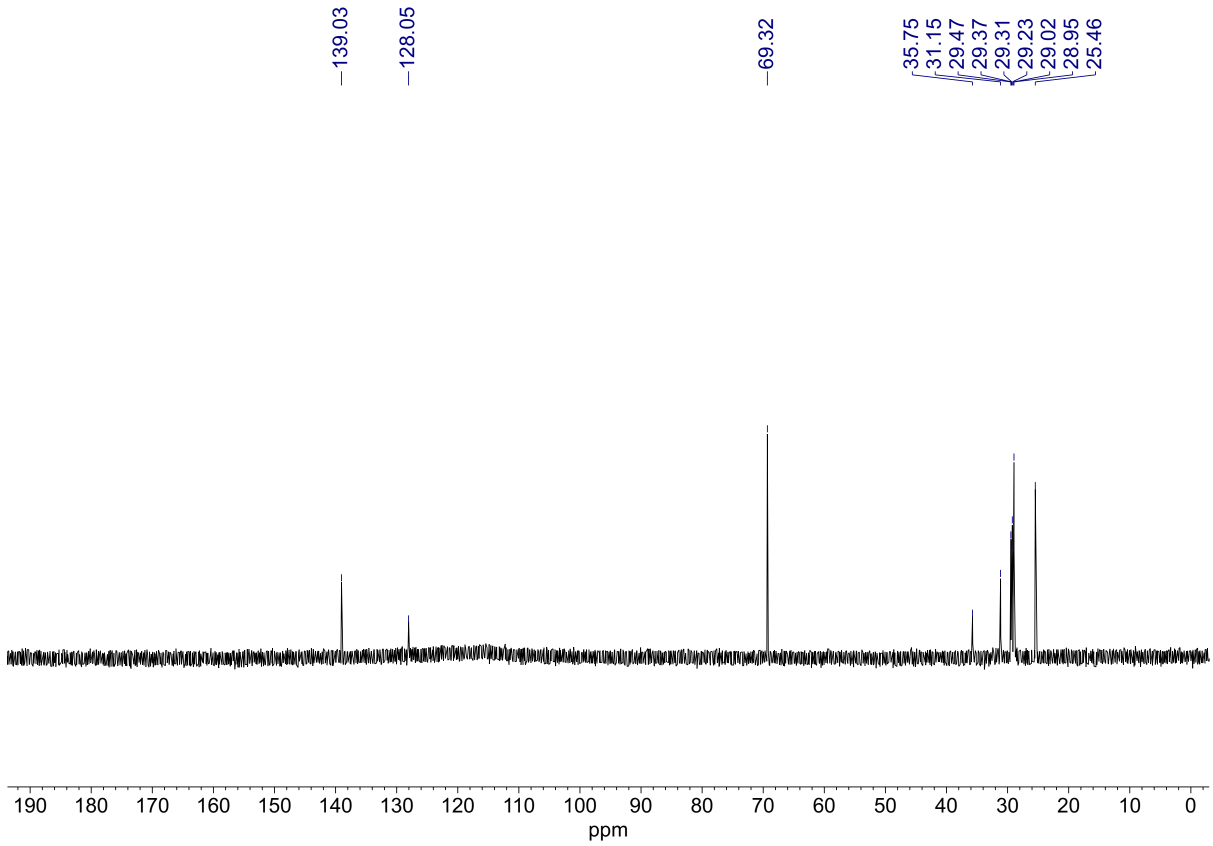

Figure S21. ^{13}C-NMR (D_2O, 101 MHz) of B3C9S

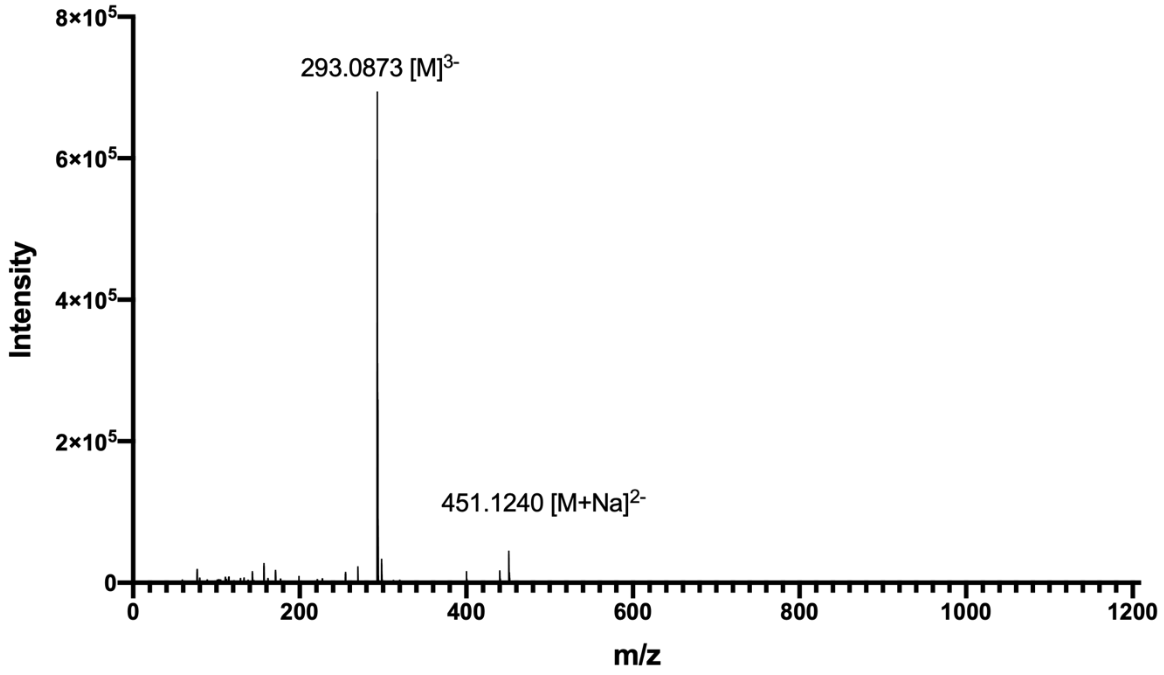

Figure S22. MS (nanochip-ESI/LTQ-orbitrap) of B3C9S

B3C6OH

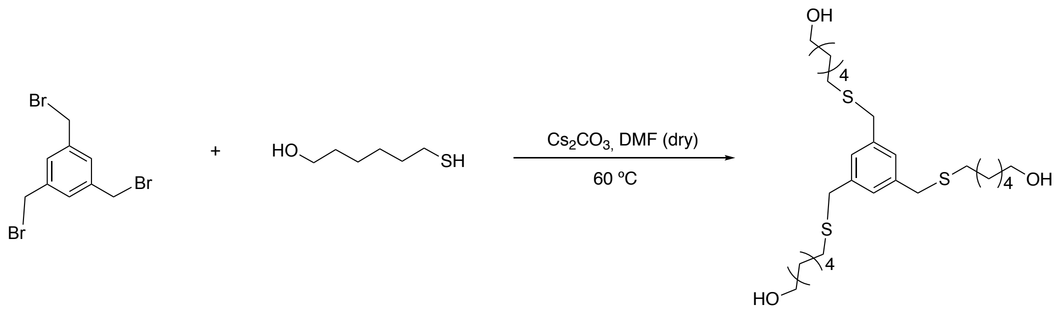

1,3,5-tris(bromomethyl)-benzene and 6-mercapto-1-hexanol were used to synthesize compound B3C6OH following the general method A. The product was a white powder, yield 50.1%. ^{1}H NMR (400 MHz, $CDCl_3$) δ 7.08 (s, 3H, Ar-H), 3.62 (s, 6H, Ar-CH_2), 3.53 (t, J = 6.6 Hz, 6H, CH_2-O), 2.66 (s, 3H, OH), 2.35 (t, J = 7.4 Hz, 6H, S-CH_2-C), 1.49 (dt, J = 11.2, 6.8 Hz, 12H, C-CH_2-C), 1.30 (dp, J = 12.5, 7.0, 6.5 Hz, 12H, C-CH_2-C). ^{13}C NMR (101 MHz, $CDCl_3$) δ 139.00, 127.94, 62.51, 36.05, 32.50, 31.30, 29.16, 28.63, 25.35. HRMS (nanochip-ESI/LTQ-Orbitrap) m/z: $[M + Na]^+$ Calcd for $C_{27}H_{48}NaO_3S_3^+$ 539.2658; Found 539.2665.

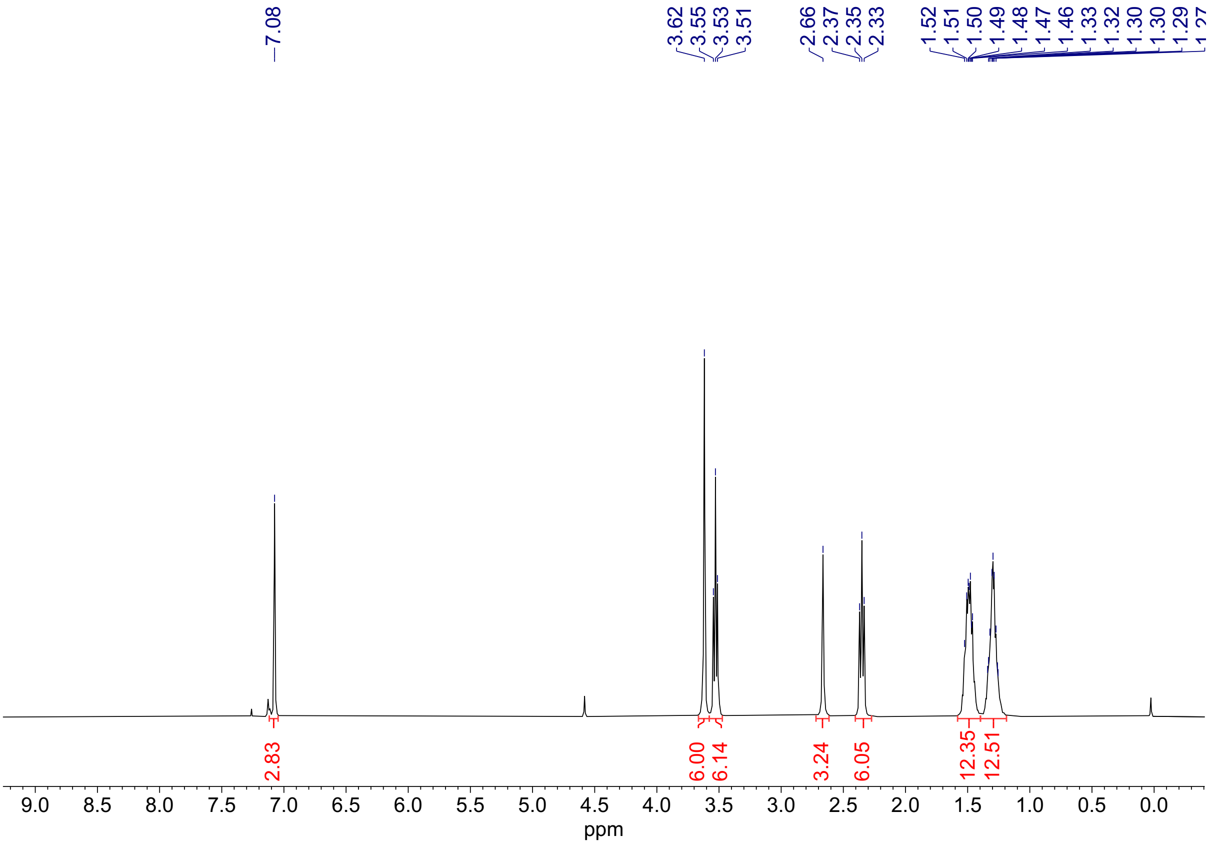

Figure S23. ^{1}H-NMR ($CDCl_3$, 400 MHz) of B3C6OH

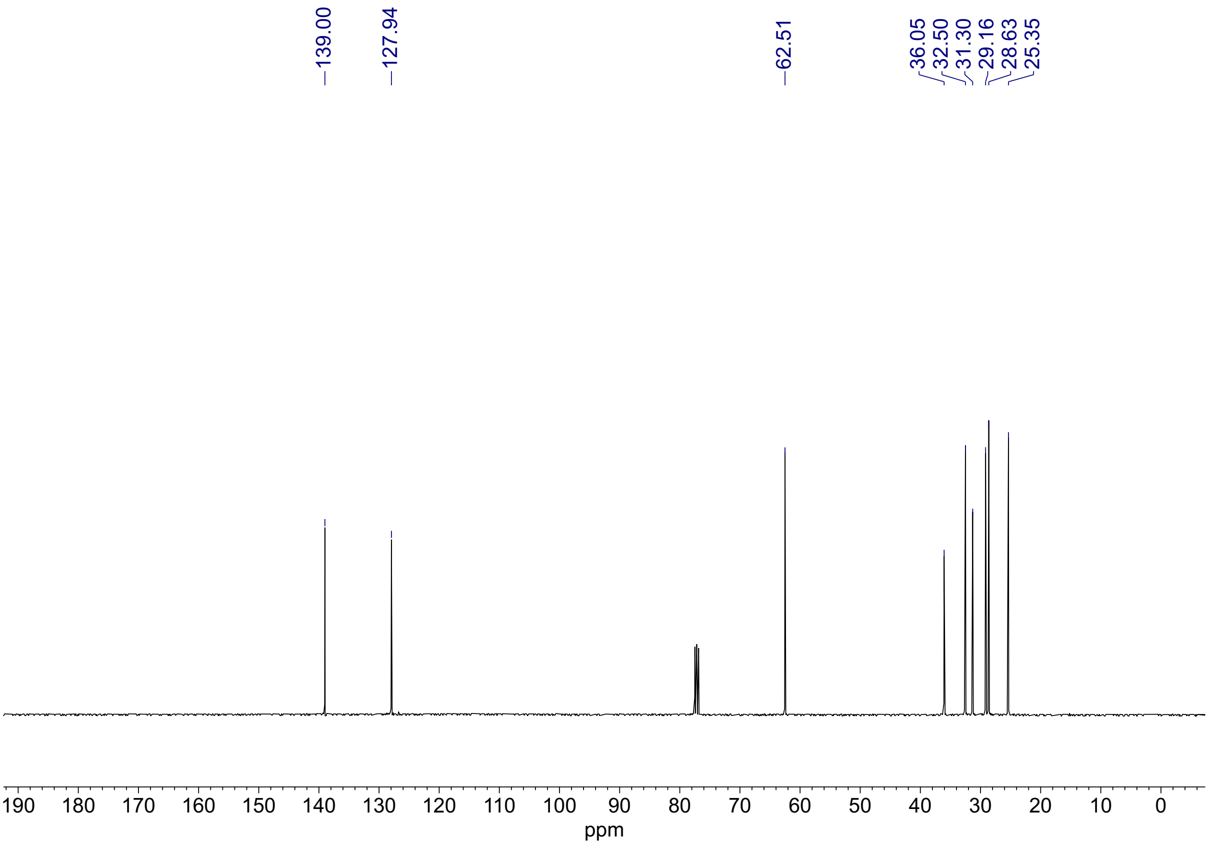

Figure S24. ^{13}C-NMR ($CDCl_3$, 101 MHz) of B3C6OH

B3C6S

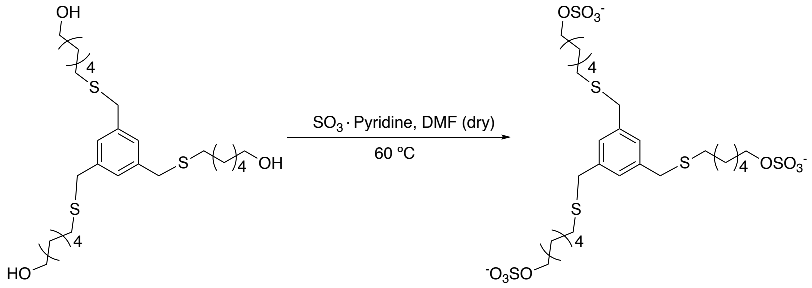

From compound B3C6OH following the general method B gave compound B3C6S as a white powder, yield 67.5%.^{1}H NMR (400 MHz, D_2O) δ 7.19 (s, 3H, Ar-H), 4.07 (t, J = 6.6 Hz, 6H, CH_2-O), 3.71 (s, 6H, Ar-CH_2), 2.45 (t, J = 7.3 Hz, 6H, S-CH_2-C), 1.69-1.38 (m, 24H, C-CH_2-C). ^{13}C NMR (101 MHz, D_2O) δ 139.19, 128.13, 69.35, 35.38, 30.91, 28.80, 28.64, 28.11, 24.82. HRMS (nanochip-ESI/LTQ-Orbitrap) m/z: $[M]^{2-}$ Calcd for $C_{27}H_{45}NaO_{12}S_6^{2-}$ 388.0572; Found 388.0555.

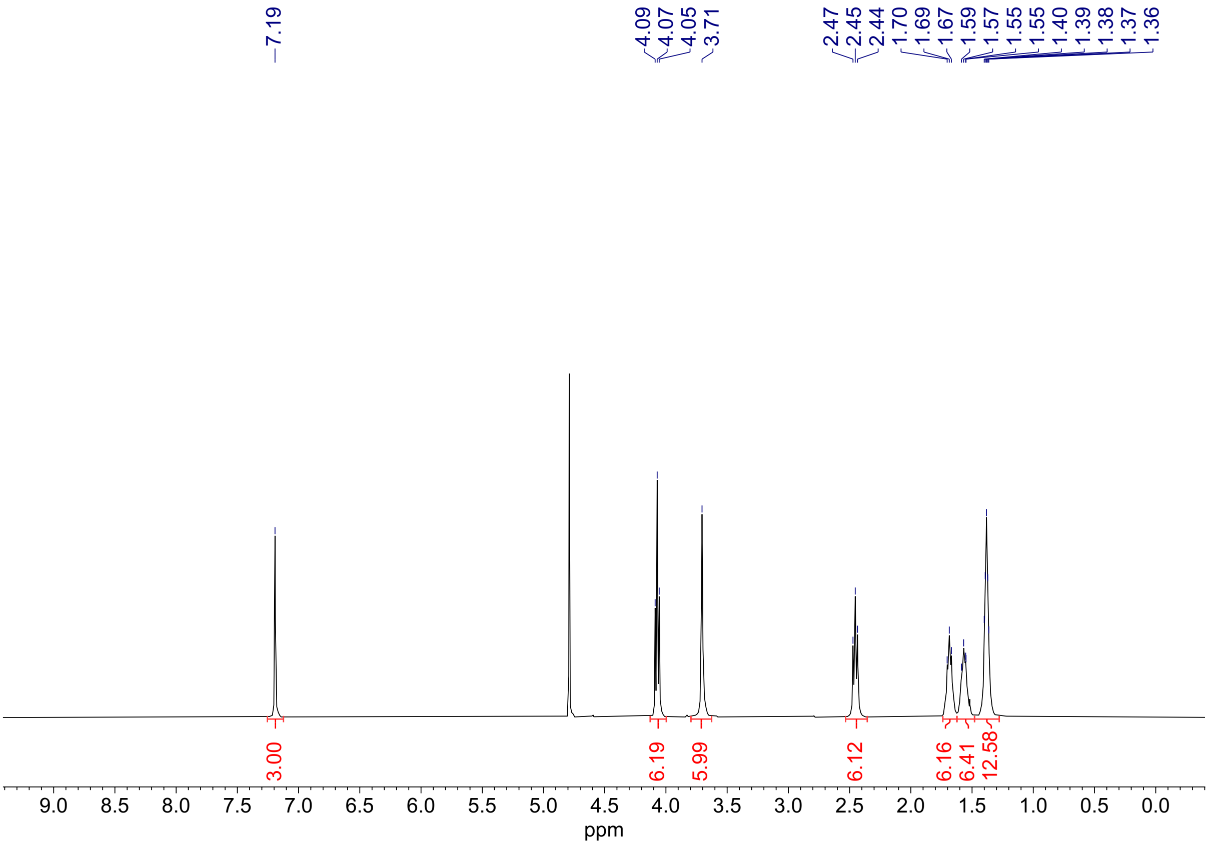

Figure S25. ^{1}H-NMR (D_2O, 400 MHz) of B3C6S

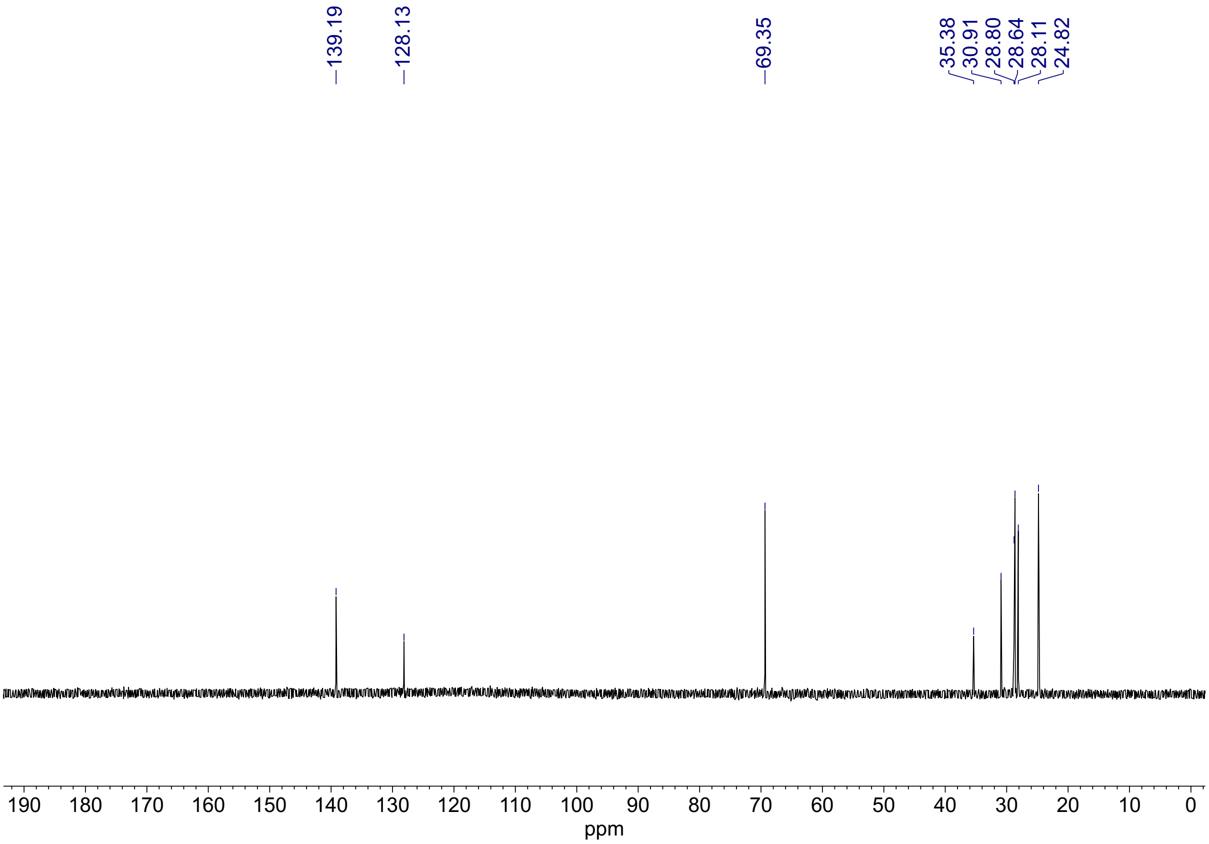

Figure S26. ^{13}C-NMR (D_2O, 101 MHz) of B3C6S

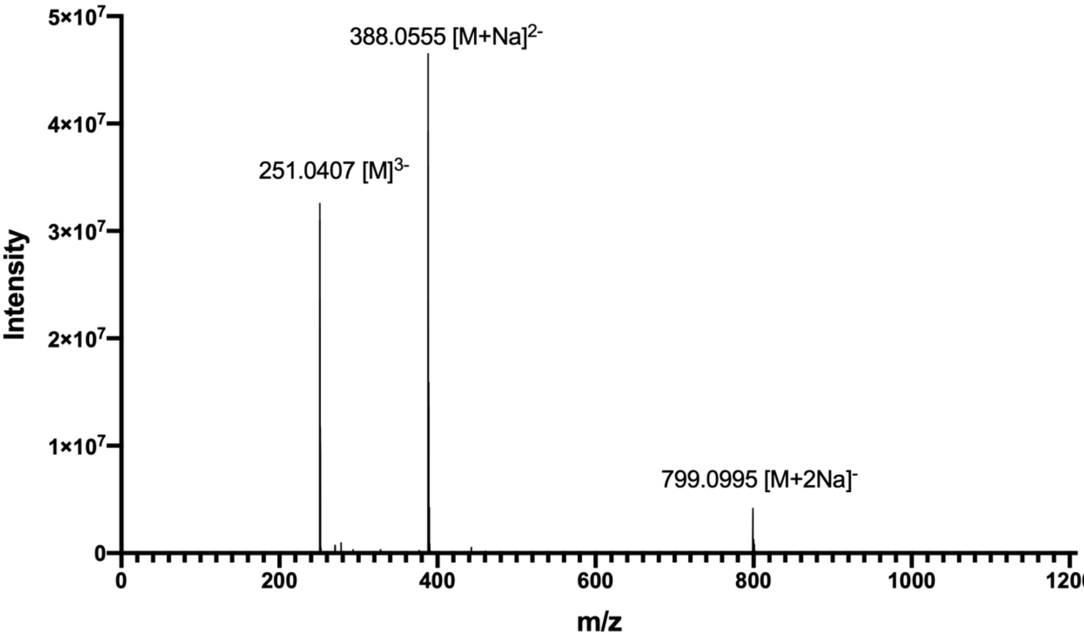

Figure S27. MS (nanochip-ESI/LTQ-orbitrap) of B3C6S

B3PEG4OH

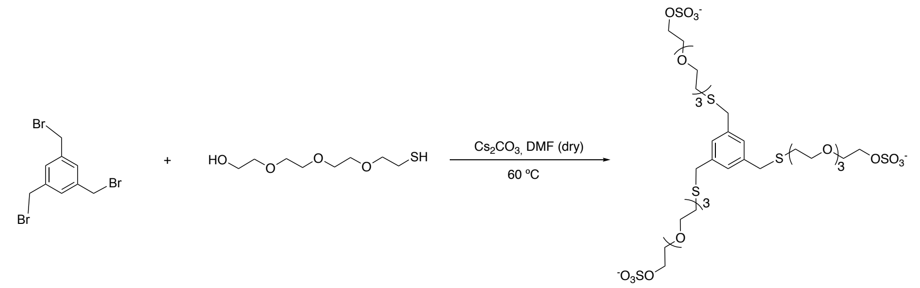

1,3,5-tris(bromomethyl)-benzene and 2-[2-[2-(2-mercaptoethoxy)ethoxy]ethoxy]ethanol were used to synthesize compound B3PEG4OH following the general method A. The product was a white powder, yield 70.2%. ^{1}H NMR (400 MHz, $CDCl_3$) δ 7.05 (s, 3H, Ar-H), 3.66 – 3.42 (m, 48H, Ar-CH_2, CH_2-O), 3.11 (s, 3H, OH), 2.51 (t, J = 6.8 Hz, 6H, S-CH_2-C). ^{13}C NMR (101 MHz, $CDCl_3$) δ 138.90, 128.17, 72.55, 70.69, 70.54, 70.44, 70.23, 70.16, 61.50, 36.31, 30.68. HRMS (nanochip-ESI/LTQ-Orbitrap) m/z: [M + Na]$^+$ Calcd for $C_{33}H_{60}NaO_{12}S_3^+$ 767.3139; Found 767.3155.

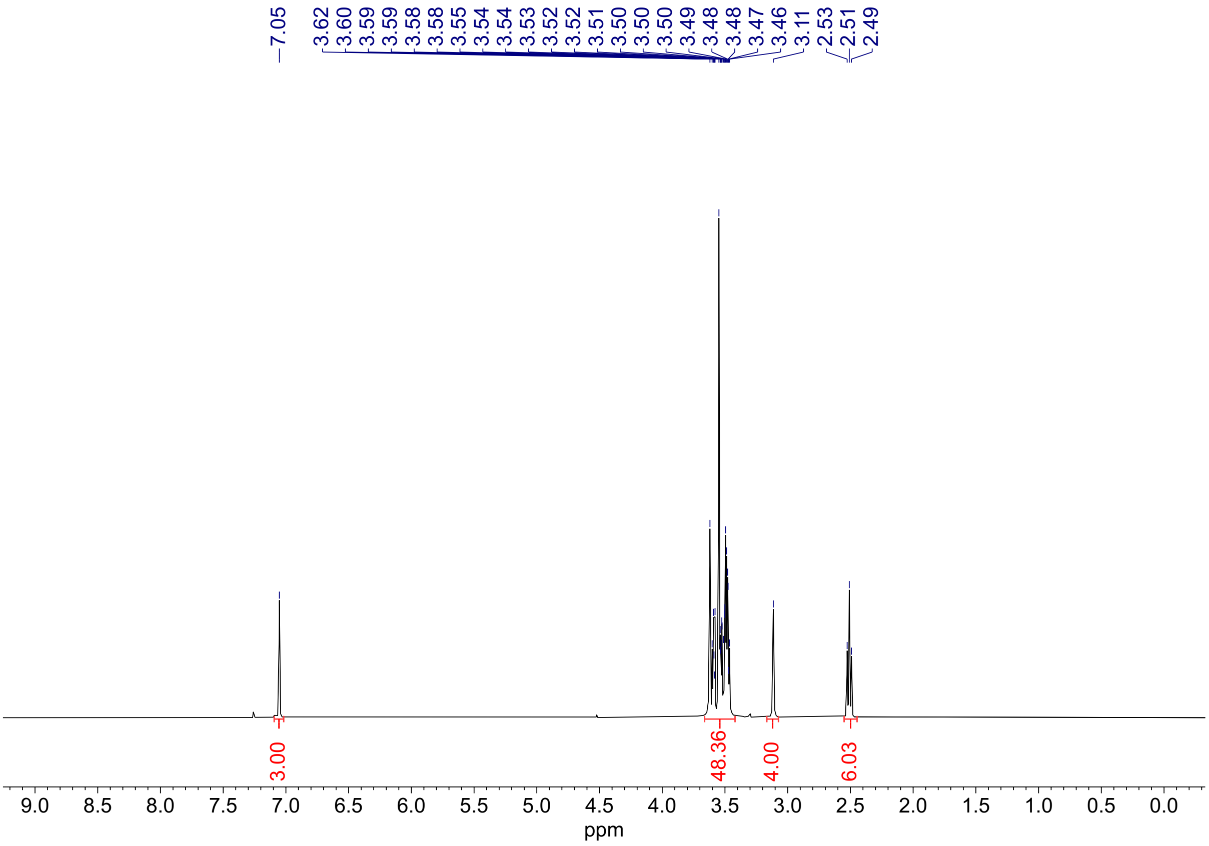

Figure S28. ^{1}H-NMR ($CDCl_3$, 400 MHz) of B3PEG4OH

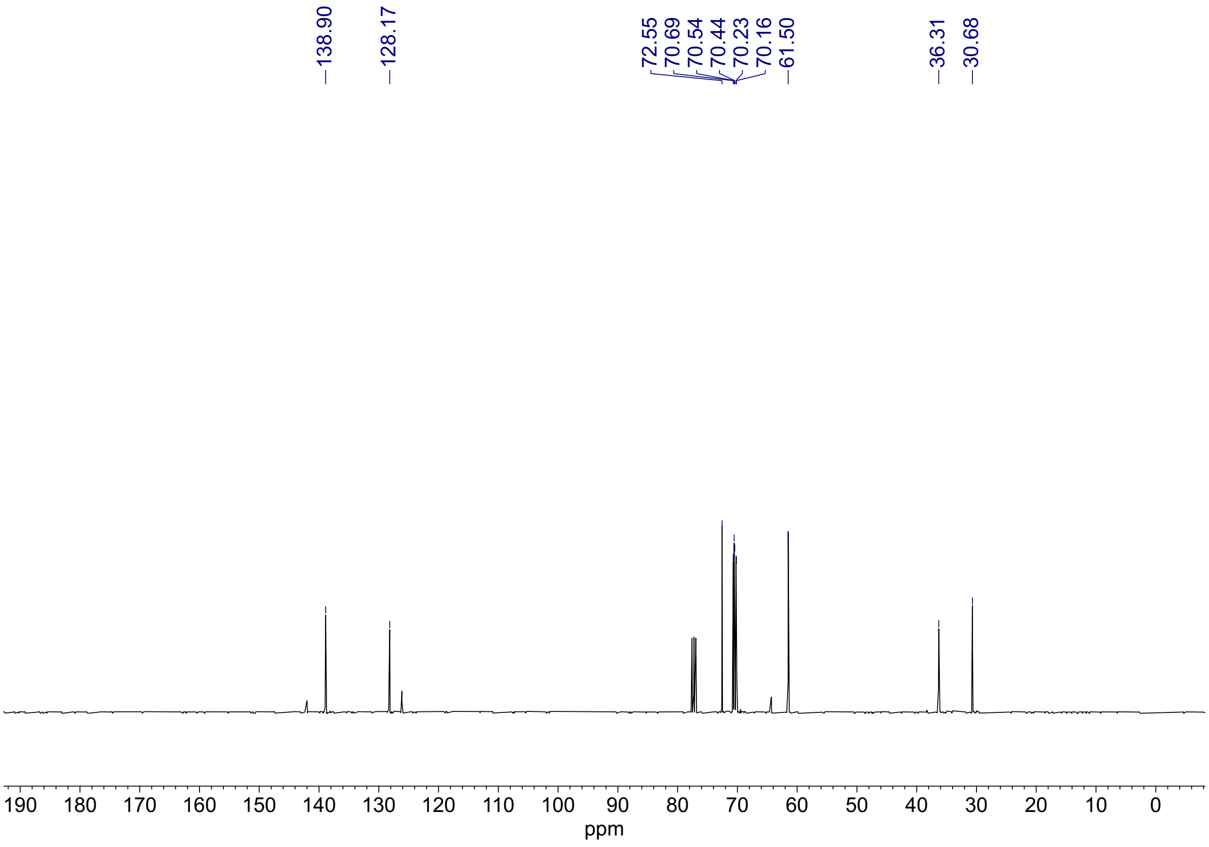

Figure S29. ^{13}C-NMR ($CDCl_3$, 101 MHz) of B3PEG4OH

B3PEG4S

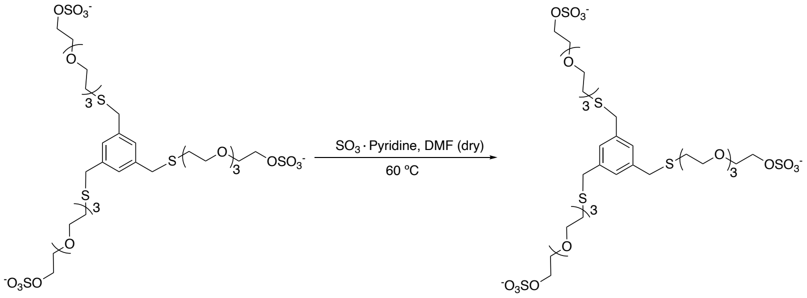

From compound B3PEG4OH following the general method B gave compound B3PEG4S as a white powder, yield 55.1%. ^{1}H NMR (400 MHz, D_2O) δ 7.29 (s, 3H, Ar-H), 4.30 –4.13 (m, 6H, CH_2-SO_4), 3.96 – 3.53 (m, 42H, CH_2-O), 2.71 (t, J = 6.4 Hz, 6H, S-CH_2-C). ^{13}C NMR (101 MHz, D_2O) δ 139.43, 128.31, 69.73, 69.61, 69.59, 69.55, 69.41, 68.88, 67.67, 35.26, 30.17. HRMS (nanochip-ESI/LTQ-Orbitrap) m/z: $[M]^{3-}$ Calcd for $C_{33}H_{57}O_{21}S_6^{3-}$ 327.0578; Found 327.0562.

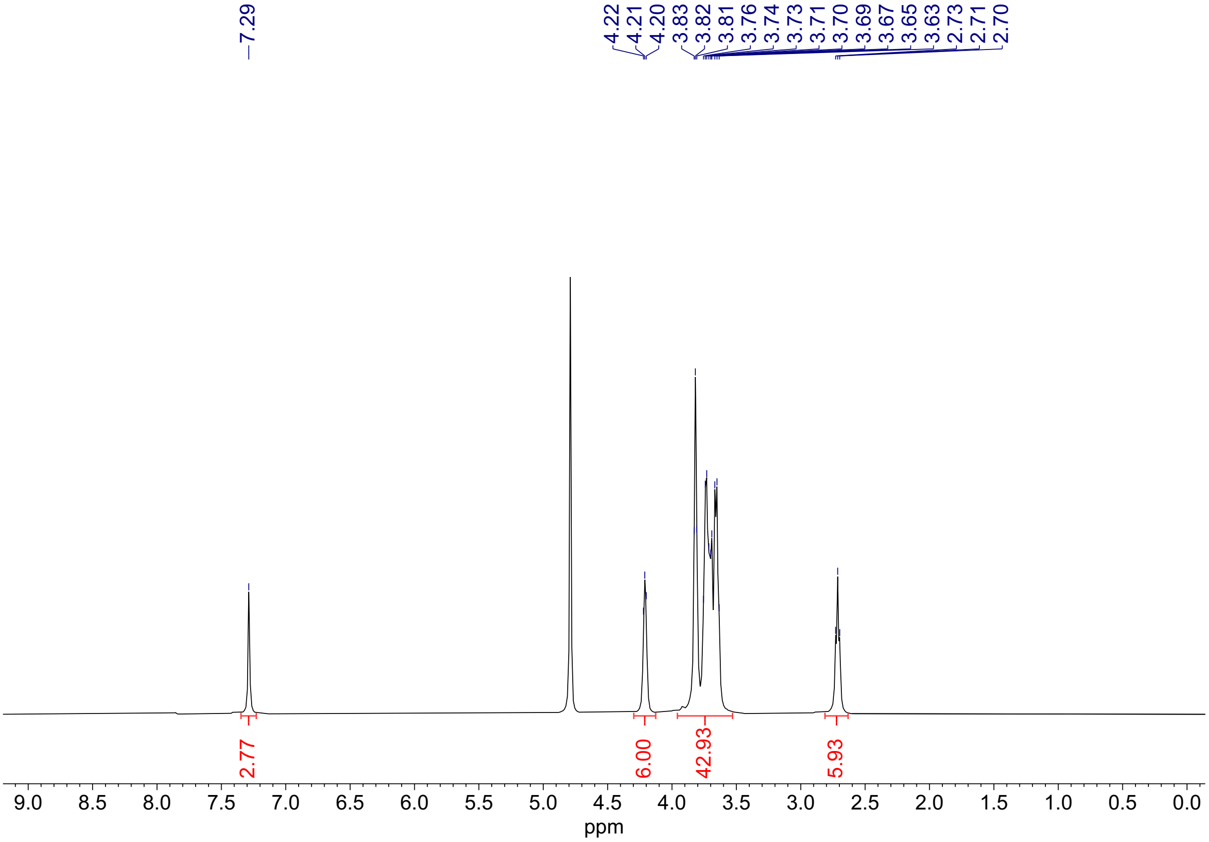

Figure S30. ^{1}H-NMR (D_2O, 400 MHz) of B3PEG4S

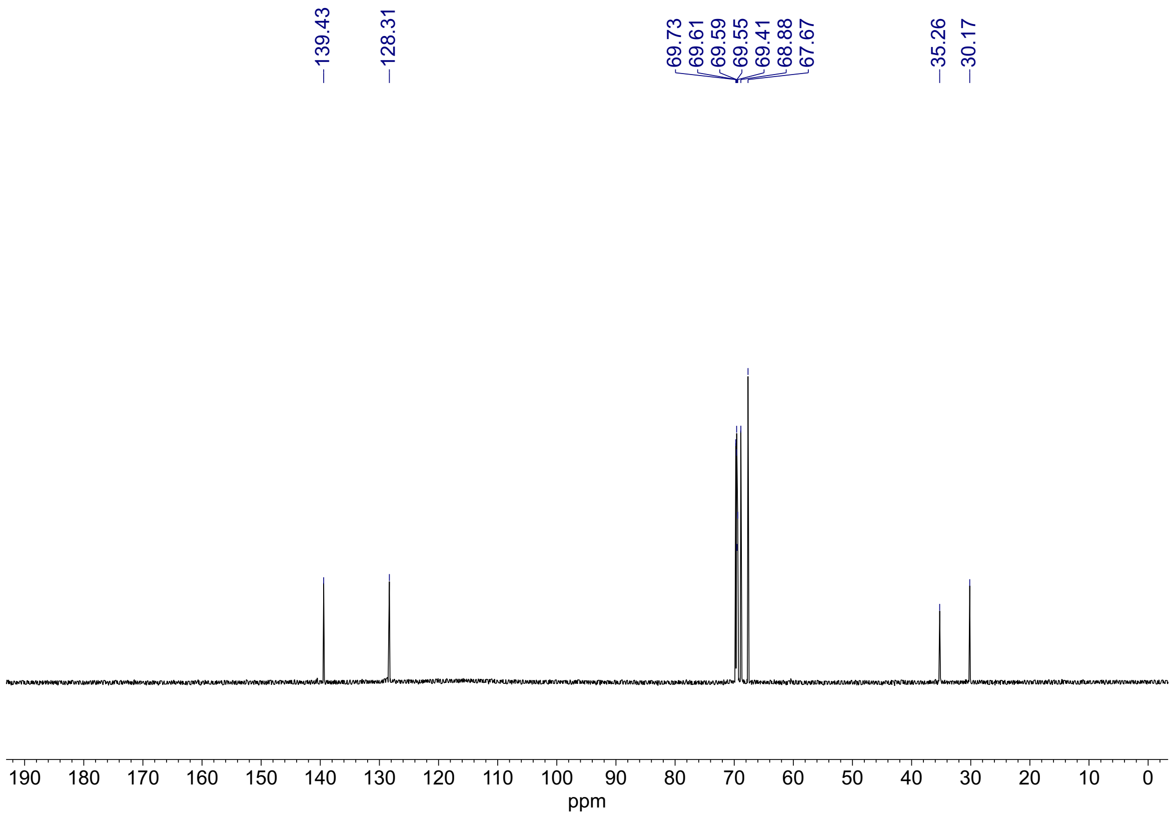

Figure S31. ^{13}C-NMR (D_2O, 101 MHz) of B3PEG4S

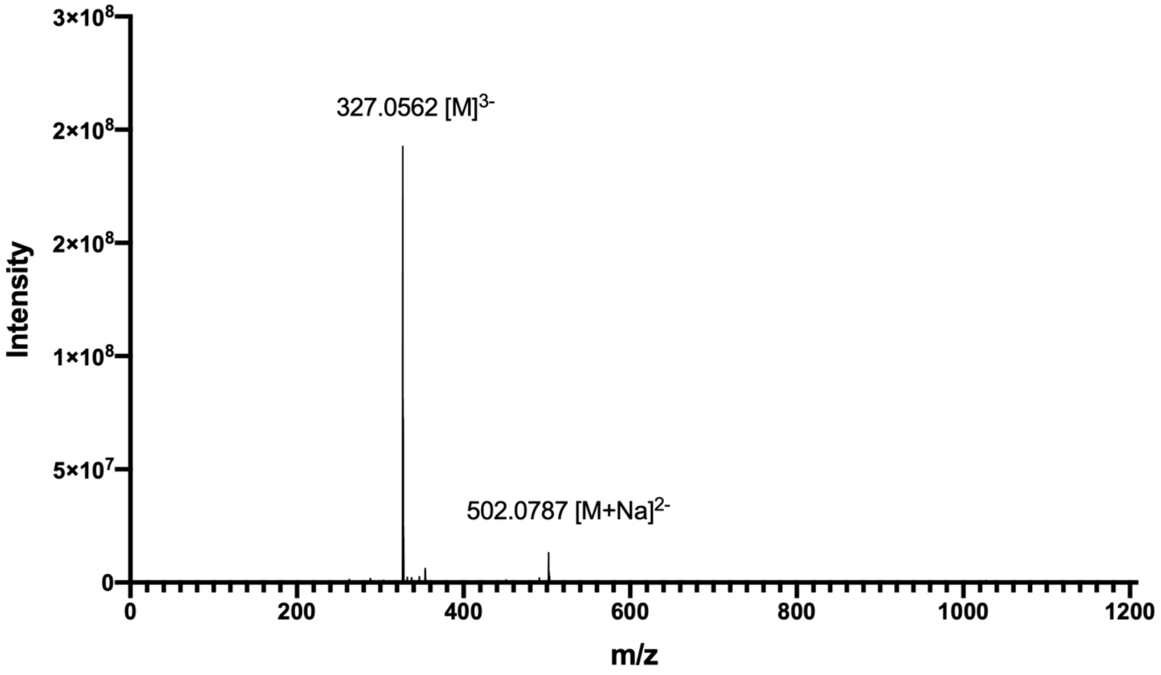

Figure S32. MS (nanochip-ESI/LTQ-orbitrap) of B3PEG4S

B6C11OH

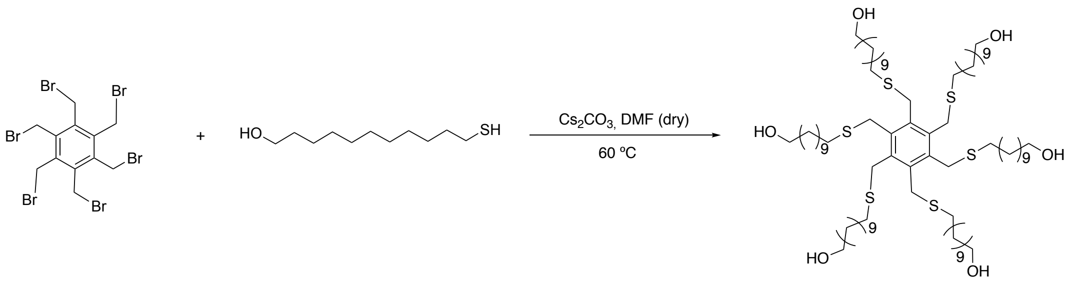

Hexakis(bromomethyl)benzene and 11-mercapto-undenol were used to synthesize compound B6C11OH following the general method A. The product was a white powder, yield 53.6%. ^{1}H NMR (400 MHz, $CDCl_3$) δ 4.00 (s, 12H, Ar-CH_2-S), 3.55 (t, *J* = 6.7 Hz, 12H, CH_2-O), 2.68 – 2.52 (m, 18H, S-CH_2-C, OH), 1.66 – 1.18 (m, 108H, C-CH_2-C). ^{13}C NMR (101 MHz, $CDCl_3$) δ 135.81, 62.73, 62.72, 33.74, 32.71, 30.94, 29.82, 29.67, 29.63, 29.60, 29.50, 29.36, 29.03, 25.81. HRMS (ESI/QTOF) m/z: $[M + H]^+$ Calcd for $C_{78}H_{151}O_6S_6^+$ 1375.9829; Found 1375.9830.

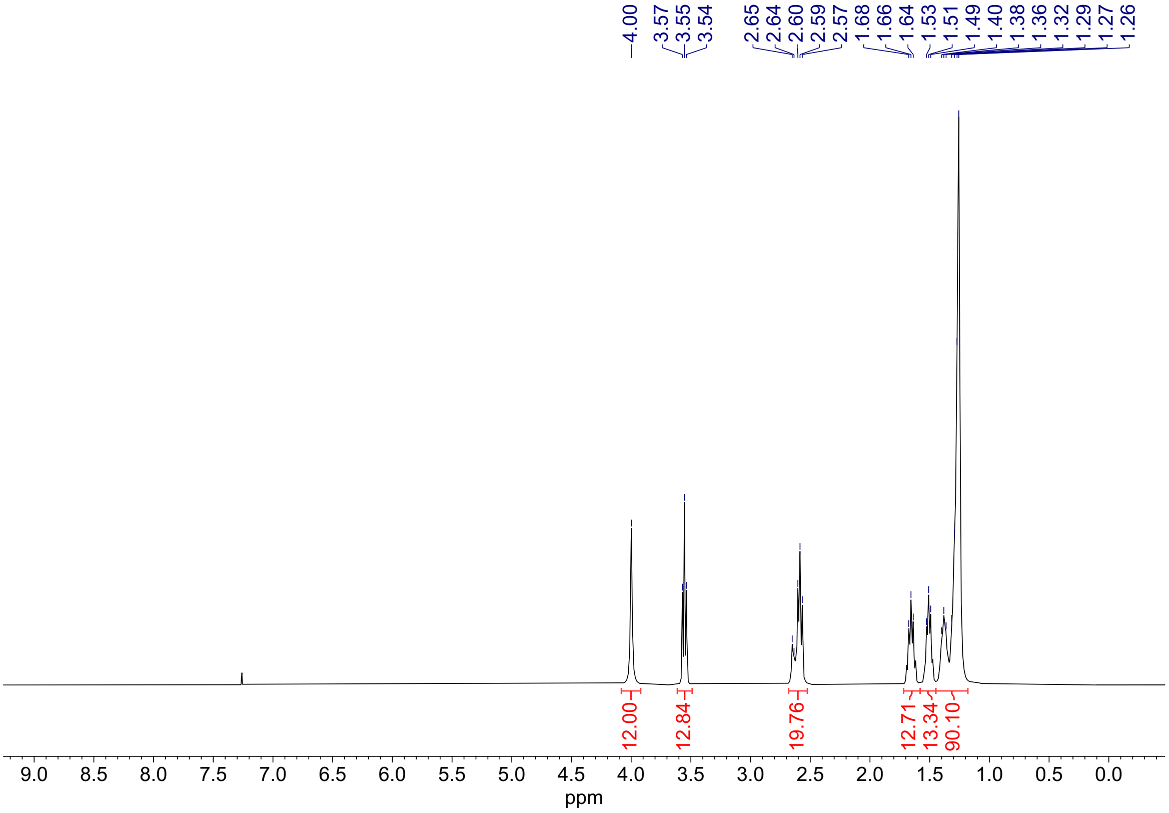

Figure S33. ^{1}H-NMR ($CDCl_3$, 400 MHz) of B6C11OH

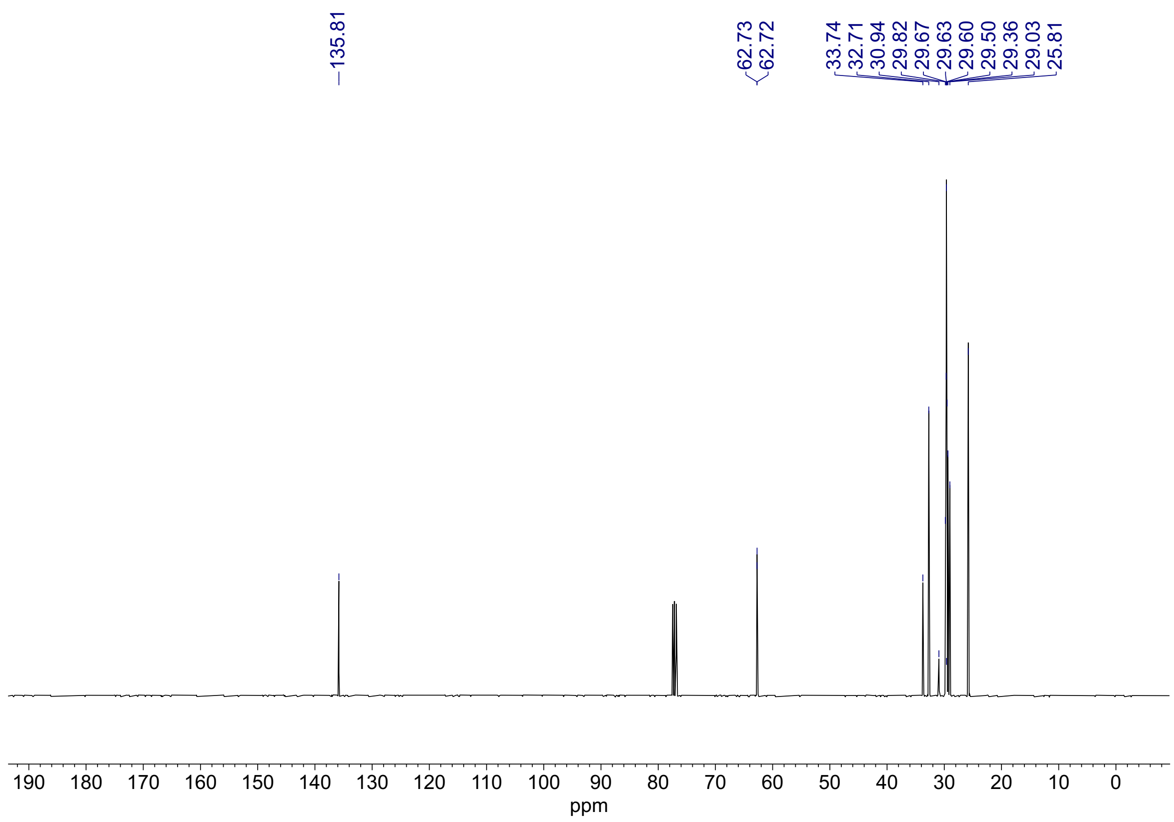

Figure S34. ^{13}C-NMR ($CDCl_3$, 101 MHz) of B6C11OH

B6C11S

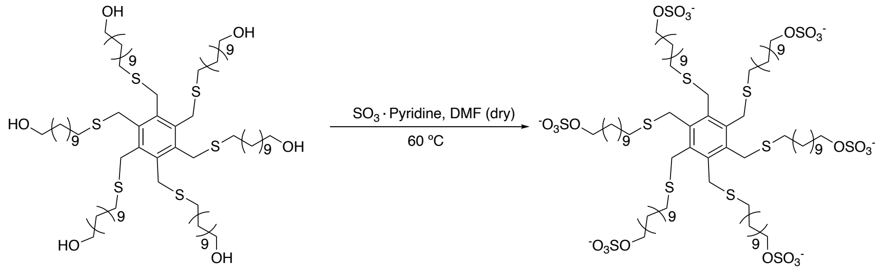

From compound B6C11OH following the general method B gave compound B6C11S as a white powder, yield 65.4%. ^{1}H NMR (400 MHz, D_2O) δ 4.13 – 3.71 (m, 24H, Ar-CH_2, CH_2-SO_4), 2.47 (s, 12H, S-CH_2-C), 1.72 – 0.92 (m, 108H, C-CH_2-C). ^{13}C NMR (101 MHz, D_2O) δ 136.33, 69.72, 34.02, 31.29, 30.45, 30.34, 30.12, 29.87, 29.57, 26.02. HRMS (nanochip-ESI/LTQ-Orbitrap) m/z: $[M]^{5-}$ Calcd for $C_{78}H_{144}NaO_{24}S_{12}^{5-}$ 374.3319; Found 374.3334.

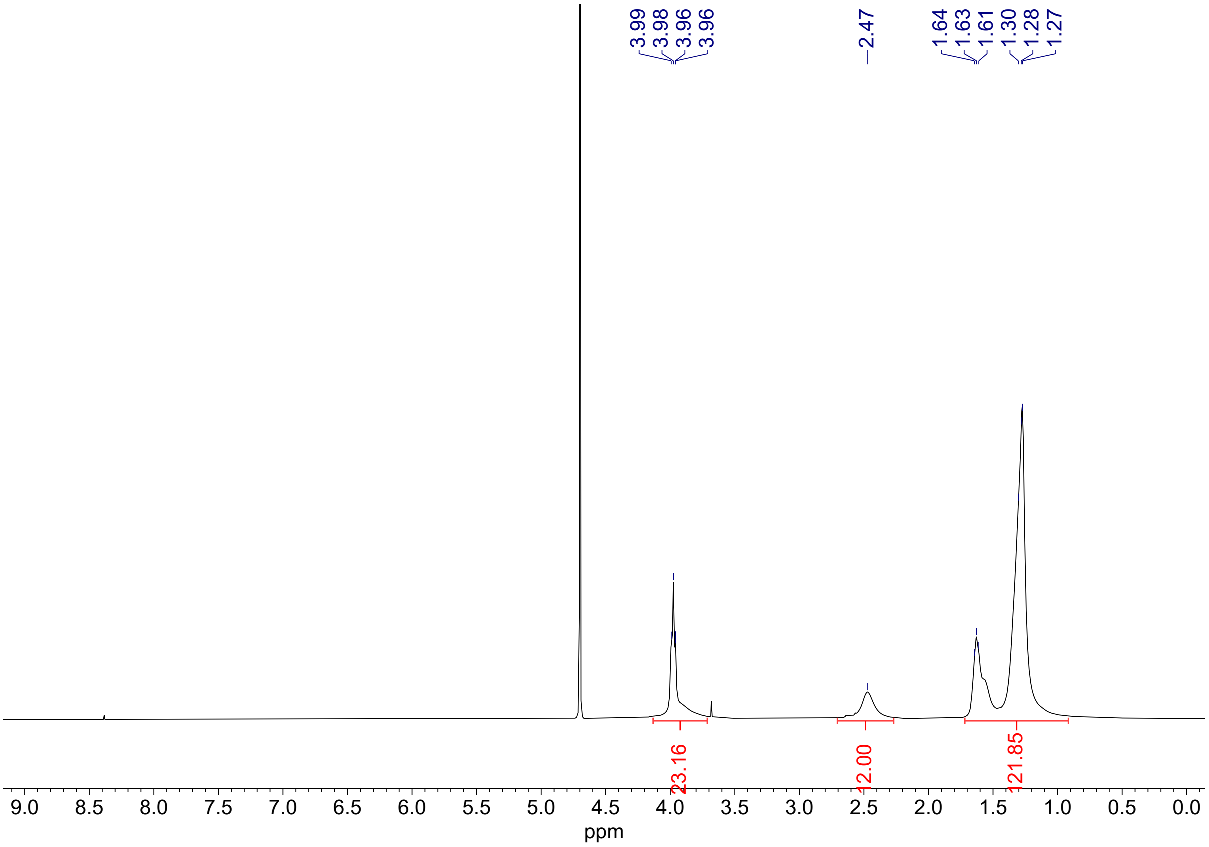

Figure S35. ^{1}H-NMR (D_2O, 400 MHz) of B6C11S

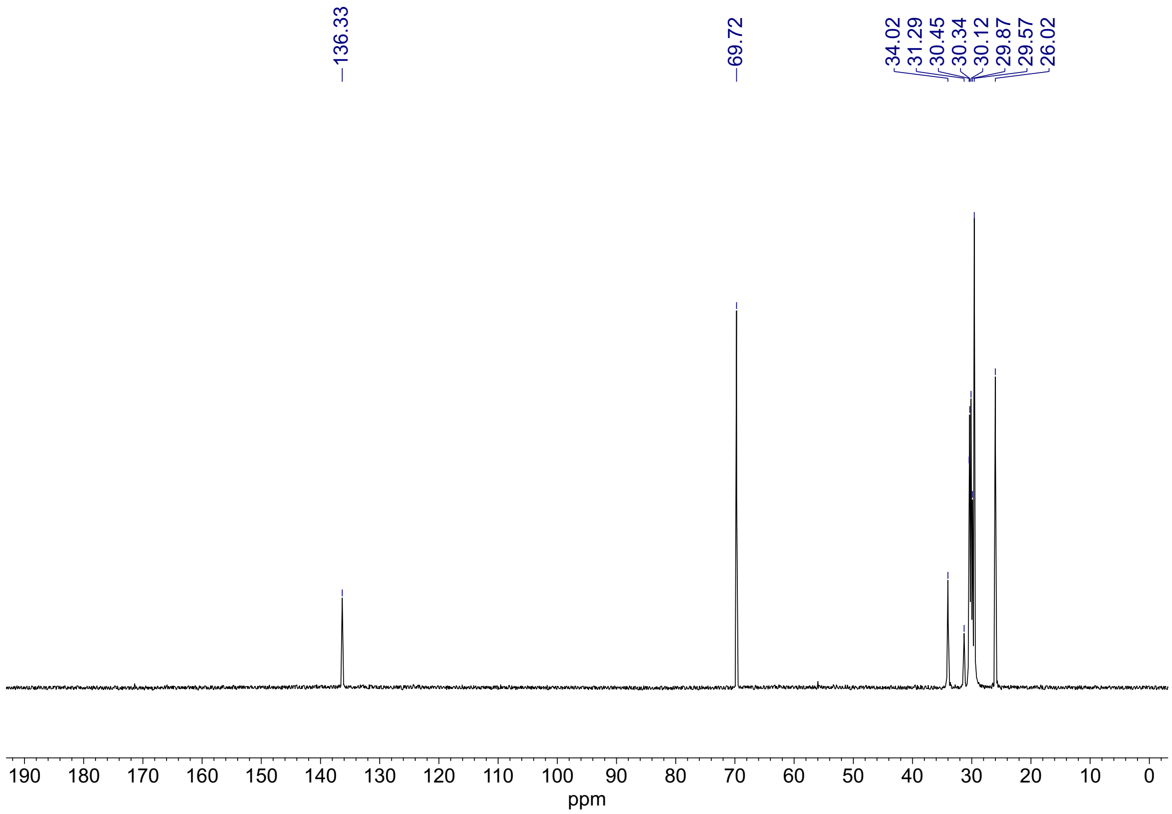

Figure S36. ^{13}C-NMR (D_2O, 101 MHz) of B6C11S

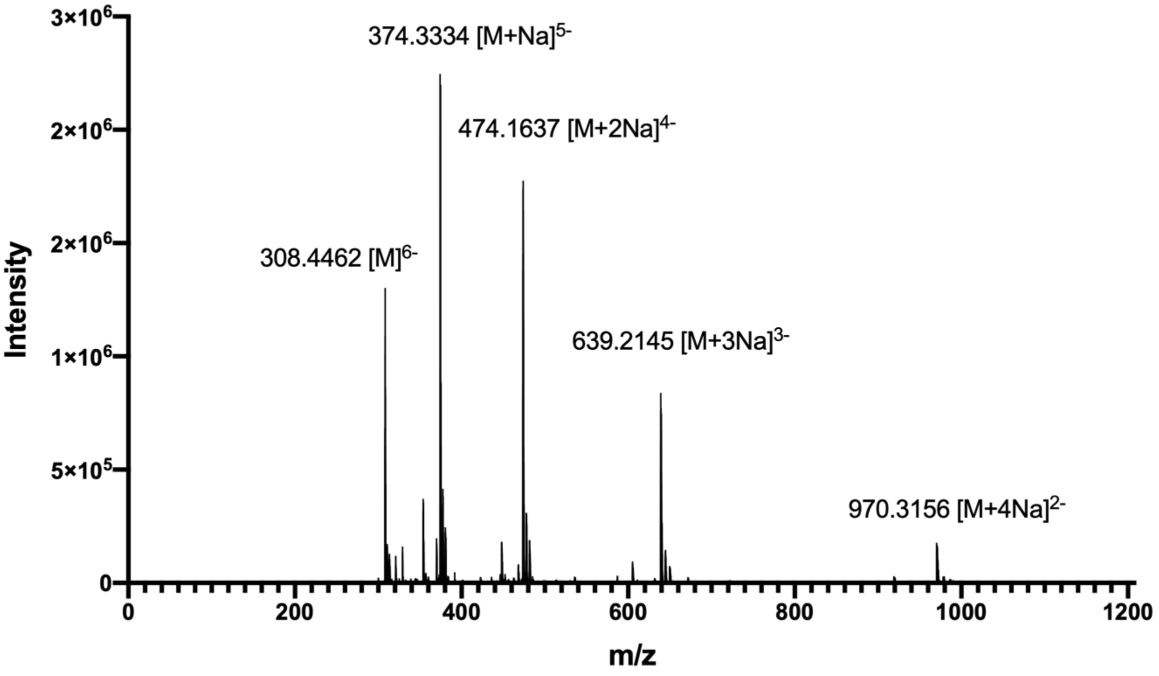

Figure S37. MS (nanochip-ESI/LTQ-orbitrap) of B6C11S

B6C9OH

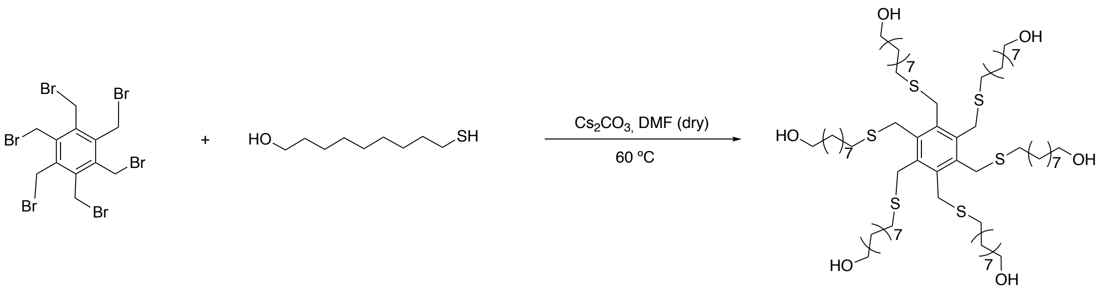

Hexakis(bromomethyl)benzene and 9-mercapto-1-nonanol were used to synthesize compound B6C11OH following the general method A. The product was a white powder, yield 53.6%. ^{1}H NMR (400 MHz, $CDCl_3$) δ 4.00 (s, 12H, Ar-CH_2), 3.55 (t, J = 6.7 Hz, 12H, $\underline{CH_2}$-OH), 2.82 (s, 6H, CH_2-$\underline{OH}$), 2.59 (t, J = 7.4 Hz, 12H, S-$\underline{CH_2}$-CH_2), 1.71 – 1.21 (m, 84H, CH_2-$\underline{CH_2}$-CH_2). ^{13}C NMR (101 MHz, $CDCl_3$) δ 135.79, 62.64, 33.71, 32.68, 30.92, 29.79, 29.60, 29.46, 29.28, 28.99, 25.79. HRMS (nanochip-ESI/LTQ-Orbitrap) m/z: $[M + Na]^+$ Calcd for $C_{66}H_{126}NaO_6S_6^+$ 1229.7771; Found 1229.7783.

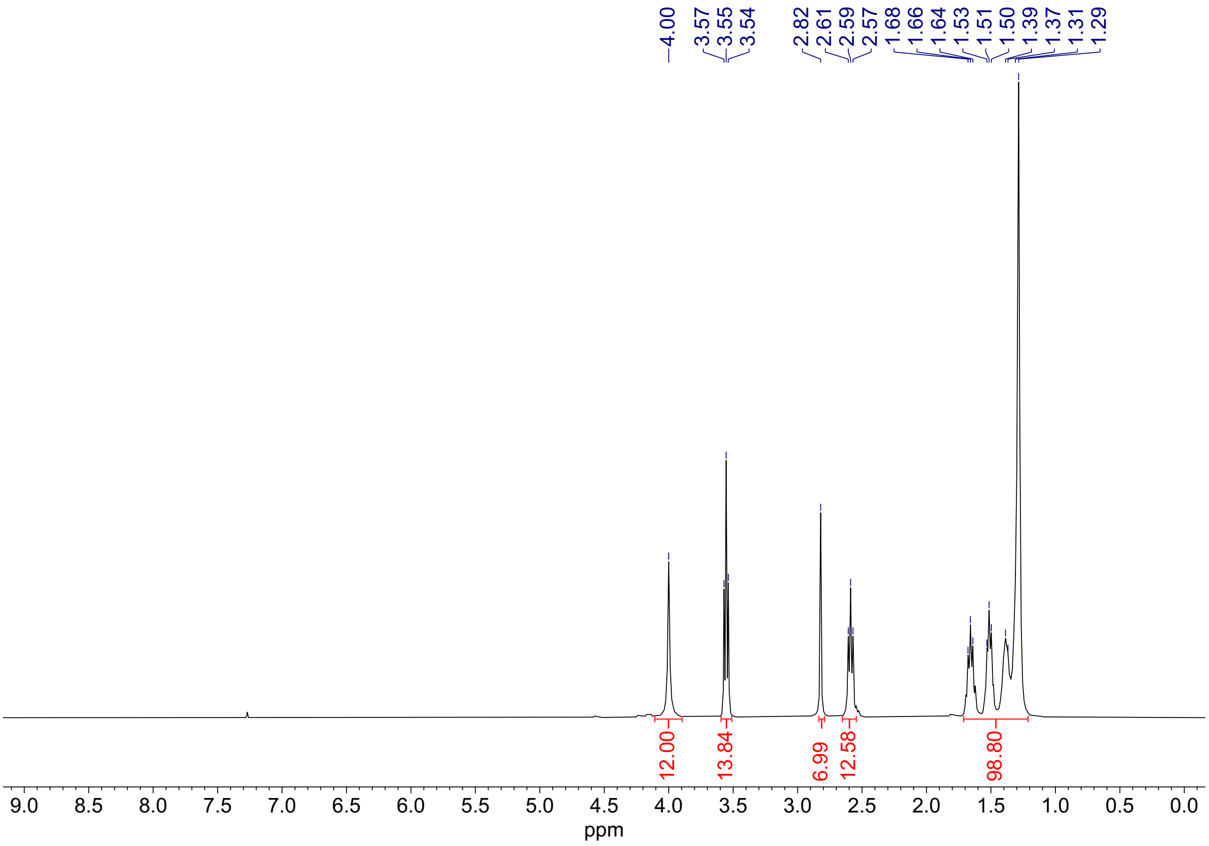

Figure S38. ^{1}H-NMR ($CDCl_3$, 400 MHz) of B6C9OH

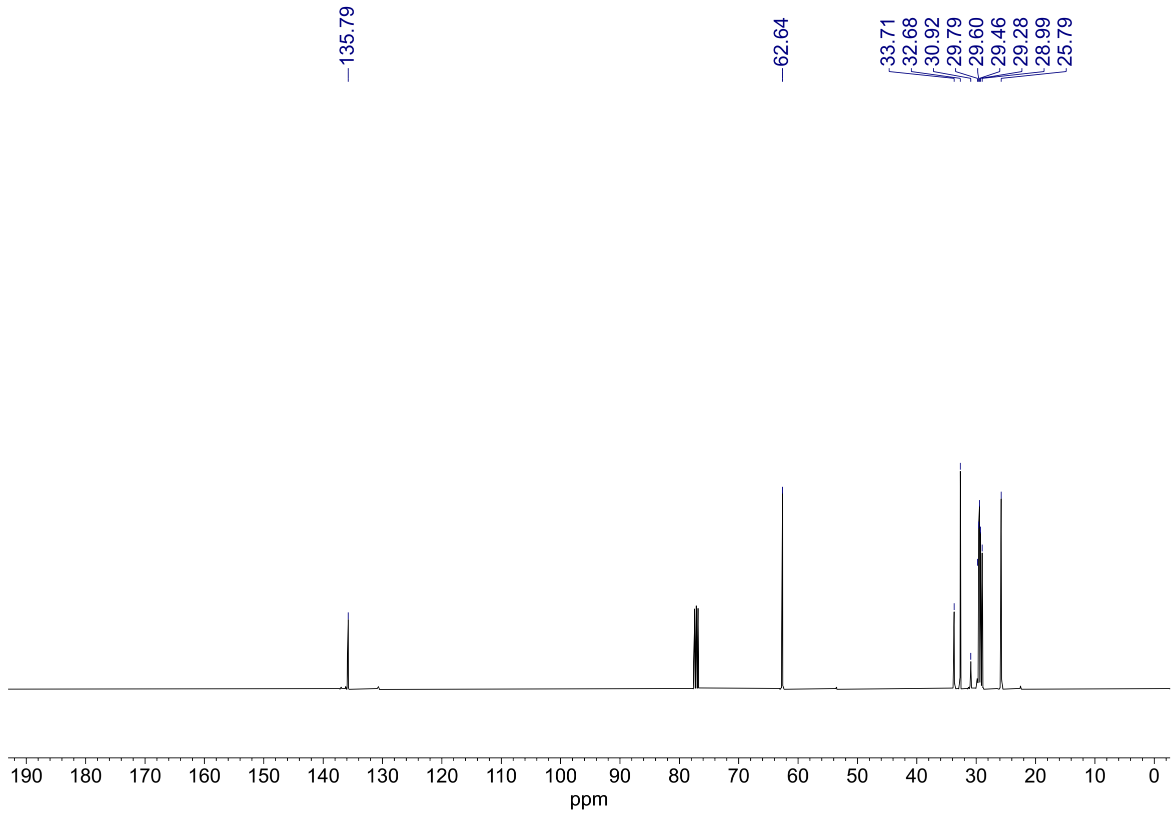

Figure S39. ^{13}C-NMR ($CDCl_3$, 101 MHz) of B6C9OH

B6C9S

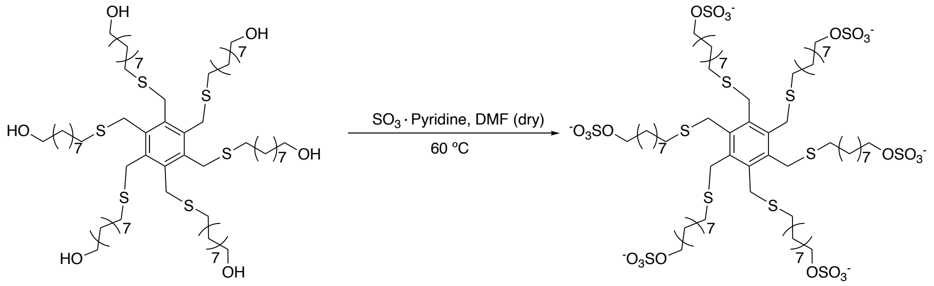

From compound B6C9OH following the general method B gave compound B6C9S as a white powder, yield 65.4%. ^{1}H NMR (400 MHz, D_2O) δ 4.07 (m, 24H, Ar-CH_2, CH_2-OSO_3^-), 2.61 (s, 12H, S-CH_2-C), 1.80 – 1.13 (m, 84H, C-CH_2-C). ^{13}C NMR (101 MHz, D_2O) δ 136.36, 69.77, 33.89, 31.20, 30.23, 29.79, 29.64, 29.58, 29.45, 29.23, 25.87. HRMS (nanochip-ESI/LTQ-Orbitrap) m/z: $[M]^{6-}$ Calcd for $C_{66}H_{120}O_{24}S_{12}^{6-}$ 280.0809; Found 280.0795.

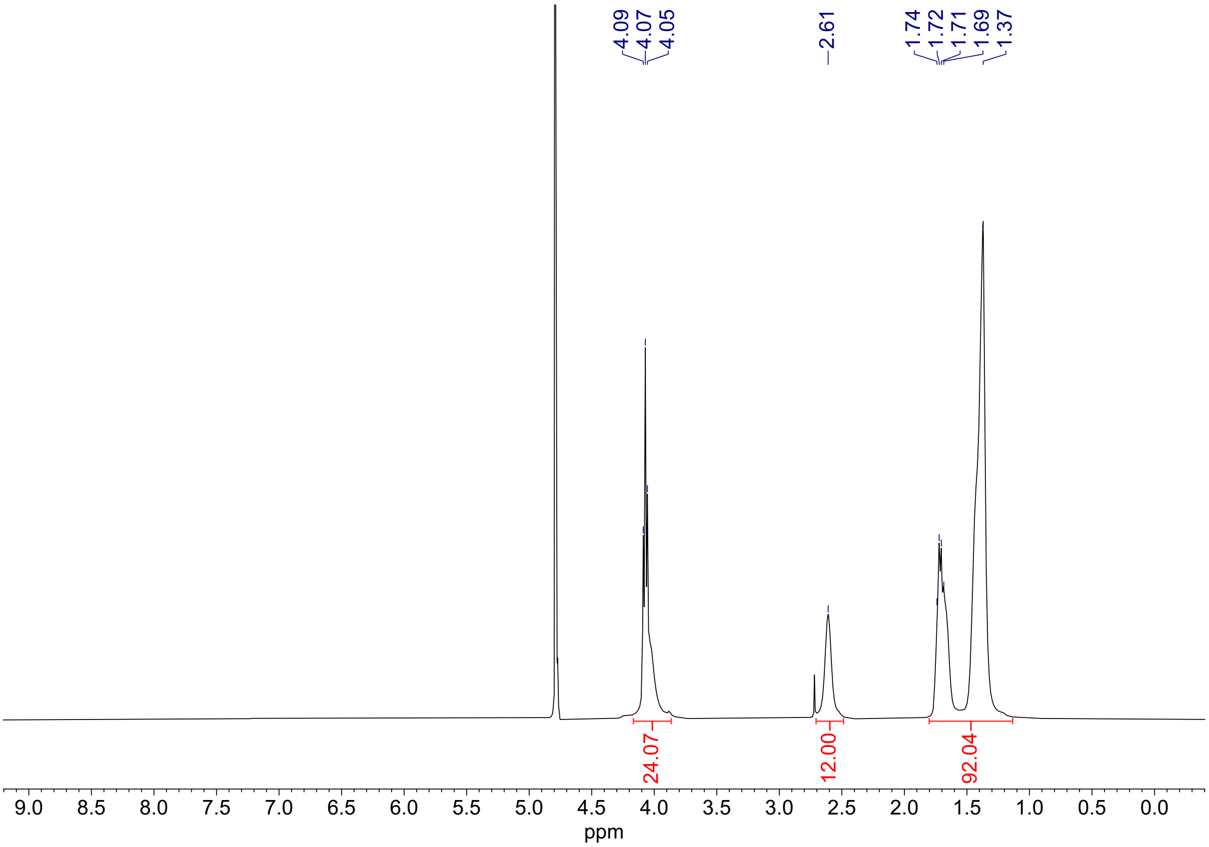

Figure S40. ^{1}H-NMR (D_2O, 400 MHz) of B6C9S

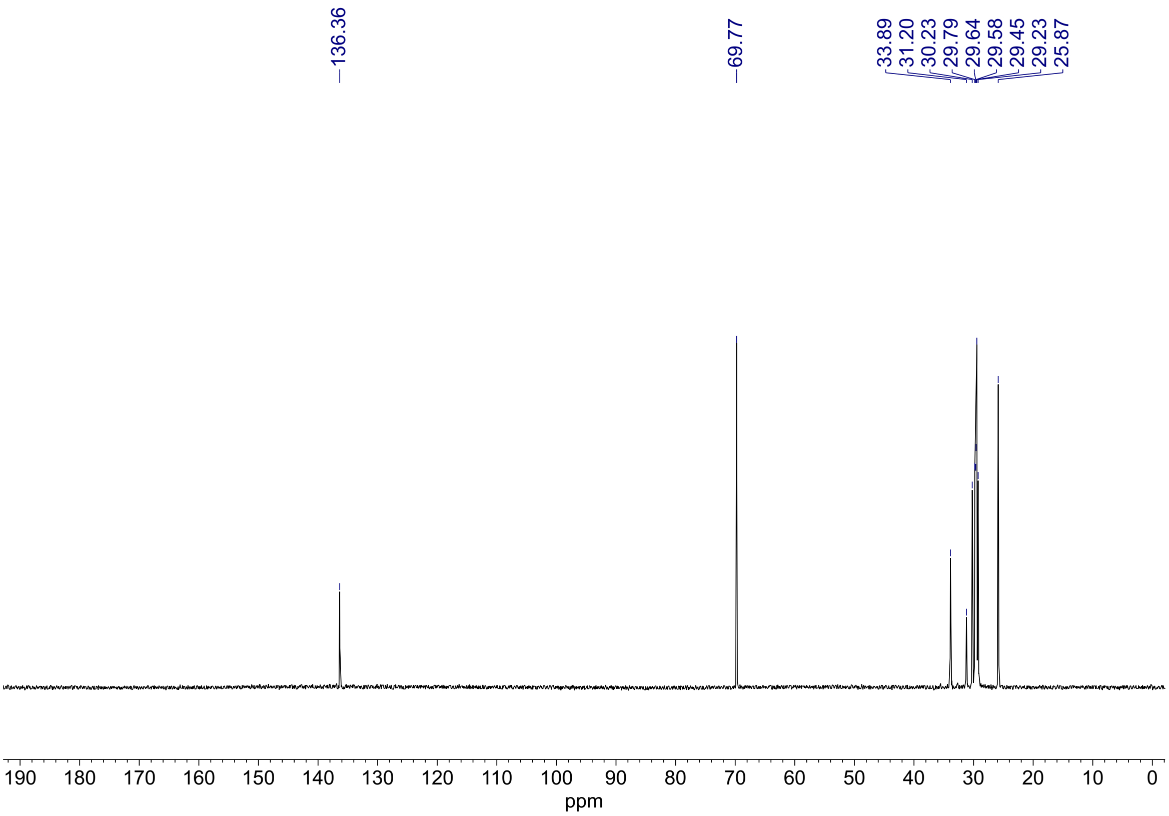

Figure S41. ^{13}C-NMR (D_2O, 101 MHz) of B6C9S

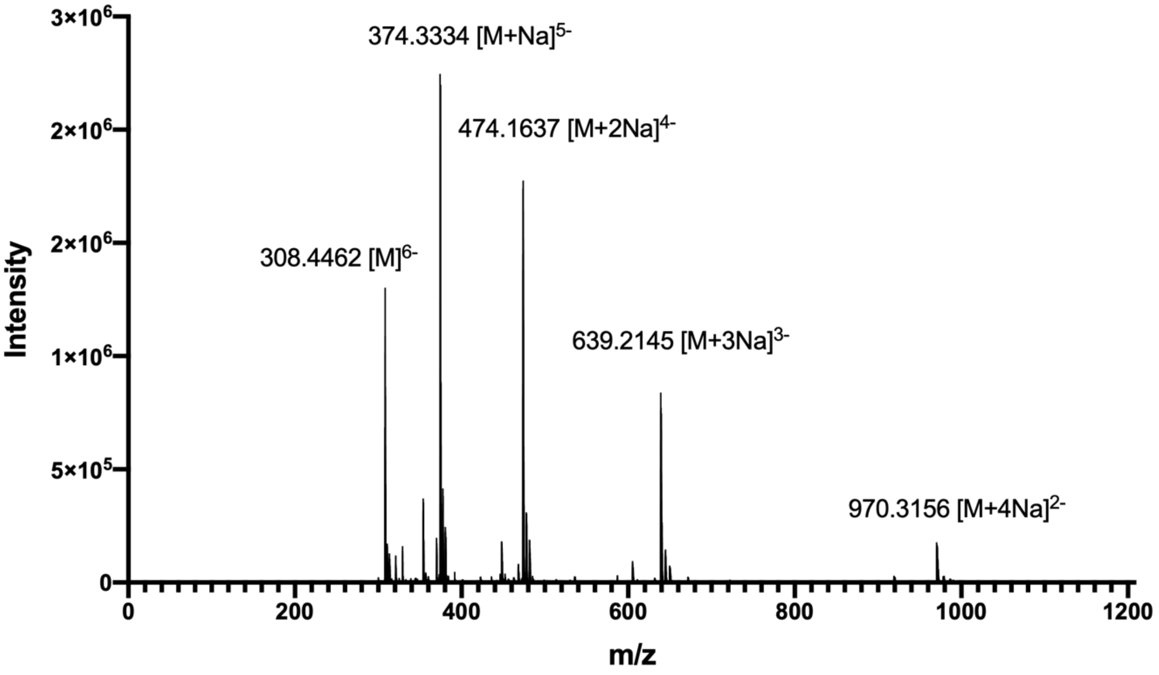

Figure S42. MS (nanochip-ESI/LTQ-orbitrap) of B6C9S

OB3C9OH

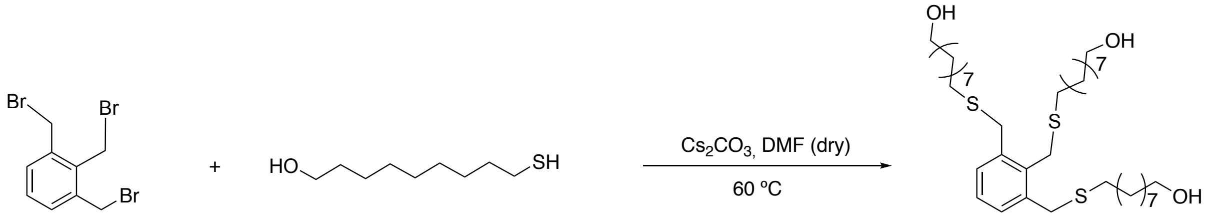

1,2,3-tris(bromomethyl)-benzene and 9-mercapto-1-nonanol were used to synthesize compound OB3C9OH following the general method A. The product was a white powder, yield 54.6%. ^{1}H NMR (400 MHz, CDCl$_3$) δ 7.19 – 7.04 (m, 3H, Ar-H), 4.04 (s, 2H, Ar-CH$_2$), 3.85 (s, 4H, Ar-CH$_2$), 3.59 (t, J = 6.7 Hz, 6H, CH$_2$-OH), 2.59 (t, J = 7.4 Hz, 2H, S-CH$_2$-CH$_2$), 2.47 (t, J = 7.4 Hz, 4H, S-CH$_2$-CH$_2$), 1.98 (s, 3H, OH), 1.73 – 1.12 (m, 42H, CH$_2$-CH$_2$-CH$_2$). ^{13}C NMR (101 MHz, CDCl$_3$) δ 137.70, 134.88, 129.50, 126.91, 63.02, 34.11, 33.57, 32.76, 32.28, 29.70, 29.52, 29.49, 29.47, 29.39, 29.37, 29.21, 29.18, 28.94, 28.91, 25.73. HRMS (nanochip-ESI/LTQ-Orbitrap) m/z: [M + Na]$^+$ Calcd for $C_{36}H_{66}NaO_3S_3^+$ 665.4066; Found 665.4083.

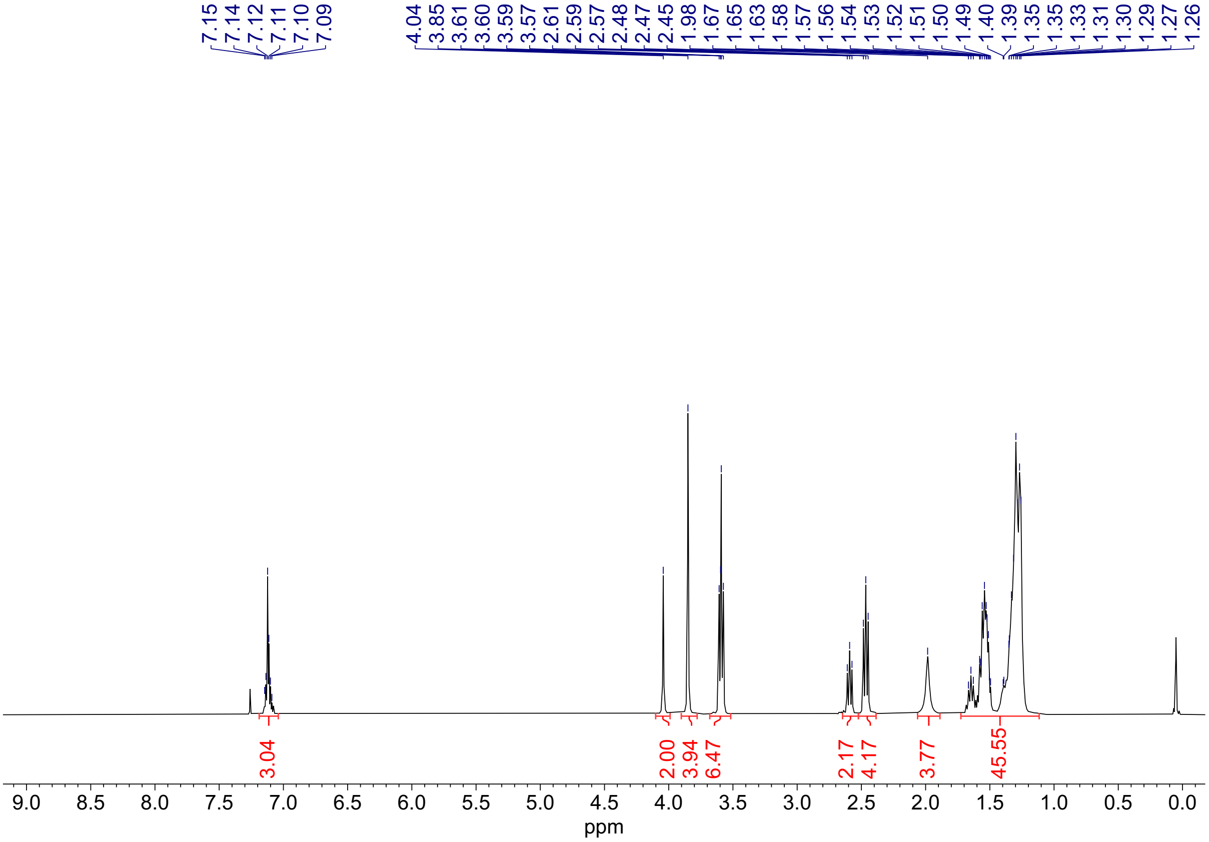

Figure S43. ^{1}H-NMR (CDCl$_3$, 400 MHz) of OB3C9OH

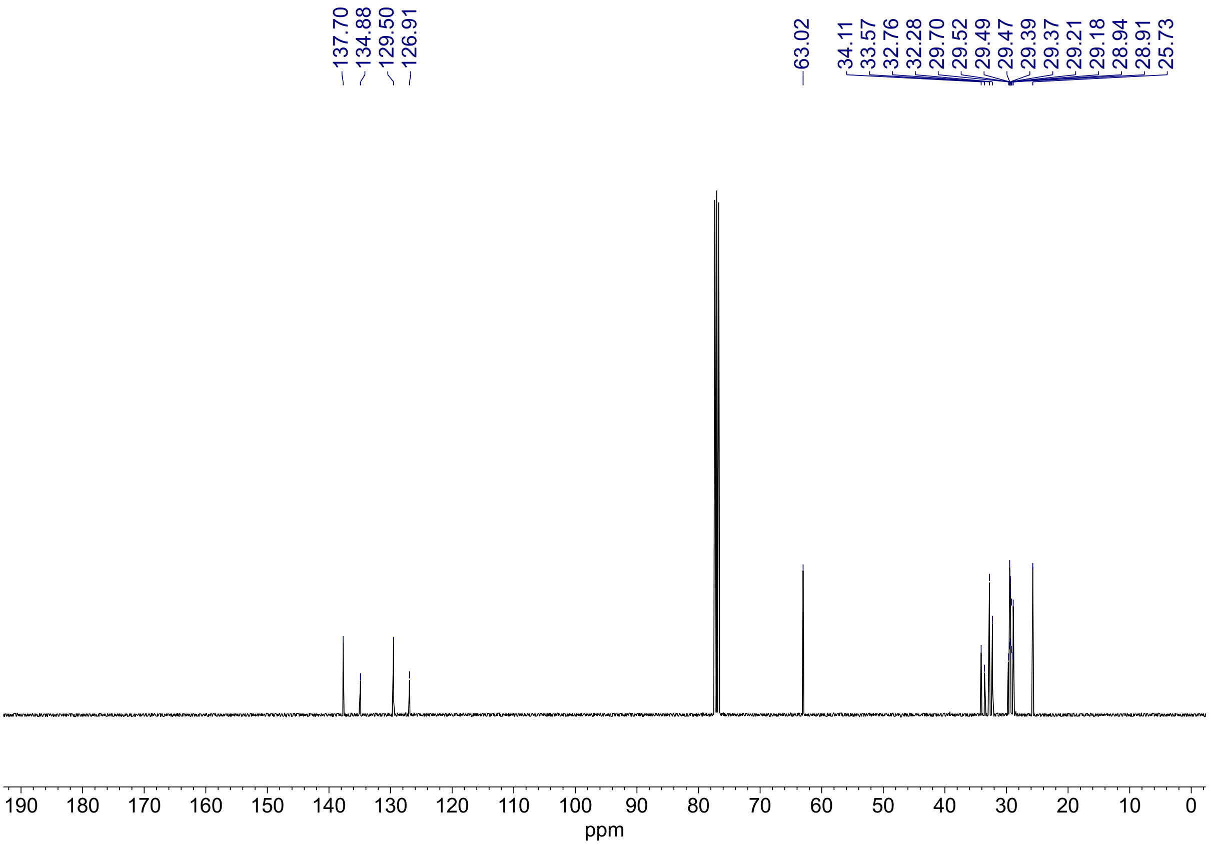

Figure S44. ^{13}C-NMR ($CDCl_3$, 101 MHz) of OB3C9OH

OB3C9S

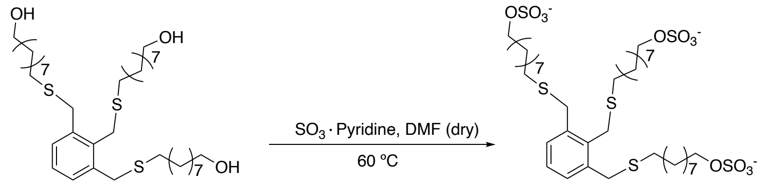

From compound OB3C9OH following the general method B gave compound OB3C9S as a white powder, yield 63.4%. ^{1}H NMR (400 MHz, D_2O) δ 7.30 – 7.03 (m, 3H, Ar-H), 4.14 – 3.78 (m, 12H, Ar-CH_2, CH_2-O), 2.66 – 2.38 (m, 6H, S-CH_2-C), 1.79 – 1.09 (m, 42H, C-CH_2-C). ^{13}C NMR (101 MHz, D_2O) δ 137.56, 134.70, 129.63, 127.16, 69.36, 69.34, 33.81, 33.34, 32.14, 29.58, 29.44, 29.36, 29.29, 29.20, 29.14, 28.94, 28.92, 28.83, 25.45, 25.41. HRMS (nanochip-ESI/LTQ-Orbitrap) m/z: $[M]^{2-}$ Calcd for $C_{36}H_{63}NaO_{12}S_6^{2-}$ 451.1276; Found 451.1254.

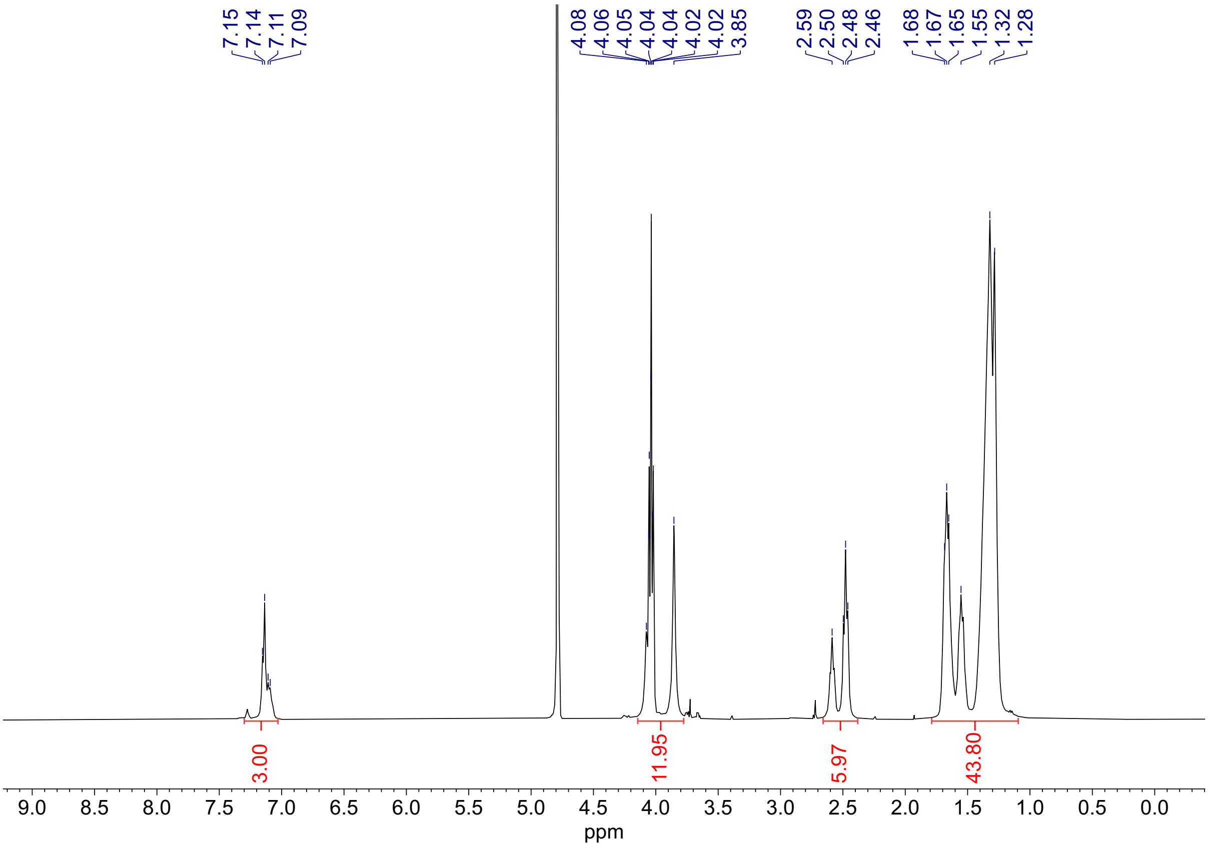

Figure S45. ^{1}H-NMR (D_2O, 400 MHz) of OB3C9S

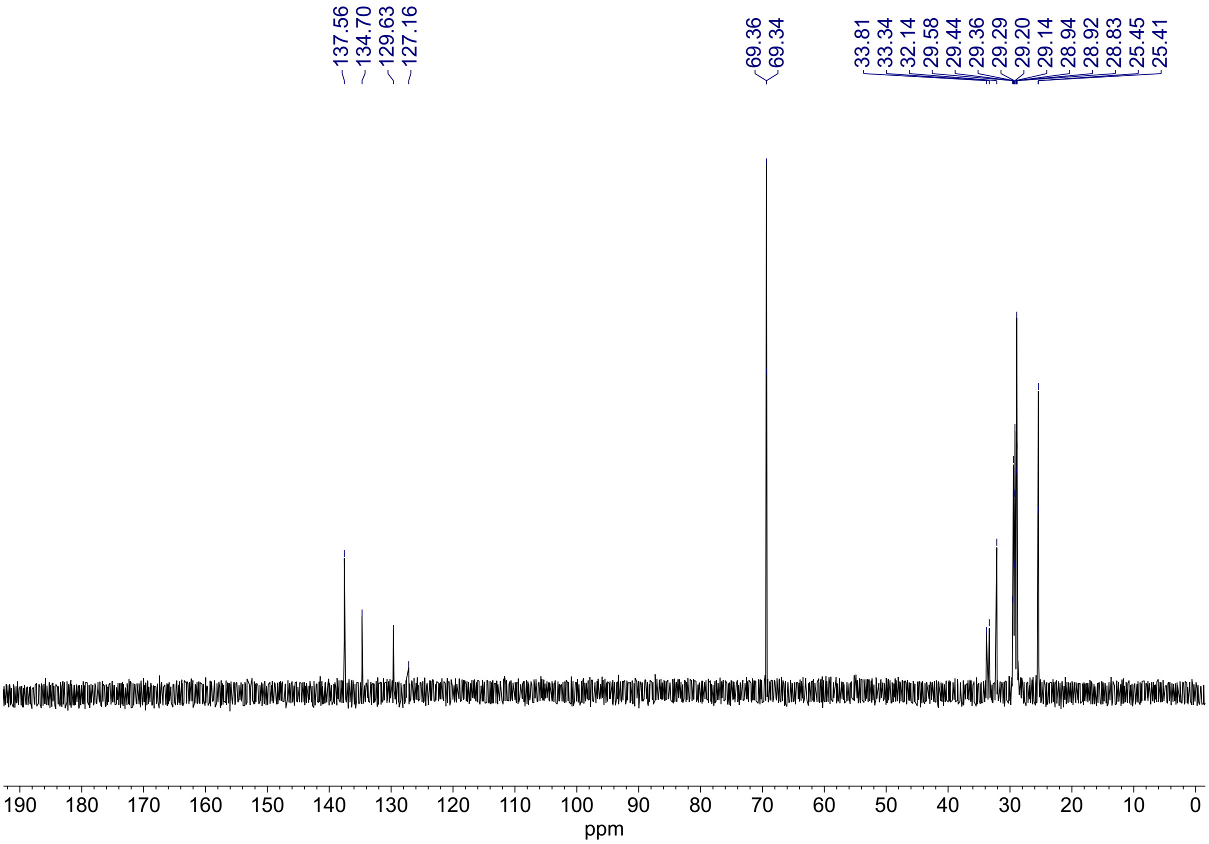

Figure S46. ^{13}C-NMR (D_2O, 101 MHz) of OB3C9S

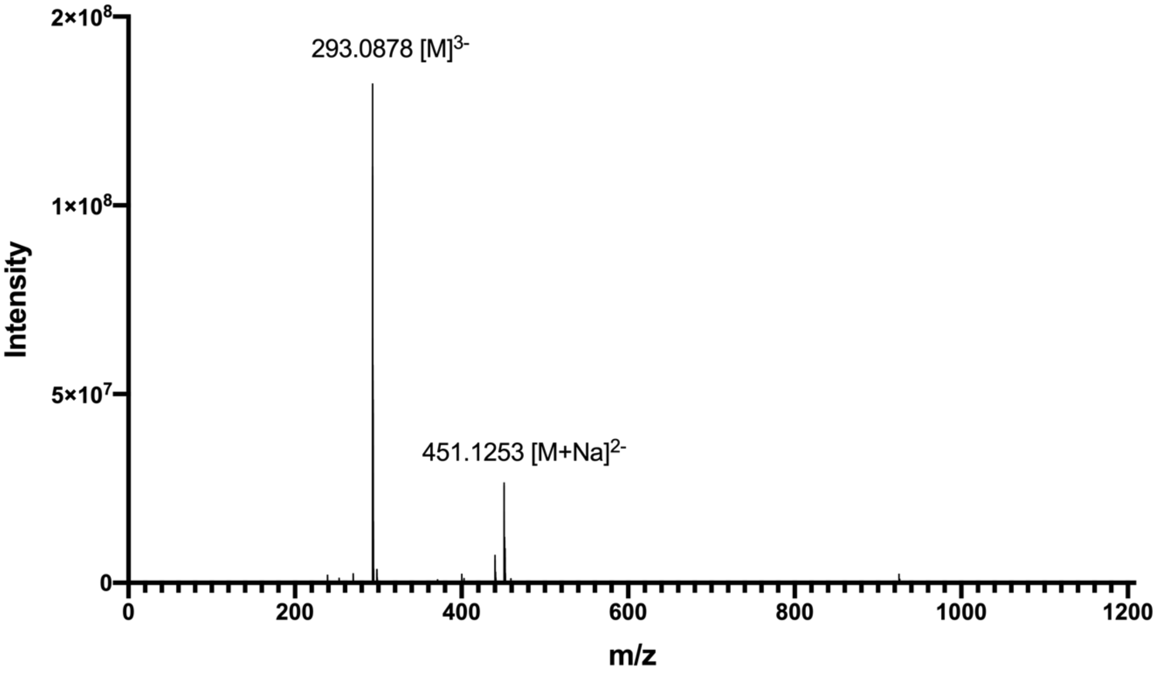

Figure S47. MS (nanochip-ESI/LTQ-orbitrap) of OB3C9S

P4C9OH

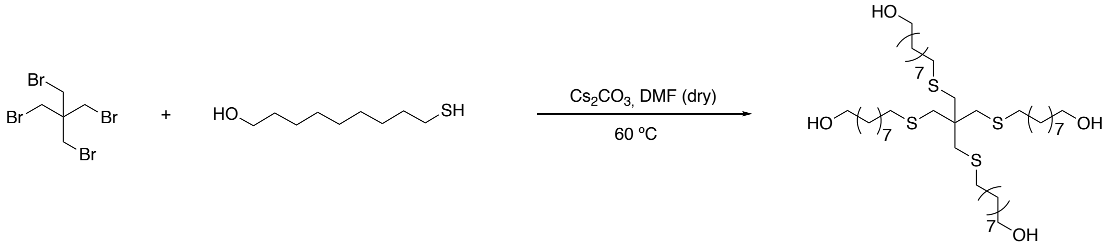

Pentaerythritol tetrabromide and 9-mercapto-1-nonanol were used to synthesize compound P4C9OH following the general method A. The product was a white powder, yield 58.1%. ^{1}H NMR (400 MHz, $CDCl_3$) δ 3.63 (t, J = 6.6 Hz, 8H, CH_2OH), 2.71 (s, 8H, C-CH_2-S), 2.55 (t, J = 7.4 Hz, 8H, S-$\underline{CH_2}$-CH_2), 1.77 (s, 5H, OH), 1.65 – 1.22 (m, 56H, C-CH_2-C). ^{13}C NMR (101 MHz, $CDCl_3$) δ 62.95, 44.10, 38.57, 33.93, 32.75, 29.96, 29.50, 29.39, 29.19, 28.83, 25.75. HRMS (nanochip-ESI/LTQ-Orbitrap) m/z: $[M + Na]^+$ Calcd for $C_{41}H_{84}NaO_4S_4^+$ 791.5145; Found 791.5165.

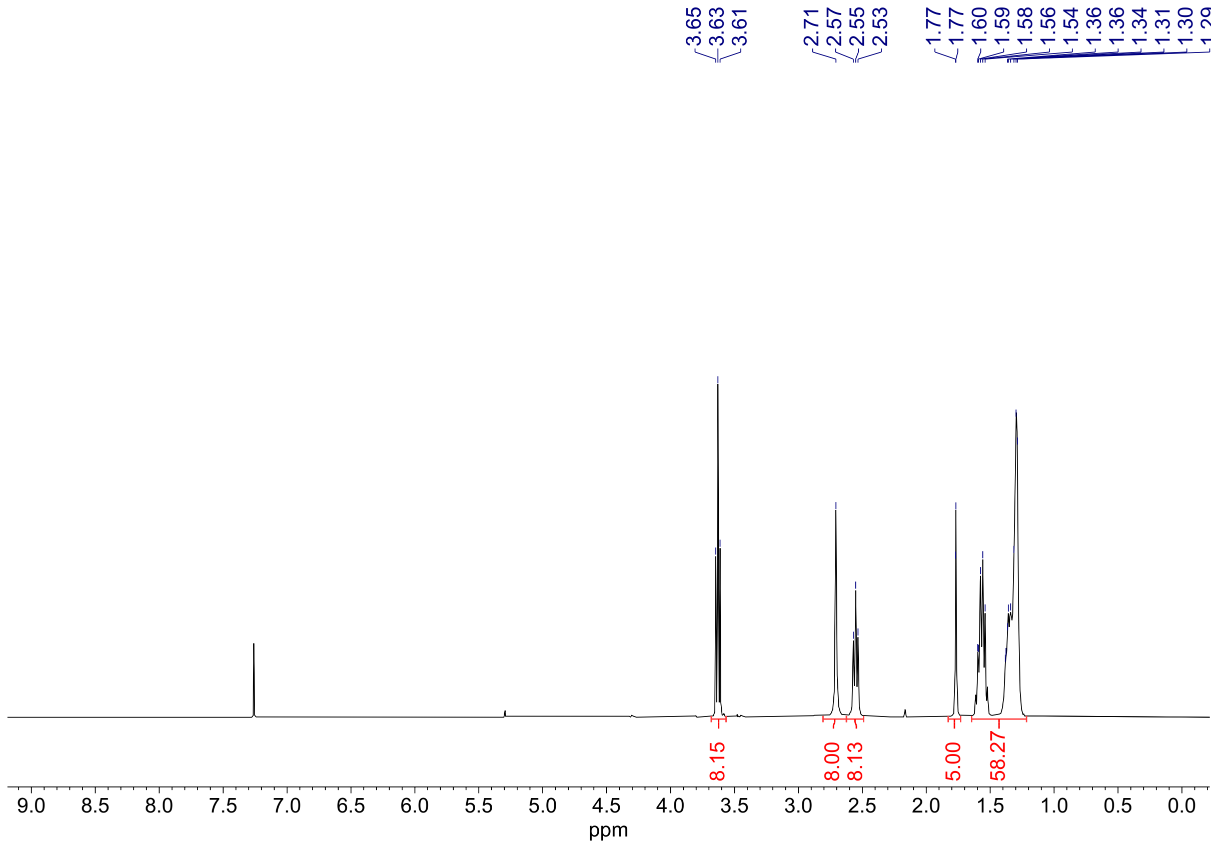

Figure S48. ^{1}H-NMR ($CDCl_3$, 400 MHz) of P4C9OH

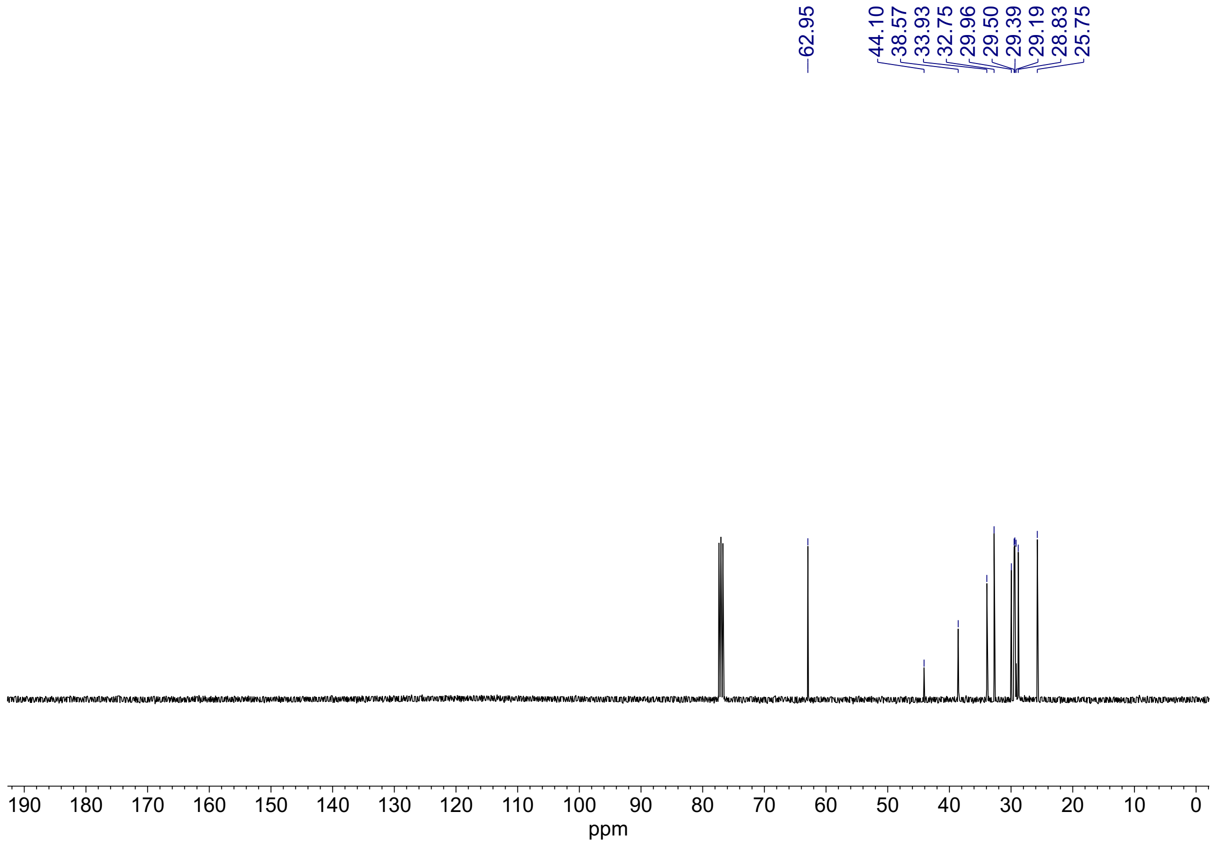

Figure S49. ^{13}C-NMR ($CDCl_3$, 101 MHz) of P4C9OH

P4C9S

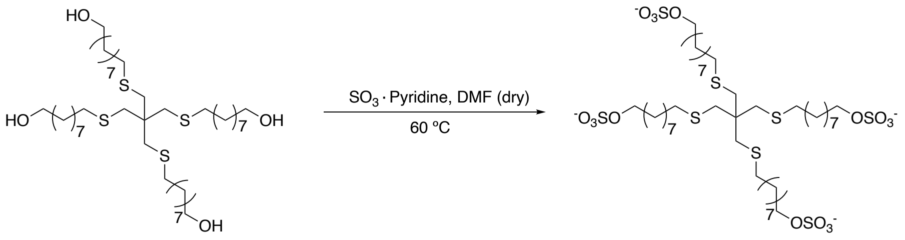

From compound P4C9OH following the general method B gave compound P4C9S as a white powder, yield 65.4%. ^{1}H NMR (400 MHz, D_2O) δ 4.08 (t, *J* = 6.6 Hz, 8H, CH_2OSO3^-), 2.81 (s, 8H, C-CH_2-S), 2.70 (t, *J* = 7.3 Hz, 8H, S-<u>CH_2</u>-CH_2), 1.83 – 1.25 (m, 56H, C-CH_2-C). ^{13}C NMR (101 MHz, D_2O) δ 69.37, 44.11, 38.40, 33.85, 30.11, 29.62, 29.49, 29.36, 29.03, 25.55. HRMS (nanochip-ESI/LTQ-Orbitrap) m/z: $[M]^{4-}$ Calcd for $C_{41}H_{80}O_{16}S_8^{4-}$ 271.0803; Found 271.0781.

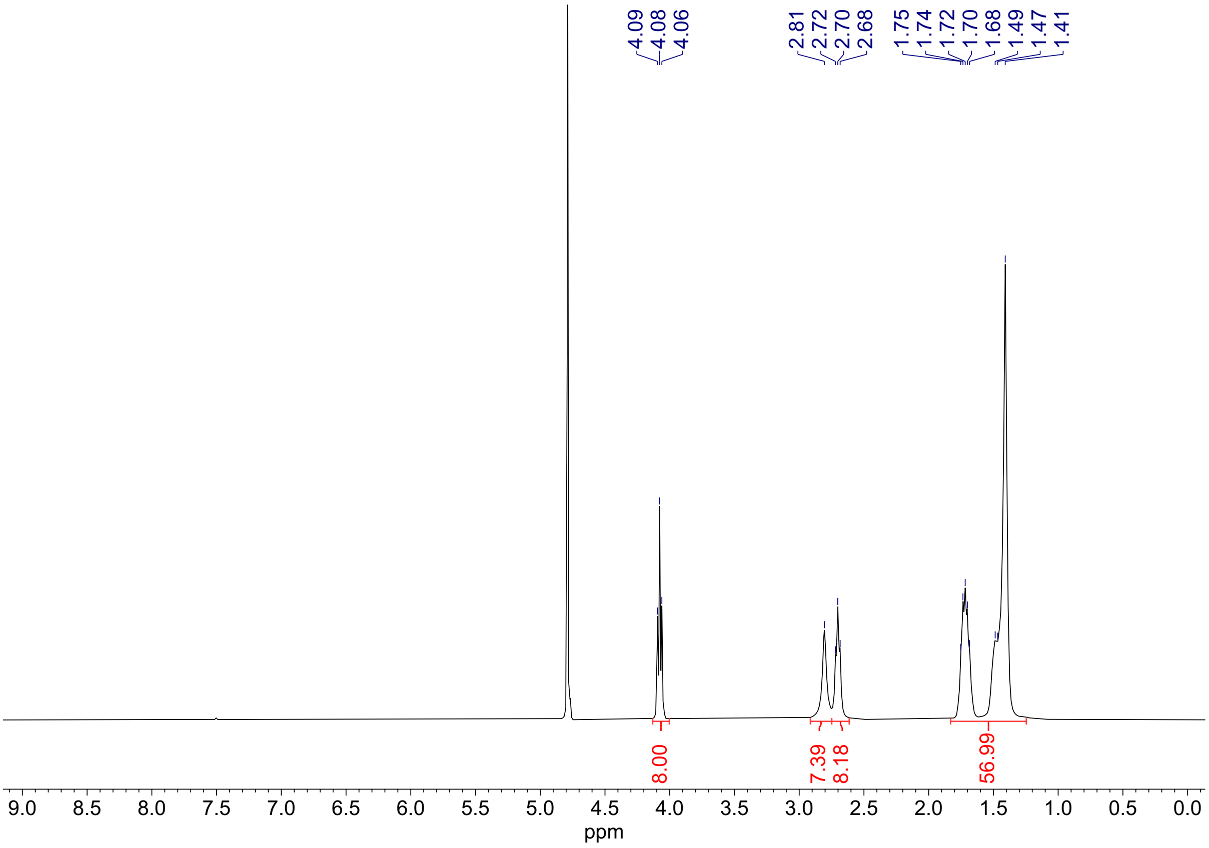

Figure S50. ^{1}H-NMR (D_2O, 400 MHz) of P4C9S

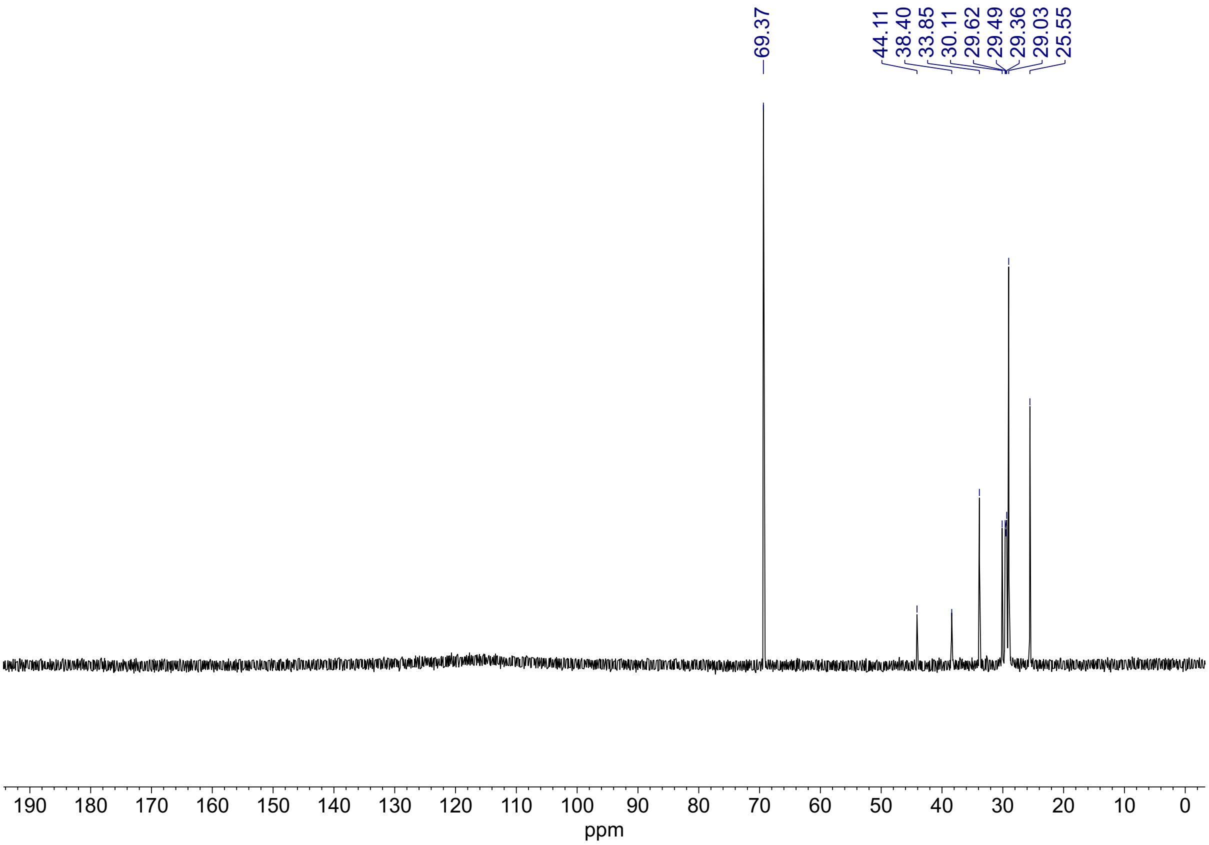

Figure S51. ^{13}C-NMR (D_2O, 101 MHz) of P4C9S

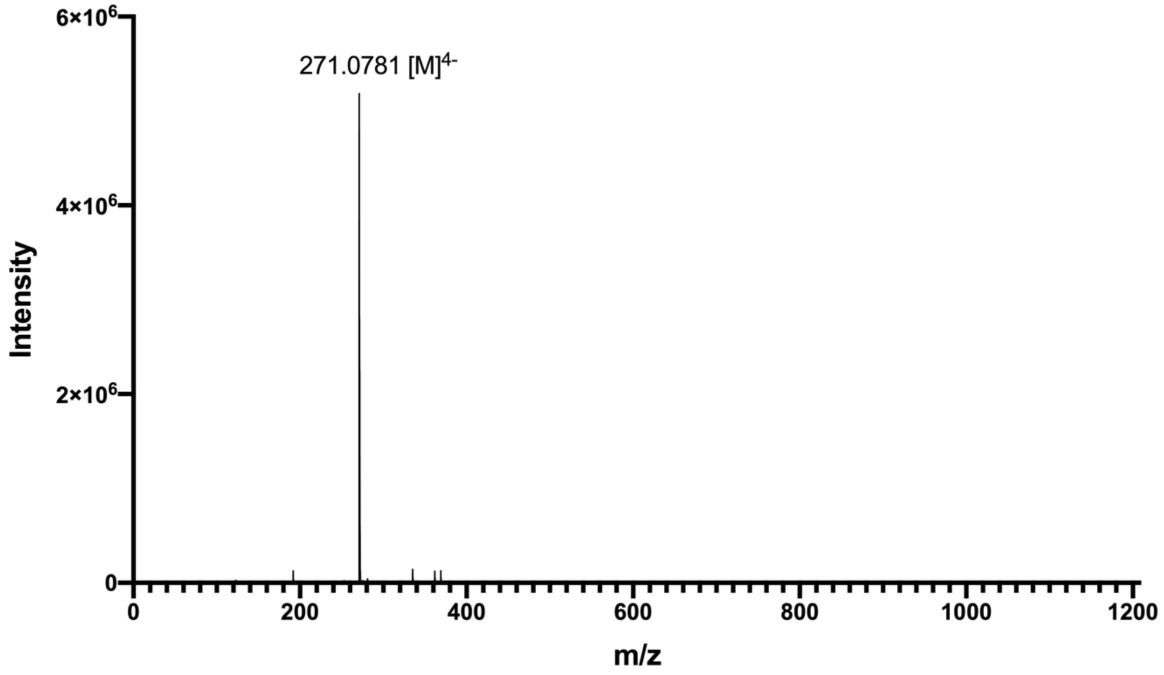

Figure S52. MS (nanochip-ESI/LTQ-orbitrap) of P4C9S

A3C9OH

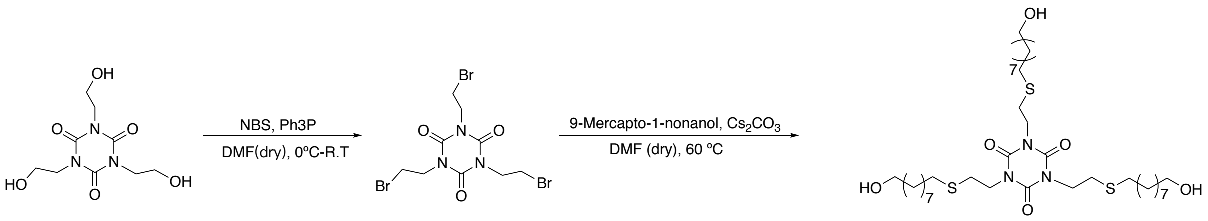

Tris(2-hydroxyethyl) isocyanurate (2g, 7.66mmol) and triphenylphosphine (6.2g, 23.7mmol, 3.1eq) were dissolved in 5mL of extra dry DMF. 4.2 g of dry DMF (5 mL) dissolved N-Bromosuccinimide (23.7 mmol, 3.1 eq) was added dropwise to aforementioned mixture with stirring at 0 °C under argon atmosphere. The reaction mixture was slowly warmed up to room temperature and stirred overnight. Thin-layer chromatography (DCM:MeOH =40:1, triol Rf= 0 with CAM stain, product Rf =0.8 with $KMnSO_4$ stain) was used to monitor the reaction. Once the reaction was complete, 50 mL of DCM was added to dilute the mixture, followed by washing with saturated 15 mL $NaHCO_3$ solution (15 mL), 15 mL H_2O (15 mL) and 15 mL saturated NaCl solution, respectively. The organic phase was collected and dried over anhydrous Na_2SO_4, and the solvent was removed by rotatory evaporation to obtain the crude product, which was further purified by flash chromatography (Biotage system, silica gel column, ethyl acetate:hexane, 0 to 5%) to obtain 1.388g tris(2-bromoethyl) isocyanurate, as a colorless oil, yield 40.2%. ^{1}H NMR (400 MHz, $CDCl_3$) δ 2.88 (s, 2H, N- CH_2), 2.80 (s, 2H, CH_2Br). HRMS (ESI/QTOF) m/z: $[M + H]^+$ Calcd for $C_6H_{13}Br_3N^+$ 335.8593; Found 335.8592. The subsequent reaction was immediately conducted because of the instability of the tribromide. Tris(2-bromoethyl) isocyanurate and 9-mercapto-1-nonanol were used to synthesize compound A3C9OH following the general method A. The product was a white powder, yield 48.2%. ^{1}H NMR (400 MHz, $CDCl_3$) δ 4.12 – 4.00 (m, 6H, N- CH_2), 3.61 (t, J = 6.6 Hz, 6H, CH_2OH), 2.79 – 2.69 (m, 6H, N-C-CH_2-S), 2.56 (t, J = 7.4 Hz, 6H, S-$\underline{CH_2}$-CH_2), 1.70 (s, 3H, OH), 1.63 – 1.20 (m, 42H, C-CH_2-C). ^{13}C NMR (101 MHz, $CDCl_3$) δ 148.72, 62.95, 42.01, 32.75, 31.92, 29.44, 29.34, 29.21, 29.12, 28.76, 25.72. HRMS (nanochip-ESI/LTQ-Orbitrap) m/z: $[M + Na]^+$ Calcd for $C_{36}H_{69}N_3NaO_6S_3^+$ 758.4241; Found 758.4261.

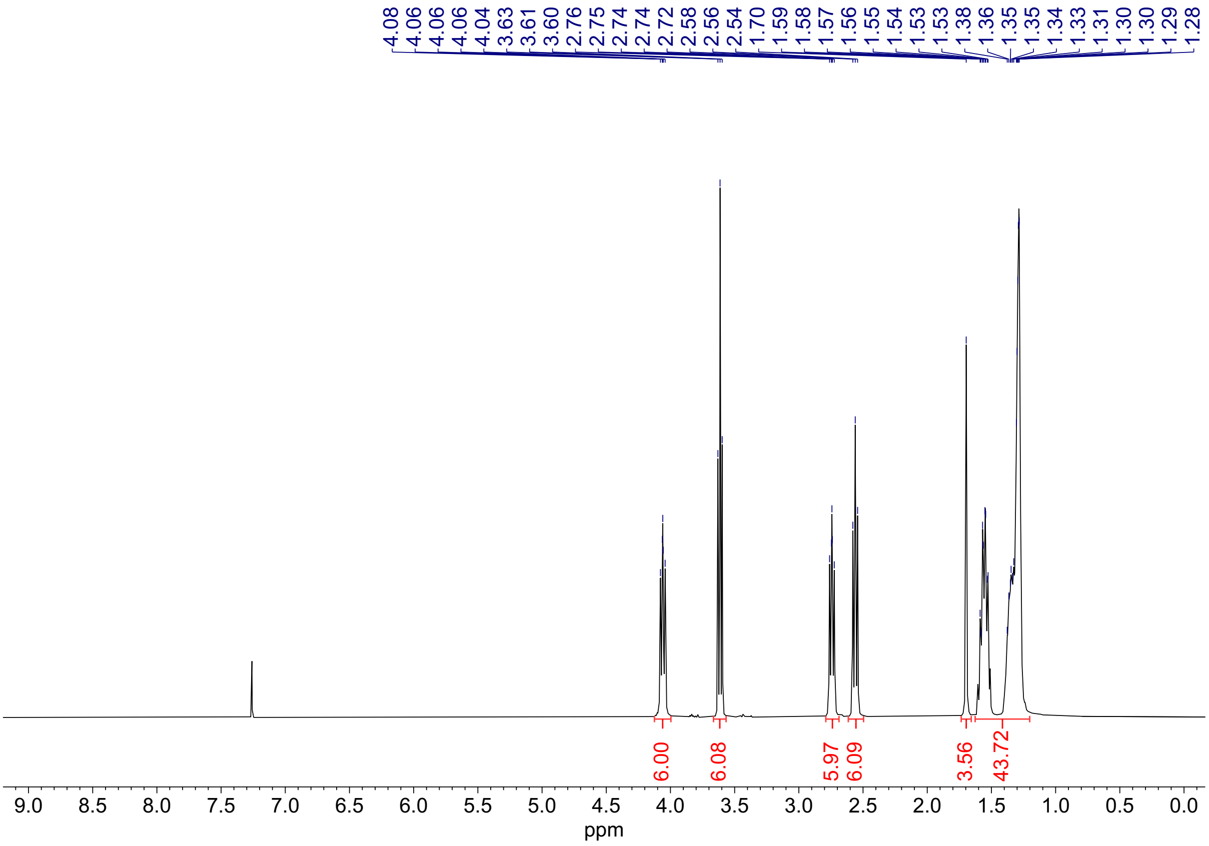

Figure S53. ^{1}H-NMR ($CDCl_3$, 400 MHz) of A3C9OH

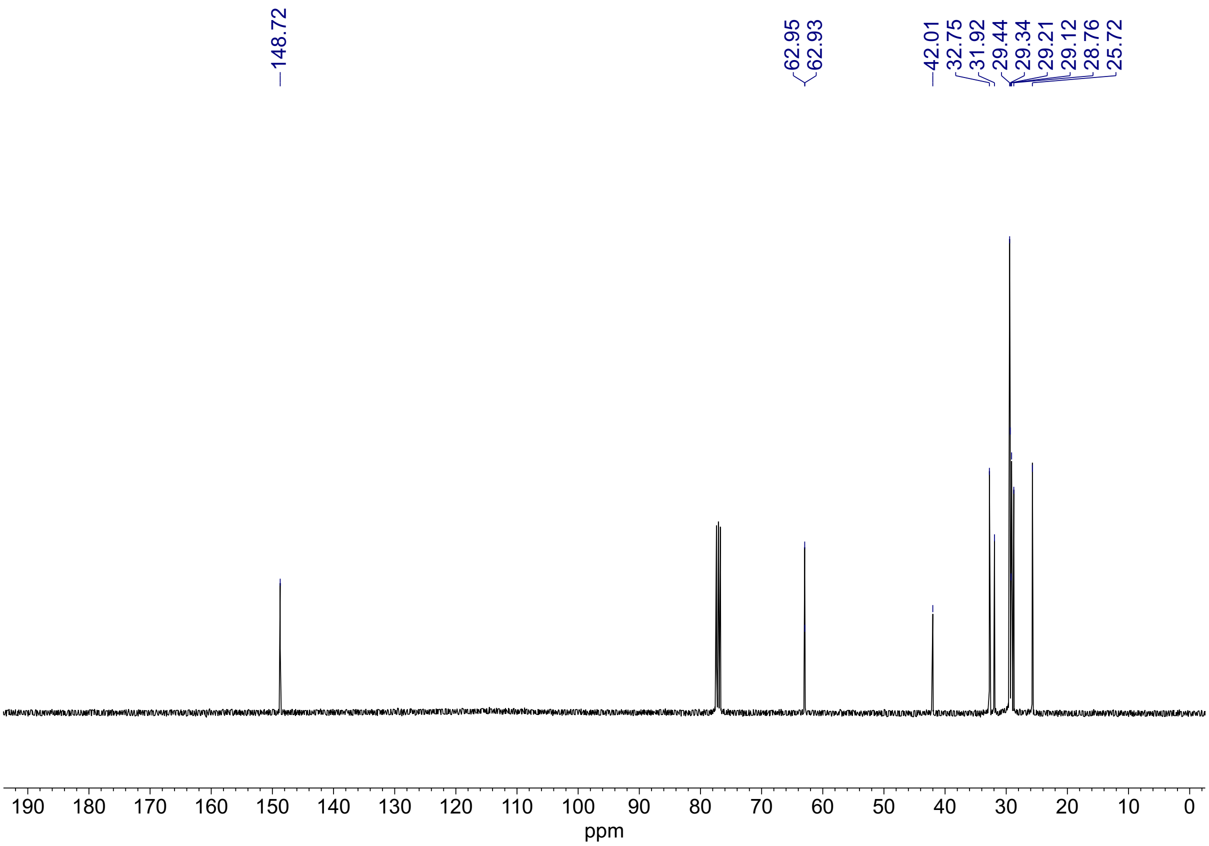

Figure S54. ^{13}C-NMR ($CDCl_3$, 101 MHz) of A3C9OH

A3C9S

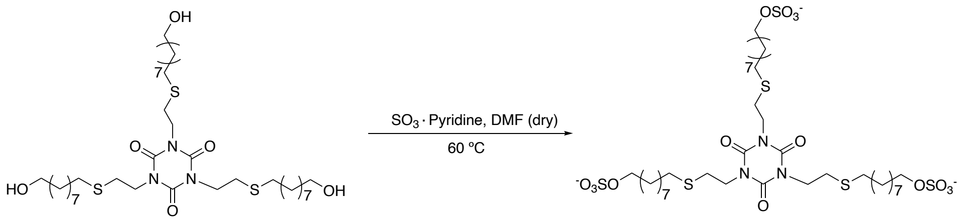

From compound A3C9OH following the general method B gave compound A3C9S as a white powder, yield 40.4%. ^{1}H NMR (400 MHz, D_2O) δ 4.07 (t, *J* = 6.7 Hz, 12H, N- CH_2, CH_2OSO3^-), 2.80 (t, *J* = 6.9 Hz, 6H, N-C-CH_2-S), 2.63 (t, *J* = 7.3 Hz, 6H, S-$\underline{CH_2}$-CH_2), 1.78 – 1.28 (m, 48H, C-CH_2-C). ^{13}C NMR (101 MHz, D_2O) δ 149.09, 69.36, 41.89, 31.68, 29.44, 29.41, 29.24, 29.20, 28.96, 28.93, 28.84, 25.47. HRMS (nanochip-ESI/LTQ-Orbitrap) m/z: $[M]^{3-}$ Calcd for $C_{36}H_{66}N_3O_{15}S_6^{3-}$ 324.0939; Found 324.0910.

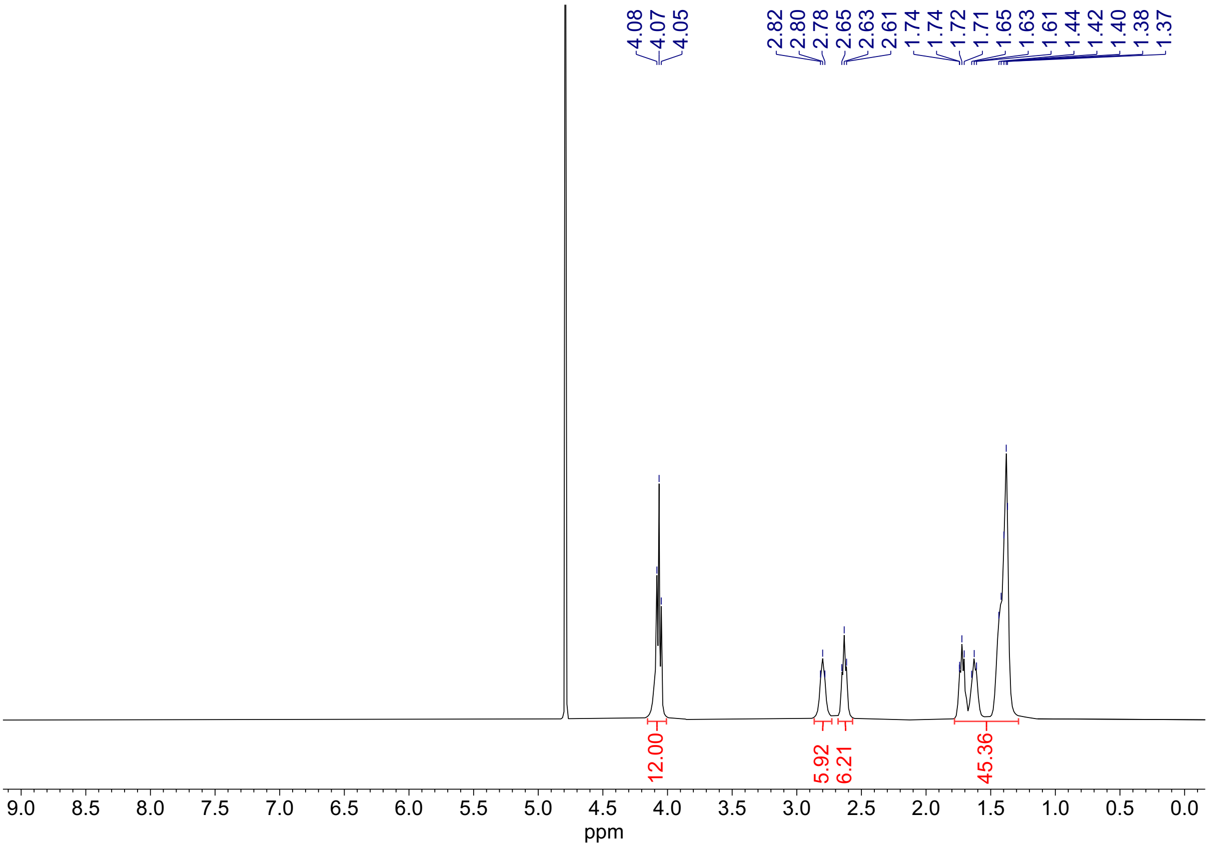

Figure S55. ^{1}H-NMR (D_2O, 400 MHz) of A3C9S

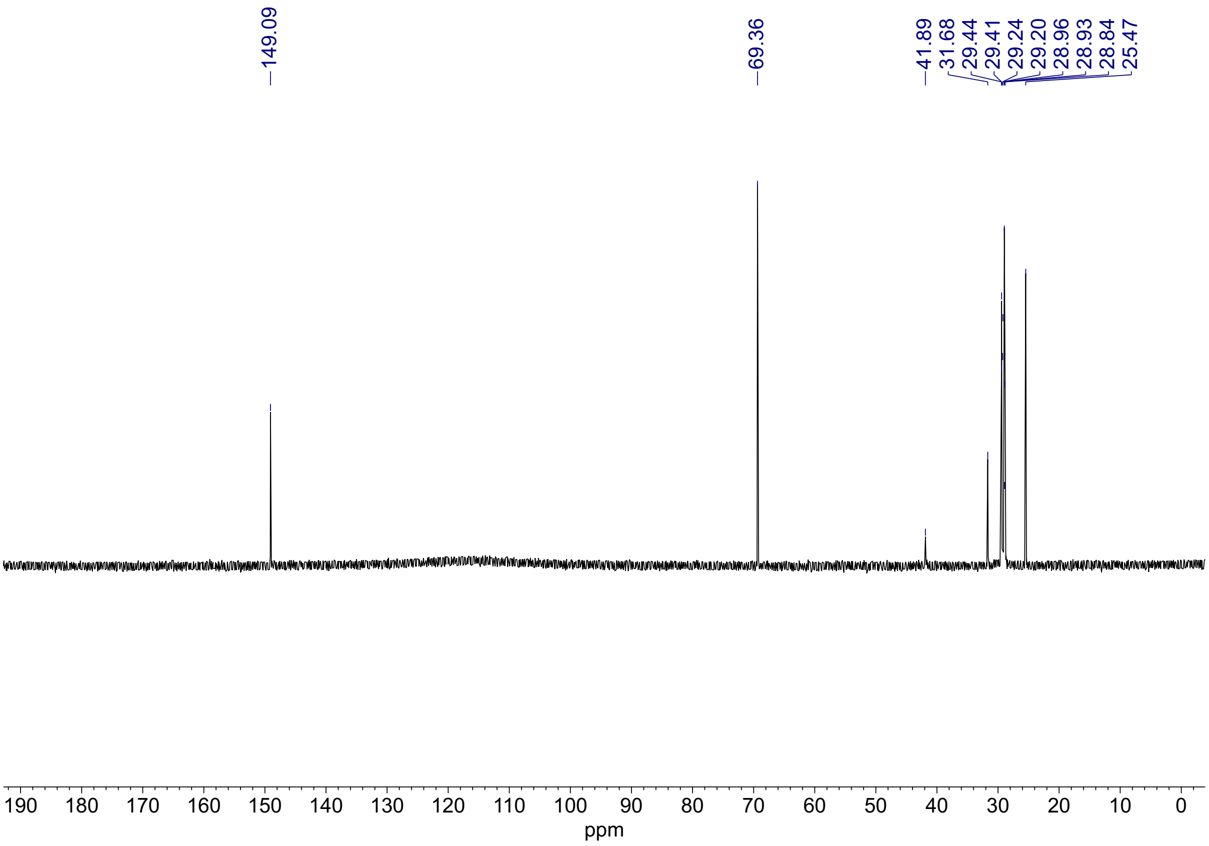

Figure S56. ^{13}C-NMR (D_2O, 101 MHz) of A3C9S

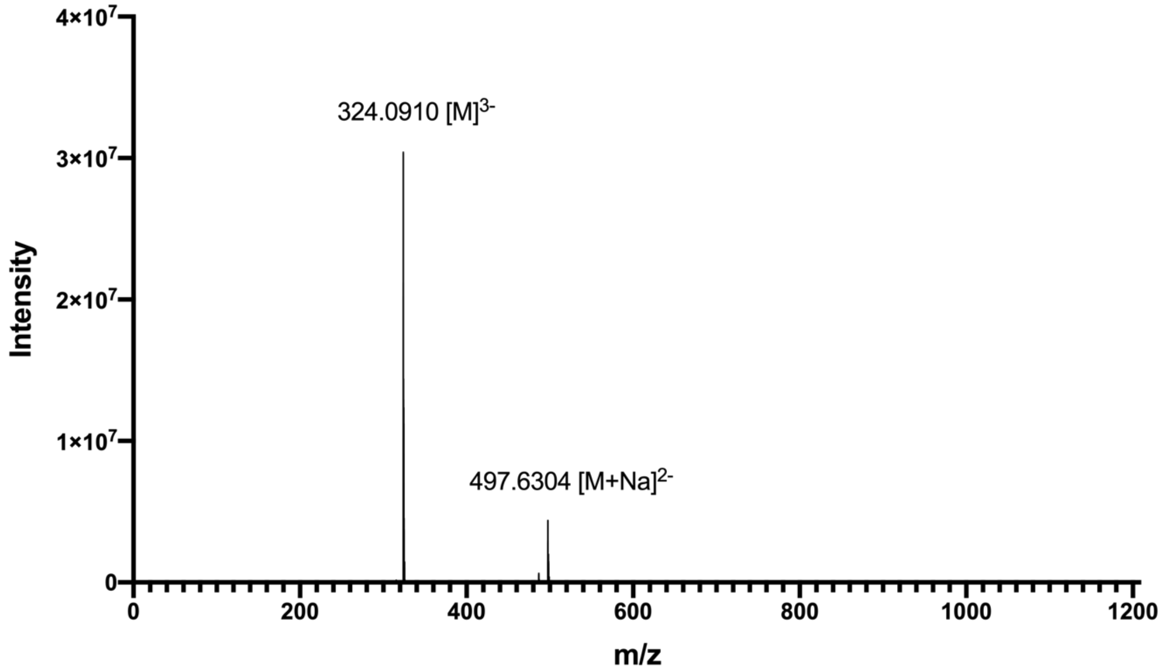

Figure S57. MS (nanochip-ESI/LTQ-orbitrap) of A3C9S

P3C9OH

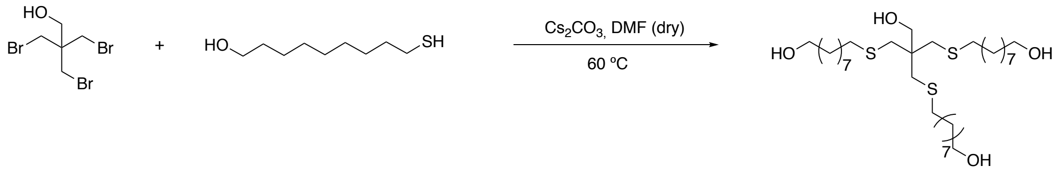

3-Bromo-2,2-bis(bromomethyl)propanol and 9-mercapto-1-nonanol were used to synthesize compound P3C9OH following the general method A. The product was a white powder, yield 56.0%. ^{1}H NMR (400 MHz, $CDCl_3$) δ 3.60 – 3.48 (m, 8H, CH_2-O), 2.64 (m, 10H, OH, C- CH_2-S), 2.49 (t, J = 7.4 Hz, 6H, S- $\underline{CH_2}$- CH_2), 1.51– 1.13 (m, 42H, CH_2- $\underline{CH_2}$- CH_2). ^{13}C NMR (101 MHz, $CDCl_3$) δ 65.42, 62.72, 44.48, 36.99, 33.92, 32.65, 29.82, 29.45, 29.35, 29.13, 28.78, 25.72. HRMS (nanochip-ESI/LTQ-Orbitrap) m/z: [M + Na]$^{+}$ Calcd for $C_{32}H_{66}NaO_4S_3^{+}$ 633.4021; Found 633.4005.

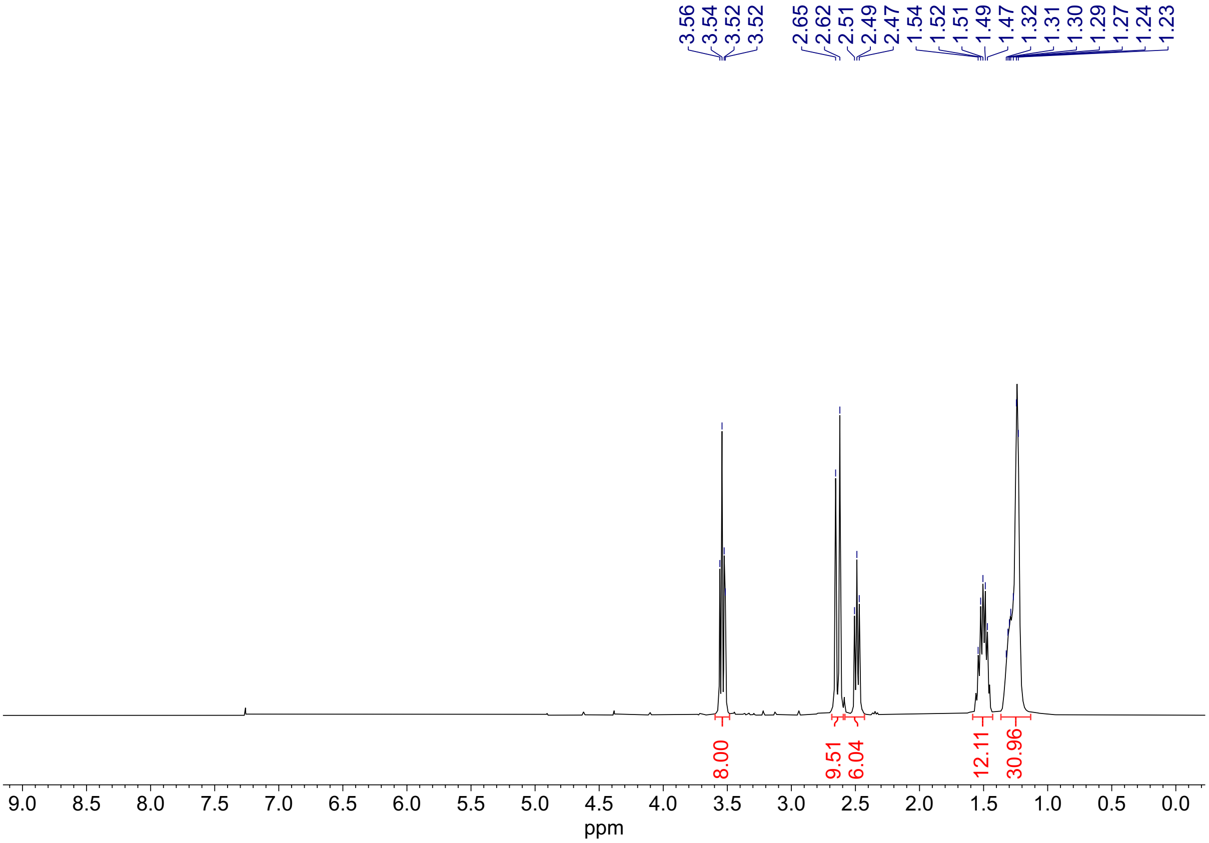

Figure S58. ^{1}H-NMR ($CDCl_3$, 400 MHz) of P3C9OH

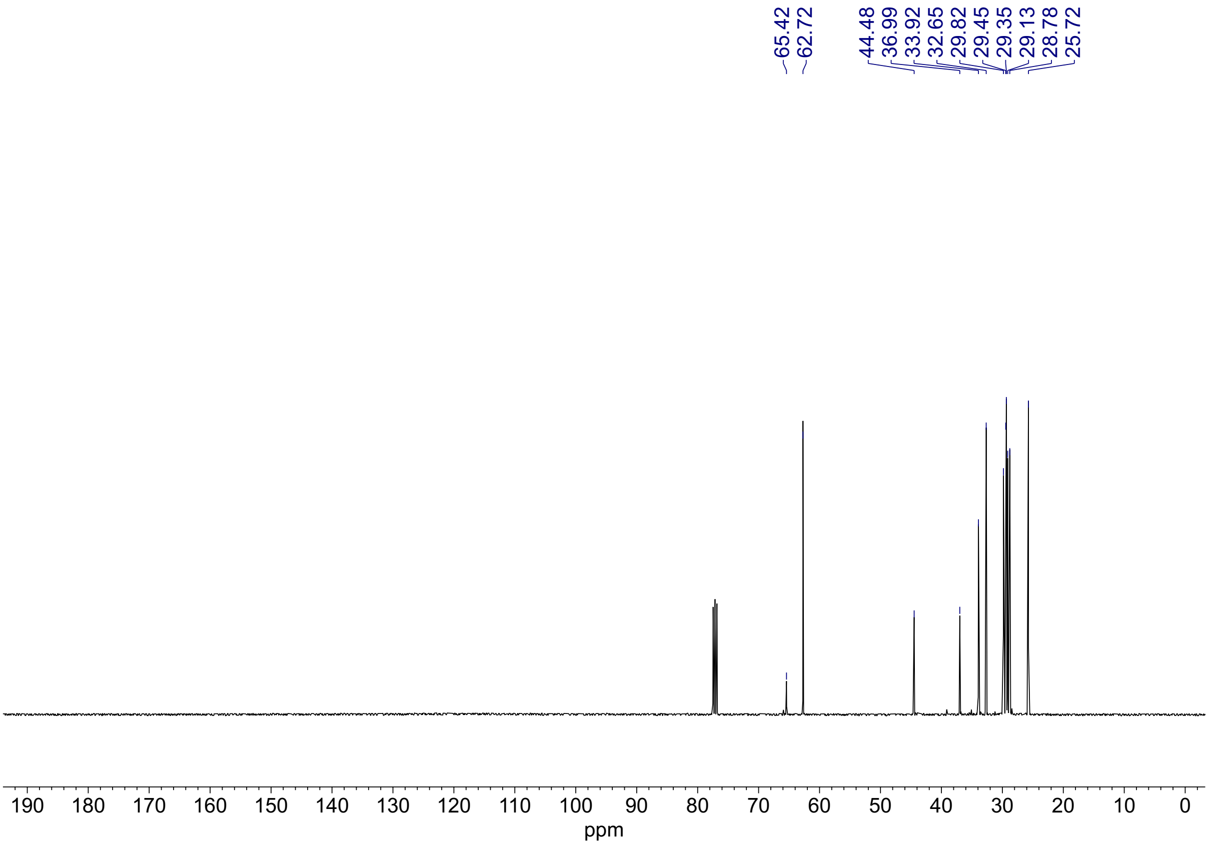

Figure S59. ^{13}C-NMR ($CDCl_3$, 101 MHz) of P3C9OH

P3C9S

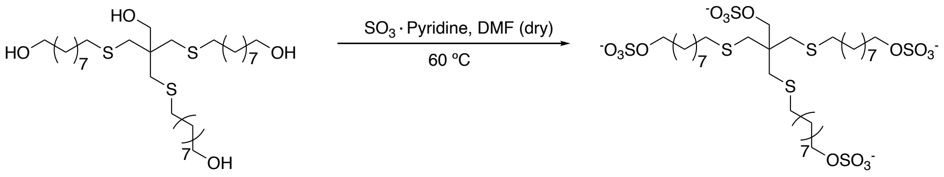

From compound P3C9OH following the general method B gave compound P3C9S as a white powder, yield 70.1%. ^{1}H NMR (400 MHz, D_2O) δ 4.14 (t, *J* = 6.7 Hz, 6H, CH_2-$\underline{CH_2}$-OSO_3^-), 4.06 (s, 2H, C-CH_2-OSO_3^-), 2.83 (s, 6H, C-CH_2-S), 2.74 (t, *J* = 7.3 Hz, 6H, S-$\underline{CH_2}$-CH_2), 1.86 – 1.34 (m, 42H, C-CH_2-C). ^{13}C NMR (101 MHz, D_2O) δ 69.55, 43.79, 36.00, 33.81, 29.86, 29.44, 29.24, 29.21, 28.99, 28.78, 25.50. HRMS (nanochip-ESI/LTQ-Orbitrap) m/z: $[M]^{4-}$ Calcd for $C_{32}H_{62}N_3O_{16}S_7^{4-}$ 231.5521; Found 231.5499.

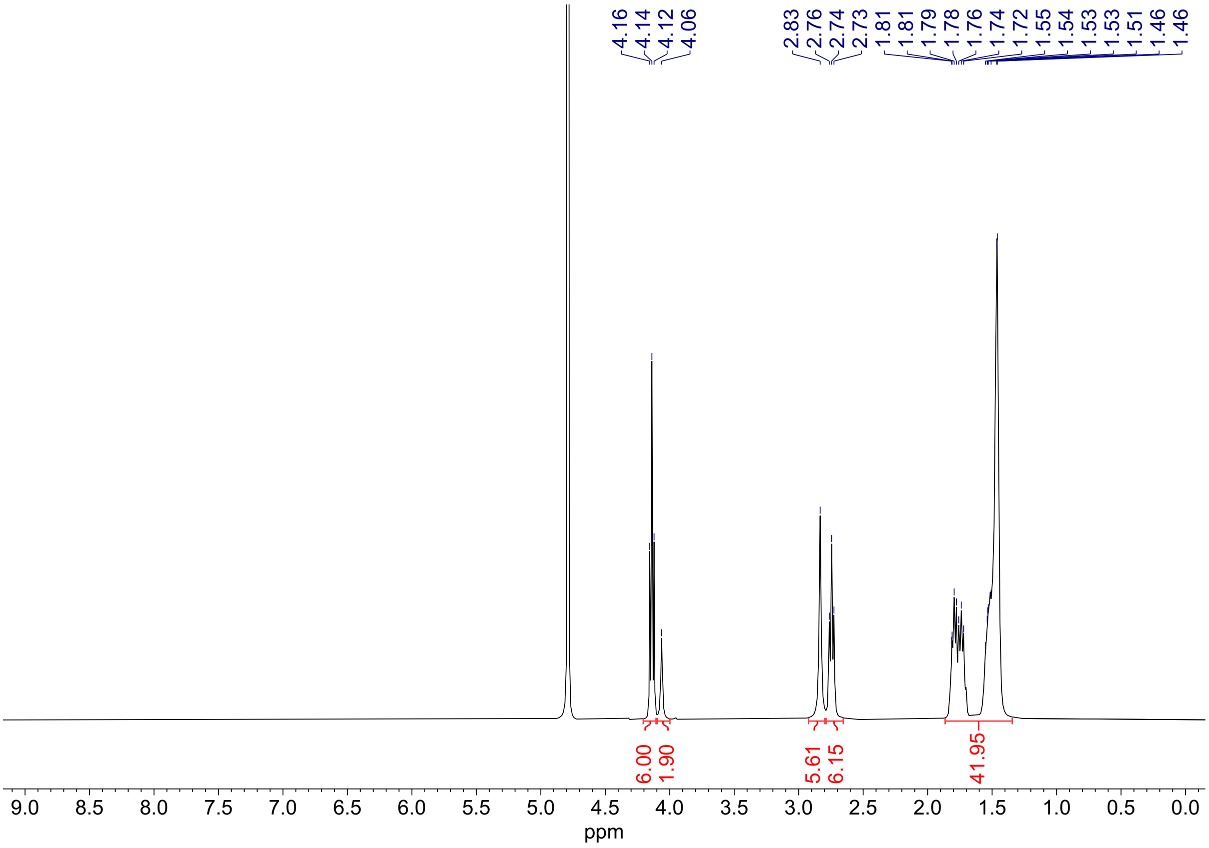

Figure S60. ^{1}H-NMR (D_2O, 400 MHz) of P3C9S

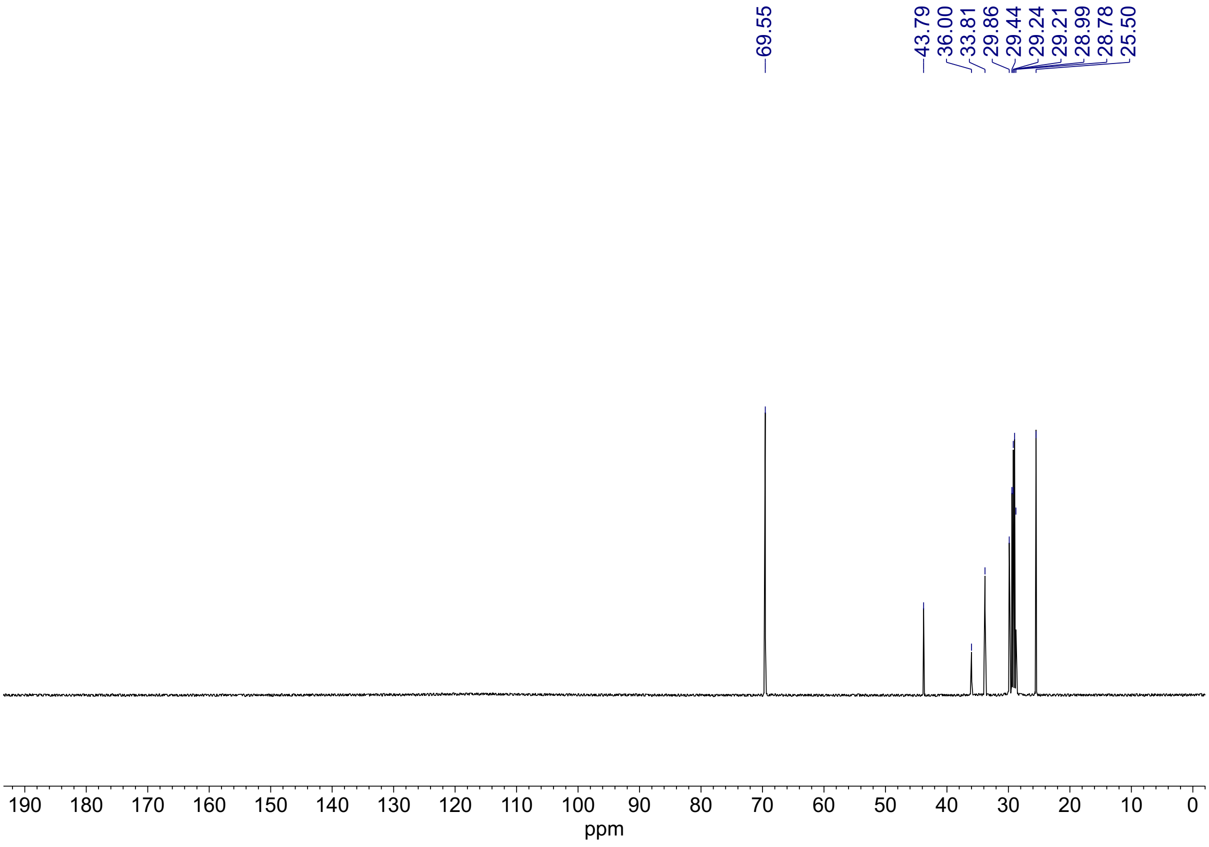

Figure S61. ^{13}C-NMR (D_2O, 101 MHz) of P3C9S

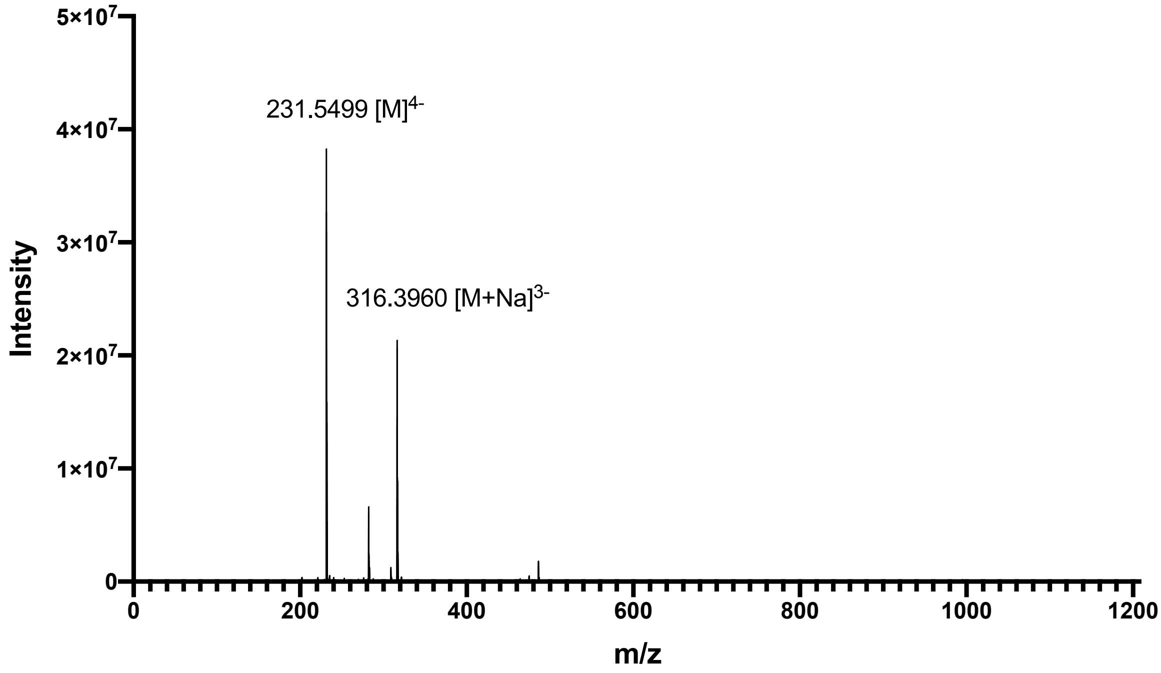

Figure S62. MS (nanochip-ESI/LTQ-orbitrap) of P3C9S

P2C9OH

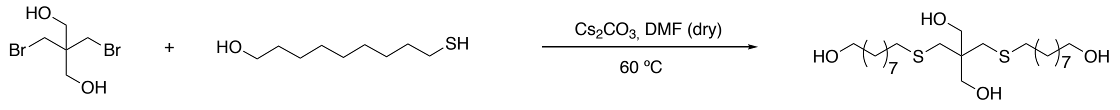

2,2-Bis(bromomethyl)-1,3-propanediol and 9-mercapto-1-nonanol were used to synthesize compound P2C9OH following the general method A. The product was a white powder, yield 59.8%. ^{1}H NMR (400 MHz, CD_3OD_SPE) δ 3.53 (m, 8H, CH_2-OH), 2.63 (s, *J* = 2.3 Hz, 4H, S-CH_2-C), 2.55 (t, *J* = 7.4 Hz, 4H, S-CH_2-CH_2), 1.67 – 1.17 (m, 28H, CH_2-CH_2-CH_2). ^{13}C NMR (101 MHz, CD_3OD) δ 77.65, 63.35, 61.71, 44.85, 34.24, 33.59, 32.36, 29.74, 29.40, 29.29, 29.06, 28.62, 25.65. HRMS (nanochip-ESI/LTQ-Orbitrap) m/z: $[M + Na]^+$ Calcd for $C_{23}H_{48}NaO_4S_2^+$ 475.2886; Found 475.2896.

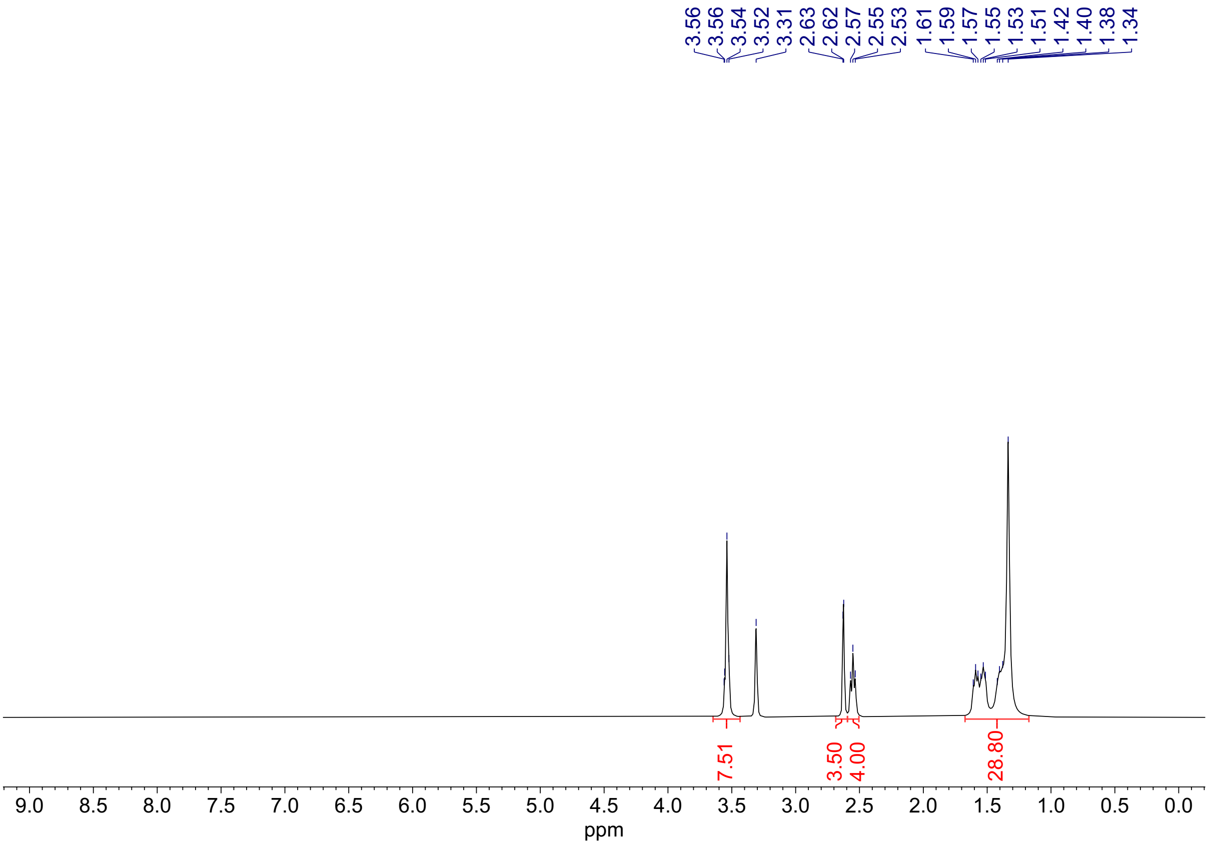

Figure S63. ^{1}H-NMR ($CDCl_3$, 400 MHz) of P2C9OH

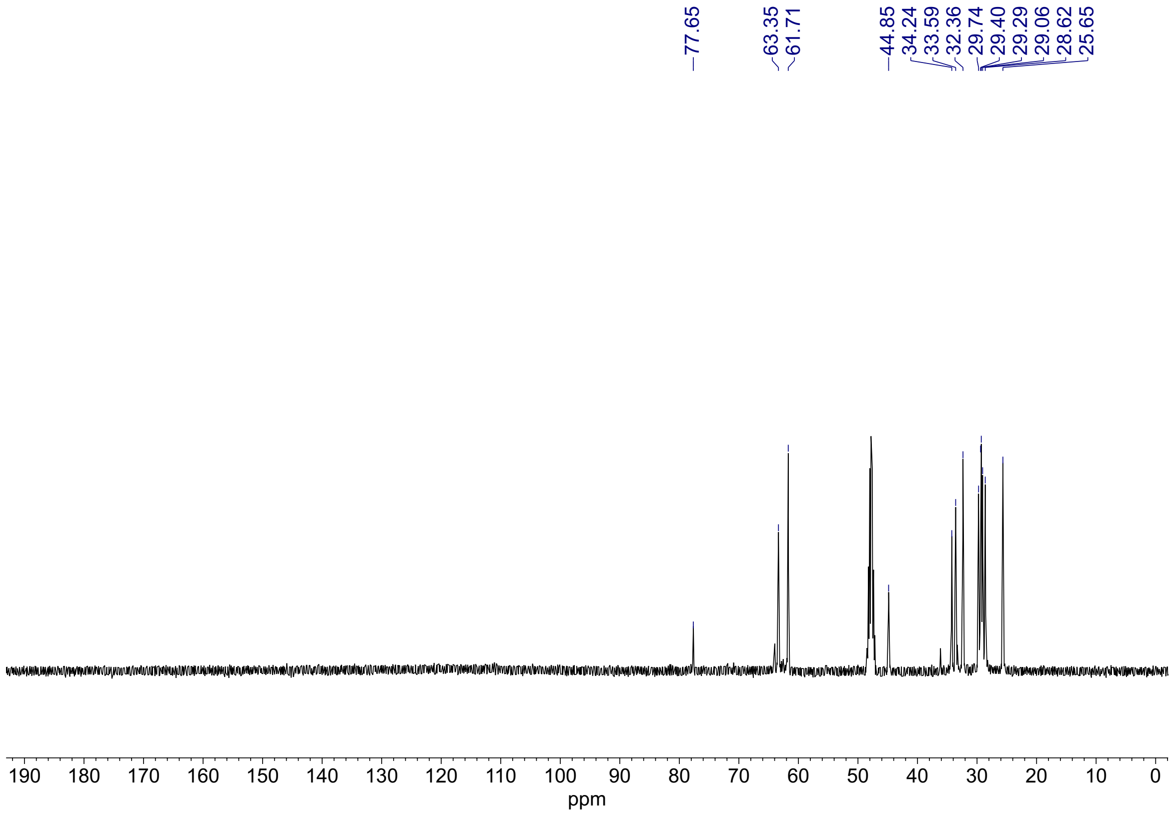

Figure S64. ^{13}C-NMR ($CDCl_3$, 101 MHz) of P2C9OH

P2C9S

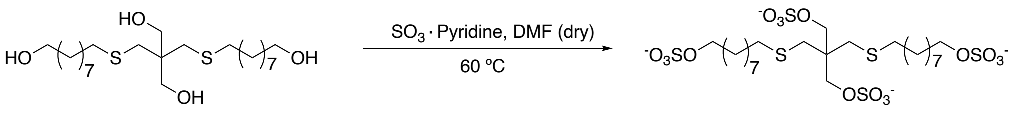

From compound P2C9OH following the general method B gave compound P2C9S as a white powder, yield 69.4%. ^{1}H NMR (400 MHz, D_2O) δ 4.10 (t, *J* = 6.6 Hz, 4H, CH_2-<u>CH_2</u>-OSO_3^-), 4.05 (s, 4H, C-CH_2-OSO_3^-), 2.78 (s, 4H, C-CH_2-S), 2.70 (t, *J* = 7.3 Hz, 4H, S-<u>CH_2</u>-CH_2), 1.79 – 1.30 (m, 28H, C-CH_2-C). ^{13}C NMR (101 MHz, D_2O) δ 69.71, 68.23, 43.23, 33.48, 33.25, 29.10, 28.73, 28.59, 28.48, 28.46, 28.04, 25.02. HRMS (nanochip-ESI/LTQ-Orbitrap) m/z: $[M]^{4-}$ Calcd for $C_{23}H_{44}O_{16}S_6^{4-}$ 192.0239; Found 192.0222.

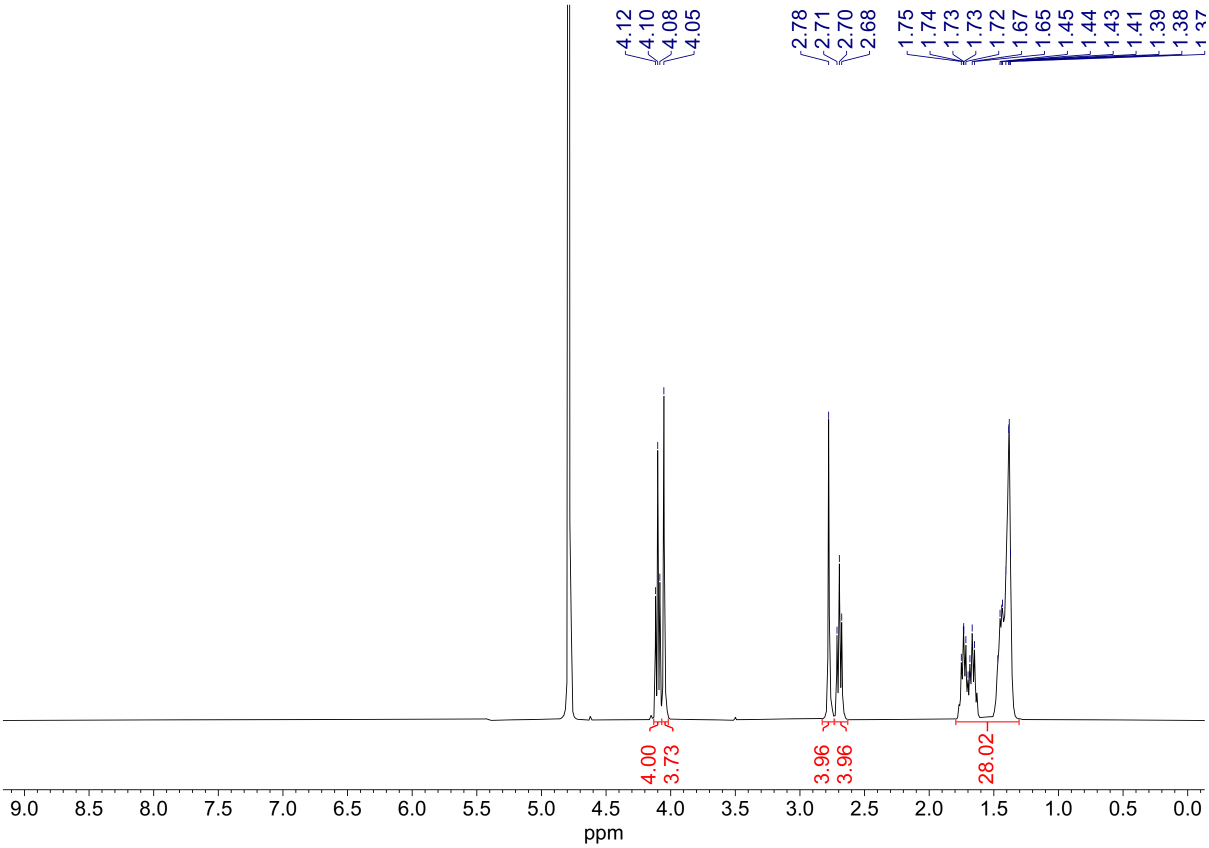

Figure S65. 1H-NMR (D2O, 400 MHz) of P2C9

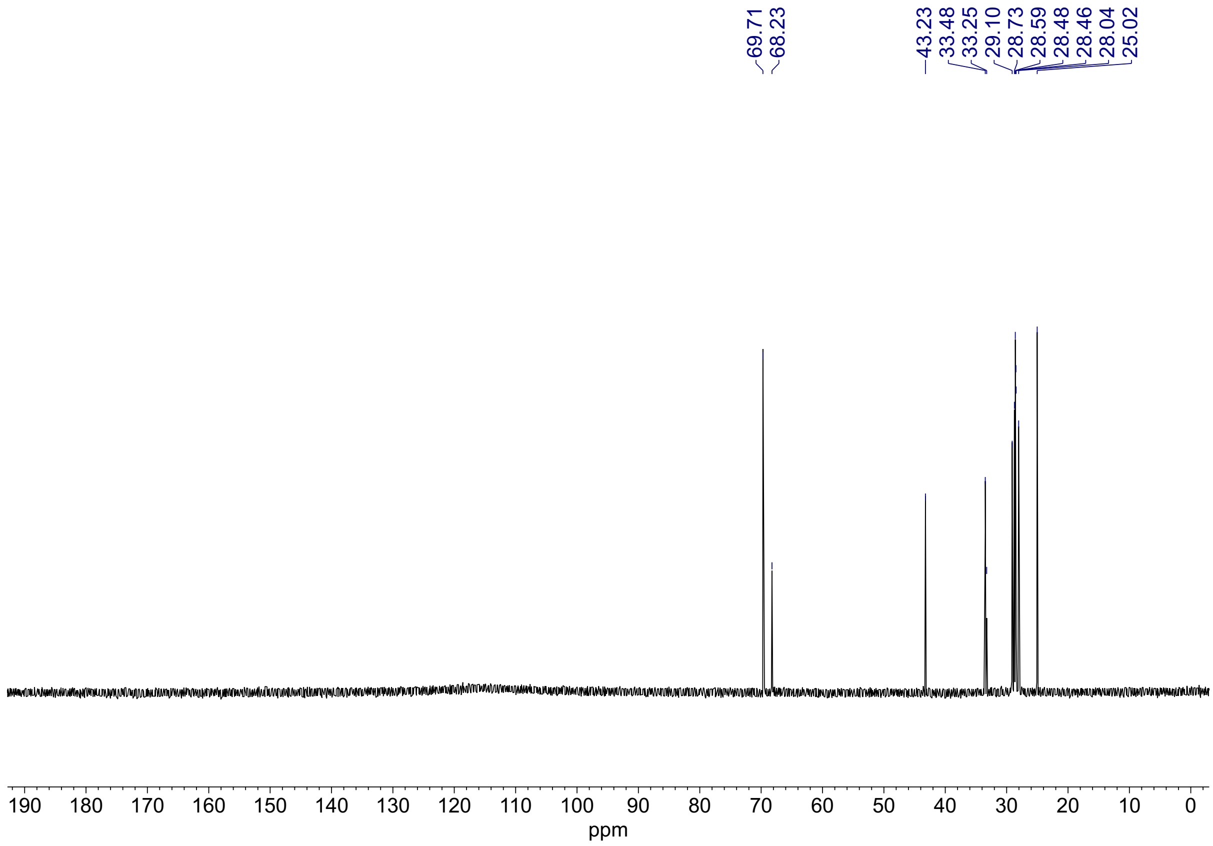

Figure S66. ^{13}C-NMR (D_2O, 101 MHz) of P2C9S

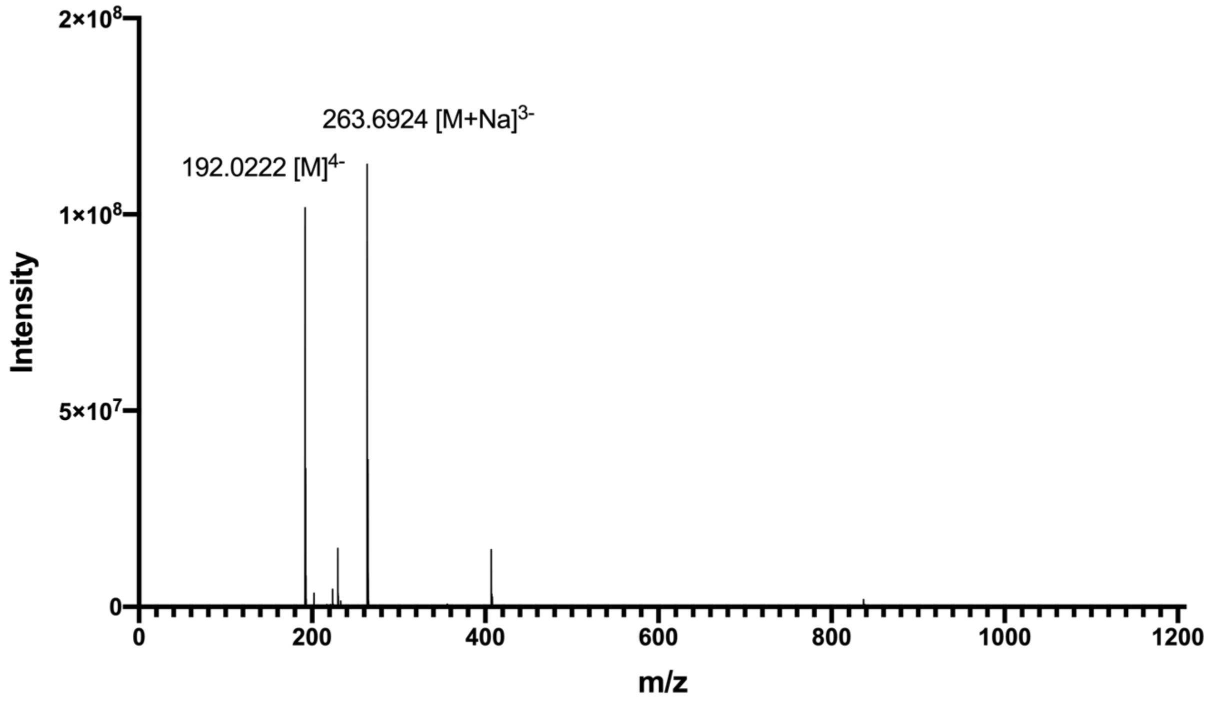

Figure S67. MS (nanochip-ESI/LTQ-orbitrap) of P2C9S

I3C10S

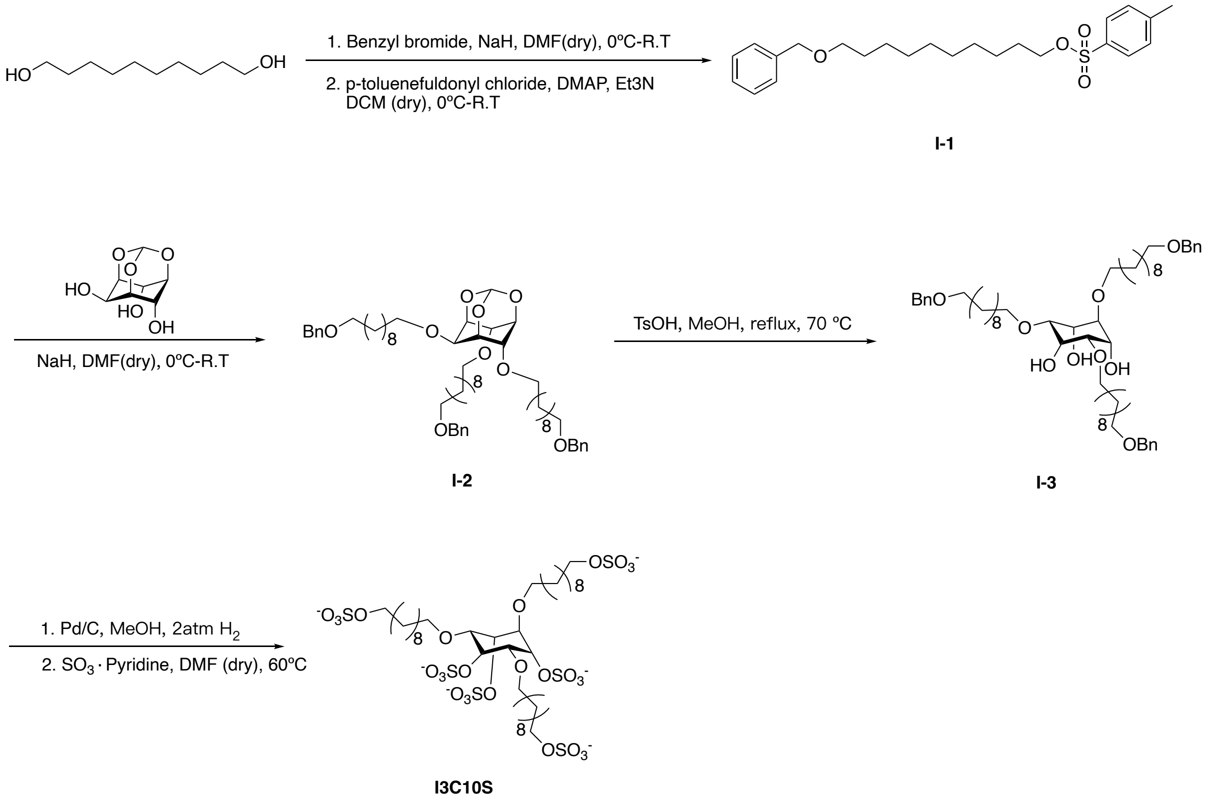

I-1 was synthesized following a procedure described in the reference[2]. Briefly, 1,10-decanediol (17.4 g, 100 mmol) and sodium hydride (6 g, 150 mmol, 1.5 eq) were dissolved in 20 mL of extra dry DMF and stirred at 0 °C for 30 min under argon atmosphere, followed by a dropwise addition of 14.3 mL of benzyl bromide (120 mmol, 1.2 eq). The reaction mixture was then slowly warmed to room temperature and stirred for another 30 min. 10 mL of distilled water was added to quench the reaction and 150 mL ethyl acetate was used in total to extract the product (3×50 mL). The organic phase was then dried over Na_2SO_4, concentrated and silica gel column chromatography (hexane: ethyl acetate, 5:1) purification was used to obtain 21.7 g of a colorless oil, 10-(phenylmethoxy)-1-decanol (yield, 82.3%). 10-(phenylmethoxy)-1-decanol (3 g, 11.4 mmol), 4-dimethylaminopyridine (293 mg, 2.27 mmol, 0.2 eq) and 2 mL triethylamine (11.9 mmol, 1.04 eq) were dissolved in 24 mL extra dry dichloromethane at 0 °C. 2.5 g of *p*-toluenesulfonyl chloride (12.77 mmol, 1.12 eq) was then added and the reaction was warmed to room temperature and stirred overnight. A saturated $NaHCO_3$ solution (15 mL) was added to quench the reaction, and the mixture was extracted three times with 30 mL of ethyl acetate. After drying the organic phase over Na_2SO_4 and filtration, the filtrate was concentrated and the crude product was purified by silica gel column (hexane: ethyl acetate= 5:1, Rf=0.5) to obtain 1.617 g of light-yellow oil, **I-1** (yield, 33.3%).

1,3,5-O-methylidyne-*myo*-Inositol (73.5 mg, 0.387 mmol) and sodium hydride (140 mg, 1.741 mmol, 9 eq.) was dissolved in extra dry DMF (4 mL) under Argon. The mixture was cooled down to 0 °C and stirred for 30 min. **I-1** (809 mg, 1.741 mmol, 4.5 eq., dissolved in 2mL of dry DMF) was added dropwisely to the mixture, and the reaction was warmed to room temperature and stirred for 2 days. 1 mL of distilled H_2O was added to quench the reaction and crude product was extracted with ethyl acetate and purified with silica gel column (Hex/EtOAc gradient, Rf= 0.8 with Hex: EtOAc 5:1). 255.2 mg of **I-2** was obtained as a white solid, yield 71.1%. ^1H NMR (400 MHz, $CDCl_3$) δ 7.46 – 7.23 (m, 15H, Ph-H), 5.58 (s, 1H, $CH-(OR)_3$),

4.53 (s, 6H, Ph-CH_2), 4.48 – 4.37 (m, 1H, inositol-H), 4.35 (dt, *J* = 3.7, 1.7 Hz, 2H, inositol-H), 4.24 (t, *J* = 3.6 Hz, 2H, inositol-H), 3.83 (q, *J* = 1.7 Hz, 1H, inositol-H), 3.69 – 3.45 (m, 12H, inositol-O-CH_2-), 1.79 – 1.19 (m, 48H, C-CH_2-C, CH_2-O-Bn). ^{13}C NMR (101 MHz, $CDCl_3$) δ 138.80, 128.35, 127.59, 127.58, 127.46, 127.45, 103.34, 74.60, 72.87, 72.87, 70.59, 70.52, 70.51, 69.79, 68.35, 68.13, 29.96, 29.93, 29.88, 29.86, 29.67, 29.61, 29.58, 29.55, 29.54, 26.31, 26.28, 26.18, 26.14.

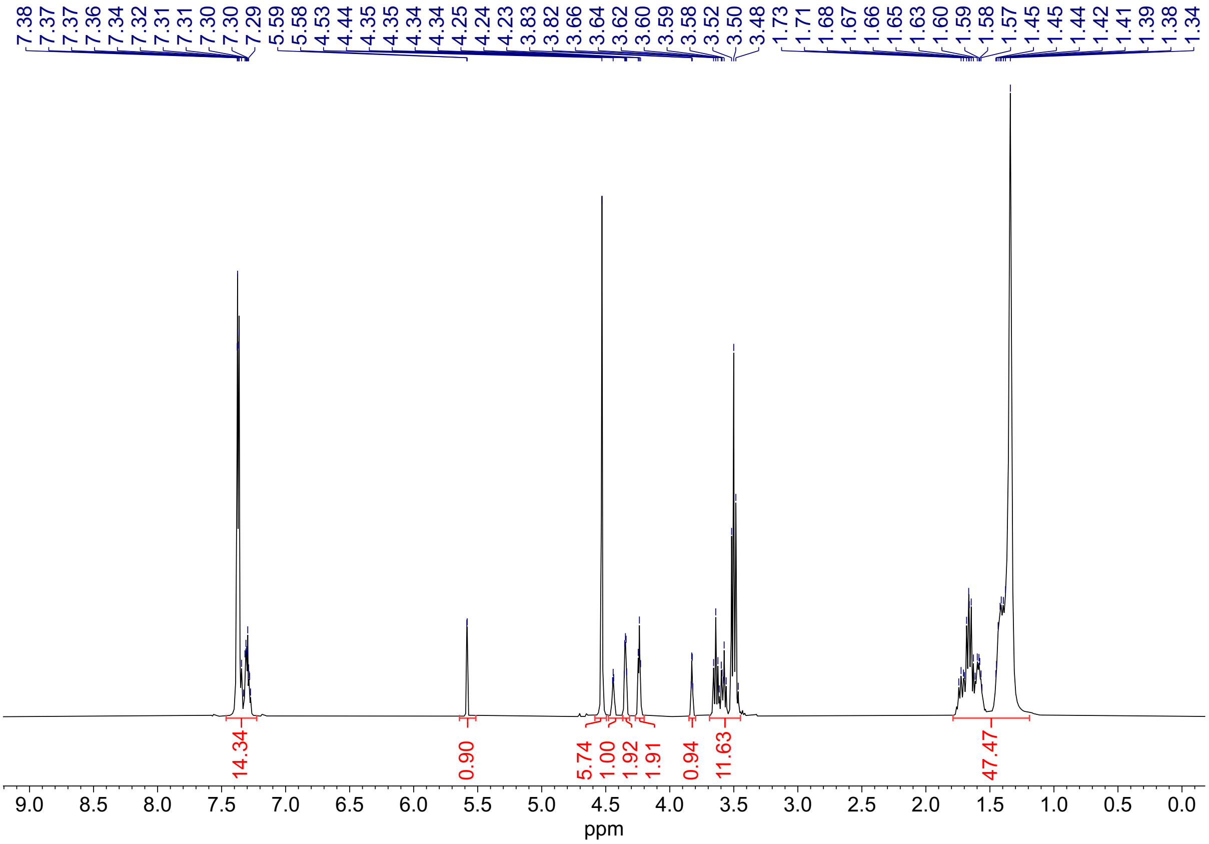

Figure S68. ^{1}H-NMR ($CDCl_3$, 400 MHz) of I-2

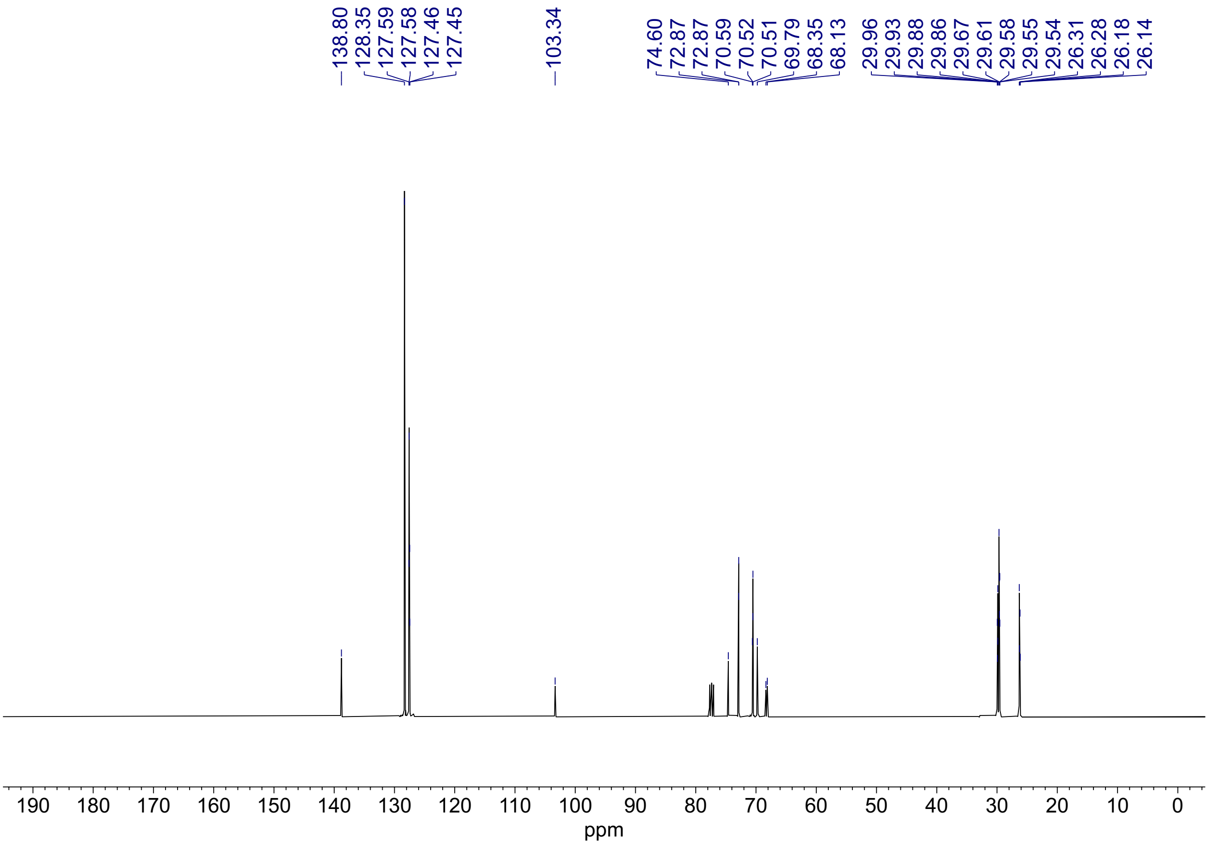

Figure S69. ^{13}C-NMR ($CDCl_3$, 101 MHz) of I-2

I-2 (255.2 mg, 0.275 mmol) and p-Toluenesulfonic acid (2.682 mg, 0.0141mmol, 0.05 eq.) were dissolved in methanol (8mL). After refluxing at 70 °C for 8 h, 3.7 mL of saturated $NaHCO_3$ and 5.5 mL of distilled water were added to quench the reaction. The reaction mixture was extracted with diethyl ether, and silica gel column chromatography was used to purify the product (hexane: ethyl acetate 1:1, Rf= 0.5). 162.4 mg of **I-3** was finally collected as a white solid (yield, 64.2%). ^{1}H NMR (400 MHz, $CDCl_3$) δ 7.45 – 7.26 (m, 15H, Ph-H), 4.53 (s, 6H, Ph-CH_2), 3.86 – 3.79 (m, 7H, inositol-H, inositol-O-CH_a), 3.53 – 3.38 (m, 11H, Inositol-O-CH_2), 2.50 (s, 3H, OH), 1.73 – 1.56 (m, 12H, CH_2-OBn), 1.47 – 1.11 (m, 36H, C-CH_2-C). ^{13}C NMR (101 MHz, $CDCl_3$) δ 138.75, 128.35, 127.63, 127.48, 82.03, 79.66, 74.68, 73.93, 73.19, 72.87, 72.54, 70.53, 30.44, 30.38, 29.82, 29.80, 29.61, 29.59, 29.56, 29.52, 29.49, 29.48, 26.23, 26.22, 26.14, 26.13. HRMS (ESI/QTOF) m/z: $[M + H]^+$ Calcd for $C_{58}H_{89}O_9^+$ 929.6501; Found 929.6501.

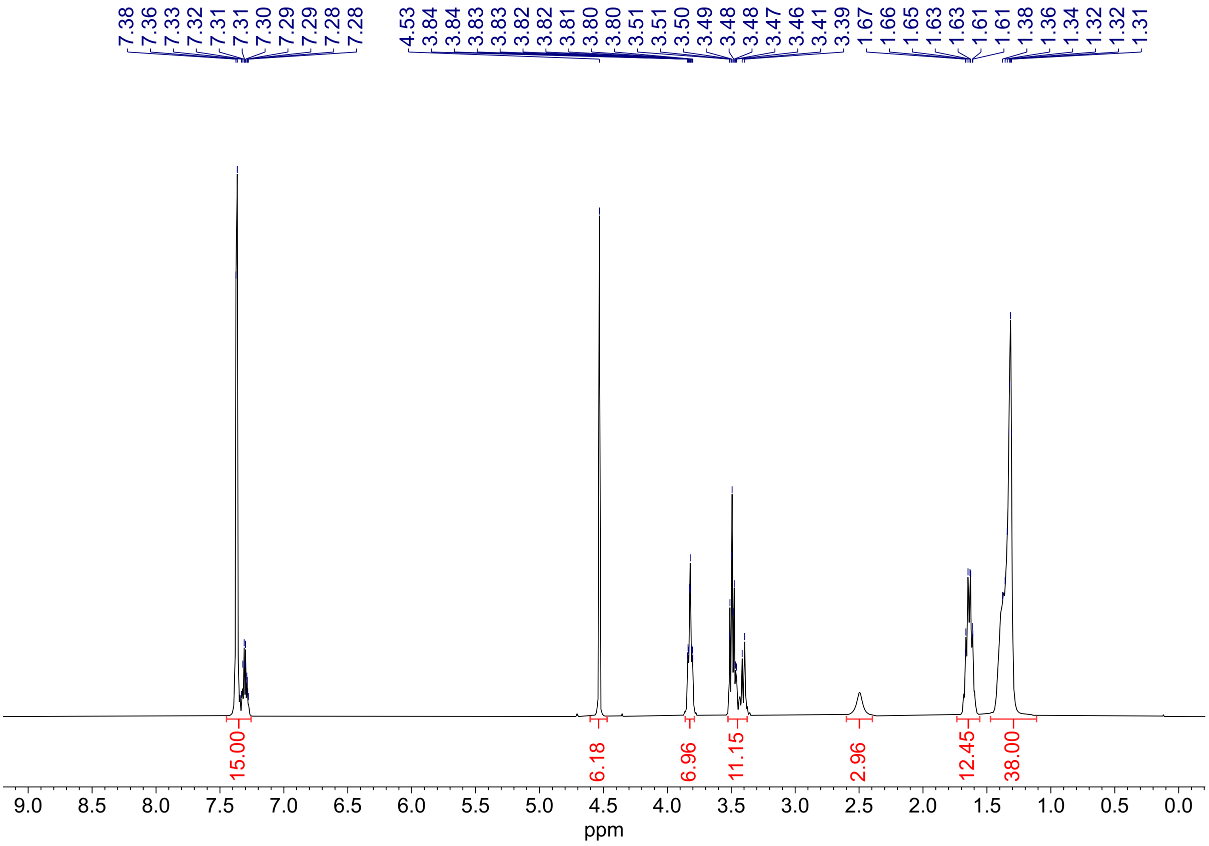

Figure S70. ^{1}H-NMR ($CDCl_3$, 400 MHz) of I-3

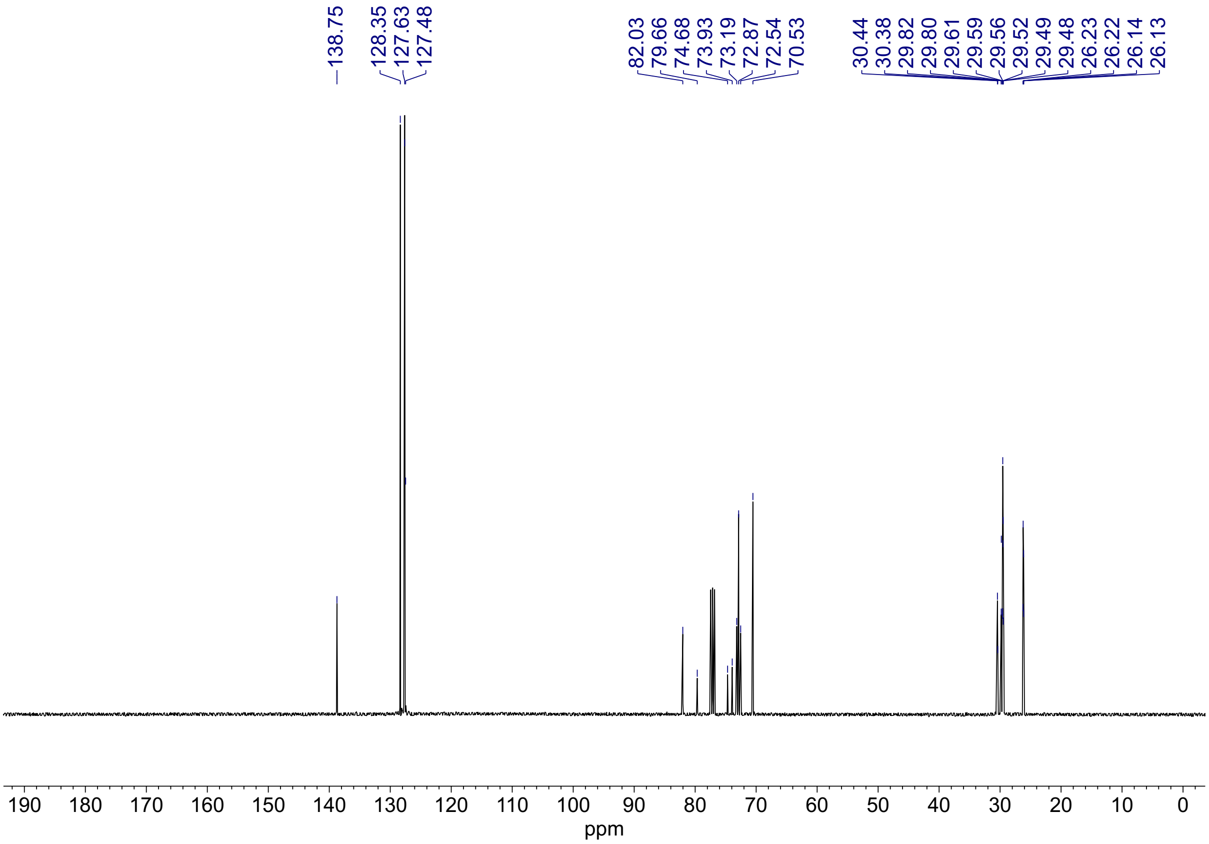

Figure S71. ^{13}C-NMR ($CDCl_3$, 101 MHz) of I-3

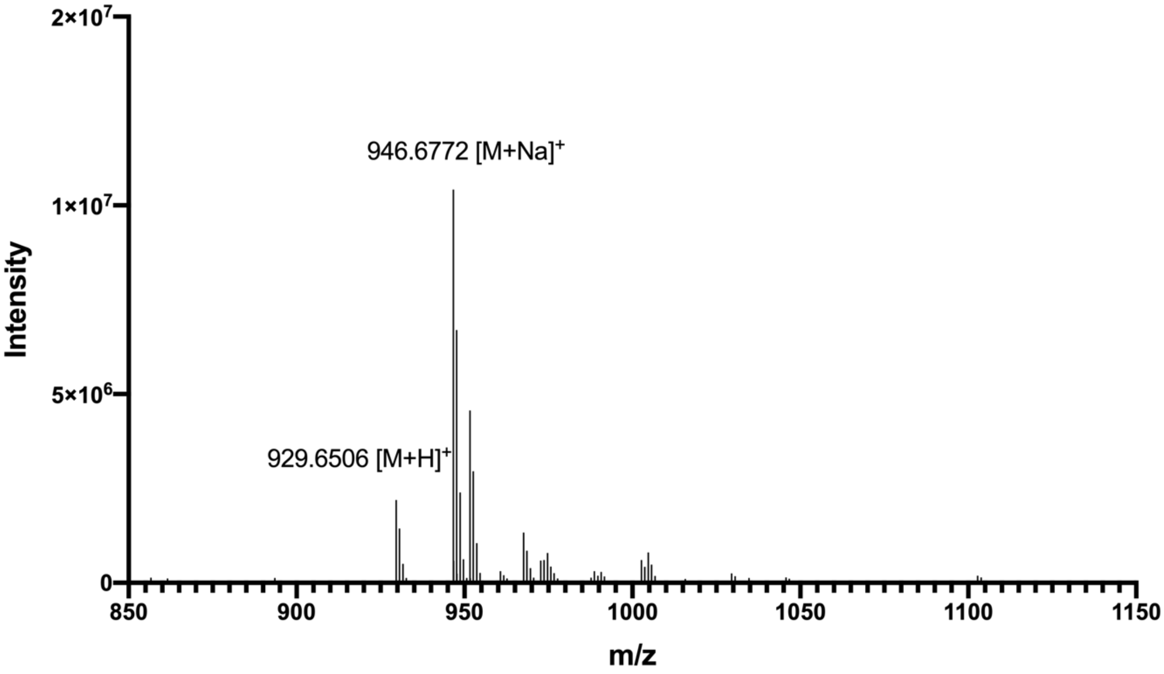

Figure S72. MS (nanochip-ESI/LTQ-orbitrap) of I-3

162.4 mg of **I-3** (0.1766 mmol) and 82 mg of 10% palladium on carbon were added to methanol (10 mL). The reaction mixture was stirred under 2 atm H_2 atmosphere for 6 h. TLC (DCM:MeOH 15:1, I-3 Rf= 0.8, product Rf= 0.1) was used to monitor the reaction. Once the reaction was complete, Pd/C was filtered out and the solvent was removed to obtain 111.0 mg a yellowish crude product, which was used for the sulfation step without further purification. The sulfation step was performed following general method B, and 100.8 mg of **I3C10S** was finally obtained as a white solid (two-step yield, 50.8%). **I3C10S** ^{1}H NMR (400 MHz, D_2O) δ 4.56 (t, *J* = 2.6 Hz, 1H, inositol H-O-C), 4.34 – 4.24 (m, 3H, inositol H-OSO_3^-), 4.11 (t, *J* = 6.6 Hz, 6H, CH_2-<u>CH_2</u>-OSO_3^-), 3.95 – 3.69 (m, 8H, O-<u>CH_2</u>-CH_2, inositol H-O-C), 1.82 – 1.27 (m, 48H, C-CH2-C). ^{13}C NMR (101 MHz, D_2O) δ 81.09, 77.60, 77.50, 75.79, 73.88, 69.81, 69.77, 29.38, 29.16, 29.06, 28.87, 28.78, 28.73, 28.67, 28.63, 28.55, 28.48, 28.45, 25.80, 25.07, 24.99. HRMS (nanochip-ESI/LTQ-Orbitrap) m/z: $[M]^{6-}$ Calcd for $C_{36}H_{66}O_{27}S_6^{6-}$ 187.0353; Found 187.0336.

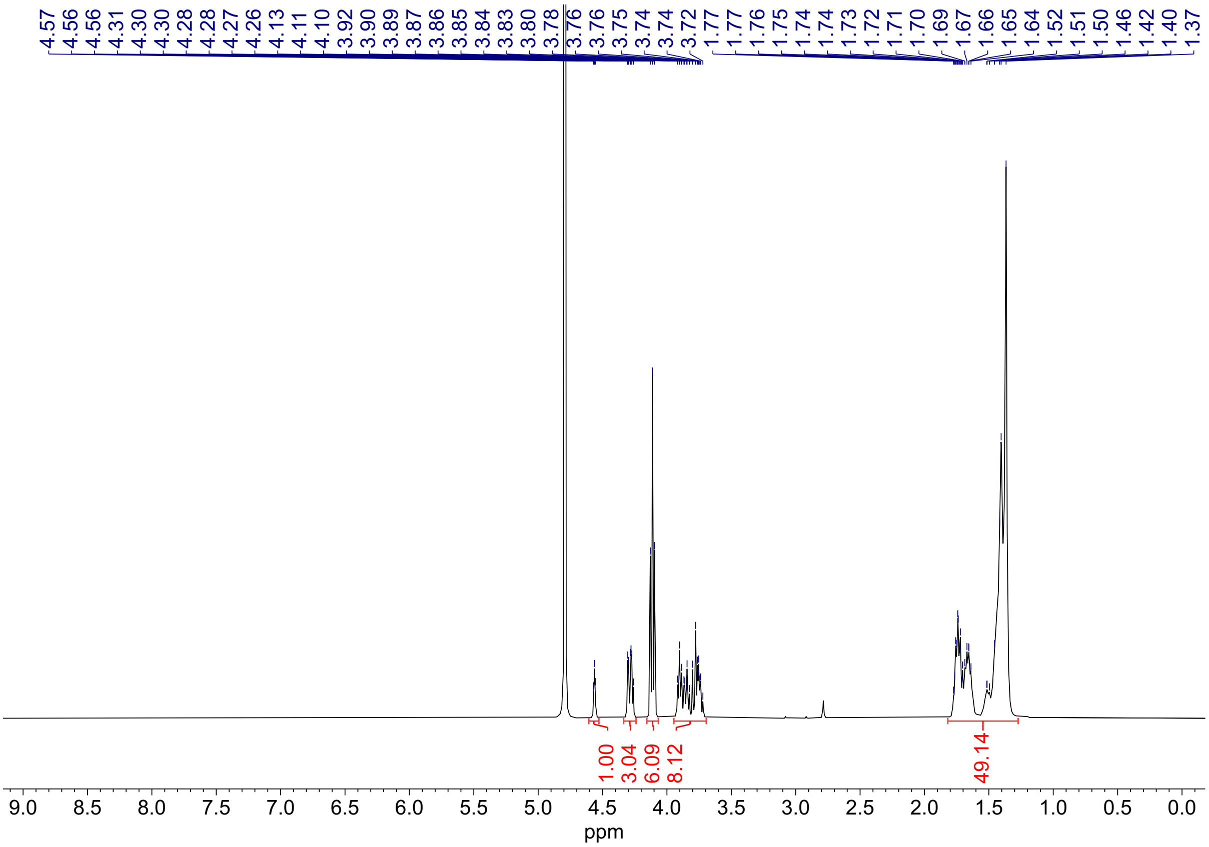

Figure S73. ^{1}H-NMR (D_2O, 400 MHz) of I3C10S

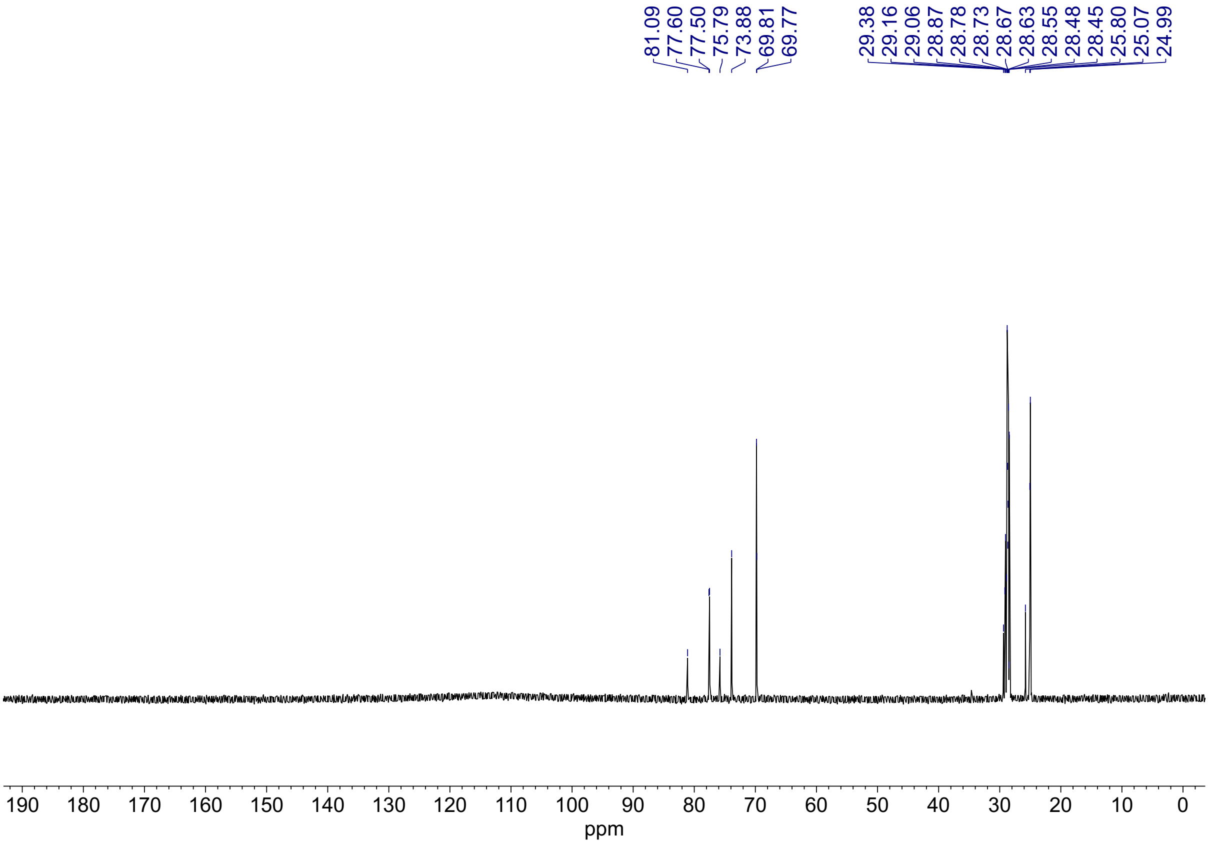

Figure S74. ^{13}C-NMR (D_2O, 101 MHz) of I3C10S

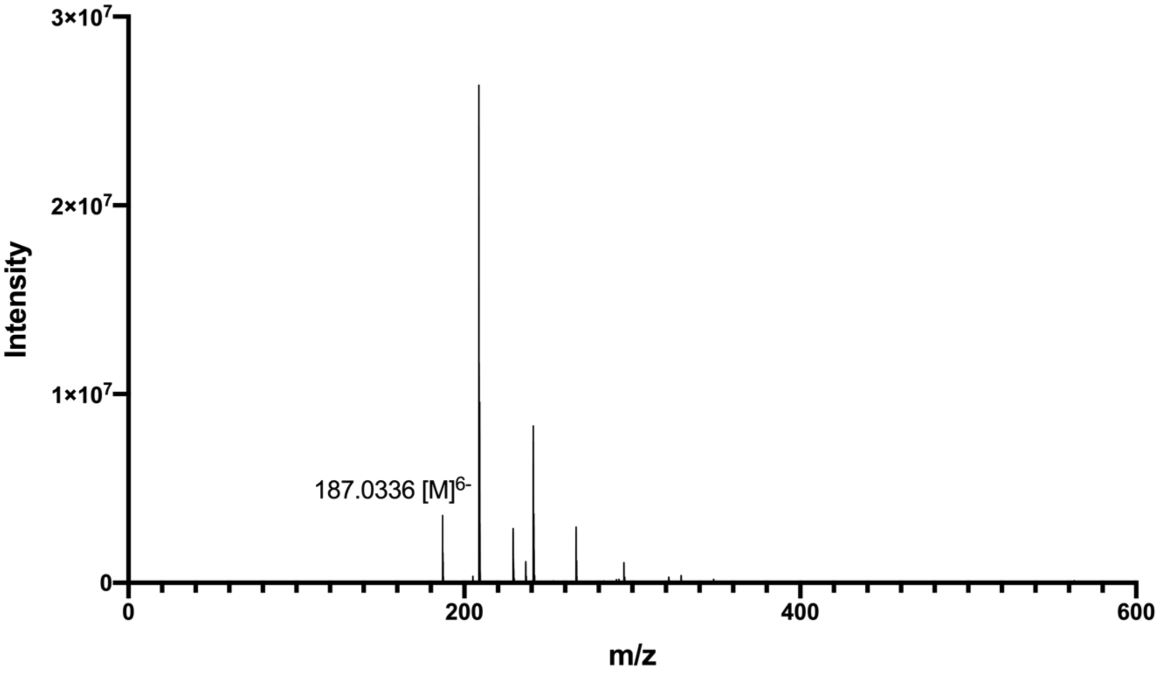

Figure S75. MS (nanochip-ESI/LTQ-orbitrap) of I3C10S

BET3C9OH

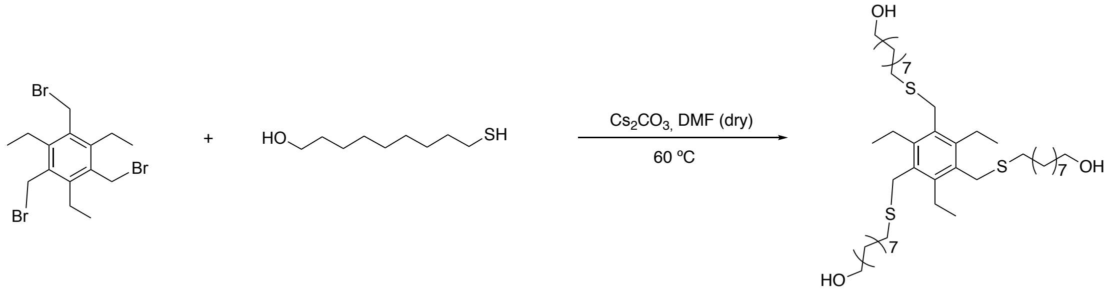

1,3,5-Tris(bromomethyl)-2,4,6-triethylbenzene and 9-mercapto-1-nonanol were used to synthesize compound BEt3C9OH following the general method A. The product was a white powder, yield 60.0%. ^{1}H NMR (400 MHz, $CDCl_3$) δ 3.71 (s, 6H, Ar-CH_2), 3.58 (t, J = 6.7 Hz, 6H, CH_2-O), 2.89 (q, J = 7.5 Hz, 6H, Ar-CH_2), 2.54 (t, J = 7.3 Hz, 6H, S-CH_2), 2.20 (s, 3H, OH), 1.56– 1.14 (m, 51H, C-CH_2-C, -CH_3). ^{13}C NMR (101 MHz, $CDCl_3$) δ 142.05, 131.71, 62.66, 33.49, 32.69, 31.25, 29.58, 29.52, 29.49, 29.41, 29.21, 28.89, 25.78, 22.86, 16.18. HRMS (nanochip-ESI/LTQ-Orbitrap) m/z: [M + Na]$^+$ Calcd for $C_{42}H_{78}NaO_3S_3^+$ 749.5005; Found 749.5019.

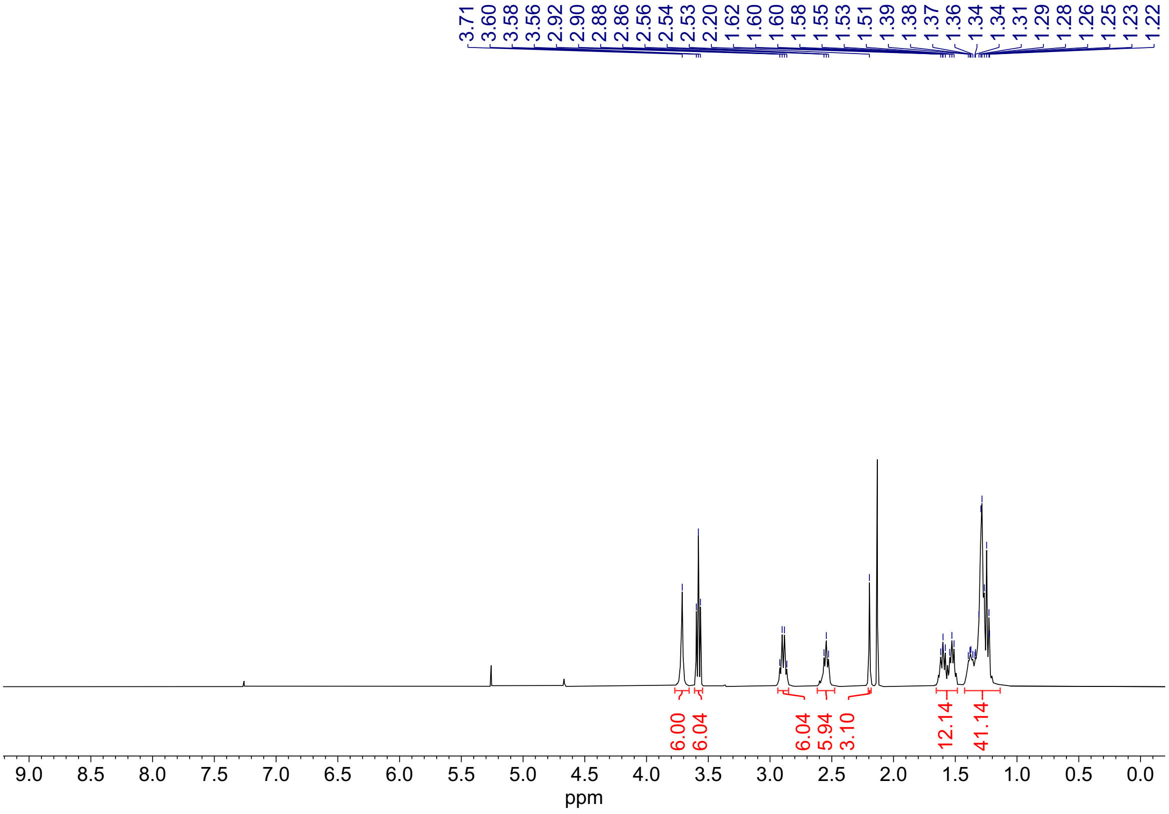

Figure S76. ^{1}H-NMR ($CDCl_3$, 400 MHz) of BET3C9OH

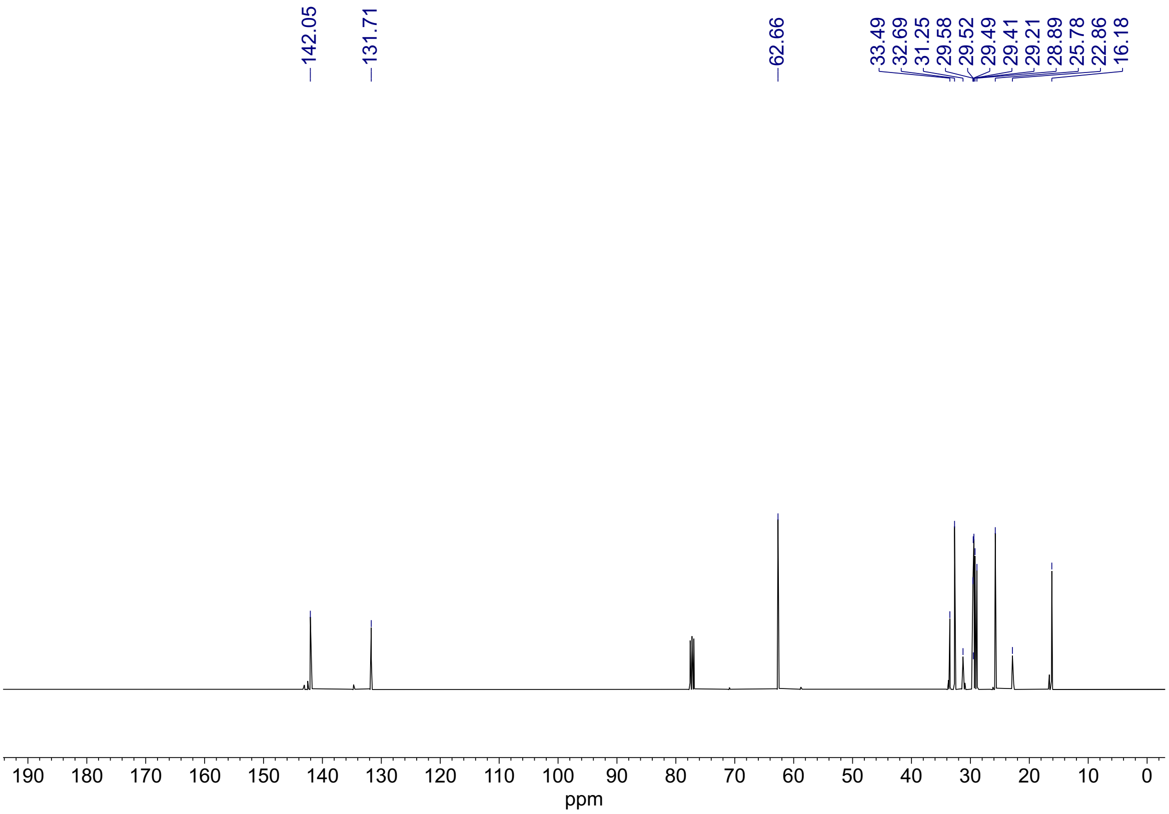

Figure S77. ^{13}C-NMR ($CDCl_3$, 101 MHz) of BET3C9OH

BET3C9S

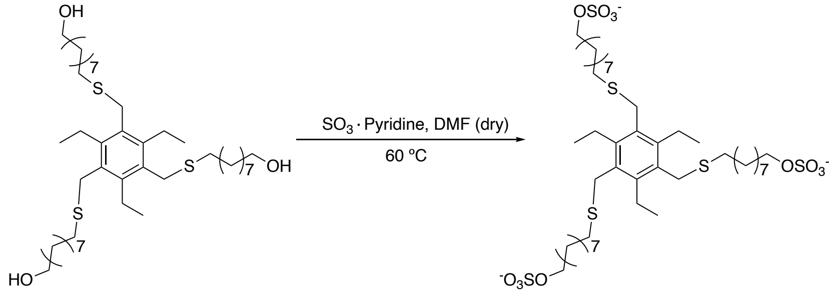

From compound BEt3C9OH following the general method B gave compound BEt3C9S as a white powder, yield 69.4%. ^{1}H NMR (400 MHz, D_2O) δ 4.05 (t, J = 6.7 Hz, 6H, CH_2-SO_4), 3.69 (s, 6H, Ar-CH_2-S), 2.84 (s, 6H, Ar-CH_2-C), 2.52 (s, 6H, S-<u>CH_2</u>-CH_2), 1.79 – 0.97 (m, 60H, CH_2-<u>CH_2</u>-CH_2, CH_3). ^{13}C NMR (101 MHz, D_2O) δ 142.50, 132.05, 69.73, 33.48, 31.52, 30.06, 29.78, 29.60, 29.58, 29.48, 29.20, 25.90, 23.20, 16.44. HRMS (nanochip-ESI/LTQ-Orbitrap) m/z: $[M]^{3-}$ Calcd for $C_{42}H_{75}O_{12}S_6^{3-}$ 321.1200; Found 321.1185.

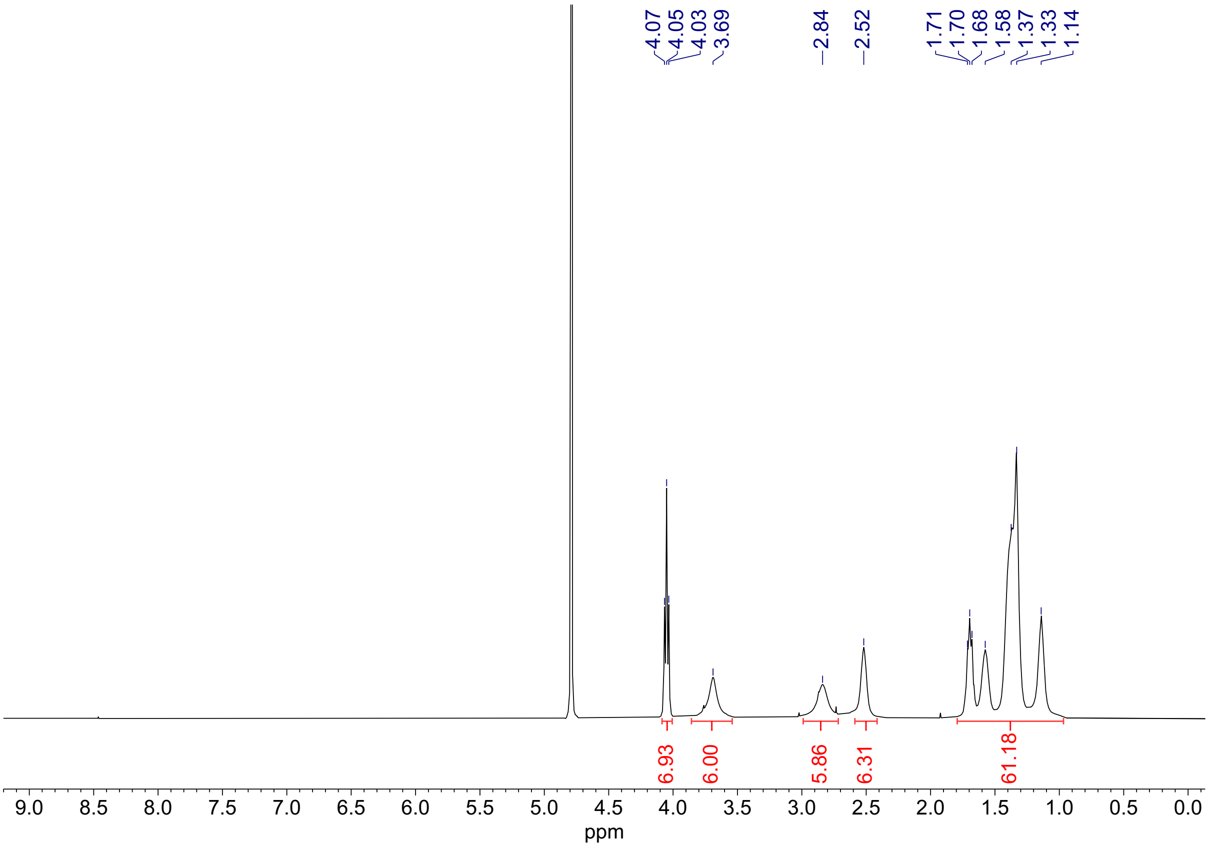

Figure S78. ^{1}H-NMR (D_2O, 400 MHz) of BET3C9S

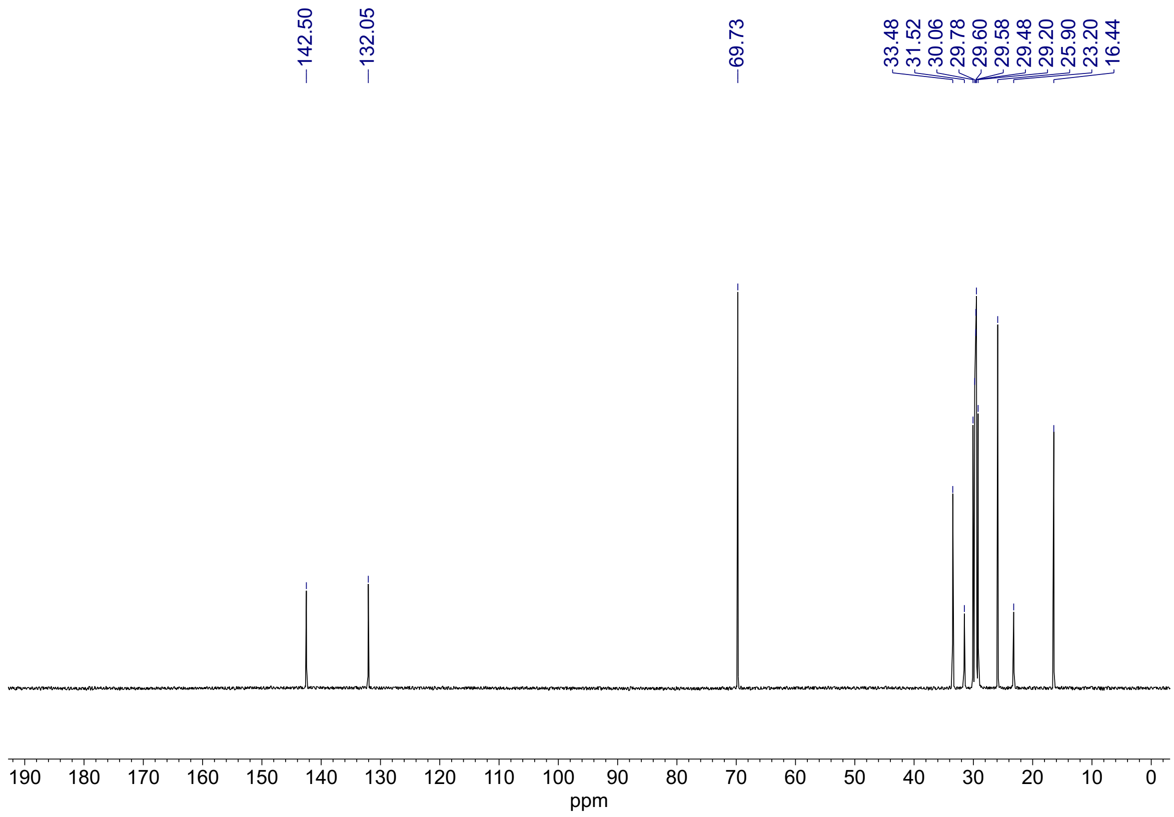

Figure S79. ^{13}C-NMR (D_2O, 101 MHz) of BETC9S

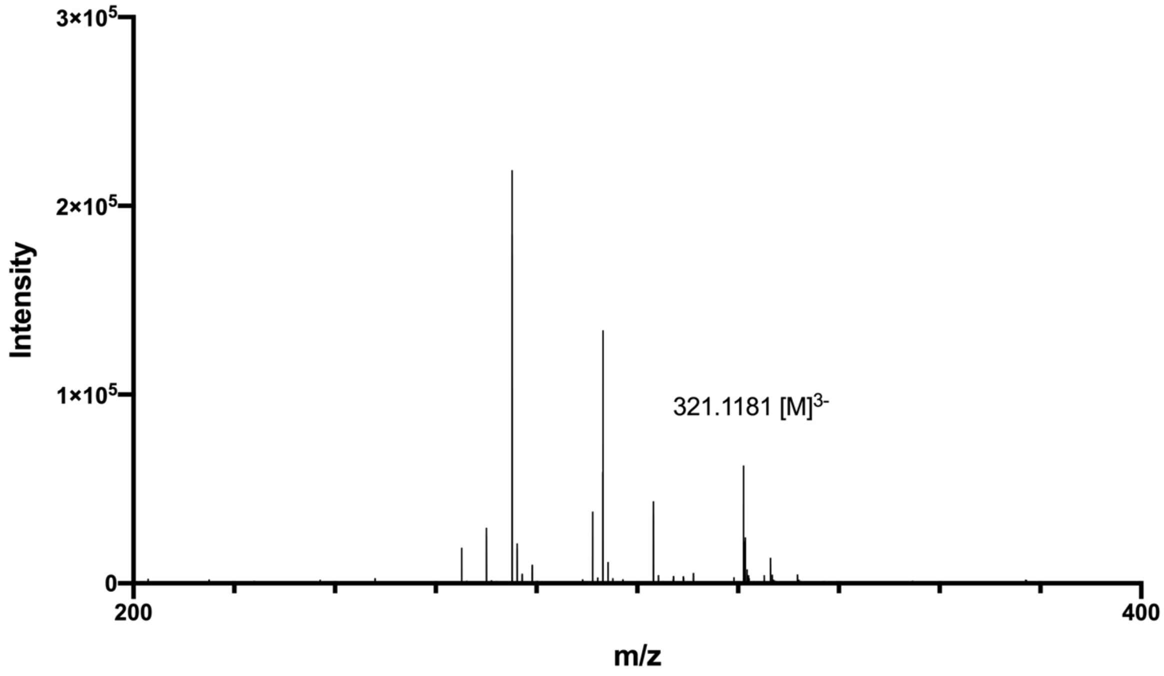

Figure S80. MS (nanochip-ESI/LTQ-orbitrap) of BETC9S

BET3C6OH

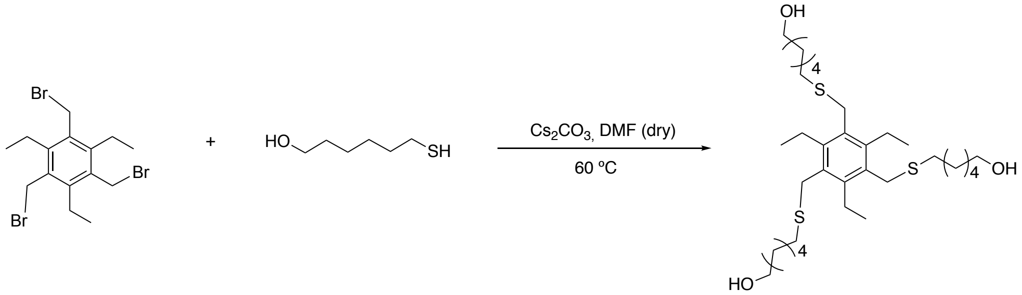

1,3,5-Tris(bromomethyl)-2,4,6-triethylbenzene and 6-mercapto-1-hexanol were used to synthesize compound BEt3C9OH, following the general method A. The product was a white powder, yield 62.0%. ^{1}H NMR (400 MHz, $CDCl_3$) δ 3.72 (s, 6H, Ar-CH_2), 3.58 (t, J = 6.6 Hz, 6H, CH_2-O), 2.90 (q, J = 7.5 Hz, 6H, Ar-CH_2), 2.55 (dd, J = 9.0, 5.6 Hz, 6H, S-CH_2), 1.67 – 1.19 (m, 33H, C-CH_2-C, -CH_3). ^{13}C NMR (101 MHz, $CDCl_3$) δ 142.12, 131.66, 62.68, 33.24, 32.58, 31.16, 29.52, 28.60, 25.37, 22.88, 16.16. HRMS (nanochip-ESI/LTQ-Orbitrap) m/z: $[M + Na]^+$ Calcd for $C_{33}H_{60}NaO_3S_3^+$ 623.3597; Found 623.3609.

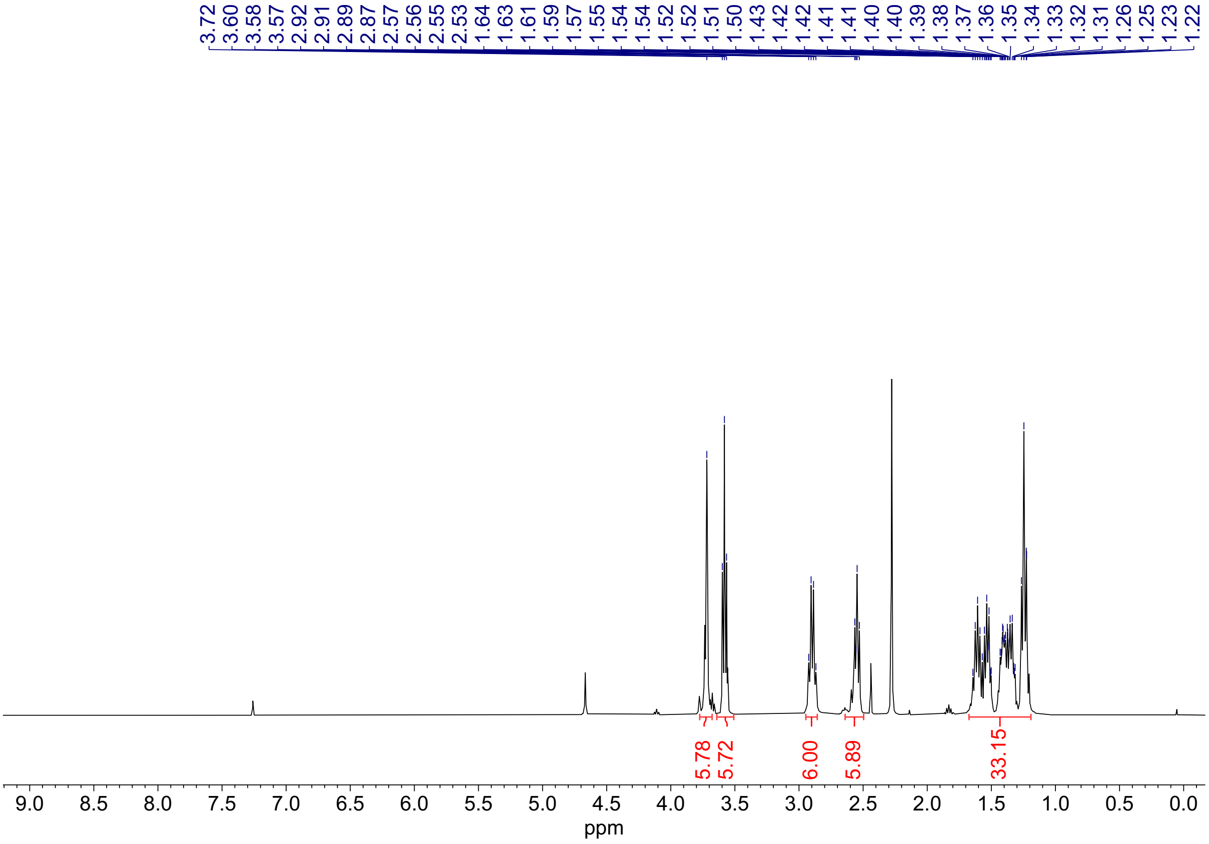

Figure S81. ^{1}H-NMR ($CDCl_3$, 400 MHz) of BET3C6OH

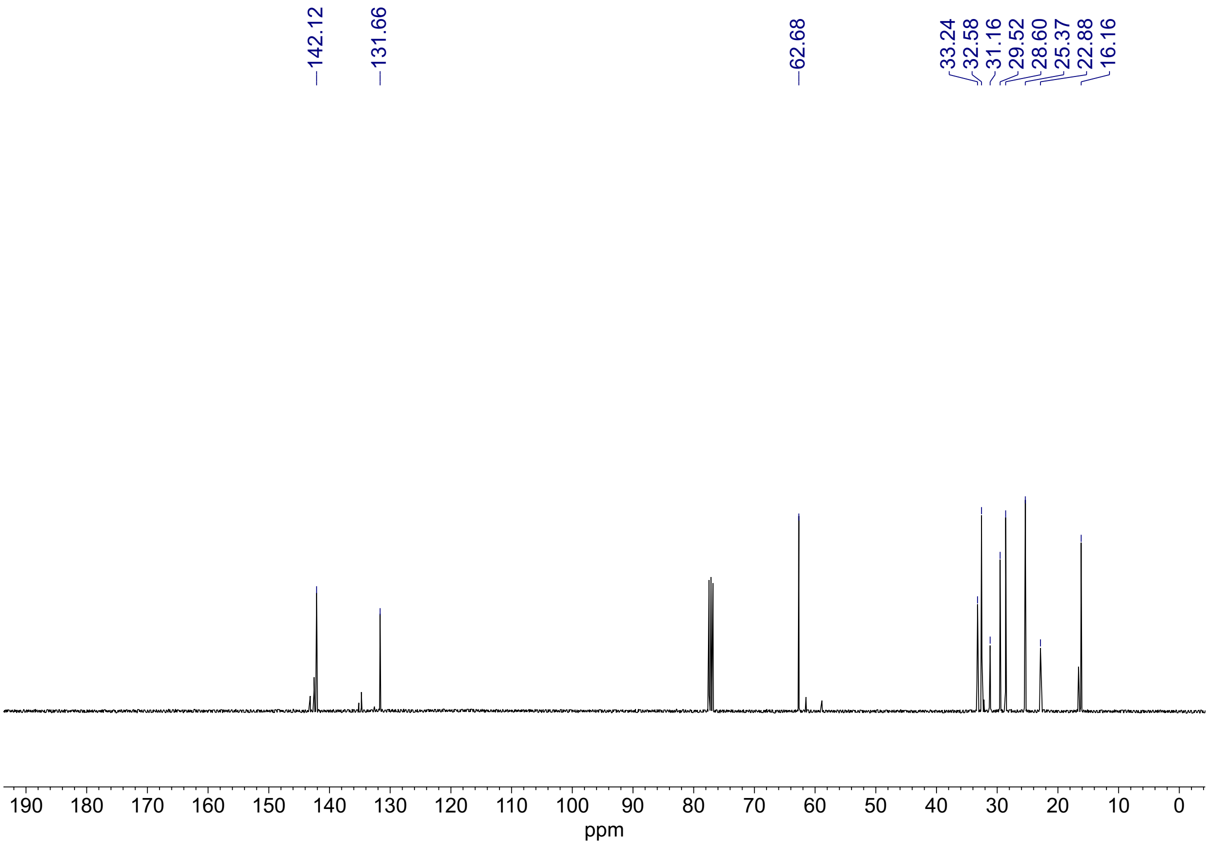

Figure S82. ^{13}C-NMR ($CDCl_3$, 101 MHz) of BET3C6OH

BET3C6S

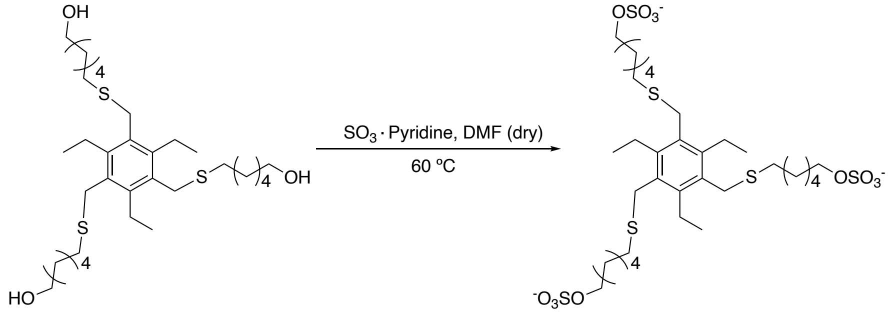

From compound BEt3C6OH following the general method B gave compound BEt3C6S as a white powder, yield 67.2%. ^{1}H NMR (400 MHz, D_2O) δ 4.05 (t, J = 6.7 Hz, 6H, CH_2-SO_4), 3.69 (s, 6H, Ar-CH_2-S), 2.84 (s, 6H, Ar-CH_2-C), 2.52 (s, 6H, S-$\underline{CH_2}$-CH_2), 1.79 – 0.97 (m, 60H, CH_2-$\underline{CH_2}$-CH_2, CH_3). ^{13}C NMR (101 MHz, D_2O) δ 142.50, 132.05, 69.73, 33.48, 31.52, 30.06, 29.78, 29.60, 29.58, 29.48, 29.20, 25.90, 23.20, 16.44. HRMS (nanochip-ESI/LTQ-Orbitrap) m/z: $[M]^{3-}$ Calcd for $C_{33}H_{57}O_{12}S_6^{3-}$ 279.0730; Found 279.0728.

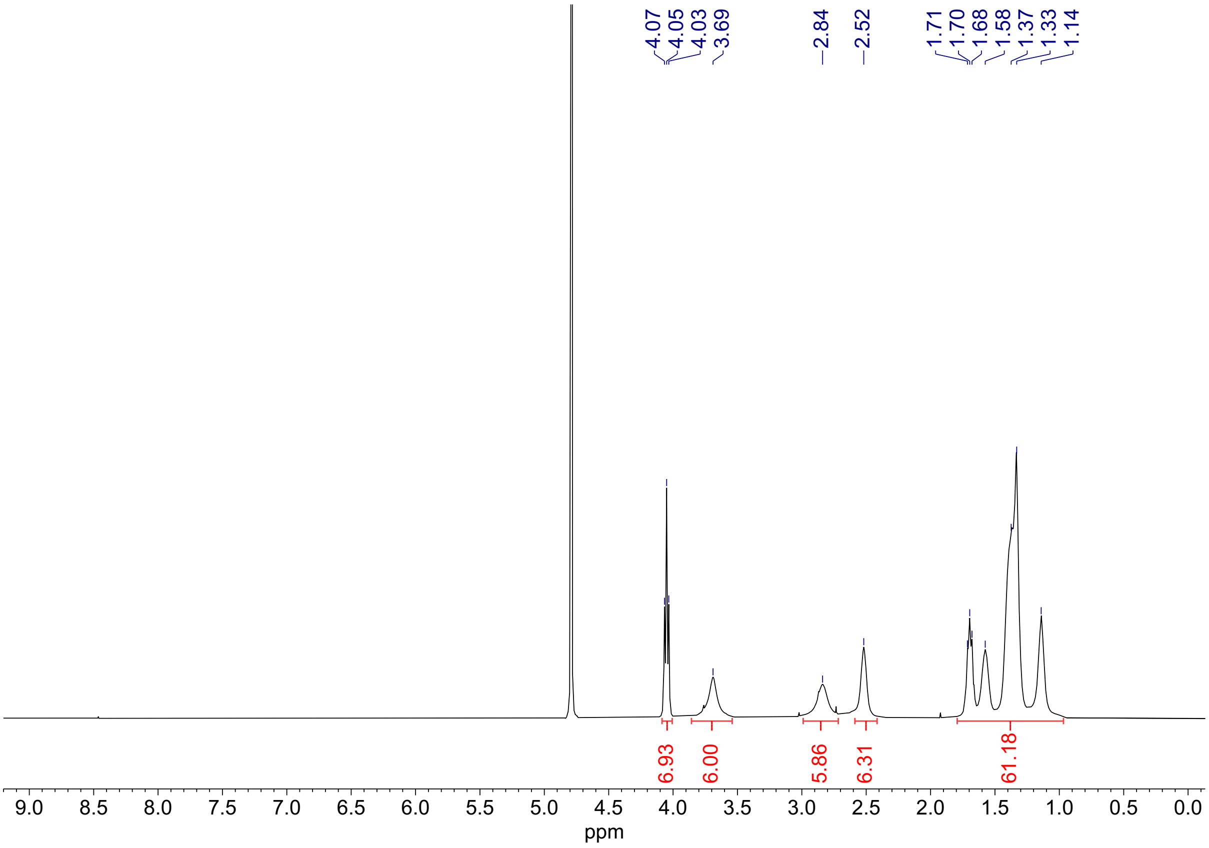

Figure S83. ¹H-NMR (D_2O, 400 MHz) of BET3C6S

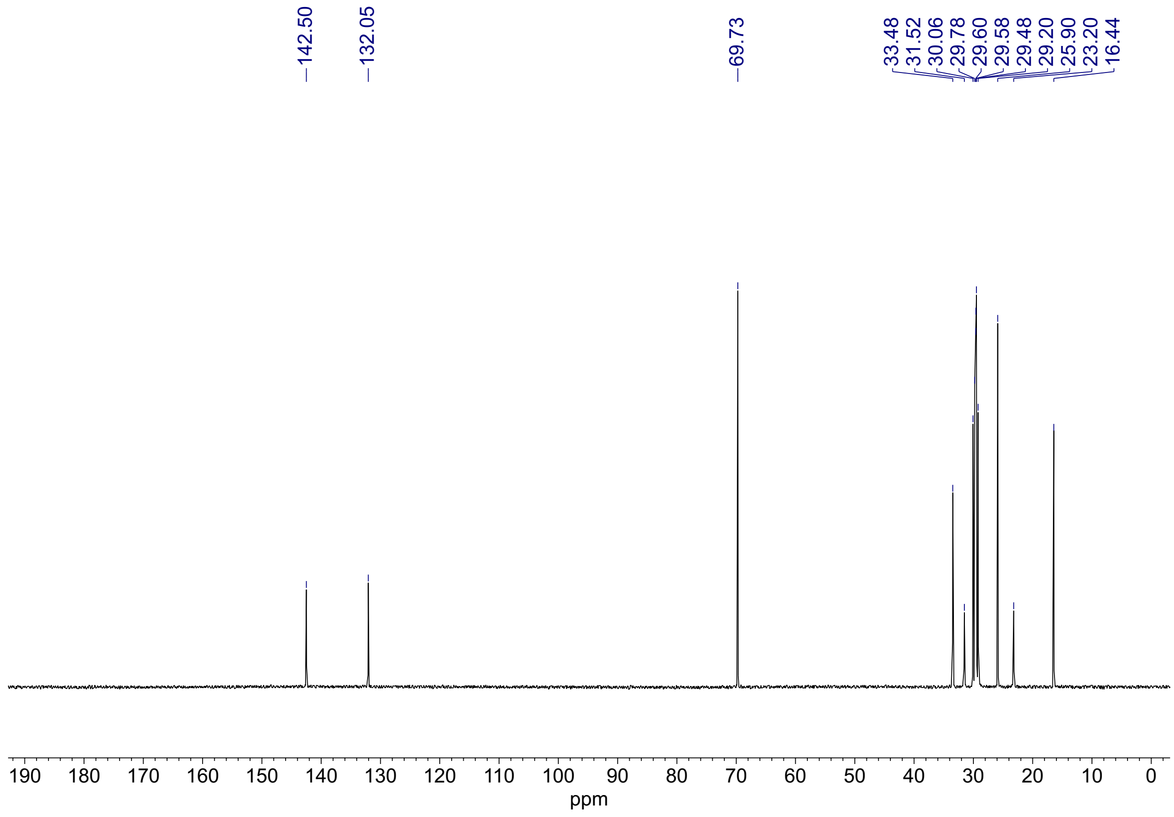

Figure S84. ¹³C-NMR (D_2O, 101 MHz) of BET3C6S

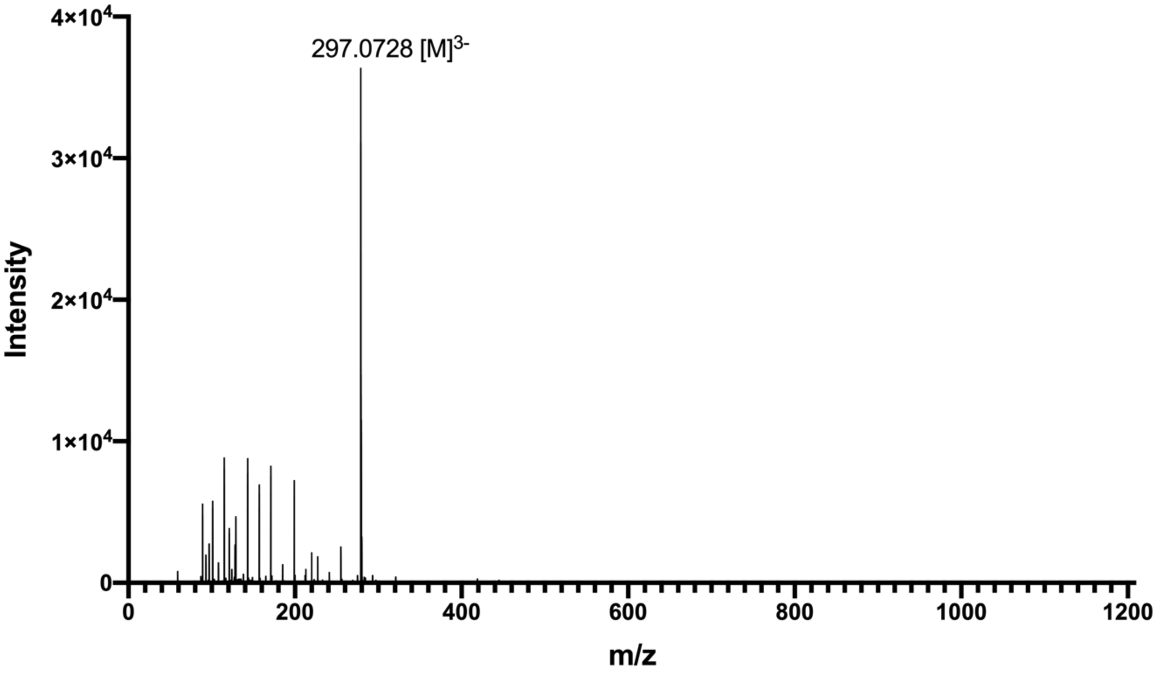

Figure S85. MS (nanochip-ESI/LTQ-orbitrap) of BET3C6S

BTA3C10OH

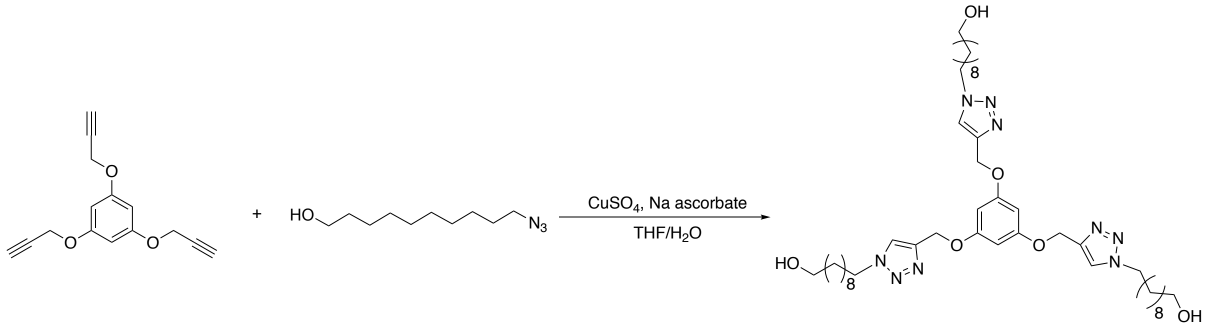

1,3,5-Tris(2-propynyloxy)benzene (100 mg, 0.416 mmol), 10-Azido-1-decanol (303.6 mg, 1.523 mmol, 3.66 eq), anhydrous $CuSO_4$ (24.2 mg, 0.152 mmol, 0.37 eq) and sodium ascorbate (30.1 mg, 1.52 mmol, 3.66 eq) were dissolved in 2 mL of THF/H_2O (1:1) mixture. The reaction solution was stirred at room temperature for two days until the product spot (Rf=0.7) with CAM stain on TLC (DCM:MeOH=20:1) stopped growing darker. After removing the reaction solvent by rotavapor, a flash chromatography system (Biotage) with silica gel column was used to purify the crude product to obtain 119 mg of white powder, yield 34.4%. ^1H NMR (400 MHz, DMSO, $CDCl_3$) δ 8.21 (s, 3H, Ar-H), 6.32 (s, 3H, CH=CR2), 5.08 (s, 6H, O-CH_2-C=), 4.35 (t, *J* = 7.0 Hz, 6H, N-CH_2), 3.36 (s, 6H, $\underline{CH_2}$-OH), 1.81 (d, *J* = 8.8 Hz, 6H, N-C-CH_2), 1.23 (s, 42H, C-CH_2-C). ^{13}C NMR (101 MHz, CD_3OD, $CDCl_3$) δ 159.84, 143.32, 123.16, 94.89, 61.96, 61.42, 50.40, 32.20, 29.93, 29.16, 29.10, 29.02, 28.65, 26.14, 25.47. HRMS (nanochip-ESI/LTQ-Orbitrap) m/z: $[M + Na]^+$ Calcd for $C_{45}H_{75}N_9NaO_6^+$ 860.5733; Found 860.5755.

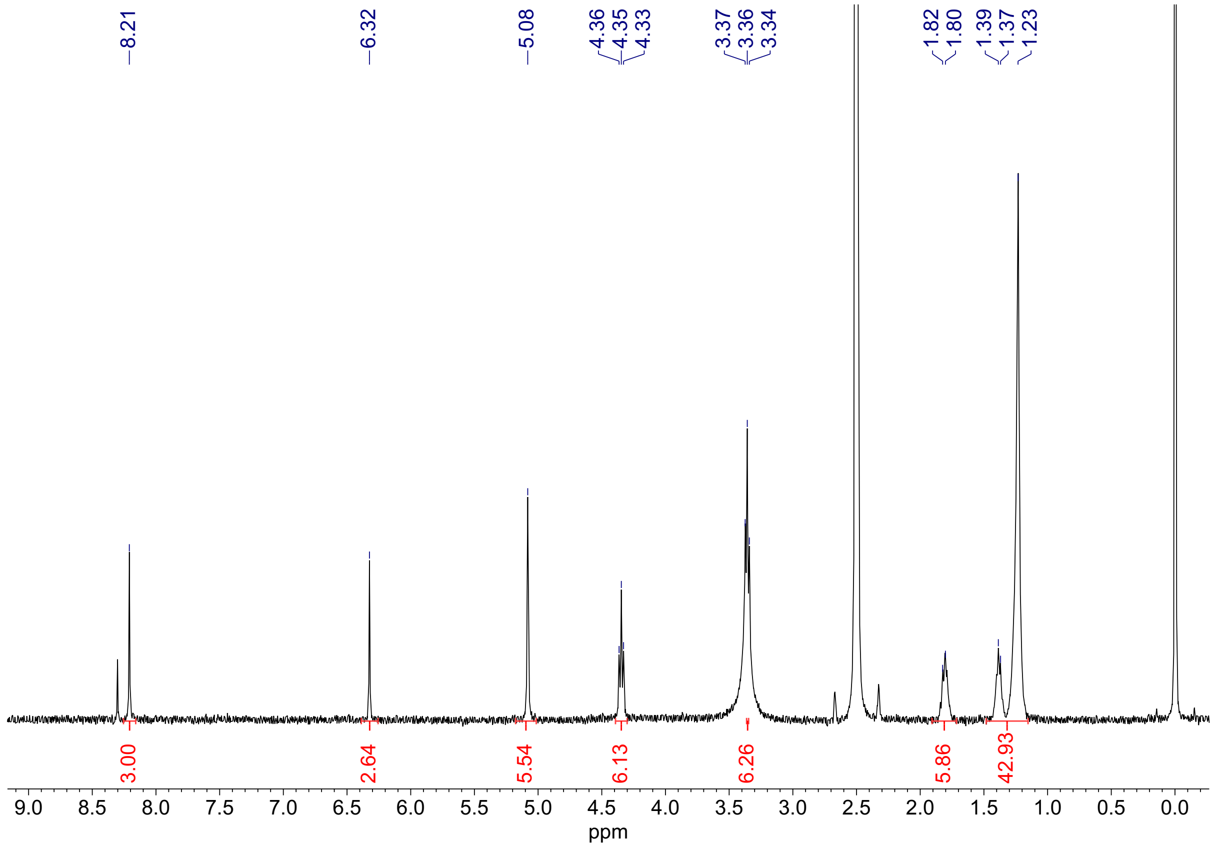

Figure S86. ^{1}H-NMR ($CDCl_3$, 400 MHz) of BTA3C10OH

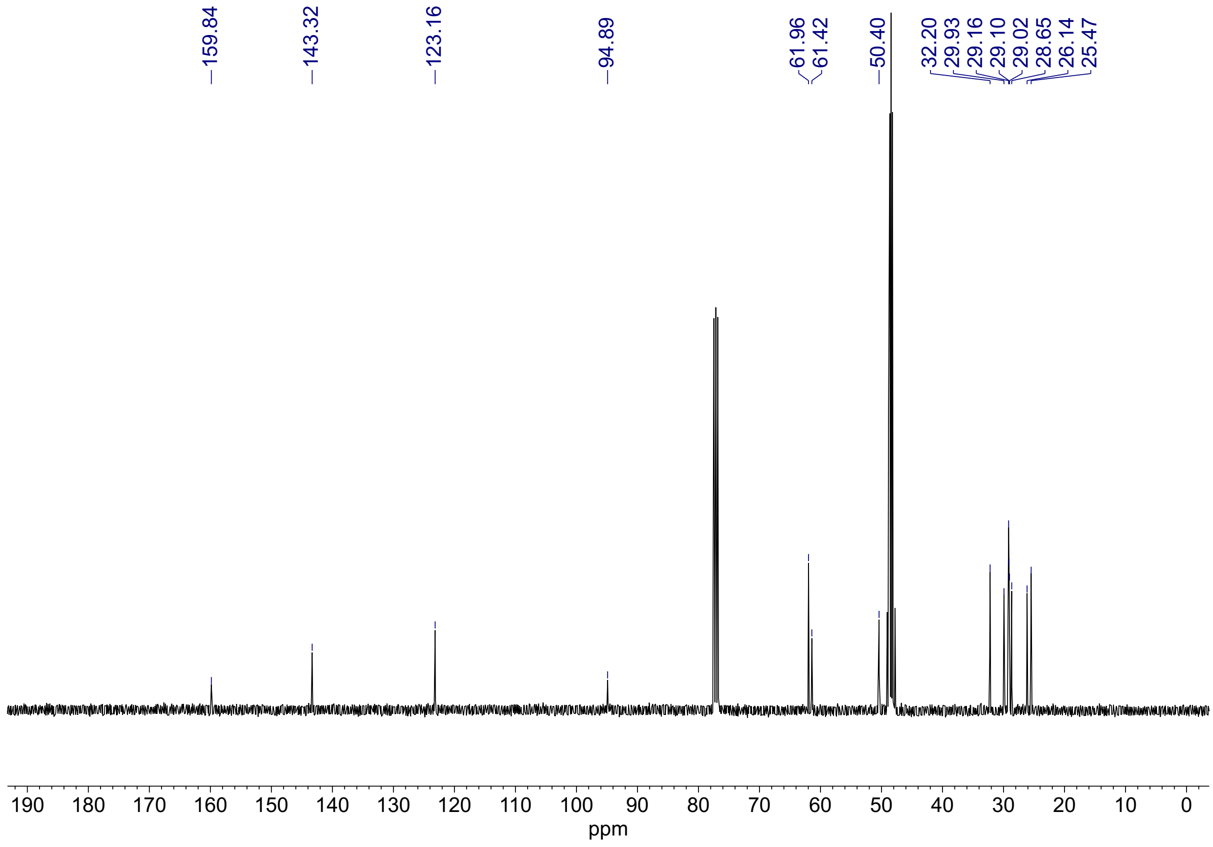

Figure S87. ^{13}C-NMR ($CDCl_3$, 101 MHz) of BTA3C10OH

BTA3C10S

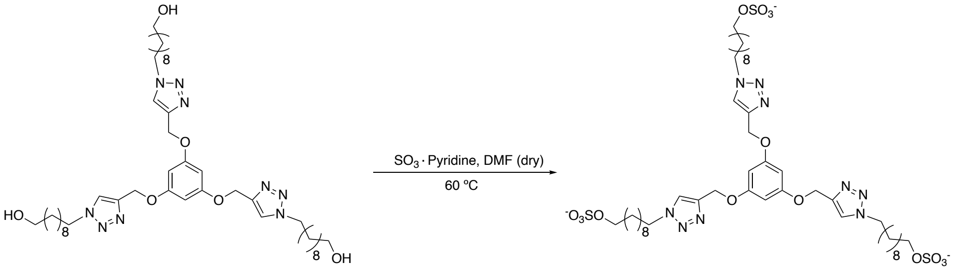

From compound BTA3C10OH following the general method B gave compound BTA3C10S as a white powder, yield 68.0%. ^{1}H NMR (400 MHz, D_2O) δ 7.92 (s, 3H, Ar-H), 6.23 (s, 3H, CH=C), 5.05 (s, 6H, O-CH_2-C=), 4.30 (s, 6H, N-CH_2-), 3.99 (t, J = 6.7 Hz, 6H, CH_2-SO_4), 1.87 – 0.79 (m, 48H, C-CH_2-C). ^{13}C NMR (101 MHz, D_2O) δ 160.27, 143.79, 124.61, 96.49, 69.68, 62.14, 50.74, 29.99, 29.22, 29.21, 29.21, 29.07, 28.82, 26.30, 25.56. HRMS (nanochip-ESI/LTQ-Orbitrap) m/z: $[M]^{3-}$ Calcd for $C_{45}H_{72}N_9O_{15}S_3^{3-}$ 358.1442; Found 358.1438.

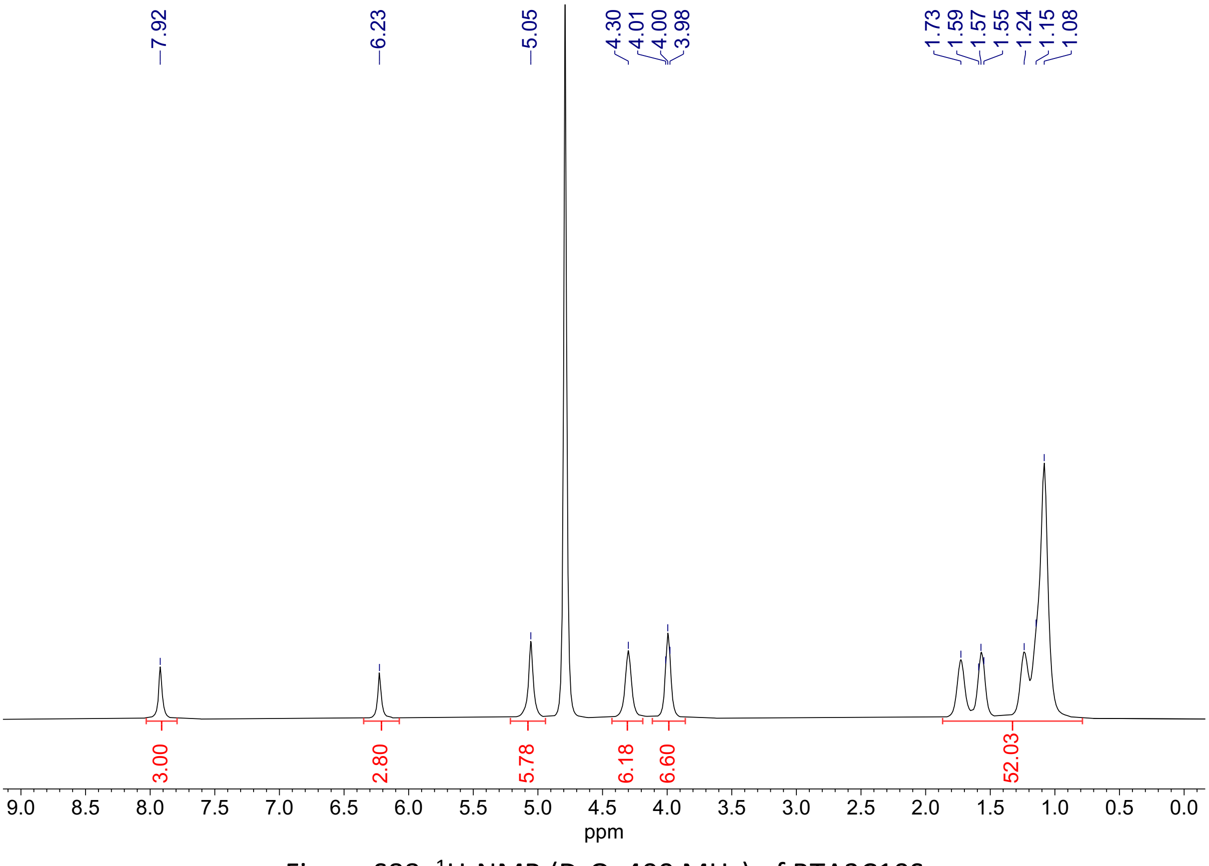

Figure S88. ^{1}H-NMR (D_2O, 400 MHz) of BTA3C10S

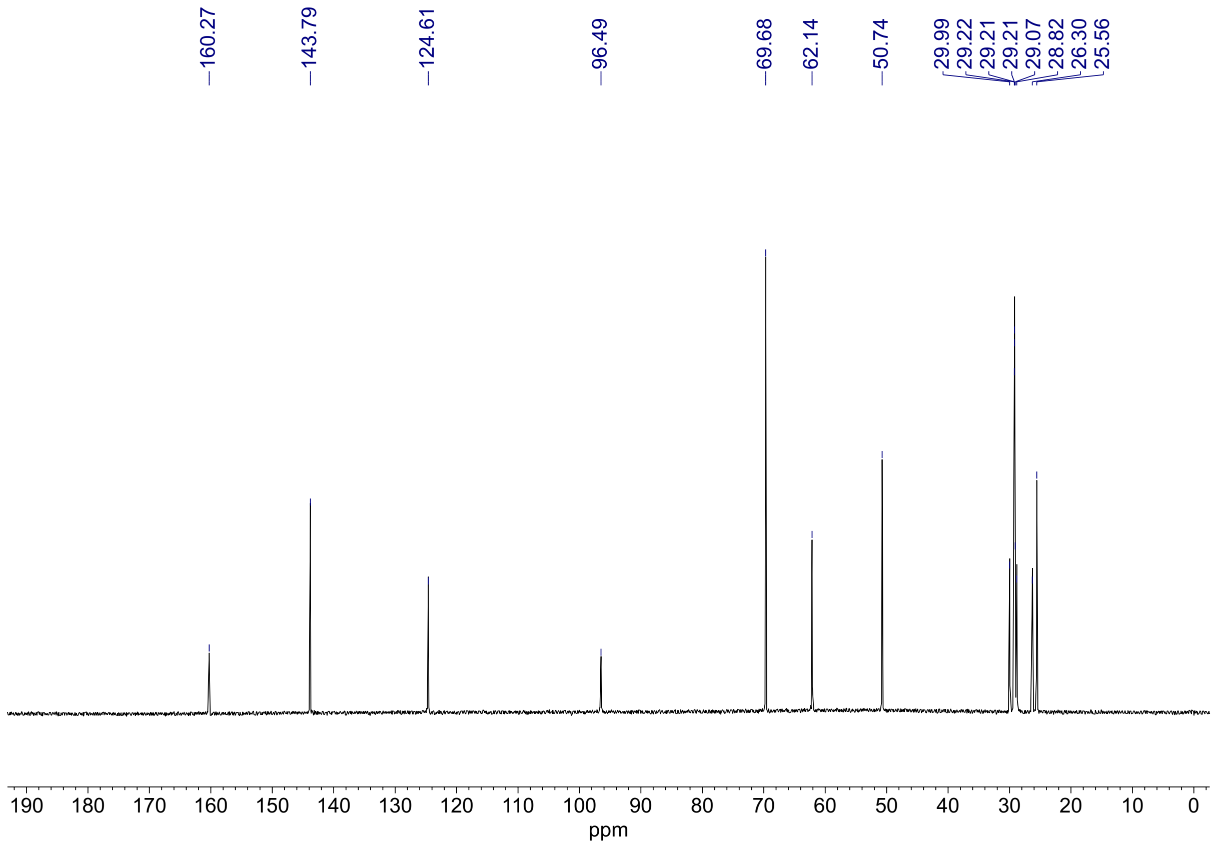

Figure S89. ^{13}C-NMR (D_2O, 101 MHz) of BTA3C10S

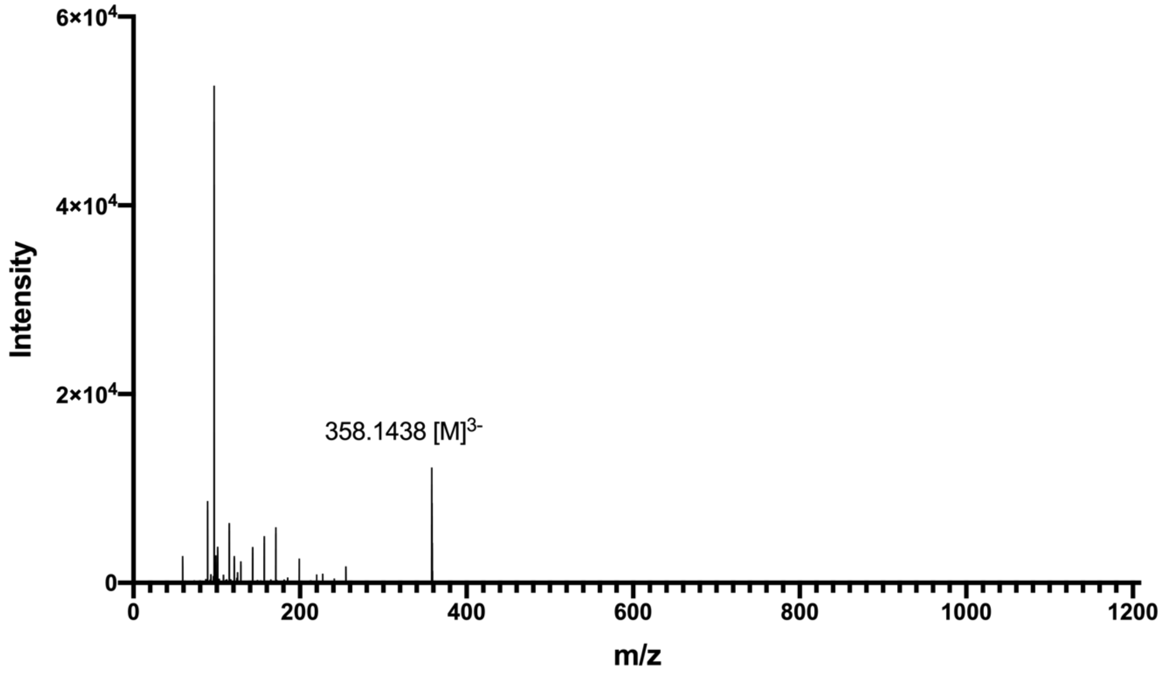

Figure S90. MS (nanochip-ESI/LTQ-orbitrap) of BTA3C10S

2. Synthesis of multivalent sulfonate B3C11Sulfonate

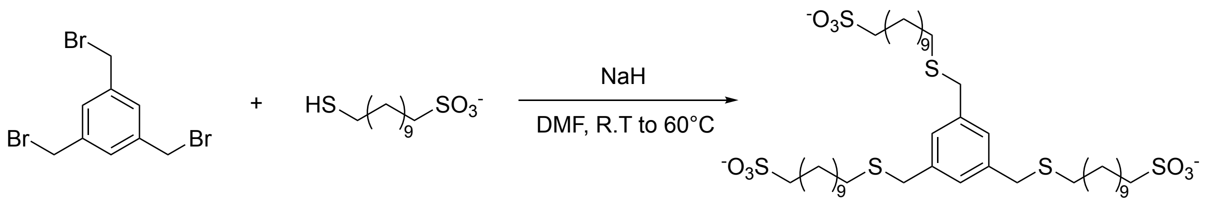

NaH (60% dispersed in mineral oil), 168 mg, 4.2 mmol) was added to a solution of sodium 11-mercaptoundecane-1-sulfonate (1.22 g, 4.2 mmol) in dry DMF (15 mL). After 30 min of stirring at R.T., a solution of 1,3,5-tris(bromomethyl)benzene (357 mg, 1 mmol) in dry DMF (10 mL) was added. The temperature was raised to 60°C and the stirring continued overnight. After cooling to r.t., ethanol (20 mL) was added and the solvent was removed under vacuum. The solid residue was purified by reversed phase flash chromatography (C18, AcCN/water gradient) to afford the product (798 mg, 0.8 mmol, 81 %) as a white solid. ^{1}H NMR (400 MHz, D_2O) δ 7.13 (s, 3H, Ar-H), 4.03 (t, *J* = 6.7 Hz, 6H, CH_2-SO_4-), 3.64 (s, 6H, Ar-CH_2), 2.37 (d, *J* = 7.6 Hz, 6H, S-CH_2), 1.80 – 1.09 (m, 42H, CH_2-$\underline{CH_2}$-CH_2).^{13}C NMR (101 MHz, DMSO-d6) δ 139.33, 128.18, 51.98, 35.32, 30.90, 29.51, 29.47, 29.45, 29.26, 29.12, 28.96, 28.80, 25.57. HR-MS (nanochip-ESI/LTQ-Orbitrap) m/z: $[M]^{3-}$ Calcd for $C_{42}H_{75}O_9S_6^{3-}$ 305.1251; Found 305.1237.

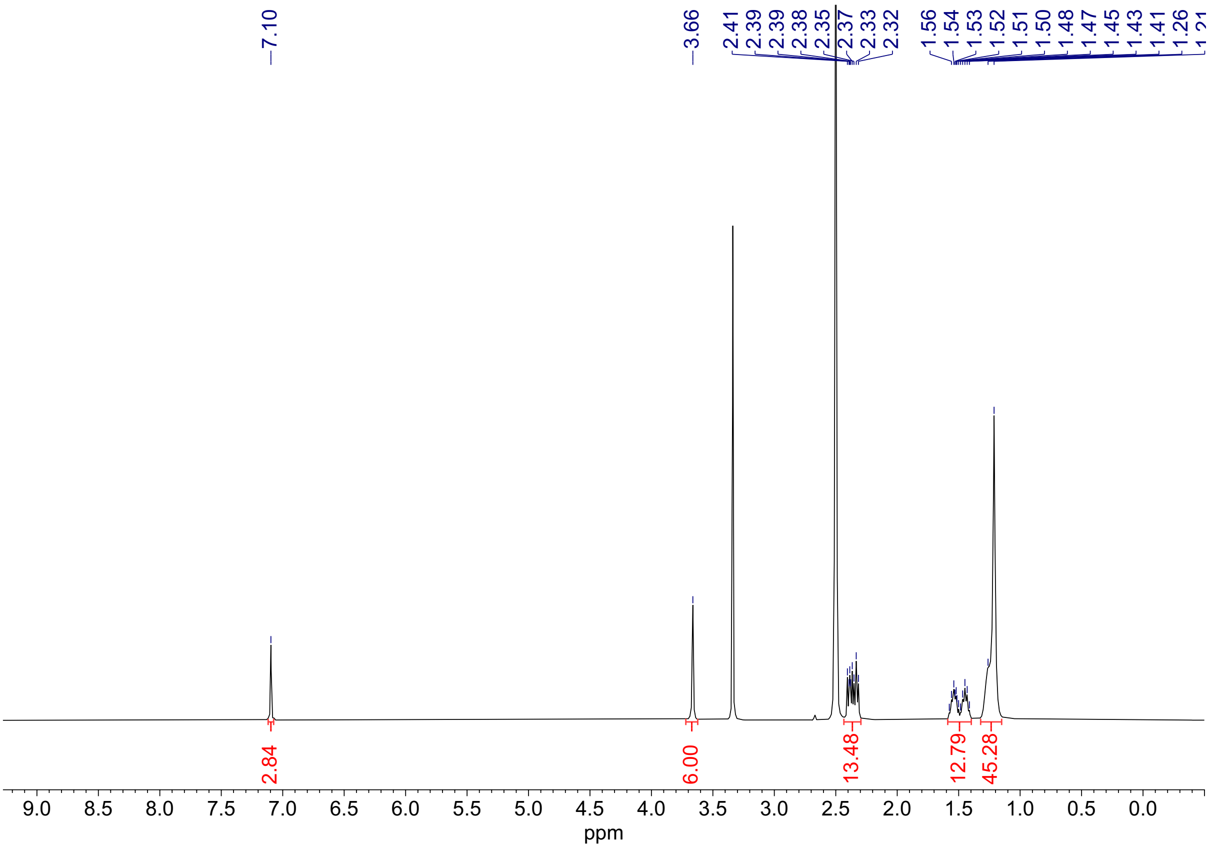

Figure S91. ^{1}H-NMR (DMSO-d6, 400 MHz) of B3C11Sulfonate

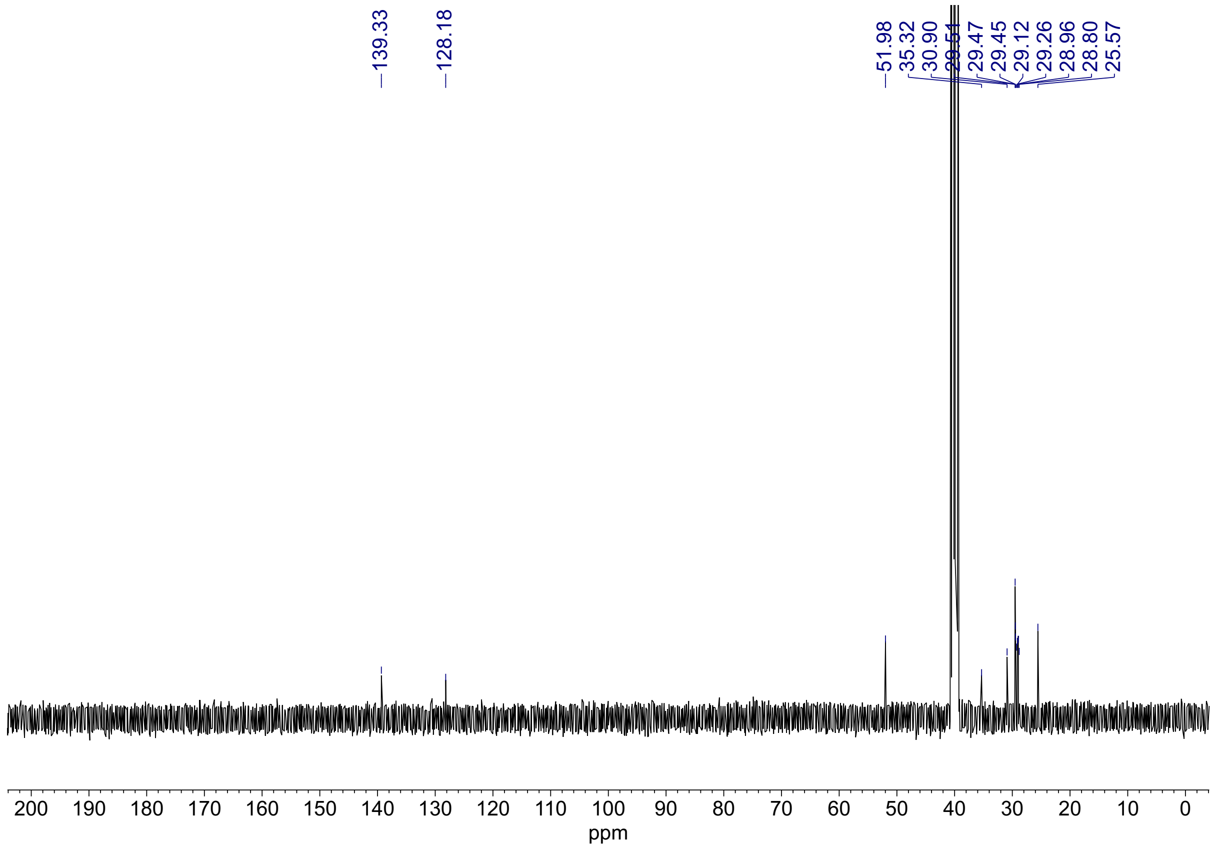

Figure S92. ^{13}C-NMR (DMSO-d6, 101 MHz) of B3C11Sulfonate

3. Synthesis of multivalent sulfonate CD-M11sulfates

Synthesis of CD-M11Sulfate

Heptakis-(6-deoxy-6-mercapto)-β-CD (100 mg, 0.080 mmol), sodium undec-10-ene-1-sulfate (305 mg, 1.12 mmol), were dissolved in DMSO (5 mL). The reaction mixture was placed in front of an ultraviolet (UV) lamp (400 W) and stirred for 12 h. The crude product was precipitated by into a 20% EtOH in Et_2O mixture (45 ml) and collected by centrifugation. The off-white solid was washed with MeOH (45 ml) and EtOH (45 ml) and collected by centrifugation. The product was purified by dialysis against Milli-Q H_2O for 3 days, with 4 changes of water, filtered through a 0.2-µm filter, lyophilised and collected as a white solid (210 mg). Confirmed by High-resolution mass spectrometry (HRMS) [electrospray ionization (ESI)/quadrupole time-of-flight (QTOF)] mass/charge ratio (m/z): $[M]^{7-}$ calculated for $C_{119}H_{217}O_{49}S_{14}^{7-}$ 427.5471; found 427.4290.

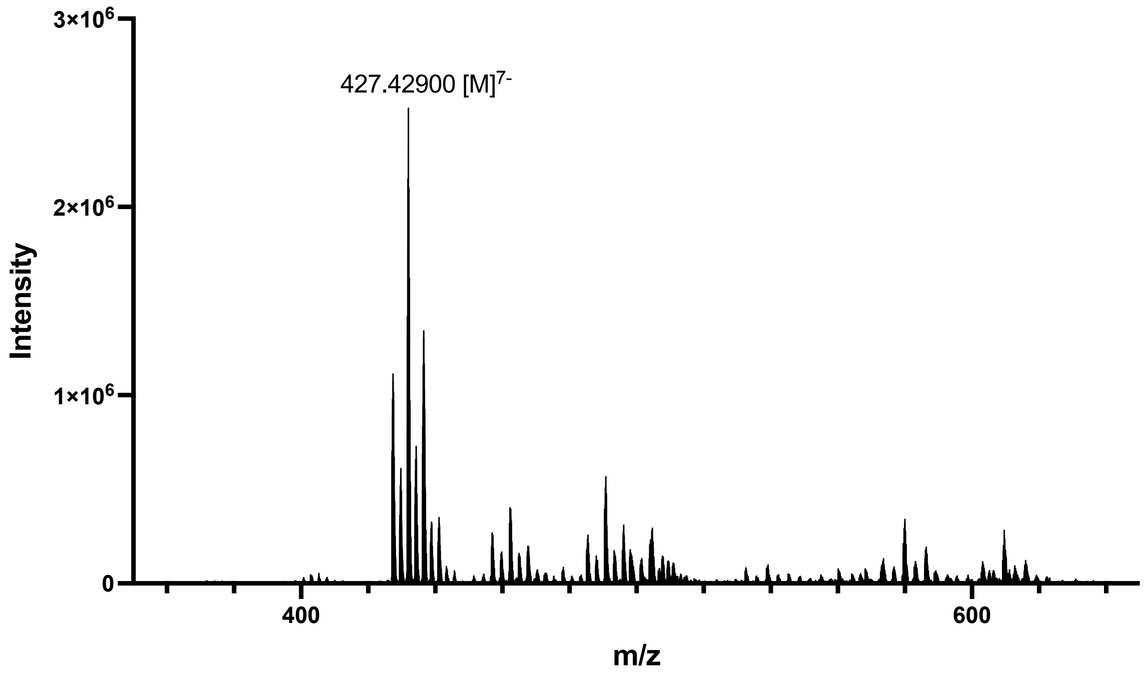

Figure S93. MS (nanochip-ESI/LTQ-orbitrap) of CD-M11Sulfate

4. *In vitro* antiviral and cytotoxicity assays

Cell lines and viruses

Vero (ATCC, CCL-81) and Vero E6 (ATCC, CCL-1586) cells were cultured in Dulbecco's modified Eagle's medium (DMEM, Gibco) containing 10% FBS (Gibco) and 1% penicillin/ streptomycin (Sigma-Aldrich). Cells were grown in humidified atmosphere with 5% CO_2 at 37 °C.

HSV-2 was provided by M. Pistello (University of Pisa, Italy) and it was propagated and titrated on Vero cells with plaque assays.

SARS-CoV-2/Switzerland/GE9586/2020 was isolated from a clinical specimen in the University Hospital in Geneva in Vero E6 and passaged twice before the experiments.

SARS-CoV-2 (alpha) used in the hamster study was originated from a patient hospitalized in March 2020 at Liège University hospital.

Inhibition assay against HSV-2

Vero cells were pre-seeded in a 24-well plate. Various concentrations of compounds were incubated with HSV-2 (MOI = 0.0005) virus in DMEM with 2% FBS and 1% P/S for 1h at 37 °C. After transferring the mixture to the cells and incubating 1h for viral attachment, the cells were washed with 1x PBS and overlaid with 0.45% methylcellulose medium before incubating at 37 °C overnight, followed by staining with crystal violet containing ethanol for 20 min for plaque counting. Virus infectivity was determined by the ratio of plaque number in treated wells and plaque number in control wells.

Virucidal assay against HSV-2

Antiviral compounds (175 µg/mL for B3C6S, 300 µg/mL for P3C9S and CD-M11Sulfate, 1mg/mL for I3C10S, 2mg/ml for P2C9S, 400 µg/mL for B3C11Sulfonate, 100 µg/mL for other molecules) and HSV-2 virus (10^5 pfu) were incubated for 1h at 37 °C, and then the serial dilutions of the virus-compound mixture were added to wells plated with Vero cells. After one-hour incubation at 37 °C, the washing, incubating and staining steps were performed

sequentially as described for inhibition assay. Viral titers were determined at dilutions at which the material was present at a concentration below the previously measured EC_{50}.

Inhibition assay against SARS-CoV-2

Serial dilutions of compounds were incubated with SARS-CoV-2 (MOI = 0.0005) in DMEM with 1% P/S for 1 h at 37 °C. Vero E6 cells pre-plated in 24-well plate were treated with these mixtures for 1h at 37 °C, and then washed and overlaid with 0.4% avicel rc581 in DMEM supplemented with 5% FBS. 48 h after inoculation, the cells were fixed with 4% Paraformaldehyde for 15 min then stained with crystal violet solution. Plaques were counted and the viral infectivity was calculated by comparing the number of treated well and that of control well.

Virucidal assay against SARS-CoV-2

Antiviral compounds (1000 µg/mL for P3C9S, 1mg/mL for I3C10S, 300 µg/mL for BETC9S, 300 µg/mL for other molecules) in DMEM with 1% P/S and SARS-CoV-2 virus (10^5 pfu) were incubated for 1h at 37 °C, and then the serial dilutions of the virus-compound mixture were added to wells plated with Vero cells. After one-hour, the washing, the incubation and the staining steps were performed sequentially as described for inhibition assay. Viral titers were determined at dilutions at which the material was present at a concentration below the previously measured EC_{50}.

Inhibition assay against H1N1

MDCK cells were pre-plated 24 h in advance in 96-well plates. Serial dilutions of compound were prepared in DMEM with P/S and incubated with the influenza virus (Influenza A/Netherlands/2009 (H1N1), MOI=0.001) at 37 °C for one hour and then the mixtures were added to the cells. Following the virus adsorption (1 h at 37 °C), the inoculum was removed, the cells were washed and a fresh medium was added. After 24 h of incubation at 37°C, the infection was analyzed with immunocytochemical (ICC) assay. The cells were fixed and permeabilized with methanol. Then, the Flu A monoclonal antibody (1:100 dilution, Light Diagnostics) was added and incubated for 1 hour at 37°C. The cells were washed with wash buffer (PBS + Tween 0.05%) three times. Then, anti-mouse lgG, HRP-linked antibody (1:500 dilution, Cell signaling technology) was added. After 1 hour the cells were washed and the DAB solution (Sigma) was added. The infected cells were counted and percentages of infection were calculated comparing the number of infected cells in treated to those under untreated conditions.

Virucidal assay against H1N1

H1N1 Viruses (10^5 ffu/mL) and the materials at EC_{99} concentration (100 µg/mL for B3C10SA and B3C106SLN, 500 µg/mL for BTA3C10SA, 3 µg/mL for B6C11S) were incubated for 1 hour at 37°C. Serial dilutions of the virus-material complex together with the non-treated control were conducted and transferred onto the cells. After 1 hour, the mixture was removed and the fresh medium was added. The next day, viral titers were evaluated with ICC assay as described above.

Inhibition assay against HIV-1 (YU-2)

TZM-bl cells were seeded at the density of 10k/well in D-10 medium (DMEM medium with 10% FBS, 1%Pen/Strep) in 96-well plates and incubated at 37°C, 5% CO_2 overnight. Serial dilutions of the tested compounds were prepared in DMEM medium and mixed with HIV-1(YU-2, MOI= 0.035). The mixtures were first incubated for 30min at 37°C, and then applied to TZM-bl cells. After 6h incubation, the inoculum was removed and cells were washed with 1x PBS and then incubated with 200uL D-10 medium for 48h at 37°C, 5% CO_2. Luciferase expression was measured by Luciferase Assay System protocol (Promega). The viral inhibition

percentage was calculated as the ratio of the luminescence of treated group and that of control group.

Inhibition assay against EBV and KHSV

GFP-EBV B95-8 (EBV) was produced in HEK293 cells and GFP-KSHV (KSHV) was produced in BrK.219 cells as previously described[3,4]. EBV and KSHV were pre-incubated with 100 µL of the corresponding concentrations of the antiviral compounds for 1h at 37 °C. Upon incubation, the EBV-antiviral mixtures were added on Raji cells and the KSHV-antiviral mixtures were added on HEK293T cells at MOI 0.1. Viral infection was evaluated 2 days later by flow cytometric analysis of GFP positive cells on FACSCanto II (BD Biosciences).

Cytotoxicity Assay on Vero, Vero-E6 and MDCK cells

Cytotoxicity assay was performed on Vero/Vero-E6/MDCK cell line with MTS assay. Cells were plated 12 h before in DMEM medium containing 10% FBS and 1% penicillin/ streptomycin with a seeding density of 2×10^4 per well in a 96-well plate. Tested compounds were serially diluted in the same medium used in the infectivity assay and added to the cells and incubated for 24 h. Then, cells were washed twice with PBS, followed by addition of 10 µL of MTS reagents together with 90 µL of DMEM medium to incubate for 4 h. The cell viability was checked by absorbance at 490 nm with plate reader.

Cytotoxicity Assay on TZM-bl Cells

TZM-bl cells were pre-plated 24 h in advance at the density of 10^4 cell/well in D-10 medium in 96-well plates. Serial dilutions of the tested compounds in DMEM medium were performed and incubated with the cells for 6 h at 37°C, 5% CO_2. The medium was then removed and cells were washed once with 1x PBS, followed by a 48 h incubation with D-10 medium at 37°C, 5% CO_2. 100 µL of 1:10 WST-1 solution in DMEM was added in each well. After a 4h incubation, the absorbance was measured at 450 nm with plate reader.

Cytotoxicity Assay on Raji and HEK293T Cells

100 µL of the corresponding concentrations of the antiviral compounds were incubated for 1 h at 37 °C to mimic the viral inhibition conditions. Upon incubation, the antiviral compounds were added on Raji cells and on HEK293T cells. Cell viability was determined 2 days later by flow cytometric analysis using the Zombie NIR fixable viability kit (BioLegend) on FACSCanto II (BD Biosciences).

5. Synthesis of multivalent sialic acids B3TAC10SA

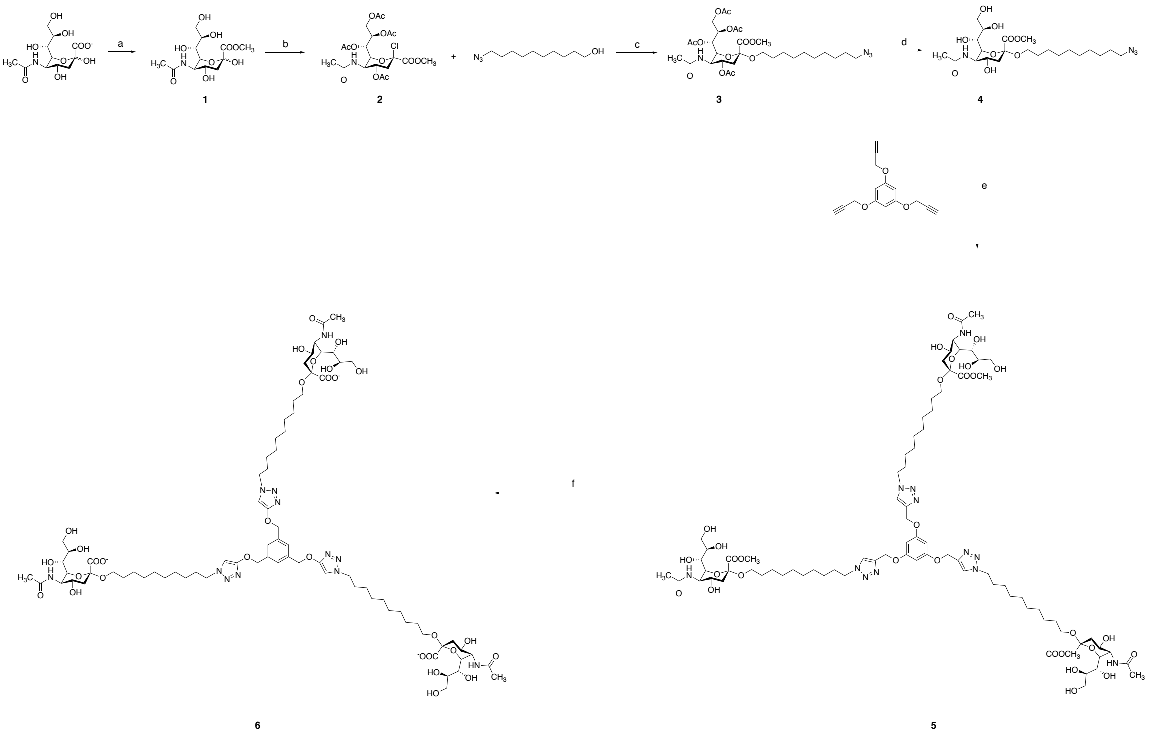

Scheme 1. Synthetic route of B3TAC10SA. Reaction conditions: a. Dowex H+ resin, dry methanol, overnight. b. acetyl chloride, methanol, 2 days. c. Ag_2CO_3, dry DCM, 4 Å molecular sieves, in dark, 2 days. d. CH_3ONa, methanol, pH = 9-10, overnight. e. $CuSO_4$, sodium ascorbate, THF/H_2O, 2 days. f. LiOH, methanol, overnight.

Sialyl chloride **2** was synthesized following a reference[5]. Briefly, 5 g N-acetyl neuraminic acid (16.18 mmol) and 1.25 g Dowex 50WX 4 resins were dispersed with 375 mL of dry methanol, and stirred overnight at room temperature. The resins were filtered off the next day and the filtrate was concentrated and dried to get the crude product compound **1** (5.01 g, yield 96%), as a light-yellow powder. 3 g of crude compound **1** (9.29 mmol) and 90 mL of acetyl chloride were mixed, followed by an addition of 1.8 mL dry methanol dropwisely with stirring. After stirring at room temperature for 5 days, the mixture was evaporated and the residue was first purified by silica gel chromatography (pure ethyl acetate, Rf =0.5) and then recrystallized from diethyl ether/petroleum ether to obtain a white powder, compound **2** (3.42 g, 6.71 mmol, 72%). Sialyl chloride **2** (2.02 g, 3.95 mmol) and 10-azido-1-decanol (1 g, 5.02 mmol, 1.3 eq) were dissolved in 16 mL extra-dry DCM and 1.33 g 4 Å molecular sieves was added. After stirring for 2 h, silver carbonate (2.19 g, 7.94 mmol, 2 eq) was added and the reaction mixture was stirred for 2 days at room temperature under Argon with exclusion of light. The solid was filtered through Celite, washed with DCM and the filtrate was evaporated to dryness to obtain crude glycoside **3**. Then, the crude product **3** was dissolved in 15 mL of dry methanol, and the pH of the mixture was adjusted to 10 by adding an appropriate amount of sodium methoxide. After stirring overnight, the mixture was evaporated and a silica gel chromatography (DCM: Methanol gradient from 50:1 to 40:1) gave compound **4** (82.0 mg, 0.163 mmol, 4.1 % two steps in total) as a white solid. ^{1}H NMR (400 MHz, D_2O) δ 3.99 – 3.57 (m, 9H, $COOCH_3$, sialic acid-H), 3.39 (d, J = 8.0 Hz, 1H, sialic acid-H), 3.28 (t, J = 6.8 Hz, 2H, CH_2-N_3), 2.73 (d, J = 9.7 Hz, 1H, 3-H_e), 2.06 (s, 3H, $COCH_3$), 1.83 (t, J = 12.1 Hz, 1H, 3-H_a), 1.67 – 1.09 (m, 18H, alkyl H). ^{13}C NMR (101 MHz, D_2O) δ 175.06, 170.02, 99.08, 73.00, 71.00, 68.08, 67.28, 64.26, 62.78, 53.17, 52.06, 51.21, 39.92, 29.57, 29.54, 29.44, 29.20, 28.79, 26.73, 25.90, 22.15.

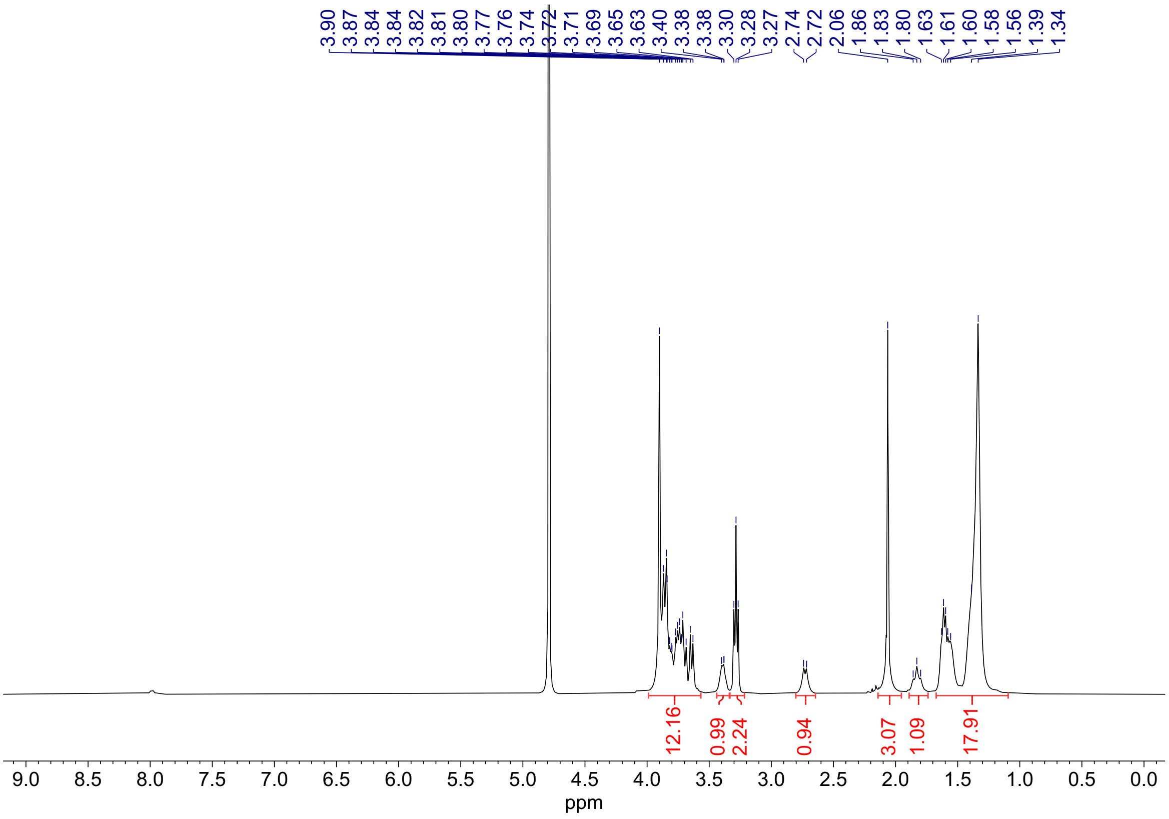

Figure S94. ^{1}H-NMR (D_2O, 400 MHz) of compound **4**

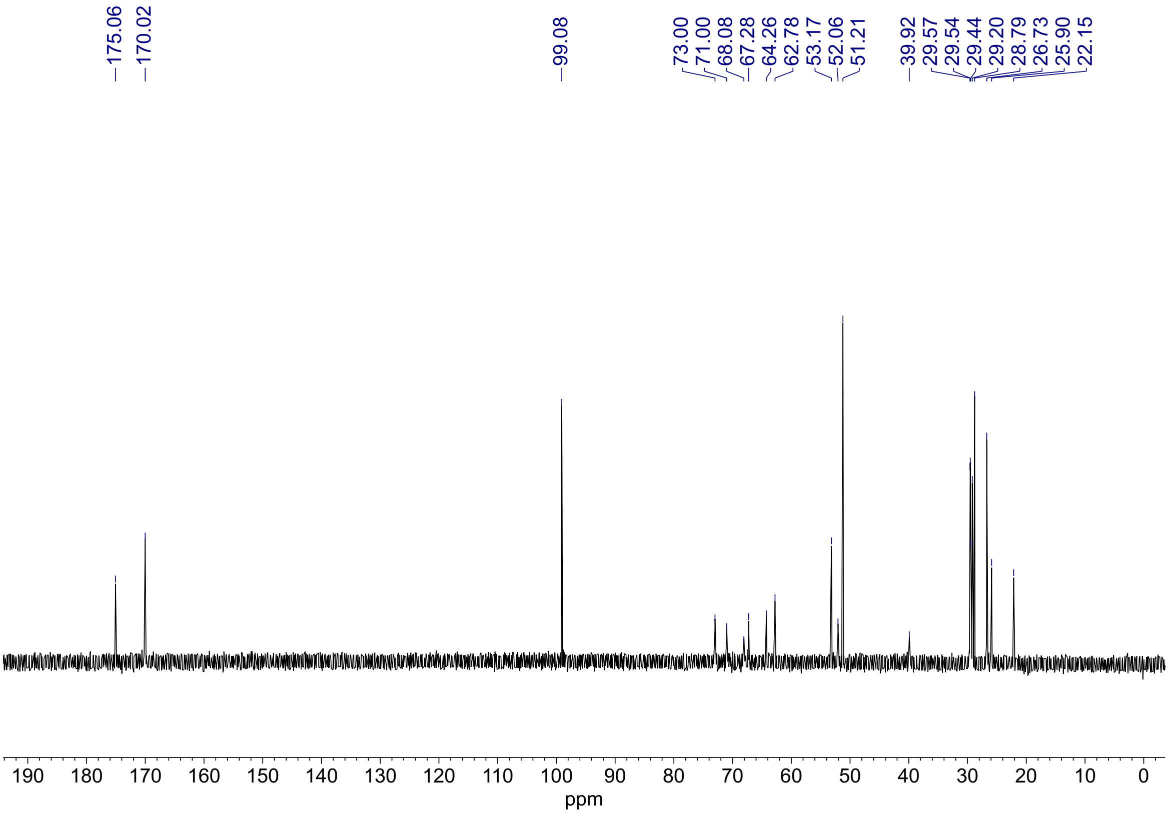

Figure S95. ^{13}C-NMR (D_2O, 101 MHz) of compound **4**

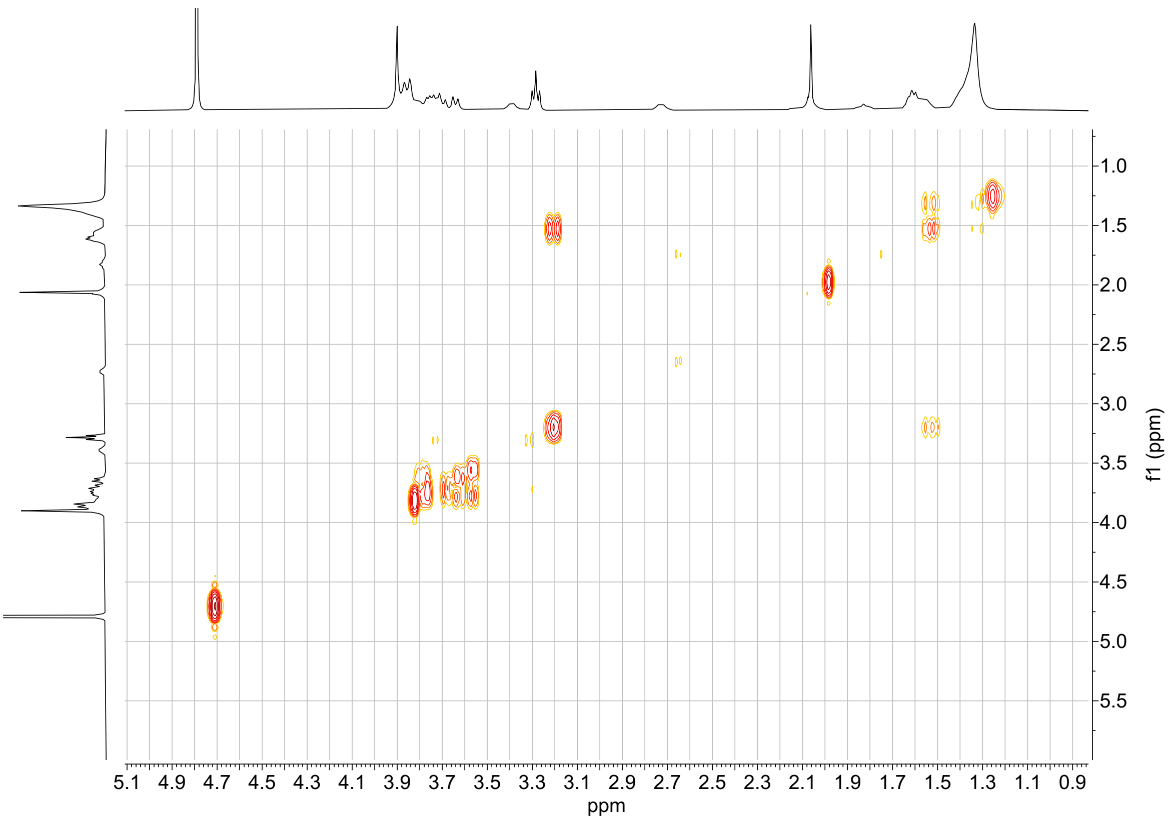

Figure S96. HH-COSY (D_2O, 400 MHz) of compound **4**

Compound **4** (20 mg, 0.04 mmol, 1.22eq per triple bond), anhydrous $CuSO_4$ (0.638 mg, 0.004 mmol, 0.1 eq per triple bond) were dissolved in 500 µL H_2O. 1,3,5-Tris(2-propynyloxy)benzene (2.64 mg, 0.011 mmol, 1 eq) was dissolved in 100 µL THF, and then mixed with the above aqueous solution, followed by adding sodium ascorbate (7.9 mg, 0.04 mmol, 10 eq per triple bond). After stirring at room temperature for 24 h, the mixture was evaporated to dryness and a silica gel chromatography (DCM: methanol gradient 7:1 to 3:1, Rf = 0.3 at DCM: Methanol= 3:1) gave compound **5** (17.4 mg,0.009 mmol, 90%). Compound **5** (17.4 mg,0.009 mmol, 1 eq) and LiOH (2.3 mg, 0.093 mmol, 9 eq) were dissolved in 1.25 mL water- methanol (4/1) mixture. After stirring for 24 h, the solution was dried and a Sephadex LH-20 column was applied to purify the mixture to obtain 3.6 mg compound **6** (0.002 mmol, 23%), B3TAC10SA. ^{1}H NMR (400 MHz, D_2O) δ 7.93 (s, 1H, Ar-H), 6.26 (s, 1H, =CH-N), 5.15 (s, 2H, Ar-CH_2), 4.36 (t, J = 6.5 Hz, 2H, CH_2-N_3), 3.92 – 3.56 (m, 8H, NeuAc-H, alkyl H), 3.42 (q, J = 7.4 Hz, 1H, NeuAc-H), 2.74 (dd, J = 12.4, 4.6 Hz, 1H, 3-H_e), 2.04 (s, 3H, $COCH_3$), 1.85 – 1.71 (m, 2H, alkyl H), 1.63 (t, J = 12.2 Hz, 1H, 3-H_a), 1.48 (t, J = 7.2 Hz, 2H, alkyl H), 1.14 (d, J = 49.6 Hz, 12H, alkyl H). HRMS (standard-ESI/LTQ-Orbitrap) m/z: $[M]^{-3}$ Calcd for $C_{78}H_{123}N_{12}O_{30}^{-3}$ 569.2828; Found 569.2827.

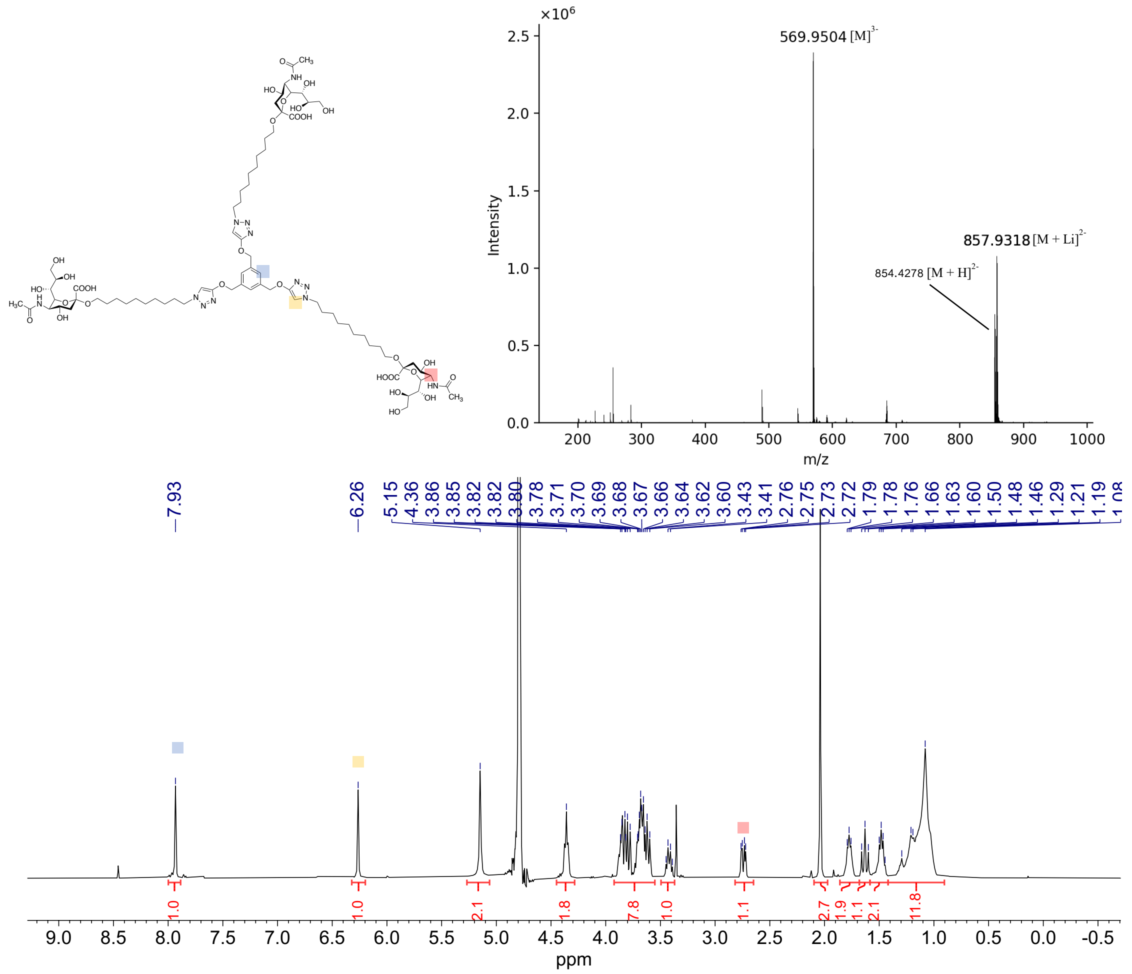

Figure S97. MS and ^{1}H-NMR of B3TAC10SA. In ^{1}H-NMR, Integral of aromatic proton/triazole proton/sialic aromatic proton is 1:1:1, indicating every benzene ring was modified with 3 linkers and sugars. In MS, only target molecule was observed.

B3C106SLN and B3C10SA

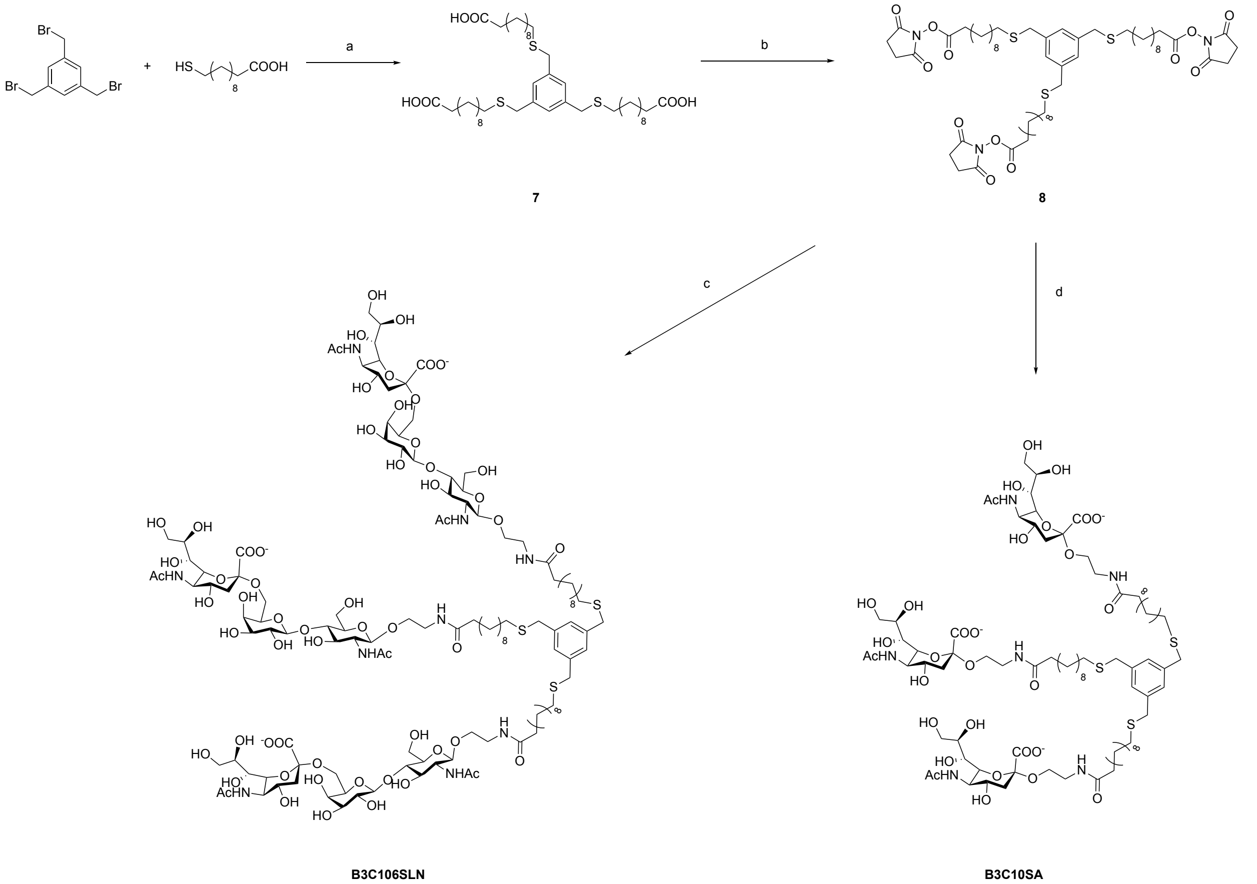

1,3,5-tris(bromomethyl)-benzene (188.7 mg, 0.53mmol), 11-Mercaptoundecanoic acid (873.4 mg, 4 mmol) and sodium methyl oxide (432 mg, 8 mmol) were added into 17 mL of anhydrous DMSO. Anhydrous methanol was added to the mixture until all the solutes dissolved. The reaction was stirred at 70 °C under Argon for 48 h. 200 mL of acetonitrile was then added to the reaction mixture and the precipitate was collected and redissolved in 1 M HCl, extracted with dichloromethane for three times. The combined organic phase was dried over Na_2SO_4, and the filtrate was concentrated in *Vacuo* then purified by silica gel column chromatography (dichloromethane/ methanol 100/1-10/1) to afford crude compound **7** (215 mg, 53%) as white solid. Compound **7** (0.100 g, 0.11 mmol) was dissolved in 10 mL of DMF and stirred for 5 min at room temperature, followed by addition of EDC (0.131 g, 0.68 mmol) and NHS (0.079 g, 0.68 mmol). After stirring at room temperature for 24 h, the reaction mixture was directly concentrated in *vacuo* to remove DMF, then the crude was diluted in CH_2Cl_2 and washed successively with aq. Na_2CO_3. The combined organic layer was dried over Na_2SO_4, and the filtrate was concentrated in *Vacuo* and then purified by silica gel column chromatography (acetone / toluene 3/8) to afford compound **8** (0.110 g, 80 %) as a white solid. ^{1}H NMR (400 MHz, $CDCl_3$) δ 7.12 (s, 3H, Ar-H), 3.66 (s, 6H, Ar-CH_2-), 2.87 – 2.79 (m, 12H, N-CO-CH_2), 2.59 (t, J = 7.5 Hz, 6H, CO-CH_2), 2.39 (t, J = 7.4 Hz, 6H, S-CH_2), 1.79 – 1.22 (m, 48H, C-CH_2-C). ^{13}C NMR (101 MHz, $CDCl_3$) δ 169.19, 168.68, 139.06, 127.93, 36.10, 31.49, 30.95, 29.42, 29.30, 29.26, 29.22, 29.07, 28.91, 28.77, 25.60, 24.58. HRMS (nanochip-ESI/LTQ-Orbitrap) m/z: $[M+H]^+$ Calcd for $C_{54}H_{82}N_3O_{12}S_3^+$ 1060.5061; Found 1060.6863.

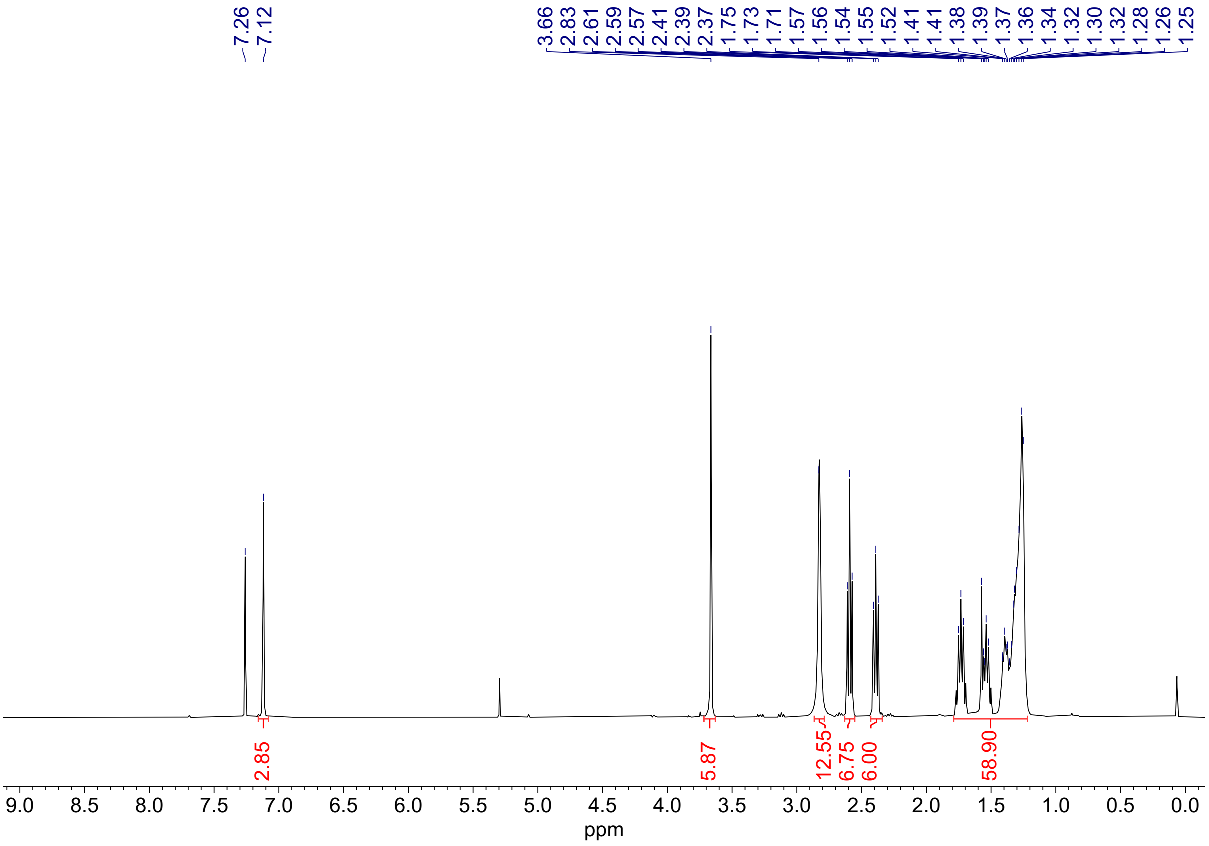

Figure S98. ^{1}H-NMR (D_2O, 400 MHz) of compound 8

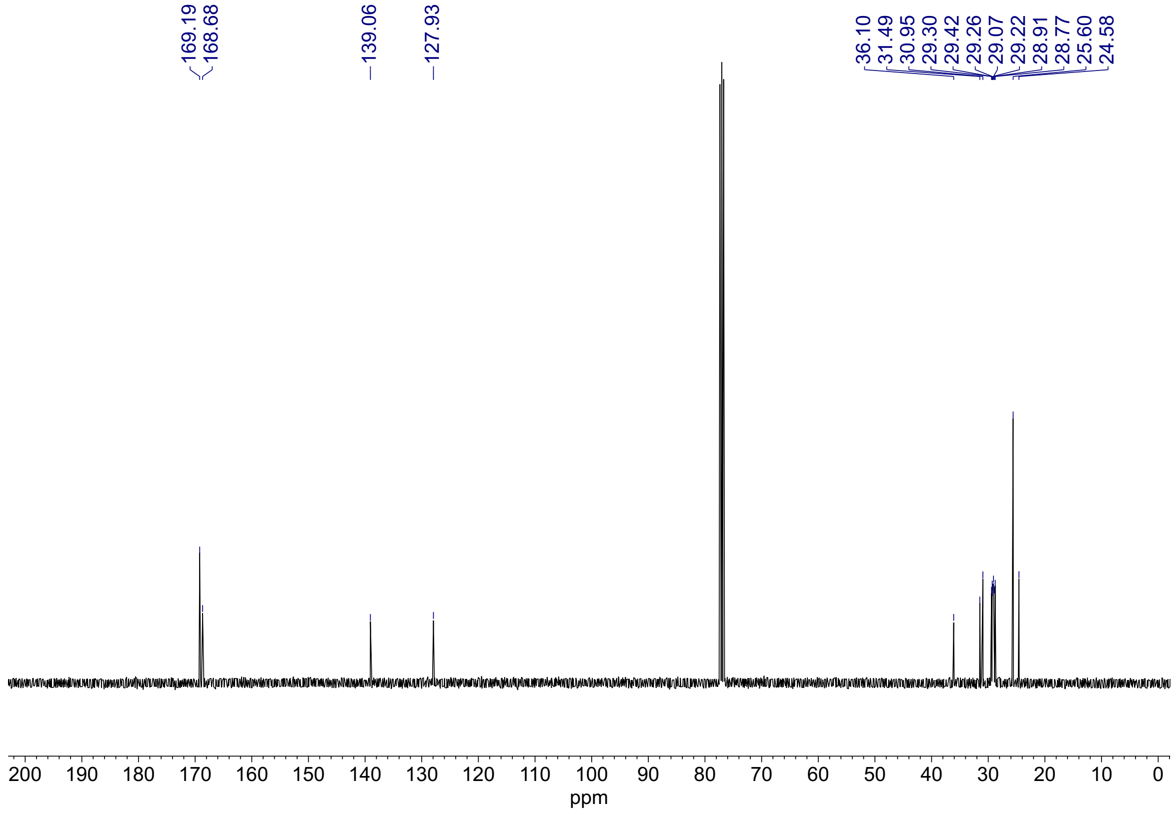

Figure S99. ^{13}C-NMR (D_2O, 101 MHz) of compound 8

A mixture of Neu5Acα(2-6)Galβ(1-4)GlcNAc-β-ethylamine (0.029 g, 0.056 mmol, purchased from Asparia Glycomics) and compound **8** (0.015 g, 0.014 mmol) was dissolved in 10 mL of DMF and stirred for 5 min at room temperature, followed by addition of 3 drops of anhydrous triethylamine and left stirring at room temperature for 24 h. The reaction mixture was directly concentrated in *vacuo* to remove DMF, and the crude was directly purified by reverse phase P2-gel (H_2O,100 %) to afford compound **B3C106SLN**[1] (0.017 g, 42 %) as a white solid.
^{1}H NMR (600 MHz, D_2O) δ 7.06 (s, 3H, Ar-H), 4.53 (d, J = 5.9 Hz, 3H, GlcNAc 1-H), 4.42 (d, J = 7.7 Hz, 3H, Gal 1-H), 4.02 – 3.45 (m, 60H, sugar H, O-<u>CH$_2$</u>-CH$_2$-NH-CO), 3.32 (s, 6H, Ar-CH$_2$-S), 2.65 (d, J = 7.2 Hz, 3H, NeuAc 3-H$_e$), 2.29 (t, 6H, S-CH$_2$), 2.18 (t, 6H, C<u>H$_2$</u>-CO-NH), 2.01 (m, 18H, COCH$_3$), 1.69 (t, J = 12.2 Hz, 3H, NeuAc 3-H$_a$), 1.48-1.21 (m, 54H, alkyl H). ^{13}C NMR (101 MHz, D_2O) δ 175.92, 174.71, 174.15, 173.50, 138.92, 127.97, 103.50, 100.87, 100.08, 80.46, 74.46, 73.55, 72.53, 72.46, 72.40, 72.34, 71.66, 70.69, 68.35, 68.10, 68.00, 63.17, 62.64, 60.27, 54.76, 51.93, 40.06, 39.05, 35.90, 35.57, 30.96, 29.28, 28.99, 25.67, 22.39, 22.10. HRMS (nanochip-ESI/LTQ-Orbitrap) m/z: $[M-2H]^{2-}$ Calcd for $C_{123}H_{206}N_9O_{60}S_3^{2-}$ 1432.6254; Found 1432.6296.

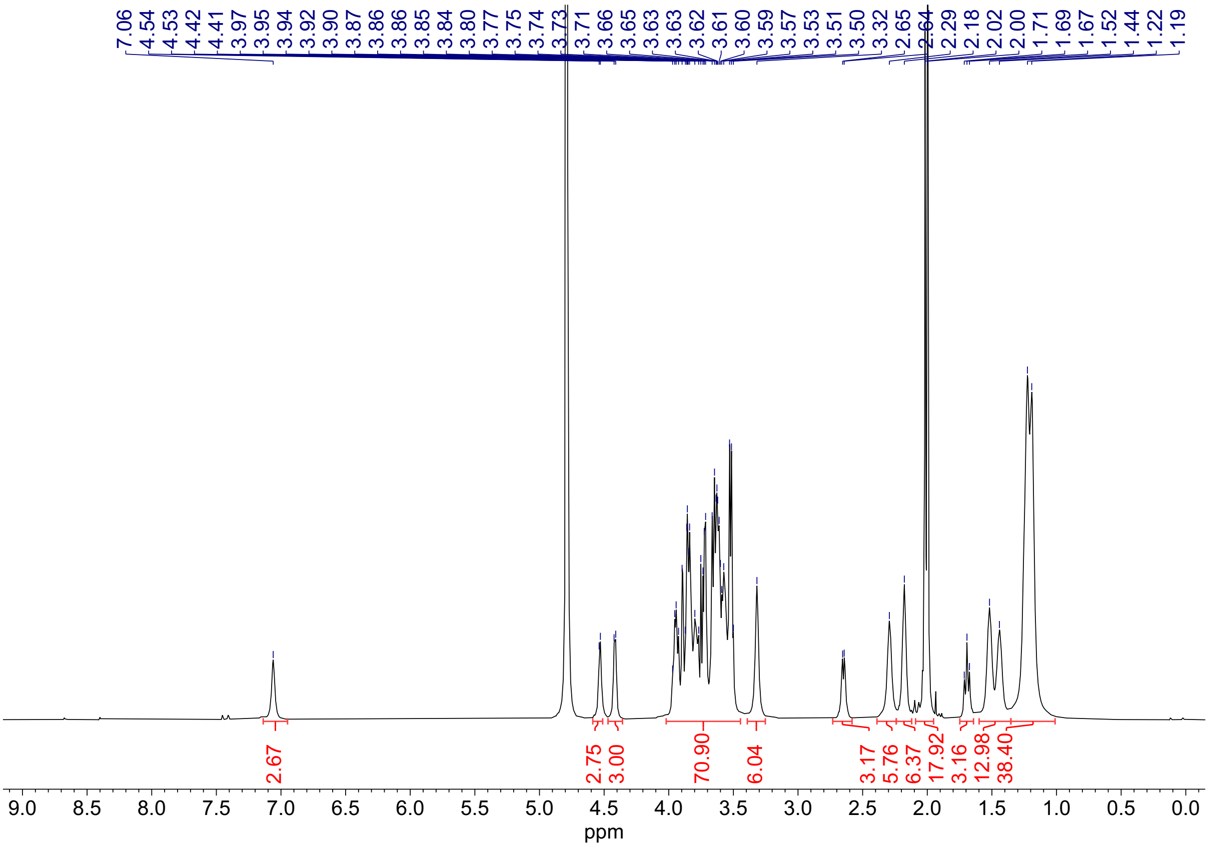

Figure S100. ^{1}H-NMR (D_2O, 400 MHz) of B3C106SLN

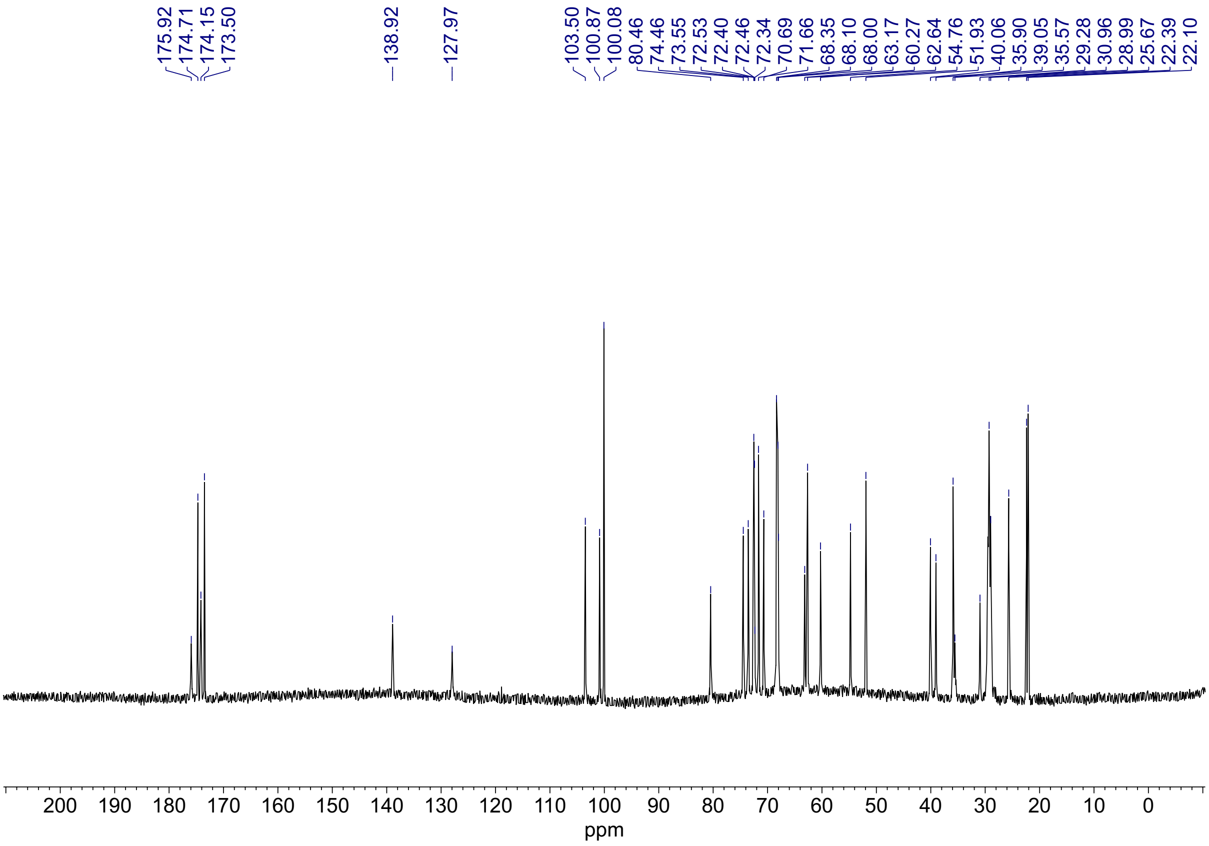

Figure S101. ^{13}C-NMR (D_2O, 101 MHz) of B3C106SLN

A mixture of *N*-Acetyl-2-*O*-(2-aminoethyl)-α-neuraminic acid (0.029 g, 0.064 mmol, purchased from Asparia Glycomics) and Compound **3** (0.018 g, 0.016 mmol) was dissolved in 10 mL of DMF and stirred for 5 min at room temperature, followed by addition of 3 drops of anhydrous triethylamine and left stirring at room temperature for 24 h. The reaction mixture was directly concentrated in *vacuo* to remove DMF, and the crude was directly purified by reverse phase P2-gel (H_2O,100 %) to afford compound **B3C10SA** (0.008 g, 26 %) as white solid. ^{1}H NMR (400 MHz, D_2O) δ 7.11 (s, 3H, Ar-H), 3.98 – 3.46 (m, 21H, sialic acid-H), 3.35 (s, 6H, Ar-CH_2-S), 2.75 (d, *J* = 8.7 Hz, 3H, 3-H_e), 2.35 - 2.26 (m, 12H, alkyl H, S-CH_2, C$\underline{H_2}$-CO-NH), 2.05 (s, 9H, $COCH_3$), 1.69 (t, *J* = 12.0 Hz, 3H, 3-H_a), 1.64 – 1.07 (m, 60H, alkyl H). ^{13}C NMR (101 MHz, D_2O) δ 174.92, 173.52, 160.33, 138.93, 128.07, 100.44, 72.65, 71.51, 68.08, 68.02, 62.65, 62.43, 51.97, 40.32, 39.42, 35.89, 35.37, 31.04, 29.40, 25.66, 22.03. HRMS (nanochip-ESI/LTQ-Orbitrap) m/z: $[M-2H]^{2-}$ Calcd for $C_{81}H_{136}N_6O_{30}S_3^{2-}$ 884.4232; Found 888.4238.

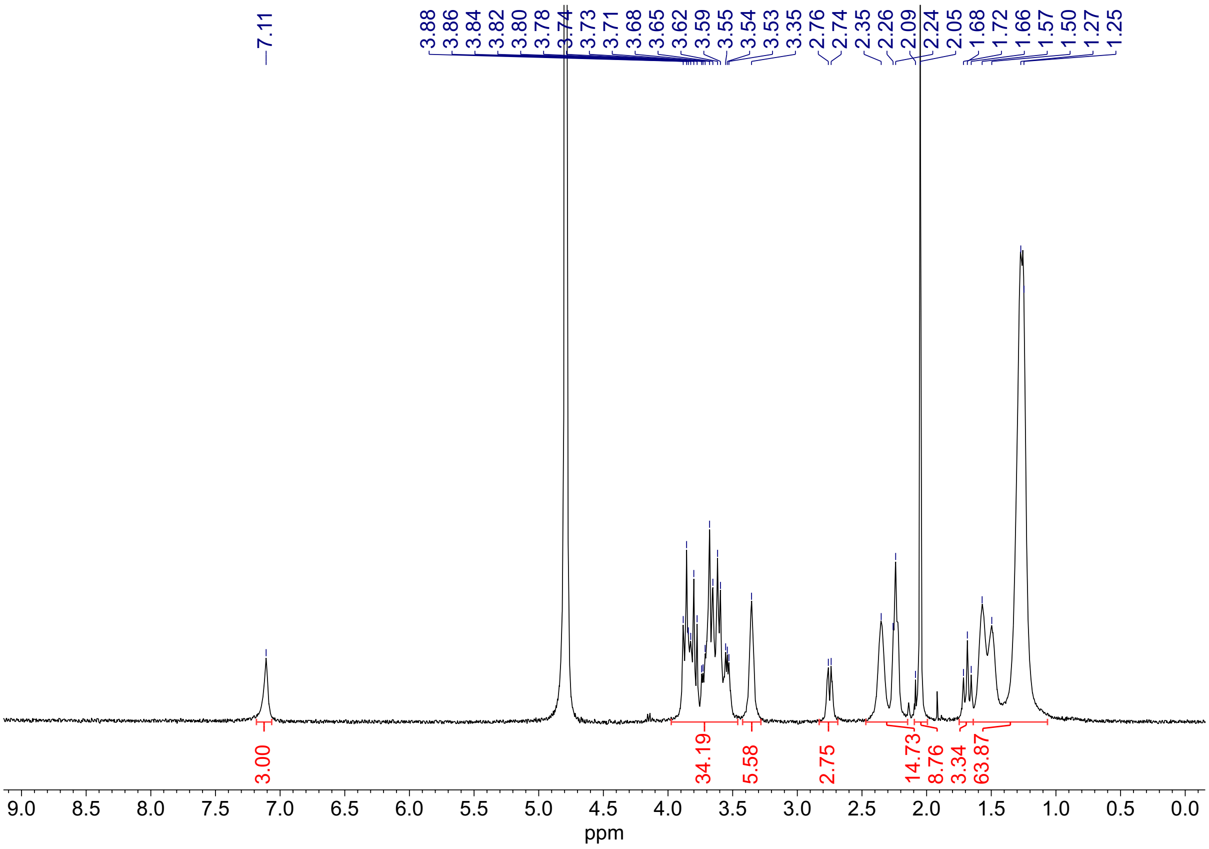

Figure S102. ^{1}H-NMR (D_2O, 400 MHz) of B3C10SA

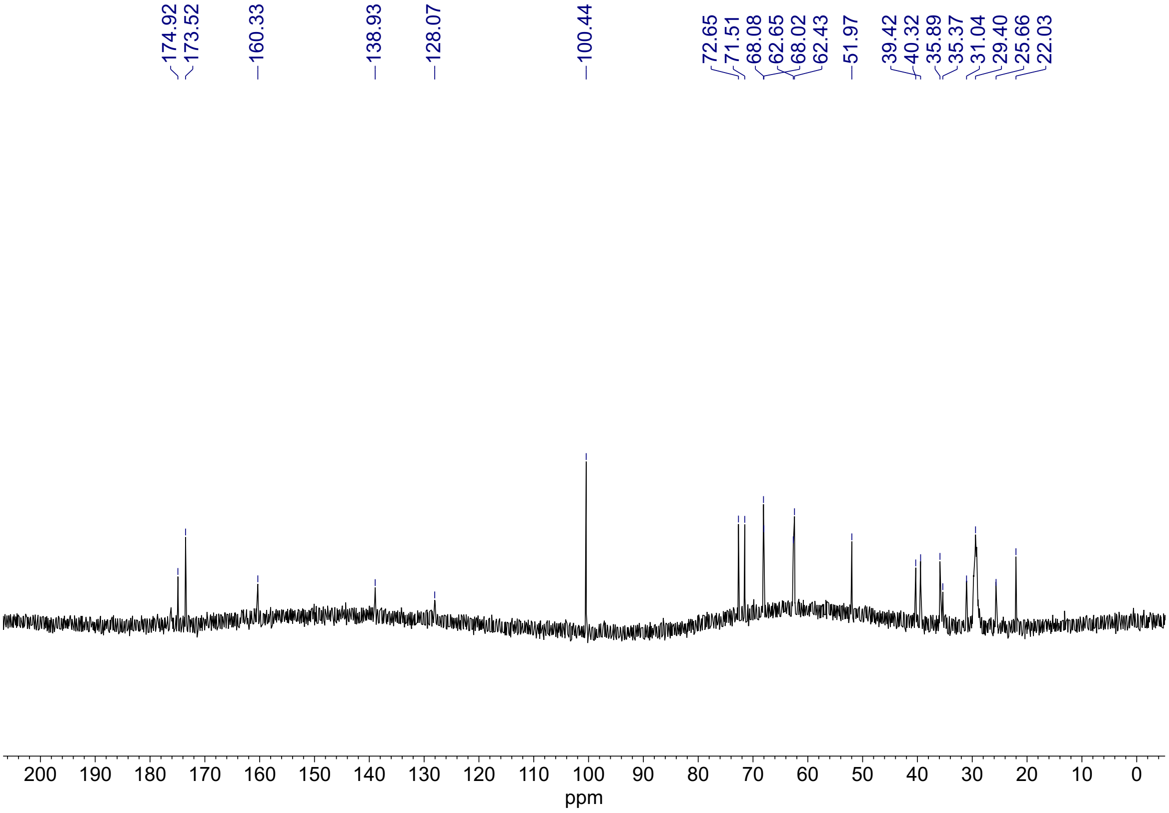

Figure S103. ^{13}C-NMR (D_2O, 101 MHz) of B3C10SA

6. SARS-COV-2 infection model in Syrian hamsters

Treatment regimen

Female hamsters, approximately 5 weeks old and homogeneous in weight, were purchased from Janvier company. Upon arrival at the lab, two experienced veterinarians conducted a clinical examination on the animals, which showed no unusual behavior, skin lesions, or atypical attitude. The animals were housed in a SPF animal facility for a week to mitigate transport stress before being moved to an A3 facility. After 24 h, the ninety 6-week-old hamsters were randomly assigned into six groups and all the groups except one uninfected group were inoculated intranasally in both nostrils with 100 µl 10^6 $TCID_{50}$/mL SARS-CoV-2 Alpha under gas anesthesia. Positive control 250 mg/kg nirmatrelvir in MTBE solvate form (Lucern Chem), solubilized in 0.5% methylcellulose, 2% Tween 80 aqueous solution was dosed orally to the hamsters twice per day for five days starting from 12 hpi. Three groups of infected hamsters received three doses of B6C11S aerosol treatment (B6C11S dissolved in PBS pH=7.4) with the same timing through Oro-Nasal and Respiratory Exposure Systems (ONARES, CH technologies, USA). The exposure time was adjusted to regulate the intake of inhaled doses, estimated to be approximately 6, 8, and 10.6 mg/kg/day, taking into account the aerosol delivery yield, which is calculated by dividing the aerosol generation speed by the minute volume of the hamsters (53.5 mL/min[6]). Hamsters in the untreated control group were given PBS aerosols. The body weight of all the hamsters were monitored twice per day and a subgroup of 3 hamsters in each group were sacrificed at 2, 4, 6, 8 and 10 dpi and the oral swab and lung samples were collected for viral titer measurement. Furthermore, lung samples were subjected to histopathological examination.

Viral RNA copies in the oral swab and lung tissues

RNA was extracted from homogenized hamster lungs or the oral swab with Trizol LS Reagent (Invitrogen) following the manufacturer's recommendations and quantified on a Nanodrop. To generate cDNA, 1 µg of RNA was reverse transcribed using the SuperScrip IV First-Strand Synthesis System (Invitrogen) according to the manufacturer's instructions. At the same time, a synthetic RNA corresponding to the region of interest was reverse transcribed through a 5-point serial dilution. qPCR was performed in a 20 µL volume with 1× PowerUp SYBR Green Master Mix (Applied Biosystems), 500 nM of each primer (ORF1ab-F: CCCTGTGGGTTTTACACTTAA, ORF1ab-R ACGATTGTGCATCAGCTGA), and 2 µl of cDNA. The amplification was carried out with the following cycling parameters: 2 min at 50°C, 2 min at 95°C, 40 cycles of 15 sec at 95°C and 30 sec at 62°C. A dissociation curve was generated after the amplification, and samples were run in triplicate. The concentrations of RNA were determined using the ΔCt to log10 (synthetic RNA concentration) standard curve.

Infectious viral particles in the lungs

Vero E6 cells were seeded onto 96-well plates at a density of 7.5×10^3 cells per 100 µL in growth media (DMEM/FBS10%). The plates were gently rocked for even cell distribution, and after an overnight incubation, cells were visually assessed under a light microscope to confirm uniform distribution and approximately 75% confluency. For the preparation of serial dilutions of lung homogenate, a series of 1:10 dilutions was made. The first tube was filled with 2.0 ml of infection medium (DMEM/FBS2%), followed by six tubes with 1.8 mL of infection medium each. Lung homogenate was vortexed, and 200 µL of the suspension was transferred to the first tube. This process was repeated to achieve serial 1:10 dilutions ranging from 10^{-1} to 10^{-7}. Pipetting of the successive dilutions onto Vero E6 cell monolayers in a labeled 96-well plate

followed. Four negative wells were included on each plate. After removing growth media from each well, 100 µL of lung dilutions were added to infect 4 wells per dilution. Following a 2 h incubation for virus adsorption at 37°C, 100 µL of infection medium was added to each well, and the plates were returned to the CO_2 incubator at 37°C for monitoring cytopathic effects (CPE) over five days. The visualization and calculation of $TCID_{50}$ involved determining the endpoint when CPE readouts were consistent for three separate readings. Titer was then calculated using the Reed and Muench method and expressed as 10^3 $TCID_{50}$/mL in five days in the Vero E6 cell line.

Histopathological examination

After euthanizing the hamsters, the lung tissues are fixed in 10% buffered formalin, followed by dehydration and embedding in paraffin wax. Thin sections are cut using a microtome, stained with Hematoxylin and Eosin (H&E), and examined under a microscope for cellular morphology and histological changes. The lungs were rated A->F, representing increased degenerative and inflammatory lung damage. The following section provides descriptions for each category:

Category A. Morphologically healthy lungs or lungs with extremely mild lesions consisting of broncho-interstitial pneumonia involving less than 5% of the parenchyma, with focal thickening of the interalveolar septa by a mixed infiltrate of inflammatory cells (neutrophils, lymphocytes and plasma cells, monocytes) and, in some cases, type 2 pneumocyte hyperplasia.

Category B. Lung displaying suppurative bronchitis, with the presence of neutrophilic granulocytes, some macrophages and cellular debris both in the bronchial lumen and between epithelial cells.

Category C: Lung displaying suppurative bronchitis accompanied by bronchial and bronchiolar epithelial cell hyperplasia and/or broncho-interstitial pneumonia affecting less than 20% of the parenchyma.

Category D. Broncho-interstitial pneumonia affecting between 20% and 50% of the parenchyma, with or without lesions of suppurative bronchitis and/or a healing process characterized, among other things, by type-2 pneumocyte hyperplasia.

Category E. Broncho-interstitial to interstitial pneumonia involving between 50 and 70% of the parenchyma, whether or not accompanied by a healing process characterized, inter alia, by type-2 pneumocyte hyperplasia.

Category F. Interstitial pneumonia involving more than 70% of the parenchyma, whether or not accompanied by a healing process characterized, inter alia, by type-2 pneumocyte hyperplasia.

Ethics statement

All animal experiments described in this study were reviewed and approved by the Institutional Animal Care and Use Committee of the University of Liège (ethical approval no. 21/2370). The 'Guide for the Care and Use of Laboratory Animals,' prepared by the Institute of Laboratory Animal Resources, National Research Council, and published by the National Academy Press, as well as European and local législations, was followed carefully. Accordingly, the temperature and relative humidity were 21 °C and 45–60%, respectively.

Reference

(1) Xu, R.; McBride, R.; Nycholat, C. M.; Paulson, J. C.; Wilson, I. A. Structural Characterization of the Hemagglutinin Receptor Specificity from the 2009 H1N1 Influenza Pandemic. *J Virol* **2012**, *86* (2), 982–990. https://doi.org/10.1128/JVI.06322-11.

(2) Sawama, Y.; Masuda, M.; Asai, S.; Goto, R.; Nagata, S.; Nishimura, S.; Monguchi, Y.; Sajiki, H. $FeCl_3$ -Catalyzed Self-Cleaving Deprotection of Methoxyphenylmethyl-Protected Alcohols. *Org. Lett.* **2015**, *17* (3), 434–437. https://doi.org/10.1021/acs.orglett.5b00106.

(3) Kalla, M.; Göbel, C.; Hammerschmidt, W. The Lytic Phase of Epstein-Barr Virus Requires a Viral Genome with 5-Methylcytosine Residues in CpG Sites. *J Virol* **2012**, *86* (1), 447–458. https://doi.org/10.1128/JVI.06314-11.

(4) Kati, S.; Hage, E.; Mynarek, M.; Ganzenmueller, T.; Indenbirken, D.; Grundhoff, A.; Schulz, T. F. Generation of High-Titre Virus Stocks Using BrK.219, a B-Cell Line Infected Stably with Recombinant Kaposi's Sarcoma-Associated Herpesvirus. *Journal of Virological Methods* **2015**, *217*, 79–86. https://doi.org/10.1016/j.jviromet.2015.02.022.

(5) Šardzík, R.; Noble, G. T.; Weissenborn, M. J.; Martin, A.; Webb, S. J.; Flitsch, S. L. Preparation of Aminoethyl Glycosides for Glycoconjugation. *Beilstein J. Org. Chem.* **2010**, *6*, 699–703. https://doi.org/10.3762/bjoc.6.81.

(6) Guyton, A. C. MEASUREMENT OF THE RESPIRATORY VOLUMES OF LABORATORY ANIMALS. *American Journal of Physiology-Legacy Content* **1947**, *150* (1), 70–77. https://doi.org/10.1152/ajplegacy.1947.150.1.70.
